# Horizontal Gene Transfer in Five Parasite Plant Species in Orobanchaceae

**DOI:** 10.1093/gbe/evy219

**Published:** 2018-11-08

**Authors:** Tomoyuki Kado, Hideki Innan

**Affiliations:** SOKENDAI, Department of Evolutionary Studies of Biosystems, The Graduate University for Advanced Studies, Hayama, Kanagawa, Japan

**Keywords:** horizontal gene transfer, parasite plants, molecular evolution

## Abstract

We sequenced genomes of five parasite species in family Orobanchaceae to explore the evolutionary role of horizontal gene transfer in plants. *Orobanche minor* and *Aeginetia indica* are obligate parasites with no photosynthetic activity, whereas the other three (*Pedicularis keiskei*, *Phtheirospermum japonicum*, and *Melampyrum roseum*) are facultative parasites. By using reference genome sequences and/or transcriptomes of 14 species from Fabaceae and Poaceae, their major host families, we detected 106 horizontally transferred genes (HGT genes), only in the genomes of the two obligate parasites (22 and 84 for *Oro. minor* and *Ae. indica*, respectively), whereas none in the three facultative parasites. The HGT genes, respectively, account for roughly 0.1% and 0.2% of the coding genes in the two species. We found that almost all HGT genes retained introns at the same locations as their homologs in potential host species, indicating a crucial role of DNA-mediated gene transfer, rather than mRNA mediated retro transfer. Furthermore, some of the HGT genes might have transferred simultaneously because they located very closely in the host reference genome, indicating that the length of transferred DNA could exceed 100 kb. We confirmed that almost all introns are spliced in the current genome of the parasite species, and that about half HGT genes do not have any missense mutations or frameshift-causing indels, suggesting that some HGT genes may be still functional. Evolutionary analyses revealed that the nonsynonymous–synonymous substitution ratio is on average elevated on the lineage leading to HGT genes, due to either relaxation of selection or positive selection.

## Introduction

Horizontal gene transfer (HGT) between plant species has been extensively investigated (reviewed by Richardson and Palmer 2007; Bock 2010; Davis and Xi 2015; Soucy et al. 2015). Whereas many of the detected HGTs so far involve organelles, primarily mitochondria (Richardson and Palmer 2007), there are several recent reports exhibiting evidence for HGTs between nuclear DNA (Bock 2010; Soucy et al. 2015). The close relationship between parasite plants and their host plants can be a good model system for studying nuclear HGT (Davis and Xi 2015). Indeed, most nuclear HGT genes identified thus far are in parasite plants, most likely transferred from their host plants (Yoshida et al. 2010; Xi et al. 2012; Zhang et al. 2013, 2014; Yang et al. 2016).

The first identified nuclear HGT gene in parasite plants was *ShContig9483* in *Striga hermonthica*, in the family Orobanchaceae (Yoshida et al. 2010). It was also shown that this gene has a closely related homolog in the *Sorghum bicolor*, which is known as a host plant of *St. hermonthica*. The authors found that a poly-A-like sequence at the 3′ end of the gene, suggesting that this transfer might have occurred through a reverse transcribed mRNA. This makes sense because parasitic plants form an invasive organ called a haustorium, which interconnects their vasculature with that of their hosts, thereby allowing transfer of nutrients, water, and even mRNAs. (Roney et al. 2007; Westwood et al. 2009; Kim et al. 2014). Two identifications followed in other species in Orobanchaceae (Zhang et al. 2013, 2014). Different from the first case, these two genes have introns at the same locations as their orthologs in the host plants, indicating that genomic DNAs have been directly incorporated in the genomes. Thus, through only three clear demonstrations, it is very difficult to understand which is the major mechanism of HGT, either mRNA- or DNA-mediated transfer, and their relative contributions. To address this question, more comprehensive genome-wide surveys of HGT are needed.

At this moment, there are very few such surveys. One was carried out for *Rafflesia* (Xi et al. 2012). The authors sequenced cDNAs from the parasitic plant, *Rafflesia cantleyi* (Rafflesiaceae), and its obligate host, *Tetrastigma rafflesiae* (Vitaceae), and 47 “putative” HGTs were detected, of which 31 were confirmed by genomic DNA. It was found that about a half of them have introns, suggesting an important role of DNA-mediated HGT. However, this screening is not comprehensive because they analyzed only <10% of transcripts having orthologs in the rice genome that was used as an outgroup. Because of the lack of reference genomes of closely related species, they were not able to precisely confirm the donor of the detected putative HGTs. The genomic DNA sequences used to investigate the presence/absence of introns had a very low coverage, say <×1. It is indicated that data with higher quality and quantity are needed to understand the full picture of HGTs between host and parasite plants. Very recently, Yang et al. (2016) carried out a much more comprehensive genome-wide survey on three parasite species in Orobanchaceae, *St. hermonthica, Phelipanche aegyptica*, and *Triphysaria versicolor*. The authors identified 52 HGTs, most of which were DNA-mediated transfers rather than mRNA-mediated retro transfers.

**Fig. 1.**
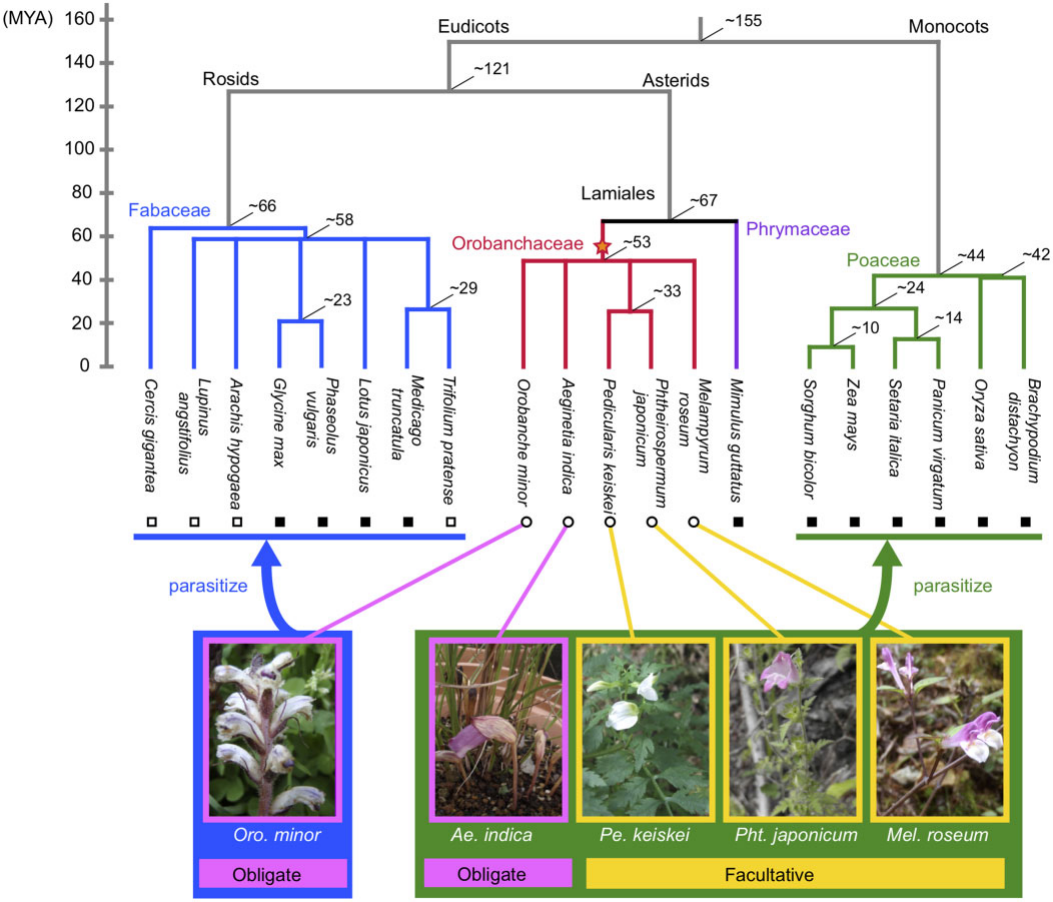
—Overview of the phylogenetic relationship of the five Orobanchaceous parasite species and their potential host species. The speciation times in million years (numbers at nodes) are according to TimeTree (Hedges et al. 2006; Hedges et al. 2015; http://www.timetree.org; last accessed November 6, 2018). The star indicates the origin of parasitism. The open circles are for the five parasite species, for which we sequenced in this study. The filled and open squares, respectively, represent species having full reference genome with annotated CDS information and those having transcriptome shotgun assemblies (TSA) data. See supplementary table S3, Supplementary Material online for the summary of the data used in this study.

We here report a survey of plant HGTs using another five species in the family Orobanchaceae (fig. 1). These five species belong to the same family as the first three documentations of plant HGTs, and also the three species used in the recent genome-wide survey by Yang et al. (2016). It has been suggested that the five species have originated from a single parasitism event, but vary in their host ranges. *Orobanche minor* mainly parasitizes the families Fabaceae, and clovers (the genus *Trifolium*) should be the most preferred host, although *Oro. minor* also parasitizes other families, such as Apiaceae. The other four species mainly parasitize the family Poaceae, and potentially other monocots. The degree of host dependence also differs, from “obligate” to “facultative” parasites (see fig. 1). *Orobancheminor* and *Aeginetia indica* are obligate parasite species with no photosynthetic activity and obtain all their reduced carbon through haustorial connections with their hosts. The other three (*Pedicularis keiskei*, *Phtheirospermum japonicum*, and *Melampyrum roseum*) are facultative parasites; They have the ability of photosynthesis so that they can live autotrophically and reproduce without host contact, while opportunistically parasitize neighboring plants when available. Therefore, it would be intriguing to explore the relationship between the degree of host dependence and the extent of HGT (see Westwood et al. 2010 for a review).

The major difference from Yang et al. (2016) is that we performed evolutionary analyses using multiple species within the two major host families, Fabaceae and Poaceae, from which most HGTs should have occurred. These data allowed us to obtain many kinds of information, such as reconstructing genomic sequence of HGT genes, detecting transfer events that involved multiple genes simultaneously, which have not been explored by Yang et al. (2016). Fabaceae covers the major range of the hosts of *Oro. minor* (shown in blue in fig. 1) and genomic sequences are available for four species and transcriptome shotgun assemblies (TSAs) for another four species, including one species in *Trifolium*, the major host genus. Poaceae includes the host species of the other four parasite species investigated in this study (green in fig. 1), and reference genome sequences for six species are available. It should be noted that the nucleotide identity is ∼85% within each family, making it possible to perform homology search on genomic sequences at the DNA level. In addition, the genomic sequence of *Mimulus guttatus* in Phrymaceae, one of the closest family to Orobanchaceae, is also available, which plays an important role as an outgroup. Thus, the availability of genomic sequences of a number of relative species both in parasite and host families facilitates to identify the time of HGT in a fine scale, making it possible to explore how transferred genes have evolved after the drastic change in the genomic environment.

Another advantage is the reliability of HGTs detected in this study. The major reason is that we here focus on the parasite species that have acquired parasitism relatively recently (roughly 53–67 Ma according to TimeTree, Hedges et al. 2006; Hedges et al. 2015). Coincidentally, the potential host species investigated in this study belong to either Fabaceae or Poaceae, both of which have their common ancestors around the same time as the acquisition of parasitism (fig. 1). Therefore, horizontally transferred genes after the acquisition of parasitism should be detected by phylogenetic analysis of multiple species in Fabaceae and Poaceae with high confidence with minimizing false positive rates. In addition, divergence at synonymous sites within each family is informative to confirm phylogenetic relationship because synonymous divergence does not exceed 1 (Pfeil et al. 2005; Gossmann et al. 2010). Thus, parasite species in Orobanchaceae and their potential host families, Fabaceae and Poaceae, can serve as an excellent model for exploring the evolutionary role of HGTs.

## Materials and Methods

### Plant Samples, DNA and RNA Extraction and Illumina Sequencing

Cultivated individuals of *Ae. indica* were obtained from a gardening shop (http://www.nihonkaki.com/; Last accessed November 6, 2018), whereas plant samples of the other four parasite species were collected from the wild. See supplementary table S1, Supplementary Material online for the sampling locations.

Total RNA was extracted from several tissues (tissues from a few individuals were pooled) for each species (supplementary table S1, Supplementary Material online) by using RNeasy Plant Mini kit (Qiagen) following the manufacturer’s instructions. Genomic DNA was extracted from stem or leaves (a single individual per species) using DNeasy Plant Mini kit (Qiagen) following the manufacturer’s instructions.

For RNA sequencing, tissue-specific libraries were constructed for each species, and the libraries were sequenced separately by using the Illumina HiSeq2000 sequencing platform. Short reads of mRNAs (paired-end reads of 90 bp with an insert of 200 bp) were obtained from 1 to 3 libraries for each species (supplementary table S1, Supplementary Material online). We also sequenced genomic DNA of the five parasite species using the Illumina HiSeq2000 sequencing platform (paired-end reads of 90 bp with an insert size of 500 bp; supplementary table S2, Supplementary Material online).

### De Novo Assemble of Transcriptome Data

The short-read data of mRNAs were first de novo assembled by using Oases (Schulz et al. 2012) with the following setting:

python oases_pipeline.py -m 31 -M 61 -s 6 -o singleEnd -d “-short data/filename.fa” -p “-cov_cutoff 10 -min_pair_count 5 -min_trans_lgth 250”

The output is a list of loci and each locus consists of variable possible transcripts. We obtained sequences of roughly 20,000–30,000 loci per species (supplementary table S1, Supplementary Material online). As there are ∼28,000 annotated genes in *Mim. guttatus*, it can be considered that our mRNA short-read data roughly cover at least three quarters of the total coding genes. These assembled sequences were screened for HGTs (see below).

### Detecting HGTs by Assembled mRNA Data

**Fig. 2.**
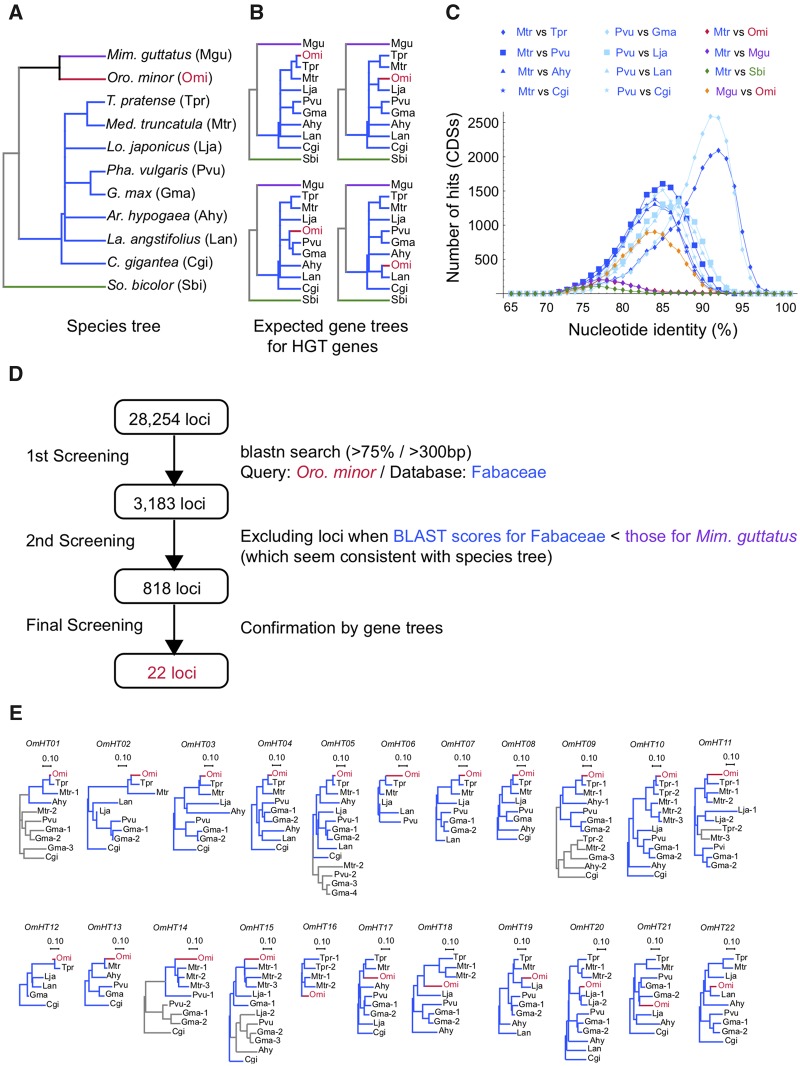
—Summary of the screening process for *Orobanche minor*. (*A*) Species tree of *Oro. minor* and eight species in Fabaceae (blue) together with two species used as outgroups (*Mimulus guttatus* and *Sorghum bicolor*). (*B*) Typical gene trees expected for HGT genes. (*C*) Distributions of nucleotide identity between species. (*D*) Screening scheme. (*E*) Gene trees for the 22 HGT genes, labeled from *OmHT01* to *OmHT22*. The branch lengths in the trees are based on pairwise distance at synonymous sites with the Jukes–Cantor correction. Bootstrap values (>75%) are shown on branches. Red and blue branches are for the parasite (*Oro. minor*) and its homologs in Fabaceae. When paralogs were detected in Fabaceae, their lineages are shown in gray.

In order to detect horizontally transferred genes (referred to as HGT genes), we screened de novo assembled mRNAs of the five parasite species. We here use the screening process of HGT genes from Fabaceae to *Oro. minor* as an example to explain our procedure for detecting HGTs (fig. 2). The Oases software identified 28,254 loci for this species, and for each of them, we BlastN-searched their homologs in the coding sequences (CDSs) or TSAs in the eight potential host species in Fabaceae (blue in fig. 1). Figure 2*A* illustrates the species tree of *Oro. minor* and its host species, together with *Mim. guttatus* as a close relative of *Oro. minor*. For detecting HGT genes, we aimed to search for genes that have trees where *Oro. minor* is more closely related to the host species than *Mim. guttatus* (four examples are illustrated in fig. 2*B*). This strategy can identify HGTs that occurred since the origin of the eight host species (∼66 Ma). Because it is estimated that the acquisition of the parasitism occurred roughly 53–67 Ma, we expected that most HGT events after the parasitism should be detected by this strategy.

We screened *Oro. minor* loci that have >75% identity in a >300 bp region with at least one of the eight Fabaceous species. This BlastN criteria was determined according to the density distributions of nucleotide identity between species in figure 2*C*. It is shown that ∼98% have >75% identity in any pair within Fabaceae, so that our criteria should be suitable to cover most HGT events after the parasitism. We found that 3,183 loci (out of 28,254) satisfied this BLAST criterion, which were subject to further screening processes to identify candidates of HGTs (summarized in fig. 2*D*. See also supplementary table S4, Supplementary Material online). We first screened out obvious false positives due to high conservation. It is predicted that for highly conserved genes, the homology between *Oro. minor* and *Mim. guttatus* is much higher than that between *Oro. minor* and the eight host species. According to this prediction, we were able to screen out 2,365 false positives due to high conservation, and 818 candidate loci remained. We selected loci showing >75% identity in >300 bp with at least two host species, for which we constructed an NJ tree by using MEGA 7.0 (Kumar et al. 2016). In order to screen for loci at which the parasite gene locates within the clade of the host family (Fabaceae), we screened out loci with the external branch leading to the parasite gene longer than twice of the longest branch between host species among the host family. We then screened for loci with Ks < 1 between the parasite gene and the most closely related host gene. Ks < 1 roughly corresponds to a divergence of 100 Ma, which predates the ancestor of Fabaceae. This screening process resulted in 22 loci exhibiting strong evidence for HGTs for *Oro. minor* (supplementary table S4, Supplementary Material online). We also repeated the same process by reconstructing ML trees, and obtained the same results (not shown).

Figure 2*E* shows the gene trees for all 22 loci (labeled from *OmHT01* to *OmHT22*. See supplementary table S5, Supplementary Material online for a list of genes used for the tree construction). The branch lengths in the trees are based on pairwise distance at synonymous site (with the Jukes–Cantor correction). The values of divergence at nonsynonymous site (Ka), those at synonymous site (Ks), and their ratio (Ka/Ks) between parasite and the most closely related host species are summarized in supplementary table S6, Supplementary Material online. The Ks values ranged from 0.037 to 0.572 (0.192 on average), as expected from the relative recent origins of this family. This range of Ks can be considered to reflect the divergence times fairly well, while is quite difficult if Ks exceeds 1 where the variances of divergence times are large.

For 21 of the 22 HGT genes (excluding *OmHT16*), *Oro. minor* is placed within the gene tree of the family Fabaceae, which can be considered as a very strong evidence for HGT. The exception is *OmHT16*, for which we found its homolog only from two species. Nevertheless, we consider that *OmHT16* shows strong evidence for horizontal transfer because the time to their common ancestor should be roughly 20 Ma according to divergence at synonymous sites (Ks = 0.181), which strongly indicates that the transfer event was younger the origin of the family Fabaceae. Note that, according to Pfeil et al. (2005), the median Ks between *Medicagotruncatula* and *Glycinemax* is 0.57, which roughly represents the largest divergence within the family Fabaceae.

A similar strategy was also applied to the four parasite species that parasitize Poaceae (shown in green in fig. 1). By using the same parameters and cutoff values, we found 84 loci in *Ae. indica* with strong evidence for HGTs (summarized in supplementary fig. S1, Supplementary Material online), where the Ks values ranged from 0.011 to 0.575 (0.207 on average). Note that Gossmann et al. (2010) reported that the average Ks between *Ory. sativa* and *So. bicolor* is 0.78, which reflects the largest divergence within the family Poaceae.

We found no loci for the other three facultative parasite species (supplementary table S4, Supplementary Material online). Furthermore, as a negative control, we performed the same screening for *Oro. minor* with the six Poaceous species and the other four parasites with the eight Fabaceous, and found no loci with strong evidence for HGTs (supplementary table S4, Supplementary Material online).

Although we focused on HGTs to the five parasite species from their potential hosts, this strategy also works for identifying HGTs in the opposite direction because they results in a gene tree that is inconsistent with the species tree. HGTs in this direction have been reported for mitochondria genes, but not for nuclear genes (Mower et al. 2004; Richardson and Palmer 2007). In our data, we were not able to find such HGT genes.

### BlastN and TBlastX Searches for Homologs of the HGT Genes

**Fig. 3.**
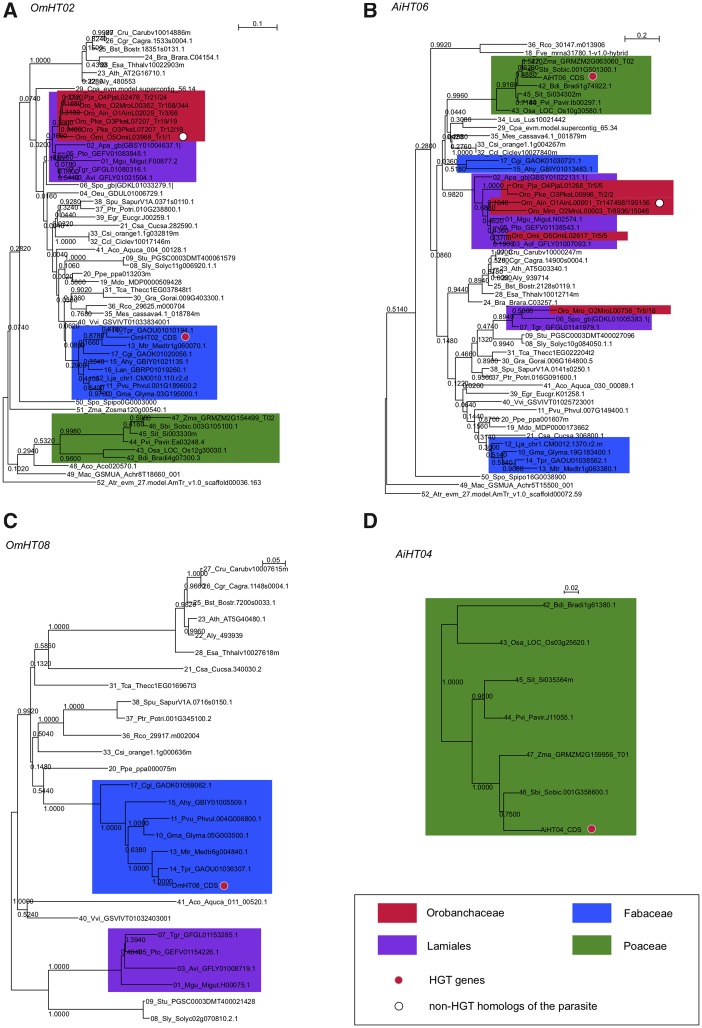
—Four representative gene trees of the HGT genes with their homologs in 52 angiosperm species. See the text for details.

In order to explore the evolution of the 106 HGT genes, we searched for their homologs in 52 angiosperm species with genome sequence data or TSA data available. See supplementary table S3 and figure S2, Supplementary Material online for the species used. We BlastN- and TBlastX-searched homologs for each of our 106 HGT genes, and the genes with highest bit scores in each species are summarized in supplementary table S7, Supplementary Material online. In most cases, we found homologs across angiosperm, and ML trees were reconstructed by using MEGA 7.0 with the HKY+gamma model and all (1st, 2nd, and 3rd) sites in codons (fig. 3 and supplementary fig. S3, Supplementary Material online). We excluded genes in which bit scores were less than a half of those in the host species.

### Genomic DNA Coverage and Reconstruction of Genomic Regions of HGT Genes

The short reads of DNA sequences of the parasite species were used to evaluate DNA coverage in order to rule out possibility of DNA contamination from host plants and to reconstruct the genomic regions encompassing the detected HGTs.

Reconstruction of genomic sequences were carried out based on the concatenated coding (i.e., exon) sequences of the 106 HGT genes. See supplementary figure S5, Supplementary Material online for details. In short, in order to fill a gap between a pair of adjacent exons, we screened for paired-end short-reads and assembled them such that the genomic sequence extends from the both exons. We repeated this process until the extend genomic sequences from the two sides met in the middle.

### Chromosome Locations

In order to examine the possibility of simultaneous transfer of multiple closely-linked genes on a donor’s chromosome, we checked chromosome locations of HGT gene homologs on host plants.

### Evolutionary Analyses

In order to investigate how the evolutionary rate has changed after transfer, we focused on ω (ratio of nonsynonymous to synonymous substitution rates). We applied the branch model in the codeml program (PAML 4 package, Yang 2007).

### GO Enrichment Analysis

In order to identify enriched GO terms in our HGT genes, we performed the SEA (Singular Enrichment Analysis) on agriGO v1.2 (http://bioinfo.cau.edu.cn/agriGO; Du et al. 2010). This analysis can be applied to a reference genome, which is not available for *Oro. minor* or *Ae. indica*. Therefore, reference genomes of their host species were used. We first identified homologs of our HGT genes in a reference genome of host species, and compared with the whole genome GO term annotation data set. Four host species in Fabaceae and five host species in Poaceae have whole genome GO term annotation data sets.

### Data Availability

The raw sequence and assembled sequence data are deposited in the DDBJ Sequence Read Archive under an accession number PRJDB5395. Other edited data used in our analyses (e.g., assembled transcripts for each species, CDSs and transcripts genome DNA assemblies for each HGT gene, and alignments for phylogenetic and molecular evolutionary analyses) are available from the lab webpage (http://www.sendou.soken.ac.jp/esb/innan/InnanLab/software.html; last accessed November 6, 2018).

## Results

### Detecting HGTs by Sequencing mRNA Data

Our screening process resulted in 22 and 84 genes exhibiting strong evidence for HGTs for the two obligate parasites, *Oro. minor* and *Ae. indica* (supplementary table S4, Supplementary Material online), but none in the other three facultative parasites, indicating the host–parasite relationship may be an important factor to determine the rate of HGTs. The proportion of HGT genes in the parasite genome is at least around 0.1% in *Oro. minor* and 0.2% in *Ae. indica* assuming the total number of loci in our Oasis assembly as a proxy of the number of genes on the genome.

As described above, we designed a very careful screening process to identify the 106 HGT genes with minimizing false positives. We further confirmed this by the evolutionary pattern of the 106 HGT genes in a long-term evolution in angiosperm. Supplementary figure S3, Supplementary Material online show ML trees of the 106 HGT genes with their homologs in angiosperm, and four representative patterns are in figure 3. Figure 3*A* shows a typical tree where the tree shape is roughly consistent with the species tree, except for the HGT gene itself. That is, genes in Lamiales (shown in purple), Fabaceae (blue), and Poaceae (green) made distinct clusters. The HGT gene presented by a red circle is the exception. If there was no horizontal event involved, the HGT gene identified in the parasite species should locate close to the Orobanchaceae cluster (shown in red) that belong to the order Lamiales. Figure 3*A* clearly demonstrates that this is not the case because the HGT gene is within the cluster of its potential host family (Fabaceae for *Oro. minor* and Poaceae for *Ae. indica*), rather than within the Lamiales cluster. It should be noted that a homolog was found in the parasite species (white circle), suggesting that the identified HGT gene is an extra-copy derived by a nonvertical event. Figure 3*B* shows a bit complicated tree where gene duplication events were involved such that we observe two distinct clusters for Fabaceae and Lamiales. Nevertheless, the relationship between the HGT genes (red circle) and its homolog (open circle) is clear. Figure 3*C* shows a case where no homolog was detected in Orobanchaceae, but homologs in Lamiales support that the HGT gene was transferred to the parasite genome. Figure 3*D* is an unfortunate case where homologs were found only within the host family. Fifteen *Ae. indica* HGT genes belonged to this pattern. Even for these cases, the tree can be considered as evidence for horizontal transfer, because the origins of HGTs were well inferred on gene trees of host family.

### Origin of HGT Genes

**Fig. 4.**
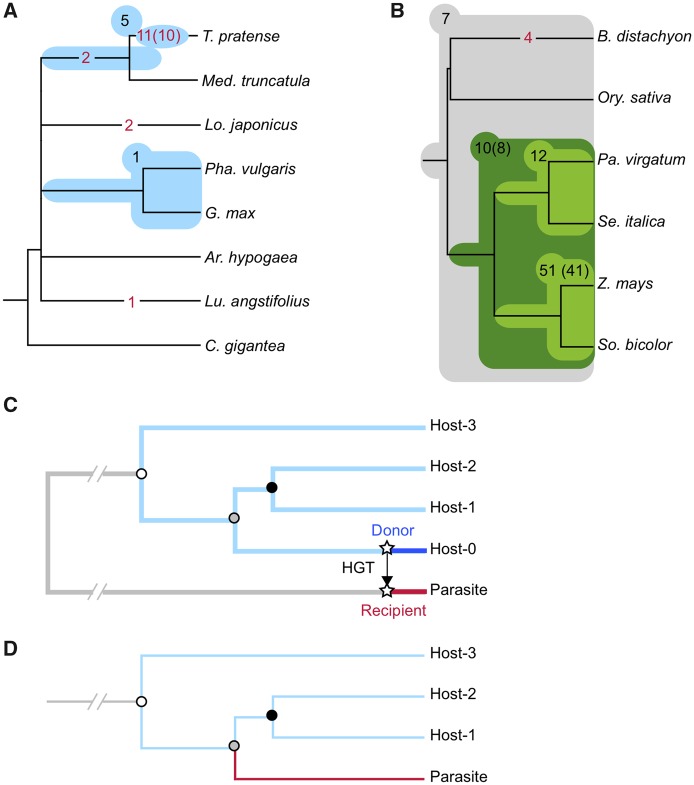
—(*A*, *B*) Putative origins of HGT genes for *Orobanche minor* and *Aeginetia indica*, presented as the number of HGT genes on the branches of host species trees. The numbers in parentheses are those for putative transfer events when multiple HGT genes were transferred simultaneously. See text for details. (*C*, *D*) Illustration of gene trees for understanding the results shown in (*A*) and (*B*).

The gene trees for the 22 and 84 genes provide insight into the origin of these HGT genes (fig. 4). In the 22 *Oro. minor* genes, it is found that 11 genes are most closely related to *Trifoliumpratense*, two are most closely related to *Lotusjaponicus*, and one is most closely related to *Lupinusangstifolius* (red numbers directly placed on each branch in fig. 4*A*). For the 11 HGT genes close to *T. pratense*, we estimated that the number of HGT events is likely 10 because two genes are very closely located on the same chromosome (see below for details). Another two genes are most closely related to the common ancestor of *T. pratense* and *Med. truncatula* (fig. 4*A*). There are six genes (five and one, black numbers in light blue shadows in fig. 4*A*), for which the origin was not able to be placed on a certain branch in the species tree, mainly because of missing data and/or presence of multiple paralogs that confused our inference. Nevertheless, we found that five HGT genes should be placed somewhere after the split of the lineages leading to *T. pratense* and *Med. truncatula*, and one gene should be placed somewhere after the split of the lineages leading to *Pha. vulgaris* and *G. max*. This result indicates that most HGT genes to *Oro. minor* should be from the ancestral lineage of *T. pratense*, one of the well-known hosts of *Oro. minor*. Figure 4*B* shows the result for *Ae. indica*. It looks that most transfer events were from the ancestral lineage leading to *Zeamays* and *So. bicolor*. This lineage includes *Miscanthus sinensis*, the most well-known host of *Ae. indica*.

There is a caveat on how to interpret the results in figure 3*A* and *B*. We placed HGT events on the species trees, from which we are able to infer the time (i.e., branch) of these events. For example, we found 11 HGT genes most closely related to *T. pratense*, indicating that the upper (older) and lower (younger) bounds of the timing of HGT events are the *T. pratense*/*Med. truncatula* speciation time and the present, respectively. It does not necessarily mean that *T. pratense* is the donor because the donor might be another close relative of *T. pratense* that was not sequenced in this study. In figure 4*A*, two genes are placed on an internal branch leading to the *T. pratense*/*Med. truncatula* common ancestor, indicating that the upper bound should be the speciation time of the *T. pratense*/*Med. truncatula* common ancestor and other species including *Lo. japonicus*, while the lower bound is the present, not the *T. pratense*/*Med. truncatula* speciation time. With our data, it is difficult to determine the lower bound. Figure 4*C* illustrates such a situation. Suppose that a very recent HGT occurred from Host-0, but this Host-0 is missed in our phylogentic analysis. Then, in a resulting gene, the HGT gene in the parasite genome may be placed on the internal branch of the species tree of Hosts-1, 2, and 3, as shown in figure 4*D*, but it does not necessarily mean that the HGT event is older than the speciation event of Hosts-1 and 2.

### Genomic DNA Sequencing

In addition to mRNA sequences, we also sequenced genomic DNA of the five parasite species (supplementary table S2, Supplementary Material online). There are two major purposes for using genomic DNA. First, genomic DNA provides strong evidence that the detected HGT genes are integrated in the genome of the parasite species, thereby ruling out the possibility of false positive detection due to contamination. We here considered two kinds of contamination. First is the possibility of contamination of host DNA that came into the parasite cells. As RNA and DNA molecules could move between cells (Kim et al. 2014), our data might include such DNA molecules from the hosts. However, if those DNA fragments are not integrated into the parasite genome, they cannot replicate along cell divisions, so that we expect to observe them in a very low frequency in our short read data. In contrast, if our HGT genes are truly integrated into the genome (i.e., not contamination), we expect similar coverages for HGT and non-HGT genes. Supplementary figure S3, Supplementary Material online shows the distributions of coverage for HGT and non-HGT genes obtained from reads with identity 100%, >95%, and >90%. For *Oro. minor*, the three distributions are in agreement with a peak around ×10–12 in both HGT and nonHGT genes (supplementary fig. S4*A* and *C*, Supplementary Material online), ruling out the possibility of contamination. For *Ae. indica*, we found similar coverage for HGT and non-HGT genes with a peak around ×5–6 (supplementary fig. S4*B* and *D*, Supplementary Material online), indicating that our HGT genes are not due to contamination. It should be noted that the distributions of reads with identity >95% and >90% is quite different from that with 100% reads; The genome-wide average coverage is almost twice larger for the former (supplementary fig. S4*B*, Supplementary Material online). This should be because the host species experienced a whole genome duplication, WGD (with divergence ∼5–10%).

The second possibility of contamination is that host cells are included when we extracted DNA. This possibility should be quite low because the parasite plants are reasonably large (>10–30 cm in height), so that it is very unlikely that host’s tissue was included in our DNA extraction process, especially when we used leaves and stems that are not closely connected to the host (i.e., we did not use root tissues [haustorium]). If host’s cells were contaminated, we expect to obtain short-read data with extremely high homology to the host species at a very low coverage, which should spread over the genome. Obviously, this is not the pattern we observe because we found a limited number of local regions where a high coverage of reads exhibited high homology to the host.

The second purpose of genomic DNA sequencing is to investigate the exon–intron structure, from which we may be able to distinguish mRNA- and DNA-mediated transfers. With Illumina short reads of genomic DNA, we constructed genomic DNA regions for introns. We developed a simple algorithm for gradually assembling introns from the already-assembled exons, as illustrated in supplementary figure S5, Supplementary Material online. We successfully obtained assemblies of 77 HGT genes (19 for *Oro. minor* and 58 for *Ae. indica*) out of the 106 genes. For the remaining 29 HGT genes (3 for *Oro. minor* and 26 for *Ae. indica*), although we were able to confirm their presence in the genomes, we could not assemble them because they are duplicated. This particularly applies more to *Ae. indica*, which underwent a recent WGD. If an HGT occurred before the WGD, we would detect two copies of the descendants of the HGT genes, which are difficult to distinguish because of a low divergence (this problem does not apply to HGTs occurred after the WGD). Other cases would be due to, for example, segmental duplication followed by HGT, simultaneous transfer of tandemly duplicated genes, and independent transfers of very similar genes.

**Fig. 5.**
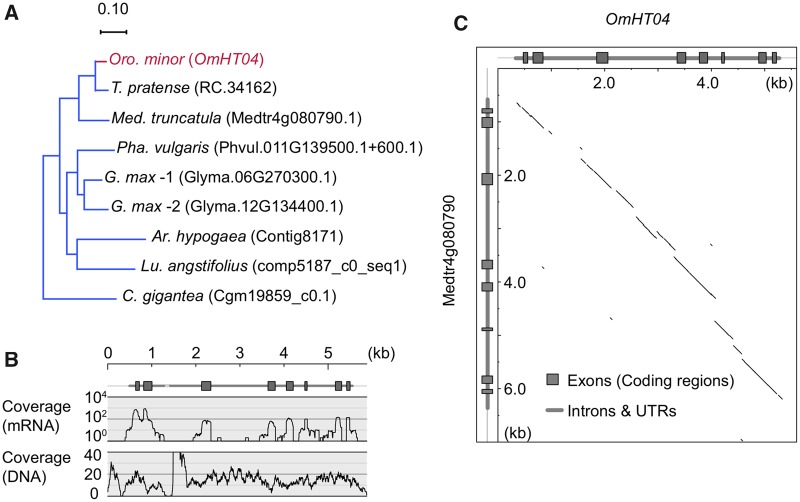
—Exon–intron structure revealed by genomic DNA sequences. *OmHT04* is used as an example. (*A*) Gene tree. (*B*) The coverage of short-reads of Illumina HiSeq2000 sequencing along *OmHT04*. (*C*) Dotplot of comparison between *OmHT04* and its homolog from *Medicago truncatula*.

Figure 5 summarizes the analysis of genomic DNA with an example of one HGT gene in *Oro. minor*. This gene (labeled as *OmHT04*) has the closest homolog in *T. pratense* (ID = RC.34162; fig. 5*A*). Using our genomic DNA sequences, we were able to reconstruct the genomic sequence of *OmHT04*. *OmHT04* has seven introns that are spliced in our assembled cDNA sequence, and this exon–intron structure and sequences are very similar to the homolog (ID = Medtr4g080790) in *Med. truncatula* (unfortunately, no genomic DNA sequence is available for *T. pratense*). Figure 5*B* shows the distribution of the coverage of short reads of both mRNA and genomic DNA over the genomic region of *OmHT04*, where genomic short-reads spread over the entire region, while mRNA reads have clear peaks on exons. It is indicated that *OmHT04* originates a DNA-mediated HGT event and that the introns are successfully spliced in the cell environment of *Oro. minor*. Figure 5*C* shows a dotplot of the genomic region of *OmHT04* and the reference genome sequence of *Med. truncatula*. We found that there are no nonsense mutations or indels in exons, suggesting that *OmHT04* is an expressed gene with potentially functional mRNA. It seems that the exon–intron structure and sequence of *OmHT04* have been well conserved since the HGT event, suggesting that *OmHT04* was introduced in the *Oro. minor* genome through a DNA-mediated transfer event.

The genomic regions of all HGT genes were reconstructed with our genomic DNA, and compared with the genomic reference sequences of the potential host species. We first aimed to examine whether the HGT genes originate from DNA- or mRNA-mediated transfer by looking at the presence/absence of introns. We applied this analysis to 90 genes (21 *Oro. minor* and 69 *Ae. indica* genes, respectively), for which there is at least one introns in the corresponding region in the host reference genome. It is found that the exon–intron structures are fairly well conserved for all genes. This result indicates that all detected transferred genes have introns, indicating a crucial role of DNA-mediated transfer, rather than mRNA-mediated retro transfers.

We then compared the distributions of the coverage of short reads of mRNA and genomic DNA for the 67 genes (see fig. 5*B* for example, supplementary fig. S6, Supplementary Material online for full results). We confirmed that all introns are spliced in most genes (17 *Oro. minor* and 41 *Ae. indica* genes, respectively). Note that we considered that an intron is spliced when we observed at least one successfully spliced mRNA read at the exon–intron junction.

We also checked if these well spliced genes accumulate any nonsense mutations and/or frameshift-causing indels. It was found that 13 *Oro. minor* and 14 *Ae. indica* genes had no such mutations for the entire coding region. We also applied this to one *Oro. minor* and nine *Ae. indica* genes with no introns that were excluded in the above analysis on intron-splicing. We found that two *Ae. indica* genes did not have any nonsense mutations or frameshift. Thus, in total 29 genes, the original function may be preserved at least at the mRNA level (supplementary table S6, Supplementary Material online).

### Chromosome Locations

**Fig. 6.**
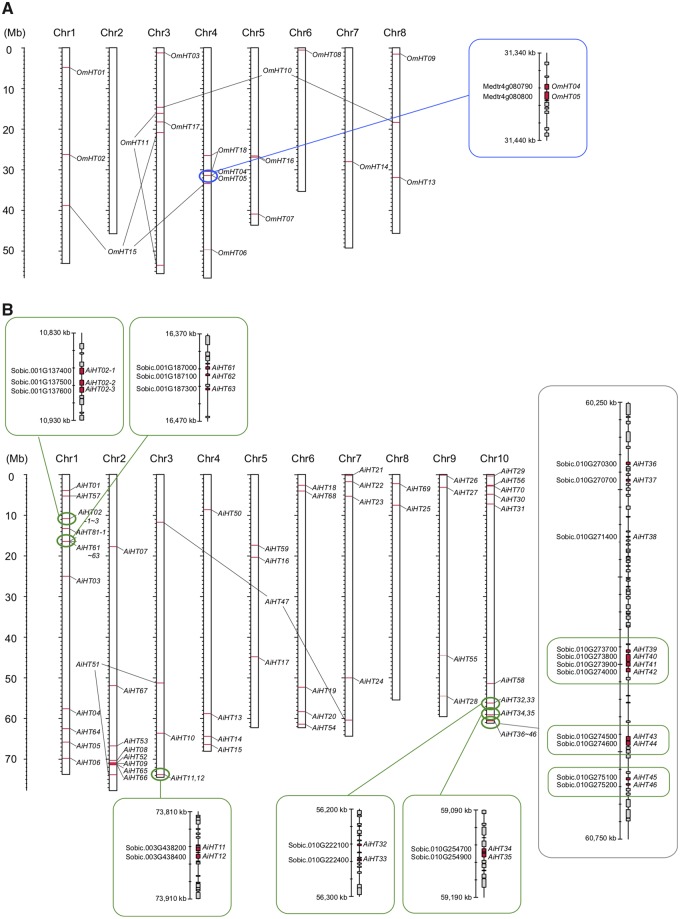
—(*A*) Chromosomal locations of the homologs of *Orobanche minor* HGT genes on the genome of *Medicago truncatula*. (*B*) Those for *Aeginetia indica* HGT genes on the genome of *Sorghum bicolor*. HGT genes that presumably transferred simultaneously are shown in boxes.

As we showed that most HGT genes originate from DNA-mediated events, we suspected that multiple closely-linked genes on a donor’s chromosome might have been transferred simultaneously. For exploring this possibility, we investigated the chromosomal locations of the 106 HGT genes in their closest potential host genomes. Note that we were not able to identify the chromosomal locations of these genes in the parasites themselves because of the lack of their whole-genome sequences or linkage maps. Figure 6*A* shows an example with 17 *Oro. minor* genes that should have originated from the ancestral lineage leading to *T. pratense* and *Med. truncatula* (the blue part in fig. 4*A*). These genes were mapped on the chromosomes of *Med. truncatula*, indicating that the 16 HGT genes distribute randomly on the host genome, except that *OmHT04* and *OmHT05* are very close to each other. A close look at this region revealed that the two genes are next to each other, suggesting a strong possibility that they were transferred by a single event. This possibility may be verified if the two genes also locate next to each other in the *Oro. minor* genome, but we were not able to prove this because of the lack of *Oro. minor* reference genome. Figure 6*B* shows an example with 70 *Ae. indica* genes that should have originated from the ancestral lineage leading to *So. bicolor* and relatives (the green part in fig. 4*B*), which are mapped on the chromosomes of *So. bicolor*. We identified 8 gene clusters, 3 of which are located on the tip of chromosome 10 of *So. bicolor* and they might be transferred by a single event although we treated them as three distinct clusters. Thus, mapping the HGT genes on the reference genomes of the parasite species is informative to know the rough lengths of the HGT events (supplementary table S6, Supplementary Material online). If all HGT genes were transferred independently, the typical length may distribute up to several ten kb, while our results suggest that at least one order of magnitude longer genomic regions might have transferred; the length exceeds ∼400 kb if the three clusters on the tip of chromosome 10 have been transferred as a single event.

### Evolutionary Analyses

In order to investigate how the evolutionary rate has changed after transfer, we focused on ω (ratio of nonsynonymous to synonymous substitution rates). We applied the branch model in the codeml program (PAML 4 package, Yang 2007). To be conservative, we assume a simple trifurcation tree of the three gene trios, the HGT gene in the parasite species, its homolog in the closest donor and an outgroup (the gene-trio for each locus used for the analysis are listed in supplementary table S8, Supplementary Material online). We set the null model such that the three branches have a single rate. Provided that our alternative hypothesis is that the evolutionary rate can be changed after horizontal transfer, the alternative model assumes that the branch to the HGT gene has a different rate from the other two lineages. Applying the likelihood test in PAML 4–22 *Oro. minor* and 83 *Ae. indica* genes, we found that the alternative model fit the observed data significantly better than the null model for 8 *Oro. minor* and 19 *Ae. indica* genes at 5% level. It was not possible to distinguish whether this elevation of ω after transfer is due to relaxation of negative selection or positive adaptive selection.

### GO Enrichment Analysis

In order to identify enriched GO terms in our HGT genes, we performed the SEA (Singular Enrichment Analysis) on agriGO v1.2 (http://bioinfo.cau.edu.cn/agriGO; Du et al. 2010). We first performed the SEA for the homologs of the 22 *Oro. minor* HGT genes against the four host species in Fabaceae (supplementary table S9, Supplementary Material online). We obtained enriched GO terms with *P* values < 0.01 as summarized in supplementary table S10, Supplementary Material online. It was found that ncRNA metabolic process (GO: 0034660) and its hierarchically related terms such as rRNA and tRNA metabolic processing (GO: 0016072, GO: 0006399) were significantly enriched in the OmHGT genes. The same analysis was applied to the 84 *Ae. indica* HGT genes with the five host species in Poaceae (supplementary tables S9 and S10, Supplementary Material online). Thiamin metabolic process (GO: 0006772) and its hierarchically related terms, such as thiamin biosynthetic process (GO: 0009288), were significantly enriched in the *Ae. indica* HGT genes. There was no GO term that exhibited significant enrichment both *Oro. minor* and *Ae. indica*. We could not find significance for defense related genes, although Yang et al. (2016) suggested their importance. It is suggested that the repertory of HGT genes largely depends on species.

## Discussion

In order to understand the evolutionary role of HGT in plants, this research focused on parasite plants in the family Orobanchaceae and their potential host species in Fabaceae and Poaceae. We have sequenced five parasite species, *Oro. minor*, *Ae. indica*, *Pe. keiskei*, *Pht. japonicum*, and *Mel. roseum*, in which we detected >100 HGT genes. We found 22 and 84 HGT genes in the two obligate parasites, *Oro. minor* and *Ae. indica* (supplementary table S4, Supplementary Material online), which account for at least around 0.1% in the *Oro. minor* genomes and 0.2% in *Ae. indica*. These proportions are consistent with other Orobanchaceae parasite species (Yang et al. 2016), while a slightly higher value (∼2%) was reported for Rafflesia (Xi et al. 2012).

We consider that very few false positives are included in our list of HGT genes for two reasons. The evolutionary trees of the HGT genes with a number of homologs across angiosperm seem to be most convincing evidence (fig. 3 and supplementary fig. S3, Supplementary Material online). We successfully identified homologs from a wide range of angiosperm species in most cases (91 out of the 106 HGT genes). For each of our HGT genes, we constructed an ML tree, which includes the HGT gene itself, its homolog detected in the parasite genome (the direct paralog within the same genome) and homologs in other species. In most ML trees, if we exclude the HGT gene, the tree shape was roughly consistent with the species tree, and the HGT gene is located within the cluster of its potential host family (Fabaceae for *Oro. minor* and Poaceae for *Ae. indica*), rather than within the Lamiales cluster. Therefore, we can conclude our 106 HGT genes are most likely transferred from the host species via HGTs.

We further carefully examine our result to exclude the possibilities of false detection of HGT genes. There are at least two other possibilities for a discordance between gene tree and species tree, incomplete lineage sorting and hidden paralogy (Than et al. 2007; Galtier and Daubin 2008; Degnan and Rosenberg 2009). We first argue against the possibility that incomplete lineage sorting caused false detection of HGT genes. Incomplete lineage sorting could confuse the shape of gene tree between relatively close species, not between different families. Therefore, incomplete lineage sorting cannot explain our observation of the ML trees in figure 3 and supplementary figure S3, Supplementary Material online, where our HGT genes located within the potential host family (Fabaceae for *Oro. minor* and Poaceae for *Ae. indica*). Along a similar argument, hidden paralogy less likely explains our results. Hidden paralogy arises as a big problem when frequent turnovers of genes occur through gene duplication and loss. If this applies, we would expect to observe a complex gene tree with many lineages shuffling between families, exhibiting a high inconsistency with the species tree. However, our results showed that only HGT genes are inconsistent with the species tree.

The above argument does not hold with the 15 cases (all are HGT genes in *Ae. indica*), where homologs were detected only in the host family (i.e, Poaceae). Even for these cases, we consider that they are not likely false positives. This is because our study focuses on relatively recent HGT events (up to 53–67 Ma), so that the origins of HGTs were well inferred on gene trees of host family. In some cases, although the trees are unrooted, the HGT genes seem to be placed within the tree of the host family, thereby providing evidence for horizontal transfer. In addition, the observed high similarity (Ks < 1) between the HGT gene and homologs in the host should be another line of evidence for horizontal transfer.

We also consider the possibility of DNA contamination causing false detection of HGT genes. By sequencing genomic DNA at high coverage, we found that all HGT genes detected were well confirmed by high coverage genomic DNA, indicating that they are integrated in the genomes of the parasite species. However, we were able to reconstruct relatively short genomic regions encompassing exons, and additional data would be useful to understand the whole picture of HGTs. For this purpose, it is desired to look at synteny along a chromosome (e.g., Yoshida et al. 2010). Unfortunately, our short-read data (paired-end with 500 bp insert) is not sufficient to reconstruct large contigs encompassing multiple neighboring genes. The DNA sequence of the host individual is also informative for checking contamination of DNA/mRNA because such contamination of host DNA can be immediately identified (e.g., Xi et al. 2012), but it was very difficult to identify the host individuals of our sample in the wild).

All 106 HGT genes were found in two obligate parasites (*Oro. minor* and *Ae. indica*) likely transferred from their hosts, indicating that their tight relationship with hosts enhances HGTs. The result is consistent with that of Yang et al. (2016), who investigated another three species in Orobanchaceae. They also reported that most HGT genes were found in the obligate parasite species. This biologically makes sense because the two obligate parasites attach to roots of their host soon after germination and form a “terminal haustorium” and then vegetative growth follows, while the other three facultative parasites can vegetatively grow independently after germination (Westwood et al. 2010). It should be noted that because a terminal haustorium must connect to host vascular tissue before further plant development can proceed (see fig. 3 in Westwood et al. 2010), it is likely that any mutations including HGTs that occurred in the terminal haustorium will contribute to the germline (e.g., Birch 1997). On the other hand, a facultative parasite does not form a terminal haustorium, and parasites a host by simply forming “lateral haustoria” between its roots and the host’s roots. It is very unlikely that HGTs occurred in a lateral haustoria contributes to the germline because they simply stay as a part of the root.

It is predicted that the lifestyle has drastically changed after parasitism, which should also have changed the genomic environment. In such a situation, it is possible that genes transferred from different species happen to acquire an important function. This might at least partly explain our observation that a number of HGT genes were detected in the obligate parasites. By contrast, we did not find any HGTs in the three facultative parasites nor in the other direction (from parasite to host).

Because we analyzed a number of potential host species (eight Fabaceae and six Poaceae species; fig. 1), we were able to roughly infer the origin (donor) of HGT genes (fig. 3), as was also done by Yang et al. (2016). Although the two parasite species, *Oro. minor* and *Ae. indica*, have relatively wide ranges of hosts, it is known that *T. pratense* is one of the favorite hosts of *Oro. minor* and that *Ae. indica* preferably parasitizes *Mis. sinensis* which is closely related to *So. bicolor* and *Z. mays*. It is found that the majority of HGT genes is likely from the preferred hosts (fig. 2), indicating that our result is quite consistent with the parasites’ lifestyles and that the physiological closeness of host–parasite relationship may be an important factor to determine the rate of HGTs.

We found that all detected HGT genes in the parasite species shared introns at the same locations with their homologs in the host species. Hence DNA-mediated transfer explains the majority of our data, in strong agreement with that of Yang et al. (2016). It may not be inconsistent with Xi et al. (2012)’s genome-wide survey of *Rafflesia*, where the authors reported similar cases for several genes although the coverage of their genomic DNA data was not high. It may be possible that genome sequencing of *Rafflesia* with higher coverage could increase the number of genes with conserved introns. Our data did not show strong evidence for mRNA-mediated HGTs.

We confirmed all detected HGT genes expressed mRNA. By comparing mRNA and genomic DNA, we found that almost all splice sites of introns were conserved between the parasite species and hosts, indicating these introns have been well spliced since they were transferred into the parasite genomes. It might suggest that the splicing machinery may be fairly conserved at least between monocots and dicots, which is consistent with the empirical demonstrations with transgenic tobacco and rice (Keith and Chua 1986; Tanaka et al. 1990).

We found that about half of the 106 HGT genes did not have any missense mutation or frameshift-causing indels. Are they really functional in the current genome? It is not very easy to answer this question from our data alone because we were not able to determine the lower (younger) bounds of the timing of HGT events. Our results suggest two possibilities. 1) First, most HGTs are very recent. If so, it is very difficult to claim that these HGTs are playing important roles in the parasite genome and are maintained by selection. It is also difficult to know whether they are fixed in the host species or are rare variants. 2) The second possibility is that most HGTs are quite old. If so, they should have been fixed and preserved for a long time probably because they conferred some advantage. Some of the HGT genes detected are to some extent old such that they have accumulated some missense mutations and frameshift-causing indels, likely on the way to be pseudogenes. This suggests that these HGT genes fixed in the population perhaps because they were not very deleterious (or even adaptive), otherwise were immediately lost from the population. Provided that many of the HGT genes have a homolog in the parasite genome, the HGT genes could be beneficial through the scenarios of neofunctionalization or subfunctionalization (e.g., Innan and Kondrashov 2010). More data are needed to distinguish these two possibilities, including polymorphism data and more species in the host families, which will also be informative to understand the role of natural selection on HGT genes.

This work is overall similar to Yang et al. (2016), who investigated three parasite species (different from ours) in the same family, Orobanchaceae. A major difference is that Yang et al. (2016) explored a wide range of potential host families within angiosperms, but used only one or two species from each family, whereas we focused on two major host families, Fabaceae and Poaceae, from which multiple species were used. This difference made us possible to uniquely inferred the timing HGT events on a branch within the species tree. We used this strategy because the time to common ancestor of Fabaceae, that of Poaceae, and the origin of parasitism are quite similar (roughly 50 Ma), so that we predicted that HGT events after the acquisition of parasitism should fall within the species tree of the two families, as we demonstrated here. A disadvantage is that our strategy will miss HGT genes from out of the two families, while Yang et al. (2016) do not.

Furthermore, we detected 84 HGTs in *Ae. indica*, much more than other species in Orobanchaceae (22 in *Oro. minor*, 1–34 in the four species used by Yang et al. 2016). This motivated us to investigate their genomic locations, from which we found many HGT genes are located closely one another, indicating that they were transferred simultaneously. What would be the reason why *Ae. indica* uniquely has many HGT genes? A possible answer would be because this species underwent a WGD, after which at most a half of the genomic region could be potentially redundant. Therefore, an HGT inserted even into a functional region may not be subject to purifying selection as long as long as the paralogous region maintains the function.

Together with the recent work by Yang et al. (2016), we here propose that Orobanchaceae provides an excellent model for studying HGTs because it covers a number of parasite species (89 genera with roughly 2,000 species, Bennet and Mathews 2006) with the full range of parasitic capabilities and there is great variability in their host species, from monocots to dicots. It is also believed that evolutionary changes in the parasitic capability, that is from facultative to obligate parasites, have occurred several times independently (McNeal et al. 2013). This situation allows us to explore how HGTs were involved in the evolution of the species in this family. In addition, some Orobanchaceous species are relatively easy to cultivate in limited space, making them easier to apply some basic techniques of molecular biology and genetics.

## Supplementary Material

Supplementary data are available at *Genome Biology and Evolution* online.

## Supplementary Material

Supplementary Data
