# Supplementary material for: Horizontal Gene Transfer in Five Parasite Plant Species in Orobanchaceae
Source: Genome Biol Evol. 2018 Nov 8;10(12):3196–210. doi: 10.1093/gbe/evy219 (PMC6294234; doi:10.1093/gbe/evy219)
Supplement: Supplementary Data [file evy219_supp.zip › Suppl_Figures_H75.pdf]

Figure S1 (1/2)

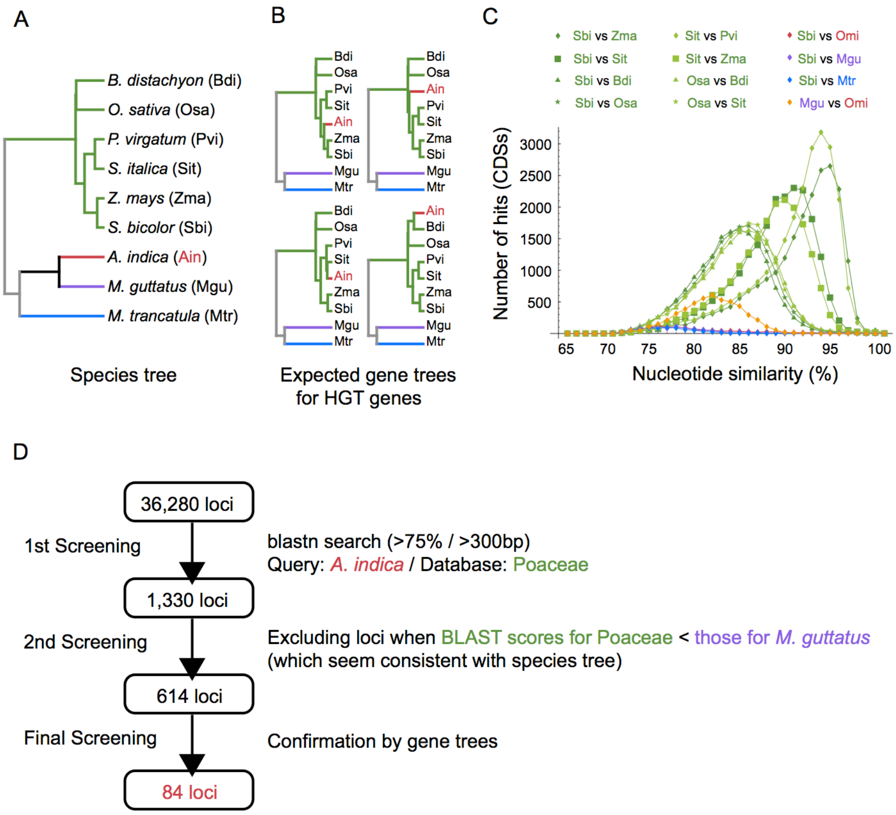

Figure S1 --- Summary of the screening process for *A. indica*. (A) Species tree of *A. indica* and six species in Poaceae (green) together with two species used as outgroups (*M. guttatus* and *M. truncatula*). (B) Typical gene trees expected for HGT genes. (C) Distributions of nucleotide identity between species. (D) Screening scheme. (E) Gene trees for the 84 HGT genes, labeled from AiHT01 to AiHT81. The branch lengths in the trees are based on pairwise distance at synonymous site with the Jukes-Cantor correction. Bootstrap values (>75%) are shown on branches. Red and blue branches are for the parasite (*A. indica*) and its homologs in Poaceae. When paralogs were detected in Poaceae, their lineages are shown in gray.

Figure S1 (2/2)

E

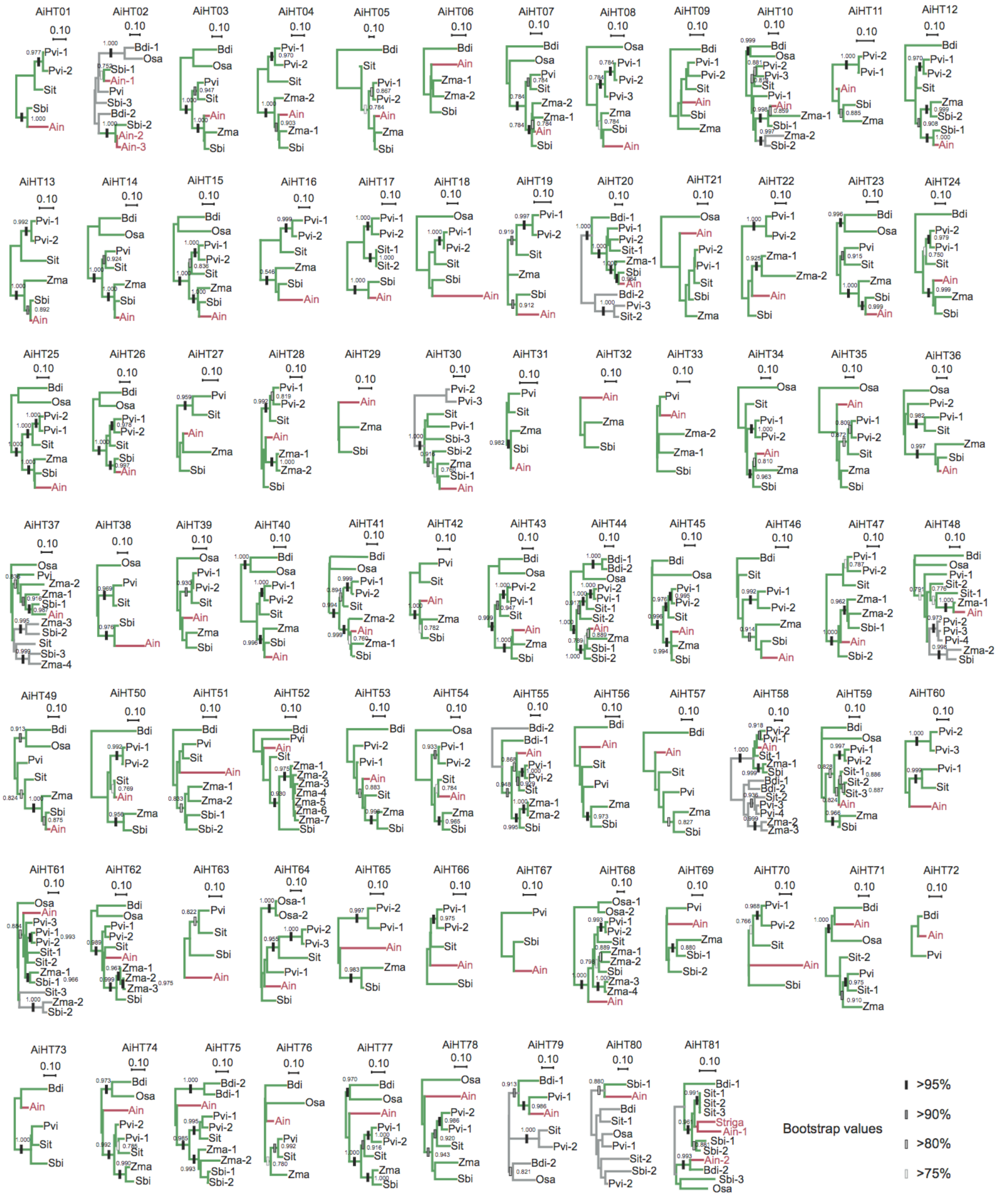

Figure S2

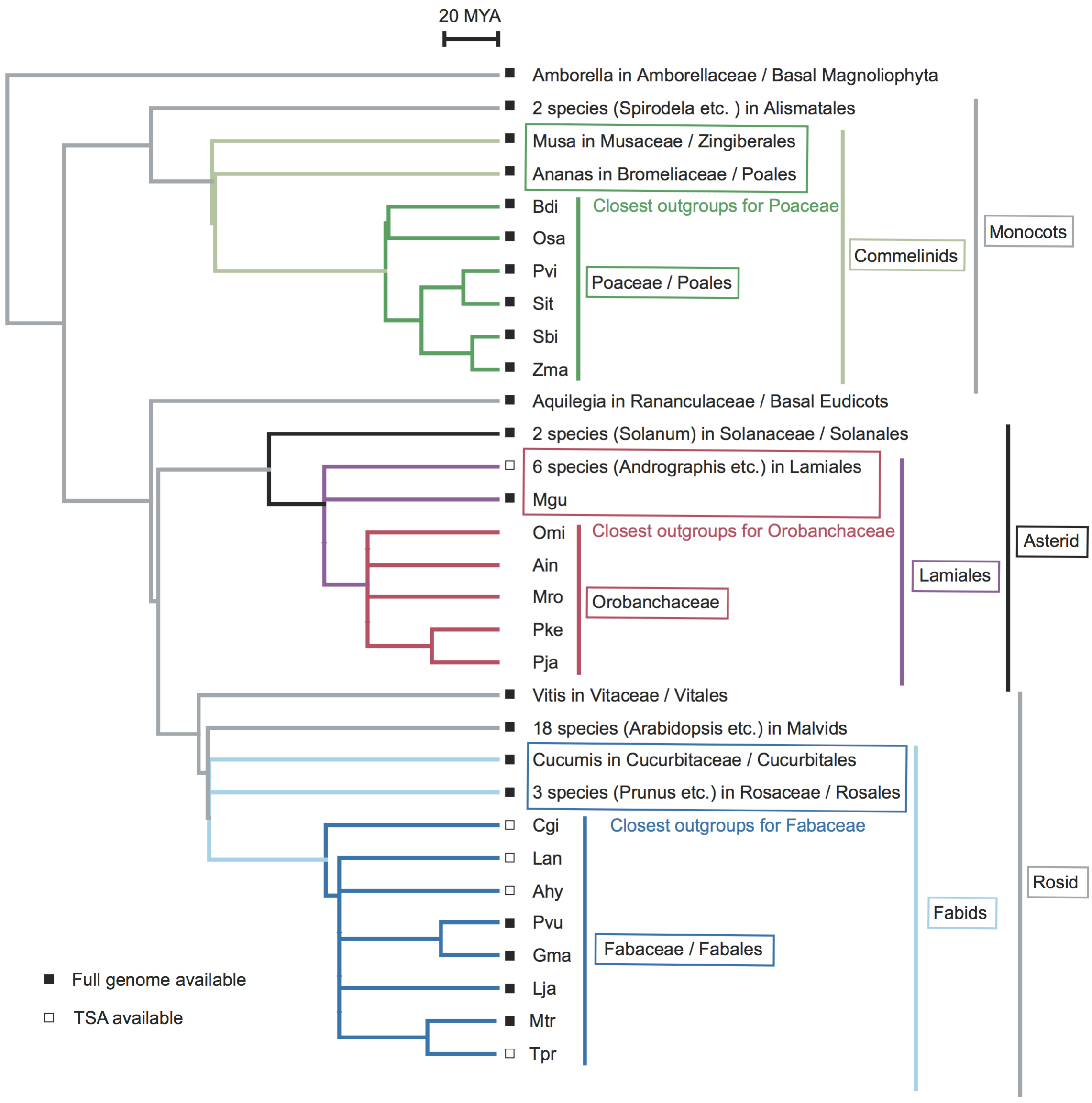

Figure S2 --- Phylogenetic relationship among the five species in Orobanchaceae and 52 angiosperm species used for BLASTN search in Table S7. The speciation times are according to TimeTree (Hedges and Kumar 2009; Hedges et al. 2015; http://www.timetree.org).

Figure S3 (1/27)

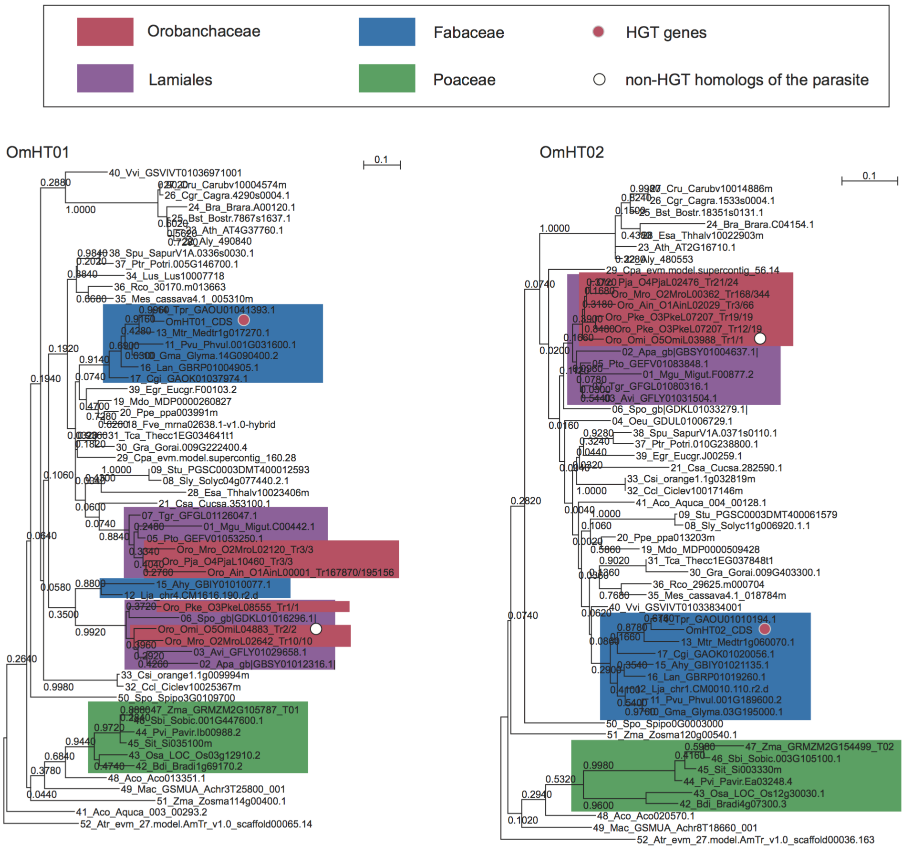

Figure S3 --- Gene trees of the 106 HGT genes with their homologs in 52 angiosperm species. See the text for details.

Figure S3 (2/27)

OmHT03

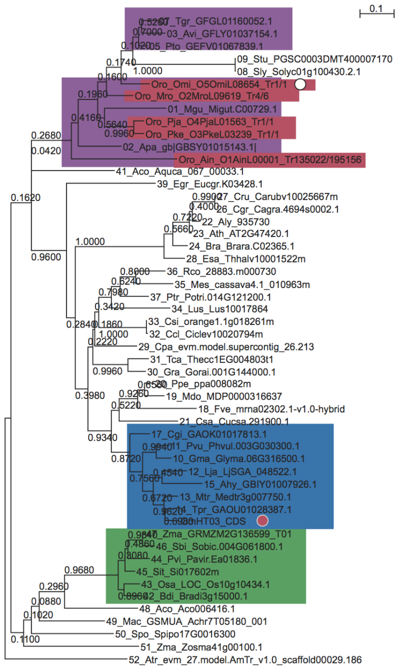

OmHT04

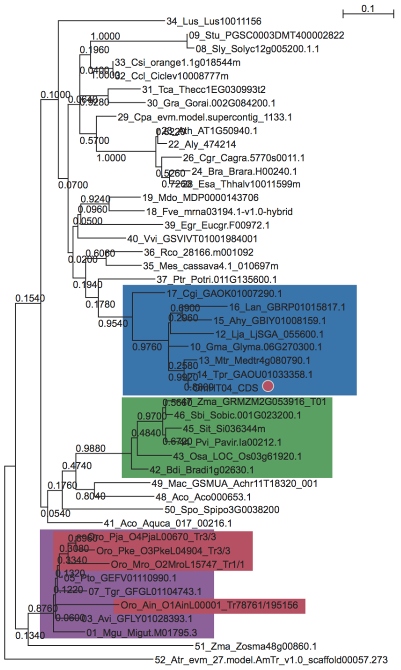

OmHT05

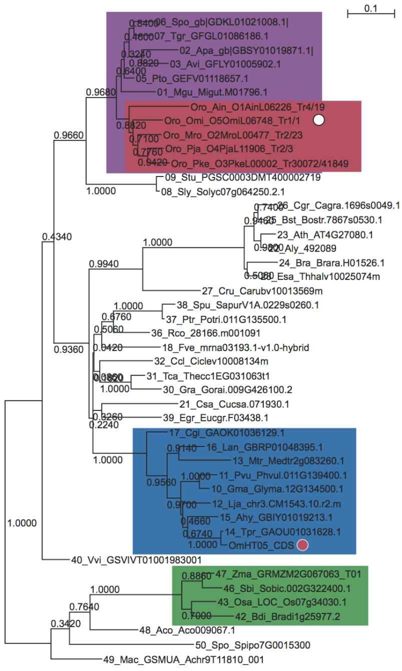

OmHT06

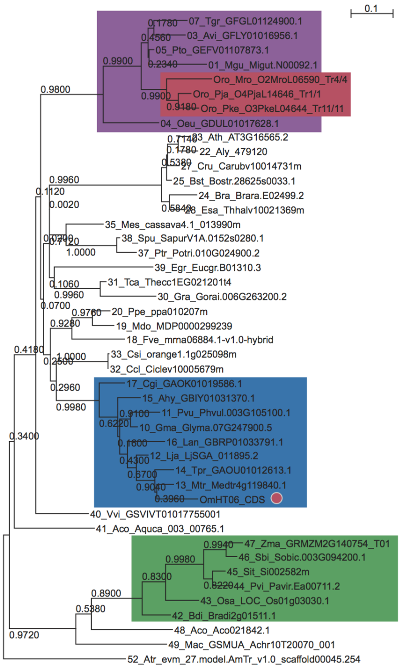

Figure S3 (3/27)

OmHT07

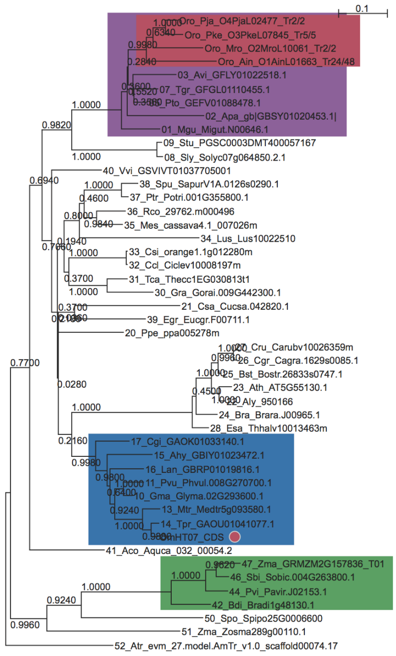

OmHT08

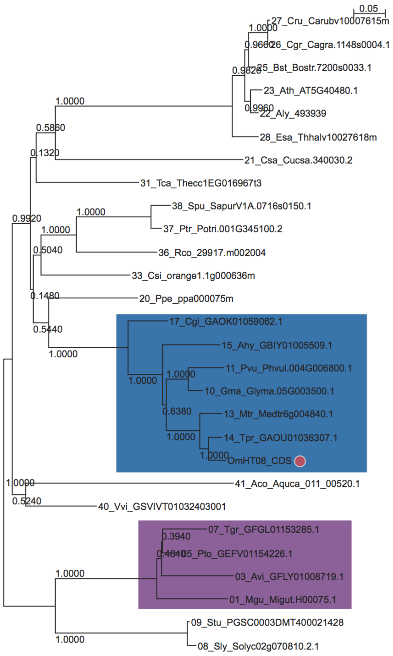

OmHT09

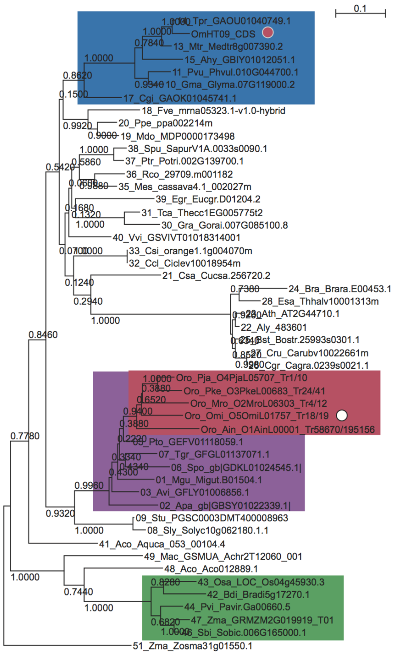

OmHT10

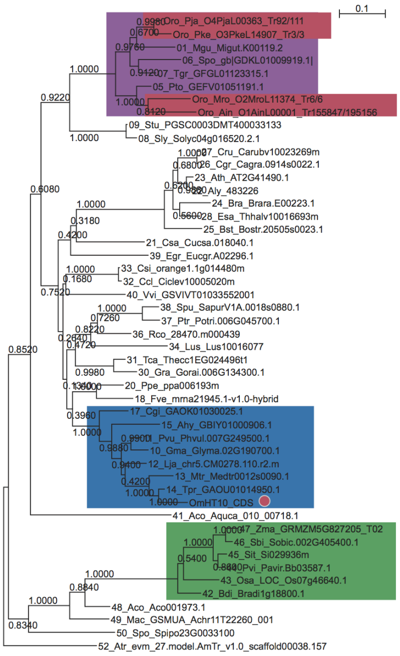

Figure S3 (4/27)

OmHT11

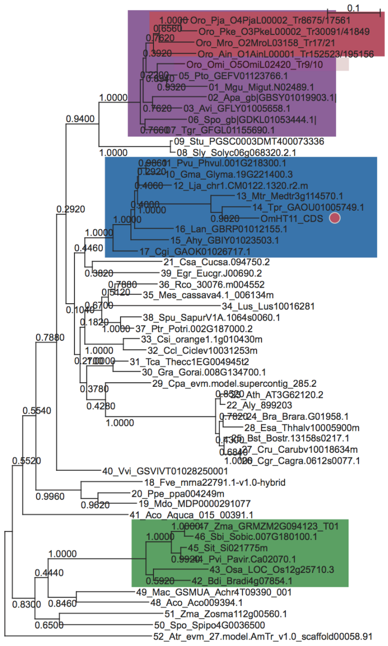

OmHT12

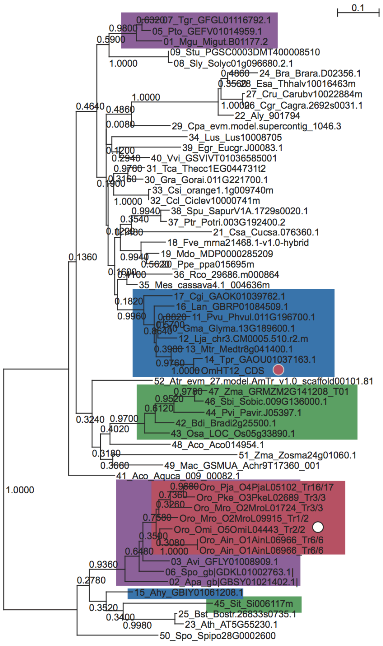

OmHT13

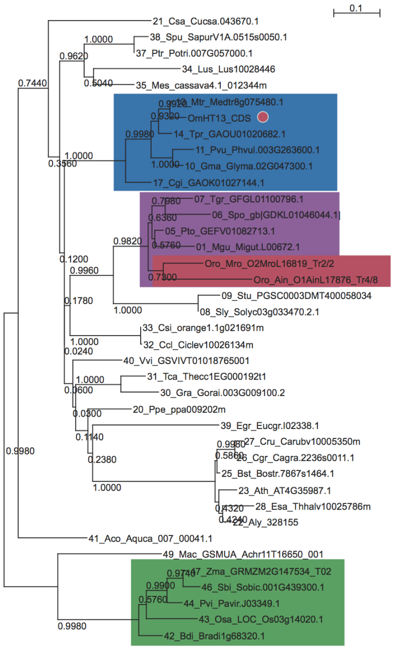

OmHT14

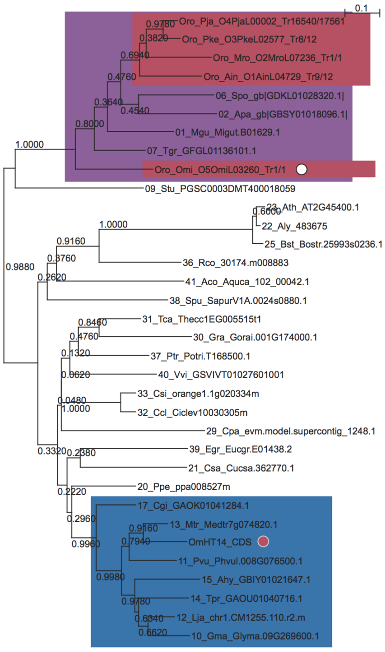

Figure S3 (5/27)

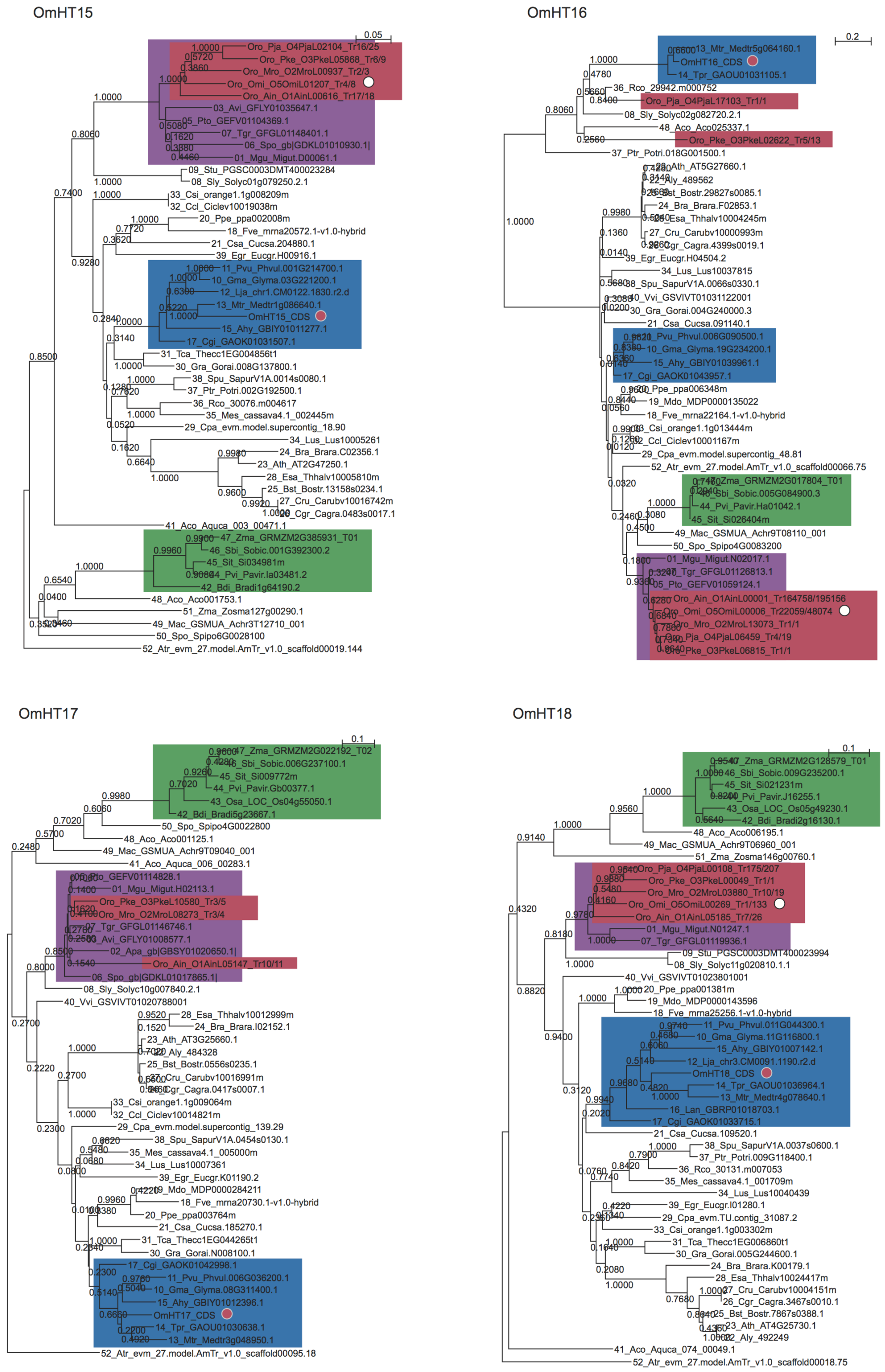

Figure S3 (6/27)

OmHT19

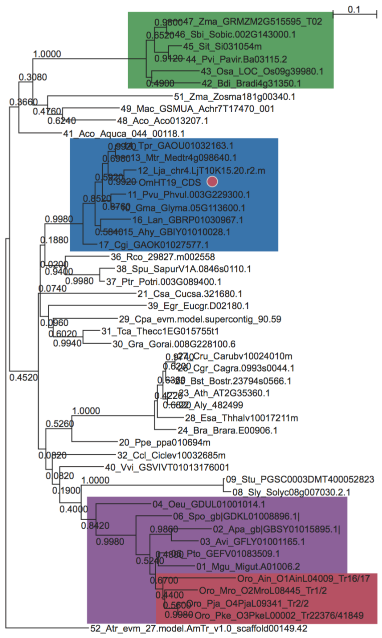

OmHT20

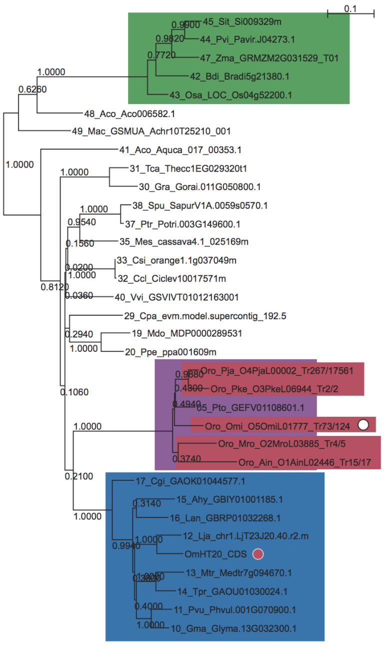

OmHT21

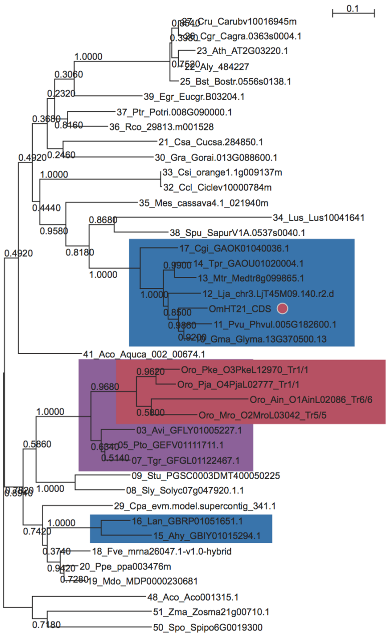

OmHT22

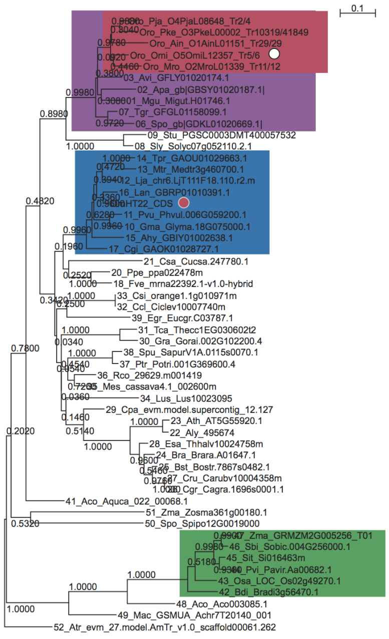

Figure S3 (7/27)

AiHT01

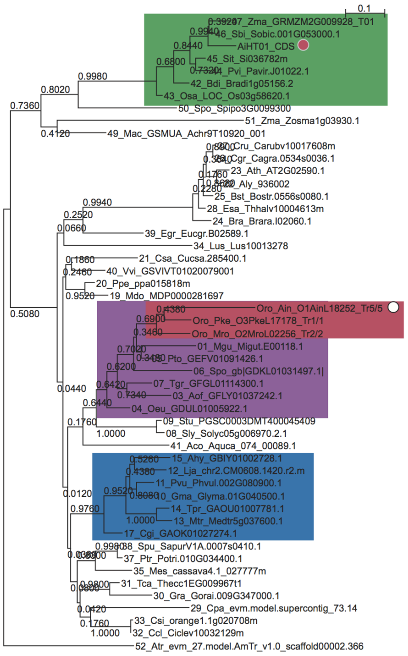

AiHT02

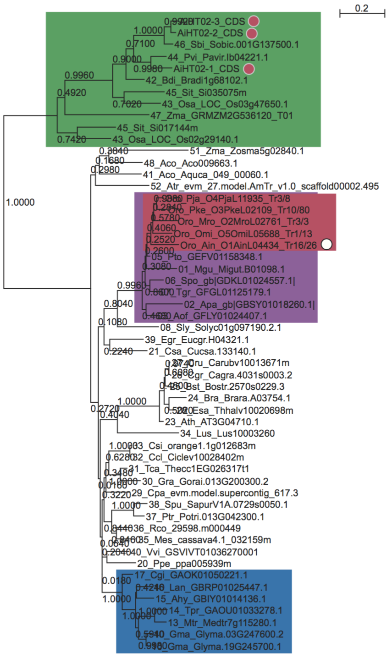

AiHT03

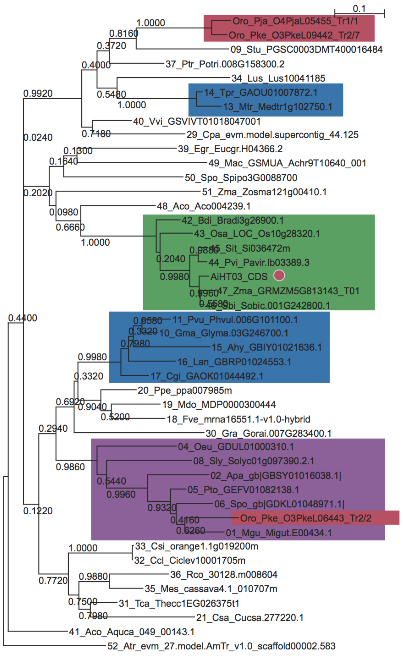

AiHT04

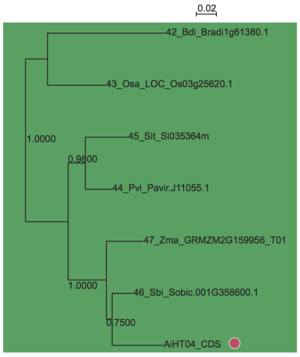

Figure S3 (8/27)

AiHT05

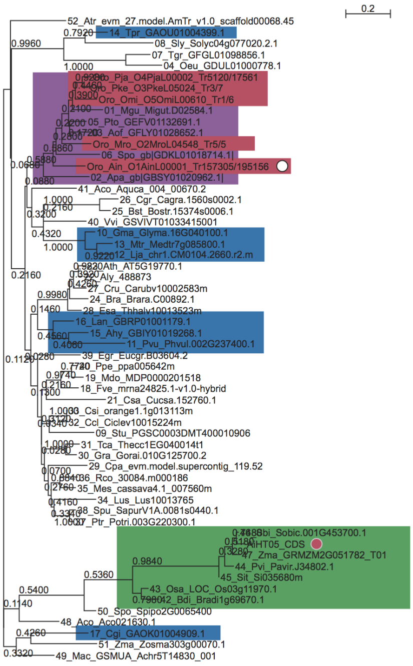

AiHT06

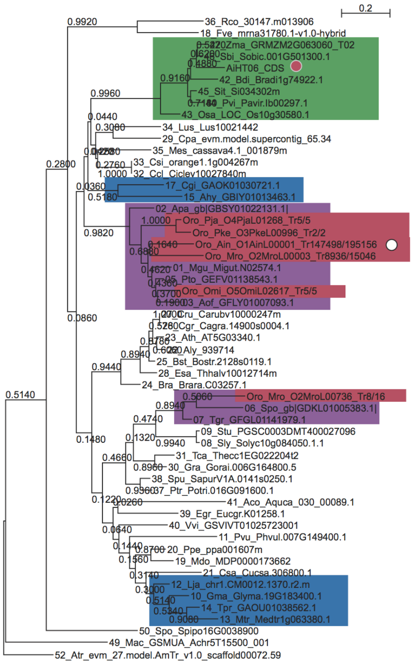

AiHT07

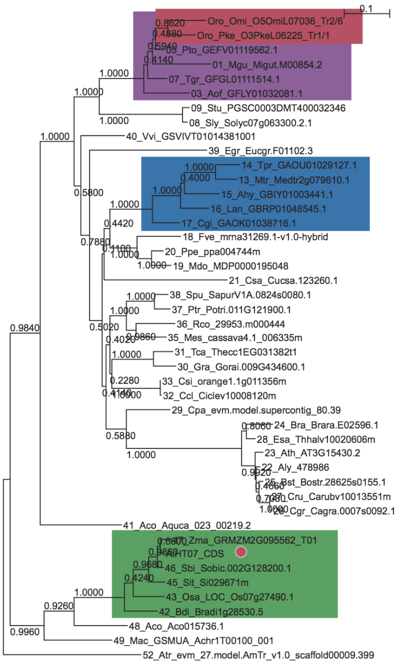

AiHT08

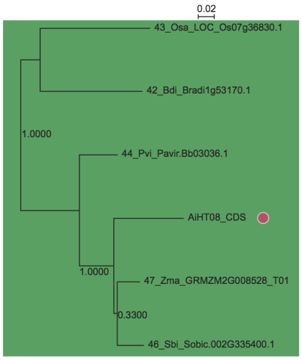

Figure S3 (9/27)

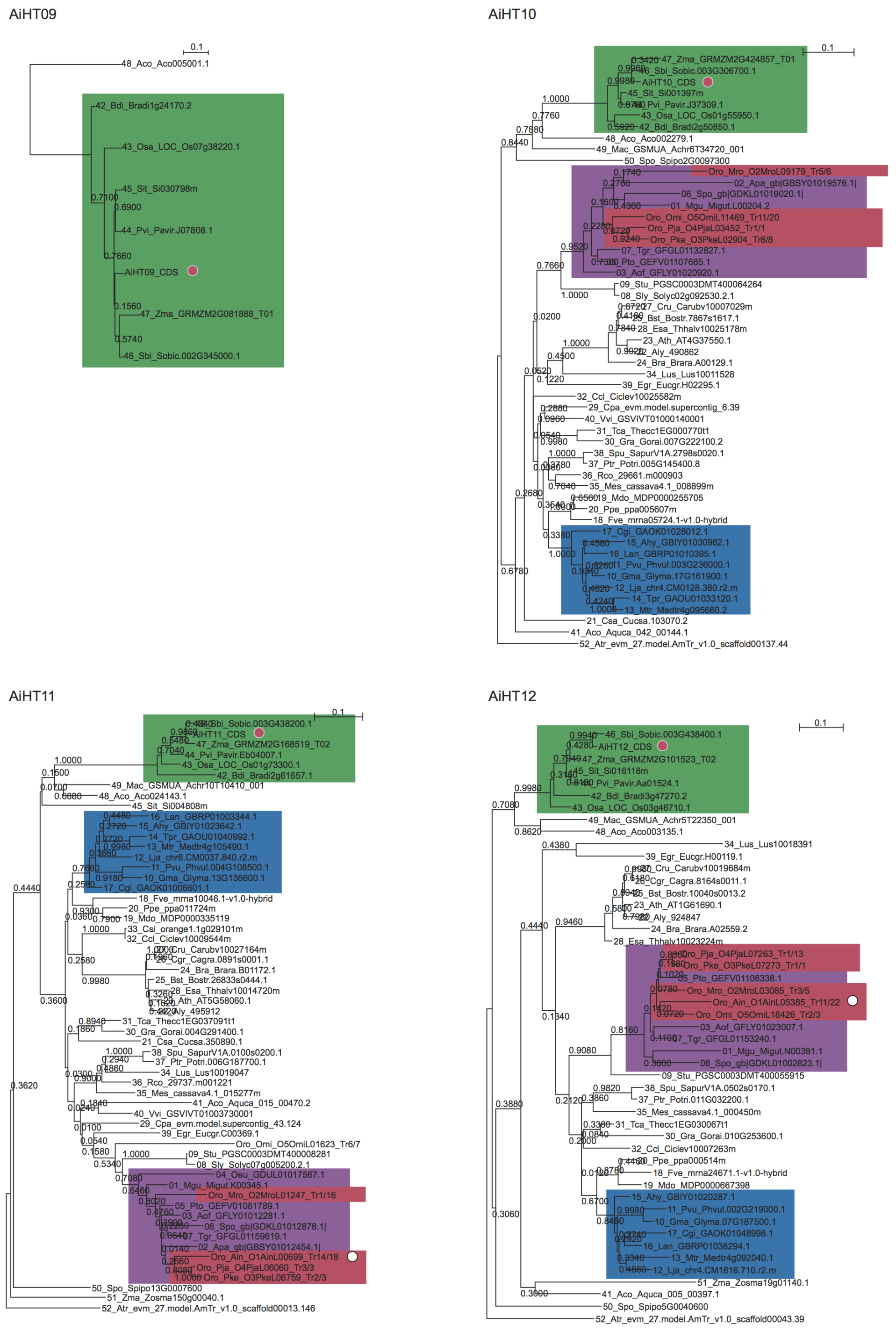

Figure S3 (10/27)

AiHT13

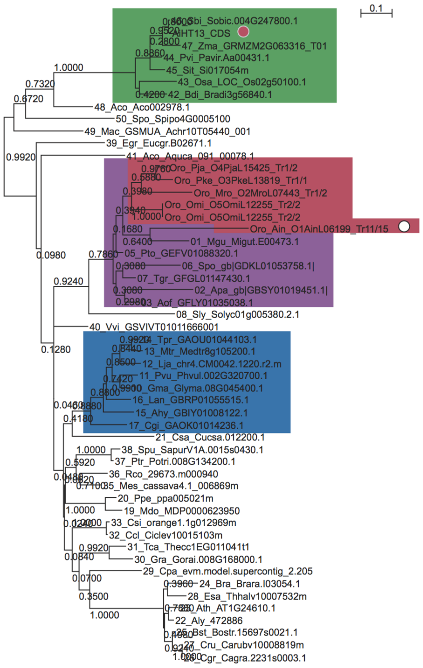

AiHT14

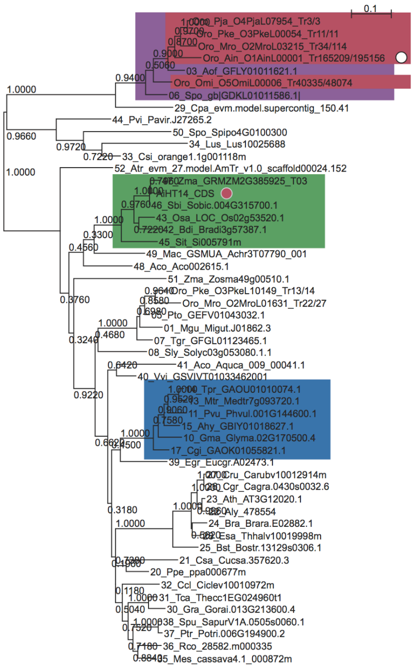

AiHT15

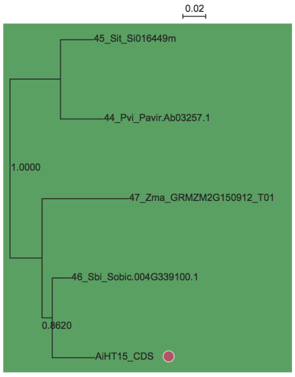

AiHT16

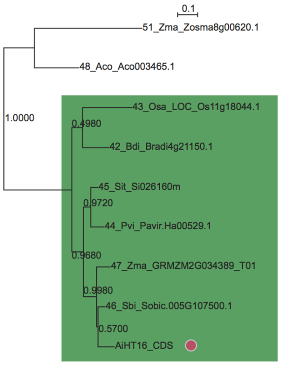

Figure S3 (11/27)

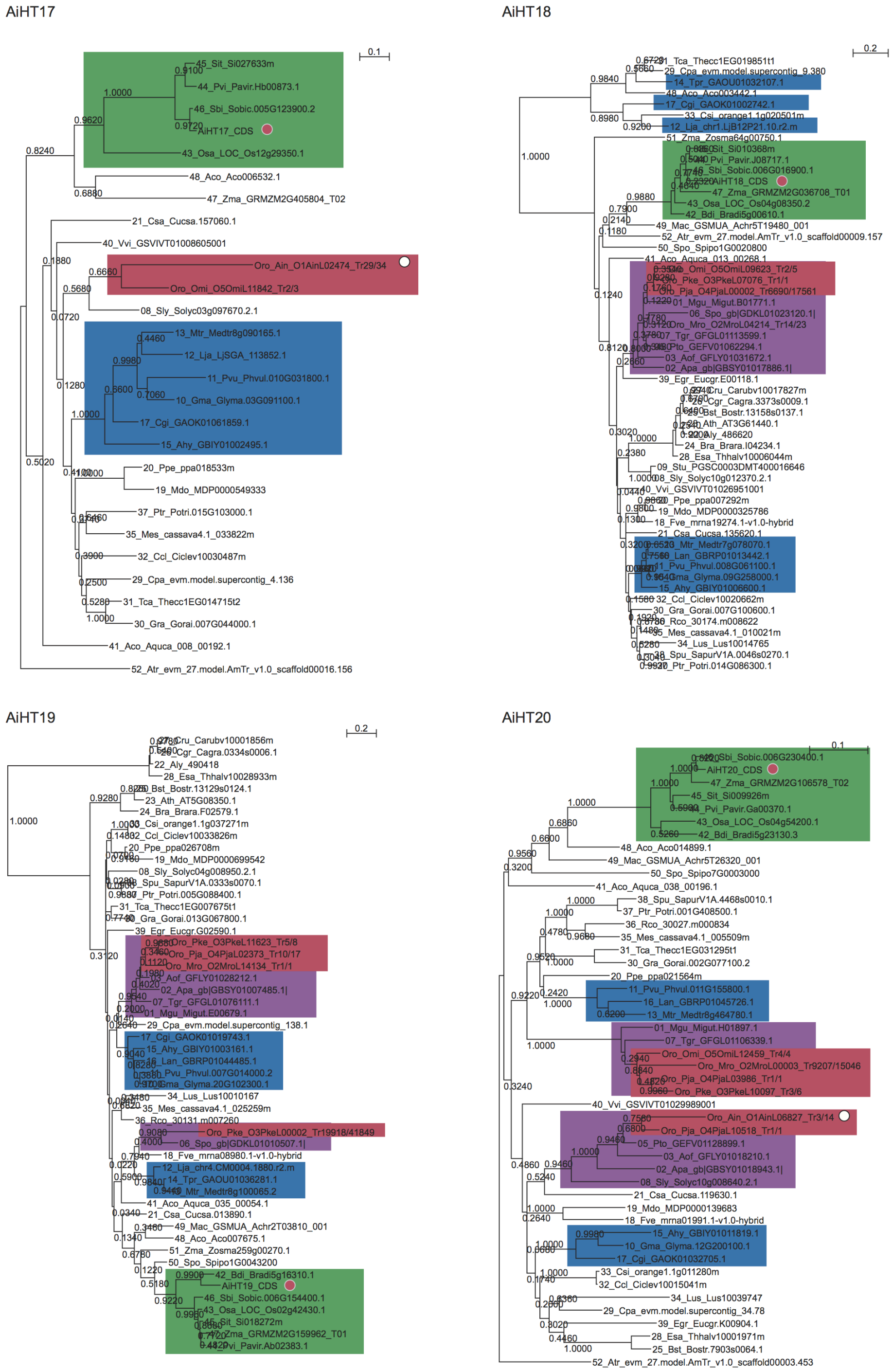

Figure S3 (12/27)

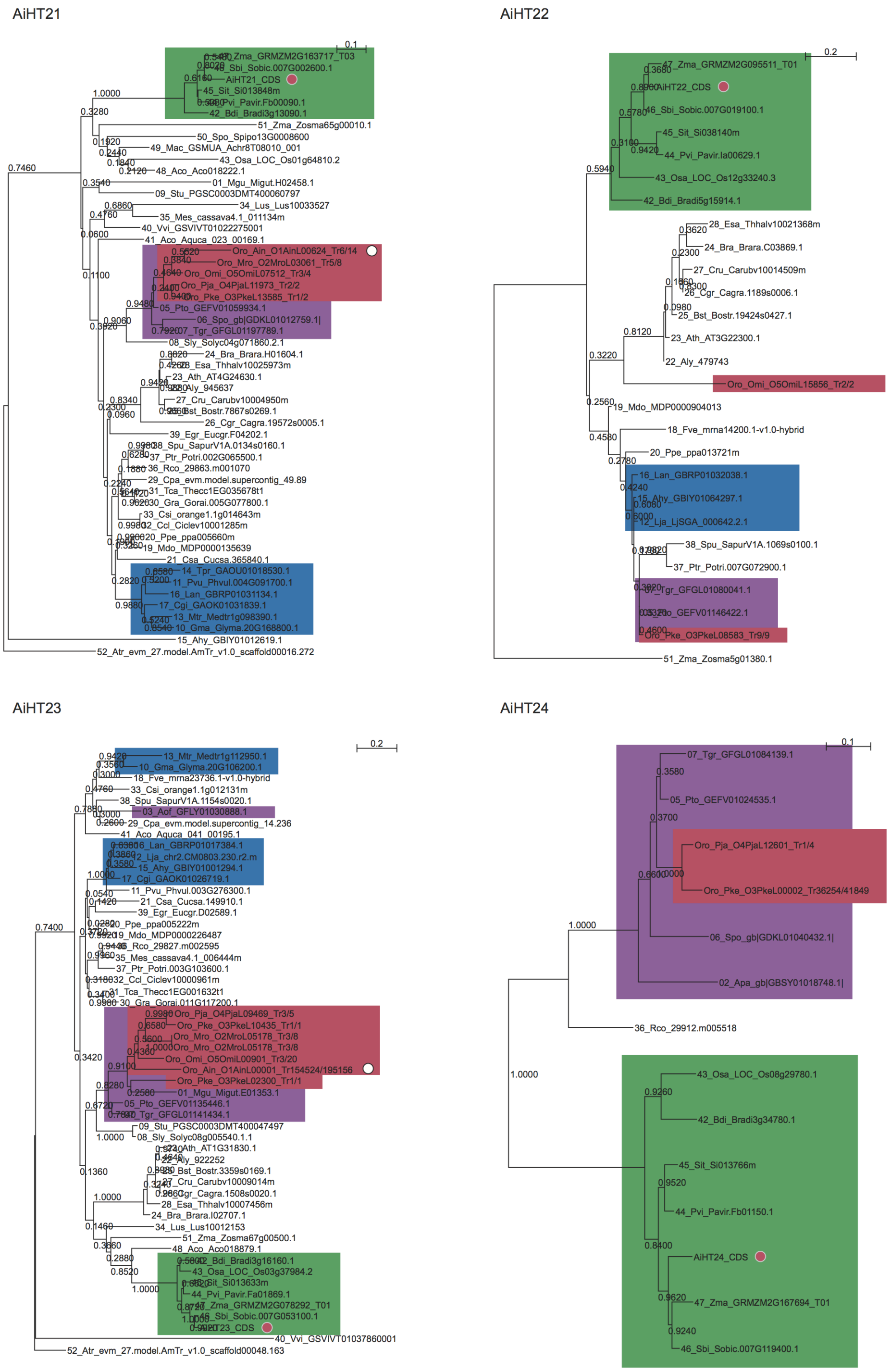

Figure S3 (13/27)

AiHT25

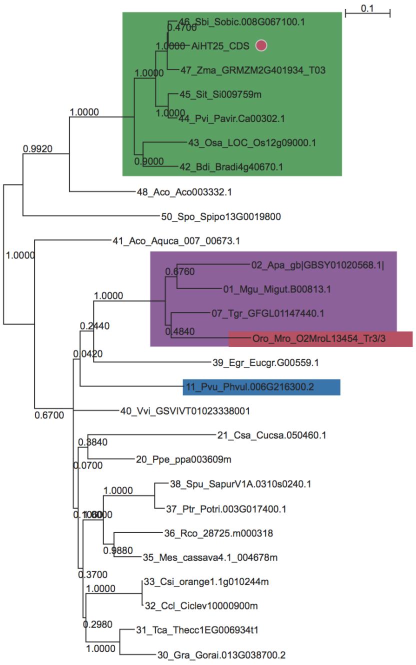

AiHT26

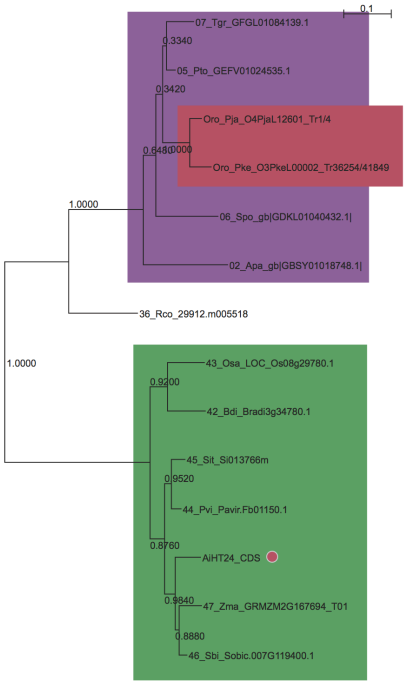

AiHT27

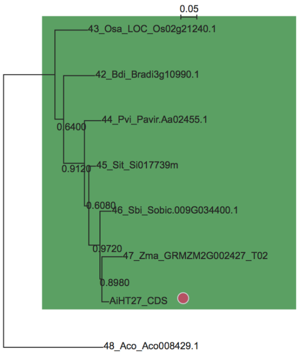

AiHT28

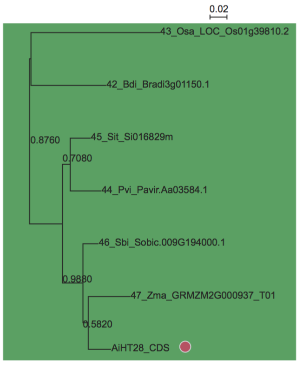

Figure S3 (14/27)

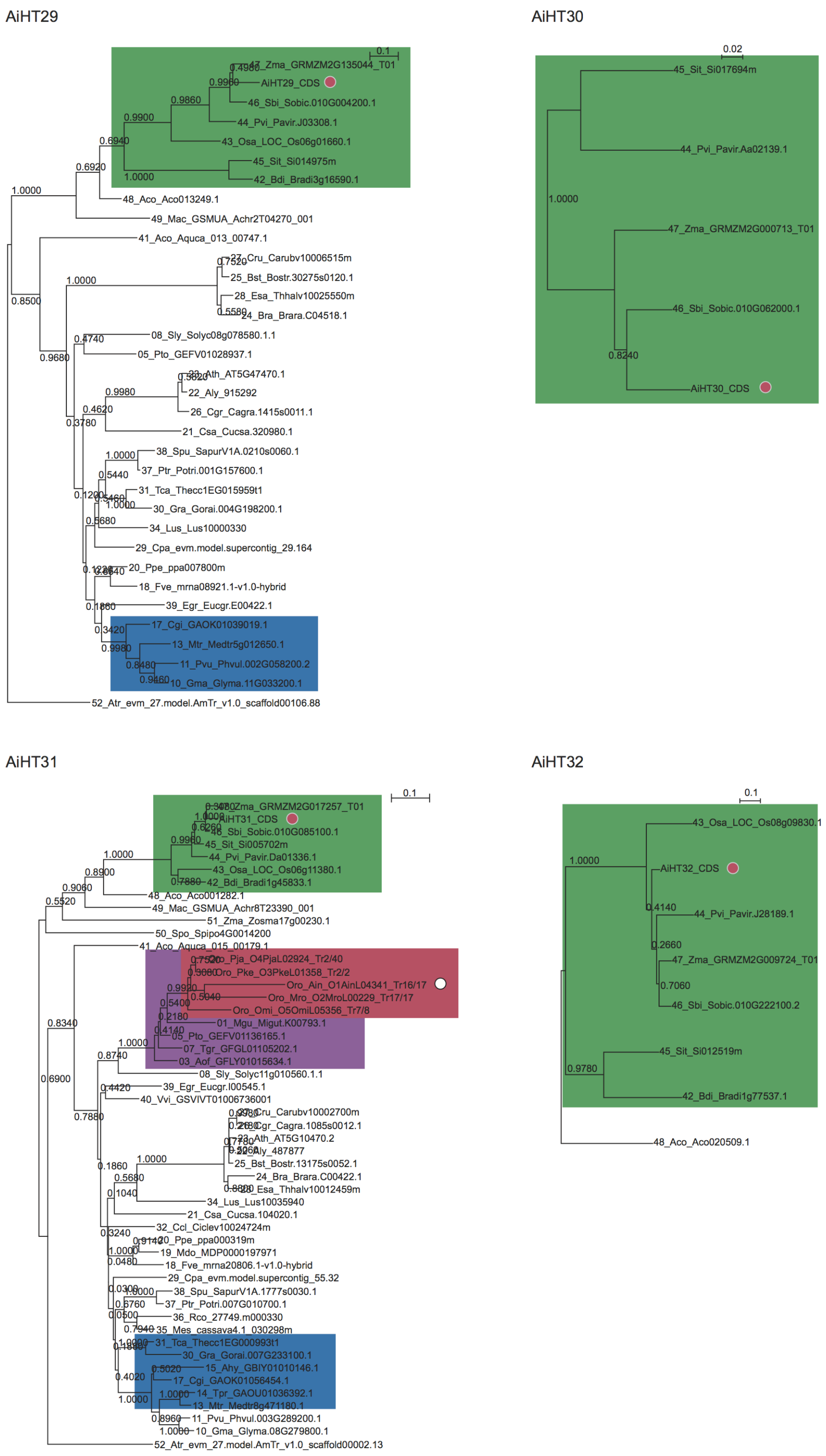

Figure S3 (15/27)

AiHT33

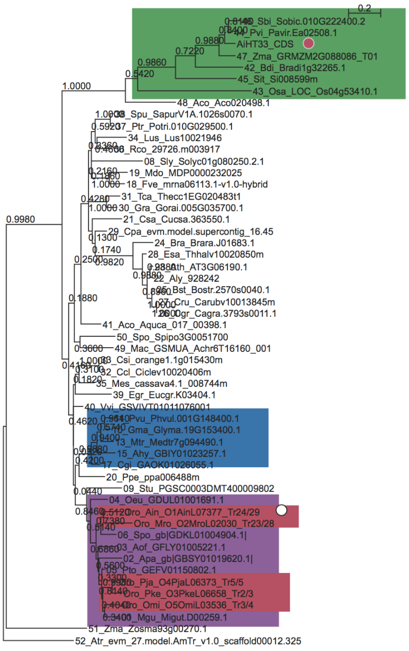

AiHT34

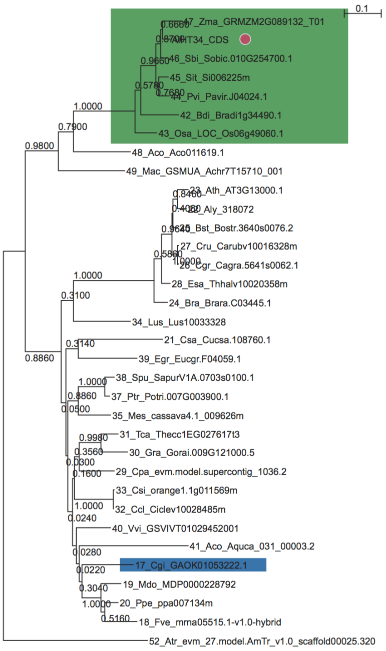

AiHT35

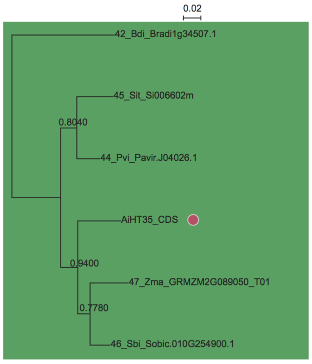

AiHT36

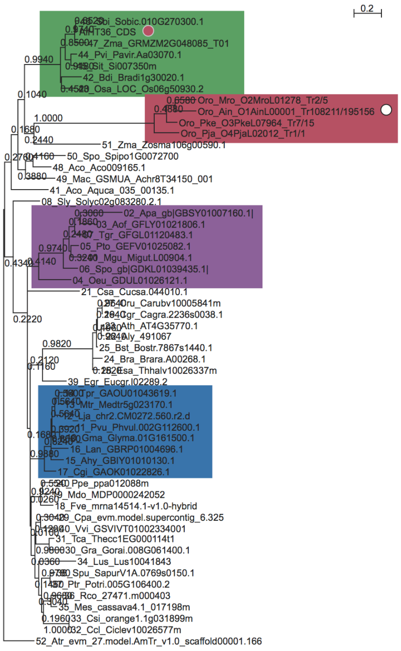

Figure S3 (16/27)

AiHT37

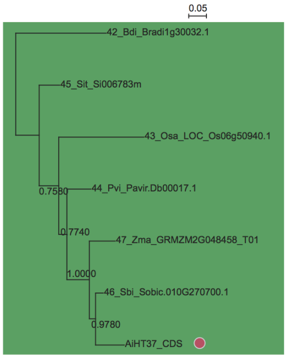

AiHT38

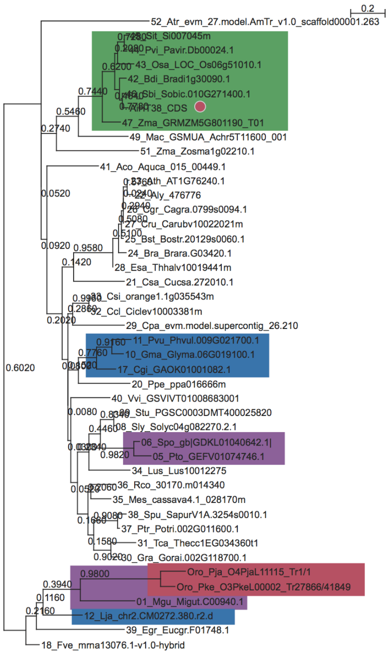

AiHT39

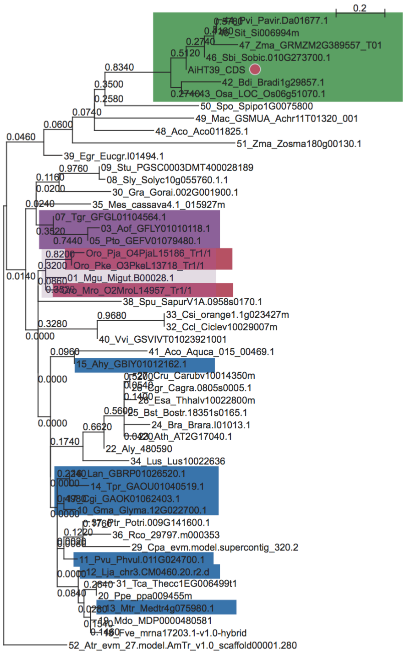

AiHT40

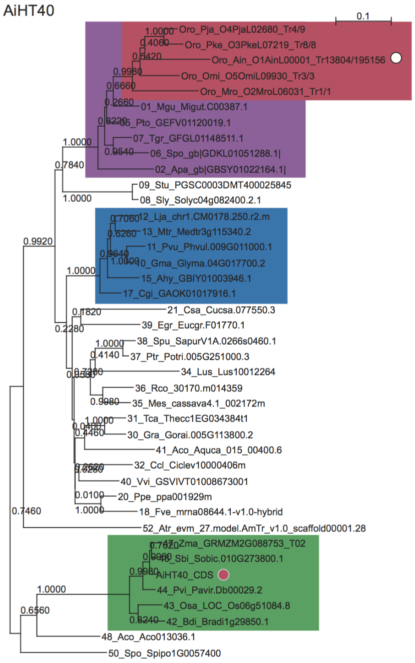

Figure S3 (17/27)

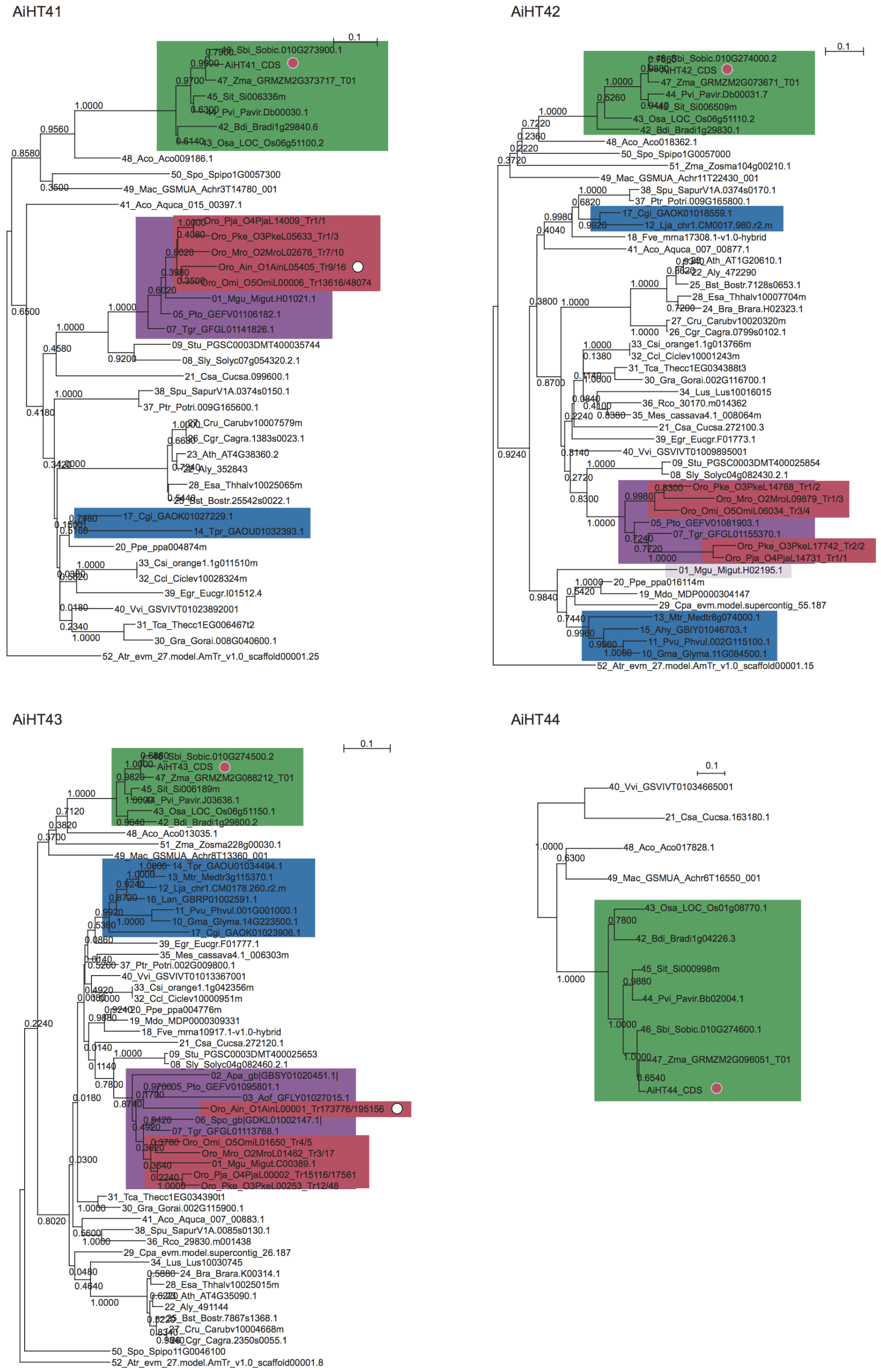

Figure S3 (18/27)

AiHT45

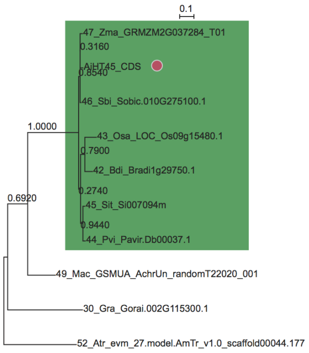

AiHT46

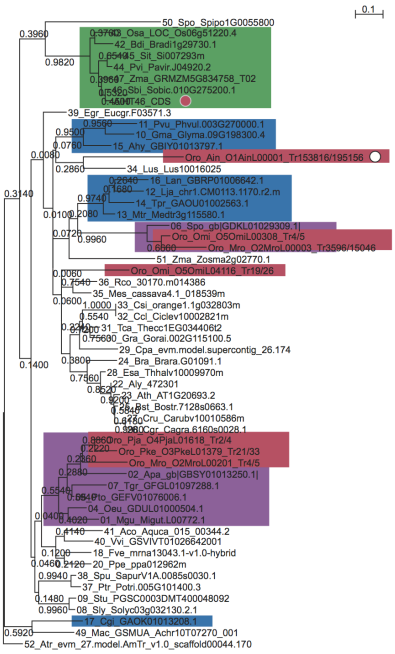

AiHT47

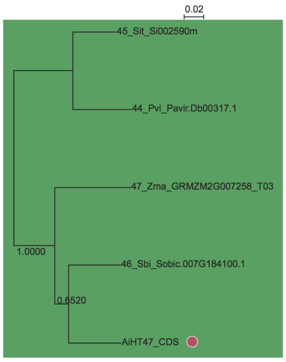

AiHT48

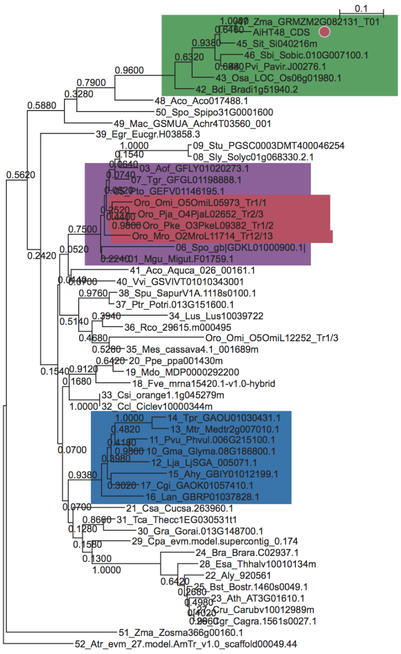

Figure S3 (19/27)

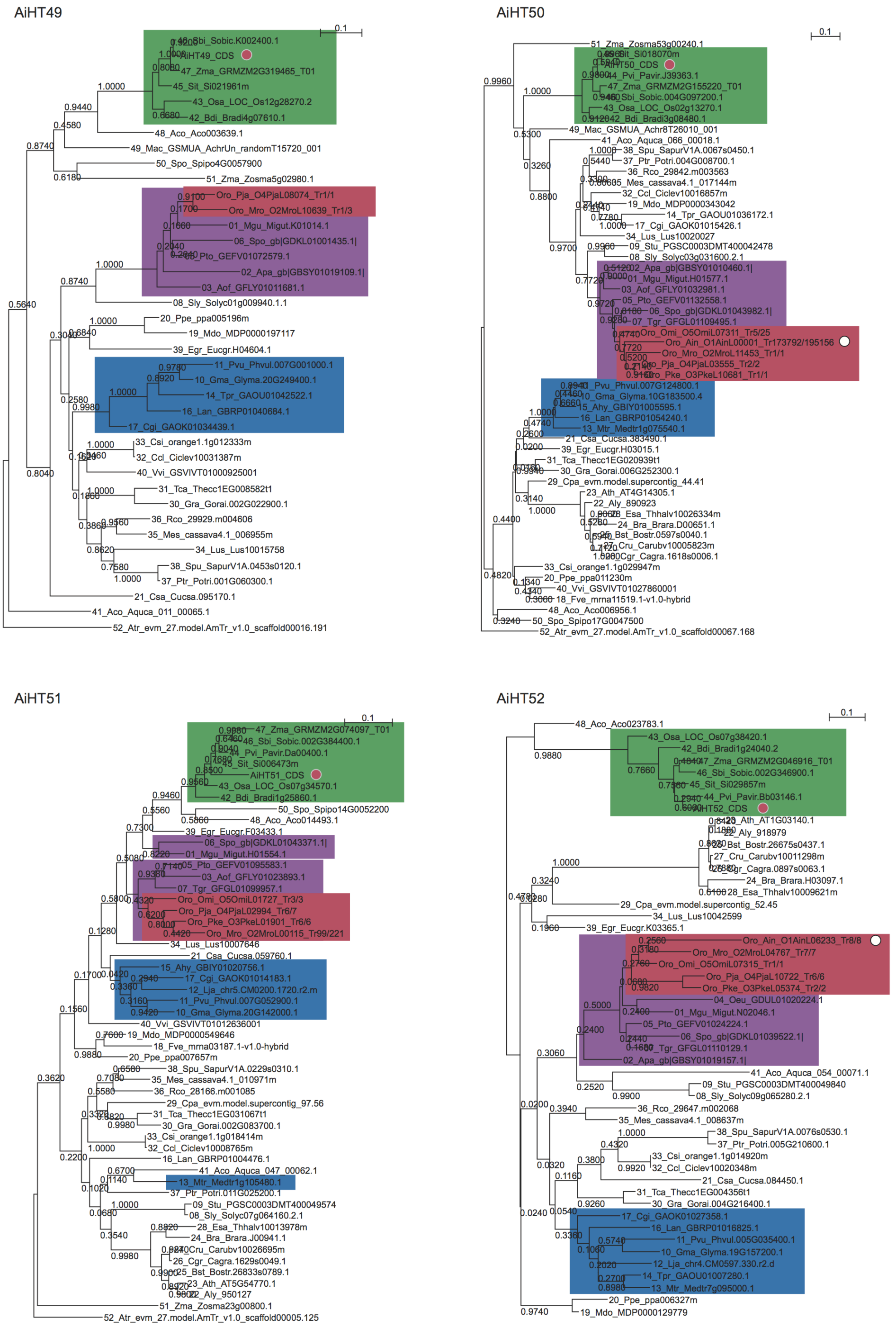

Figure S3 (20/27)

AiHT53

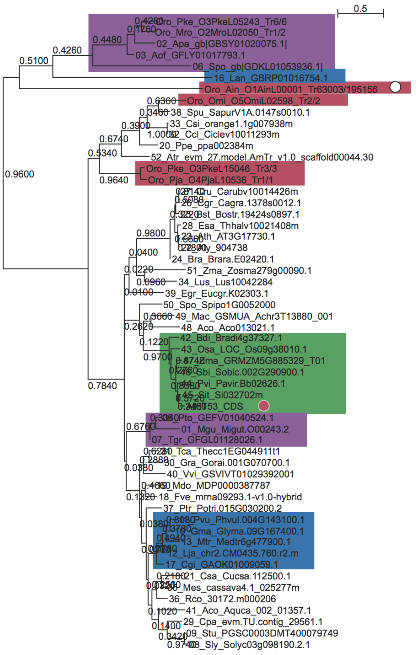

AiHT54

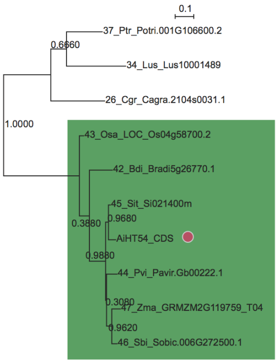

AiHT55

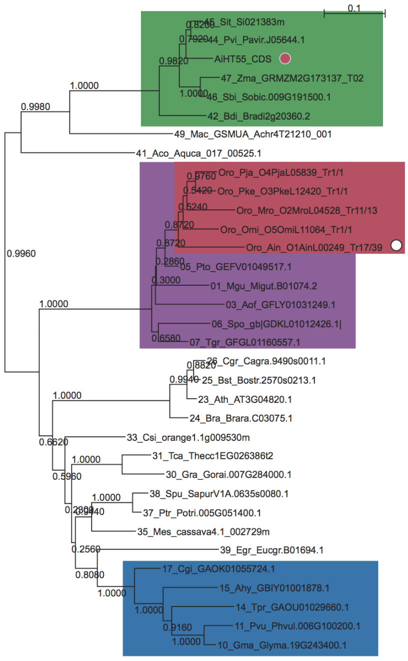

AiHT56

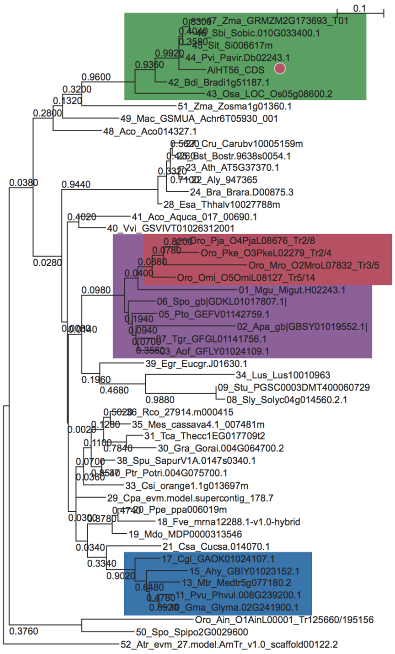

Figure S3 (21/27)

AiHT57

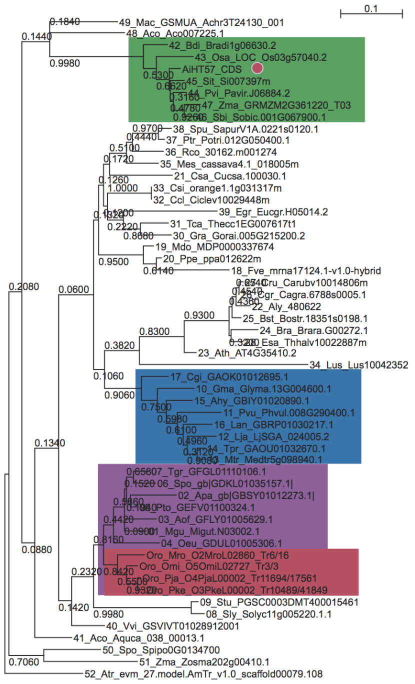

AiHT58

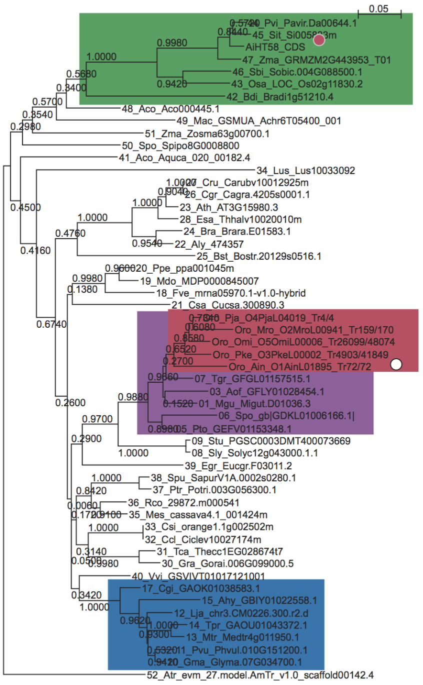

AiHT59

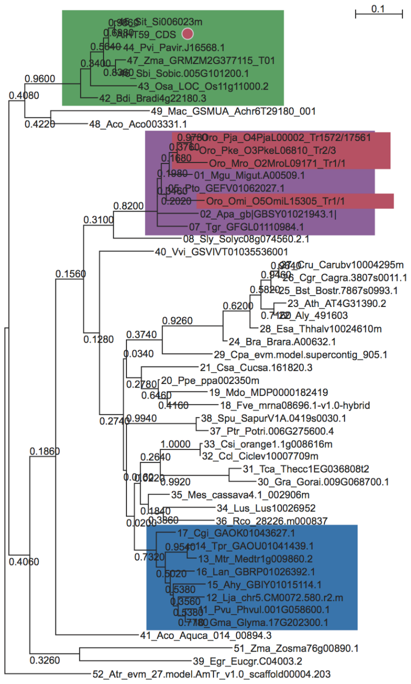

AiHT60

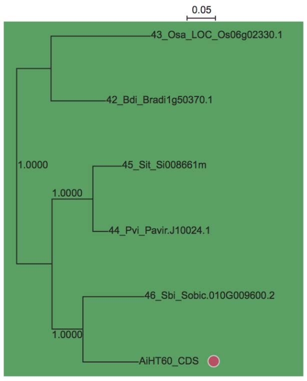

Figure S3 (22/27)

AiHT61

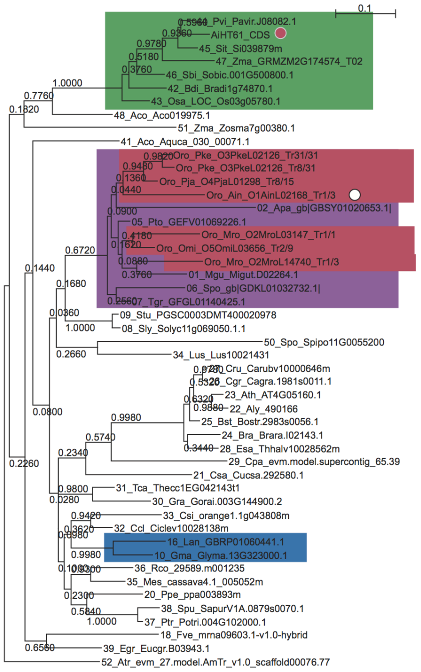

AiHT62

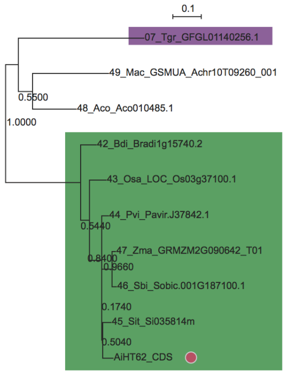

AiHT63

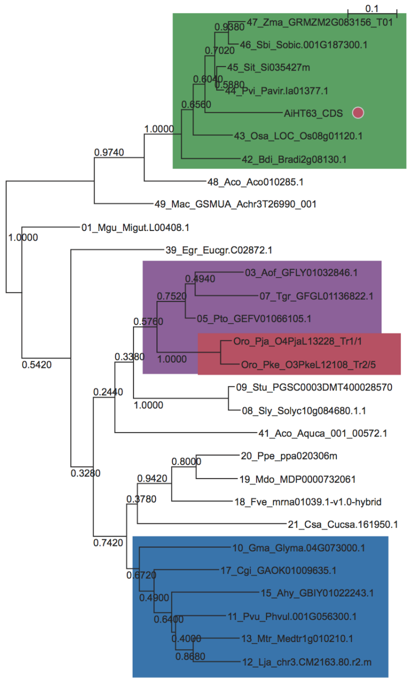

AiHT64

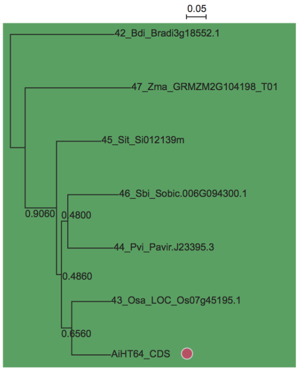

Figure S3 (23/27)

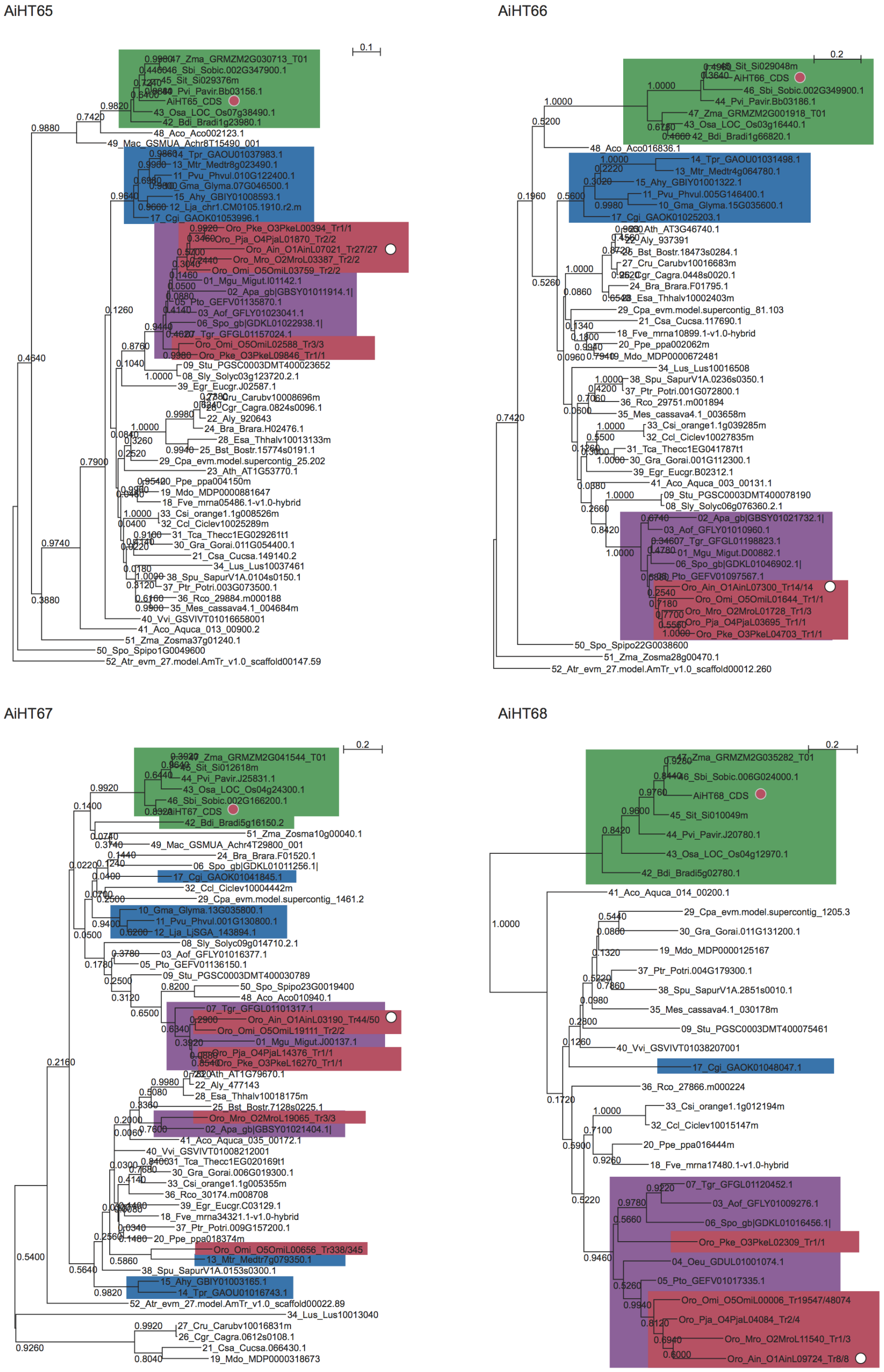

Figure S3 (24/27)

AiHT69

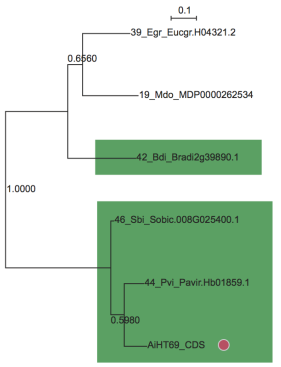

AiHT70

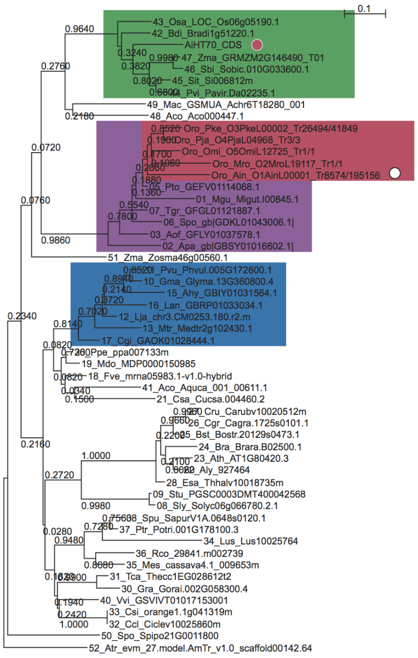

AiHT71

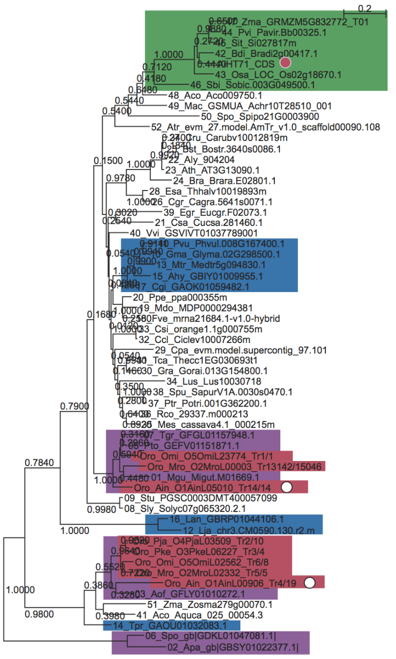

AiHT72

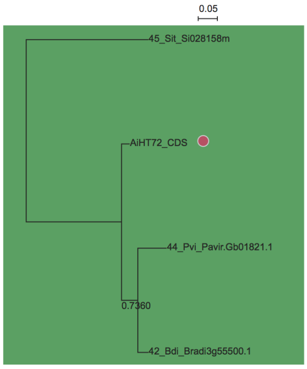

Figure S3 (25/27)

AiHT73

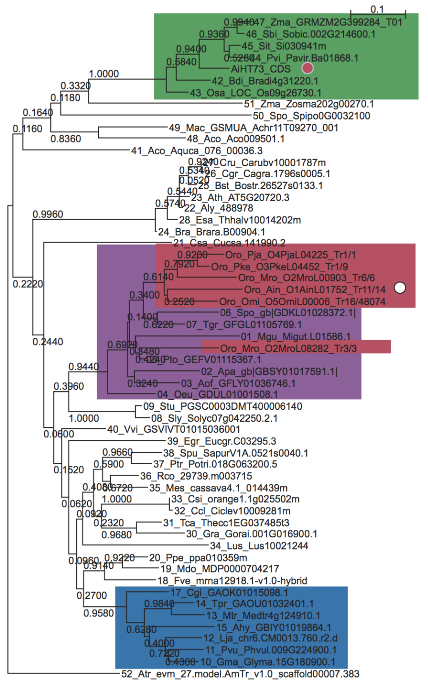

AiHT74

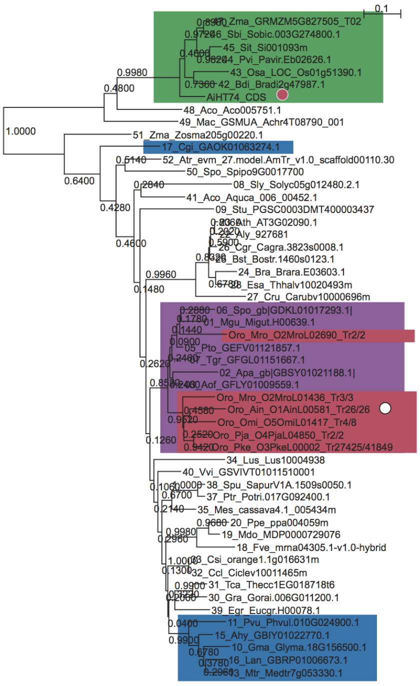

AiHT75

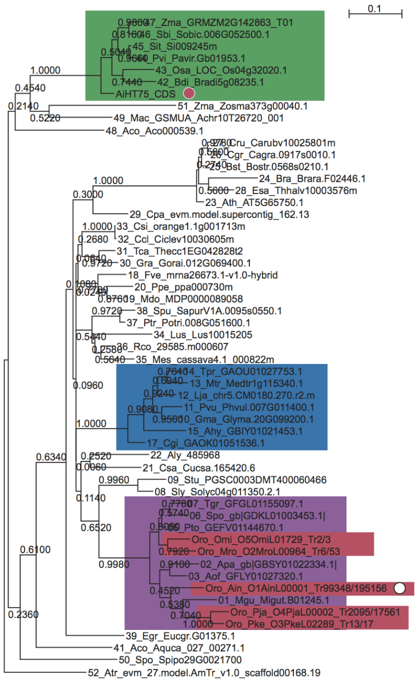

AiHT76

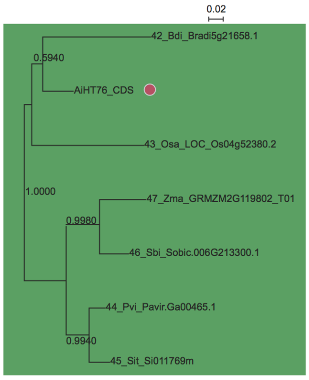

Figure S3 (26/27)

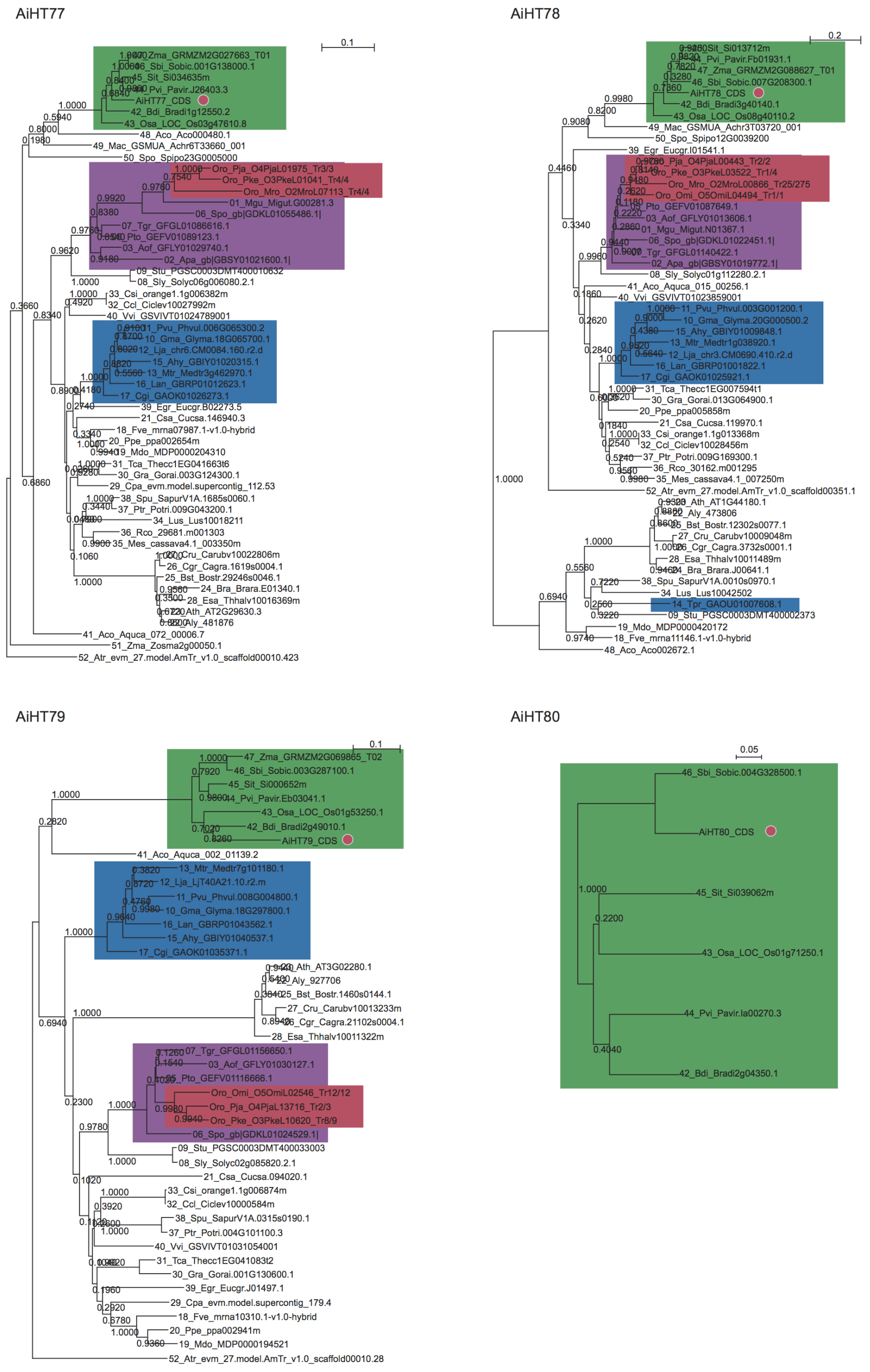

Figure S3 (27/27)

AiHT81

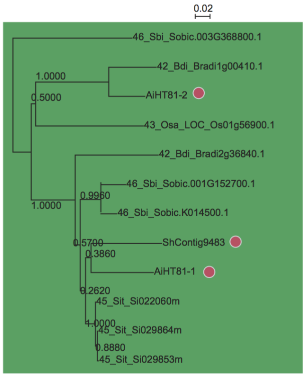

Figure S4

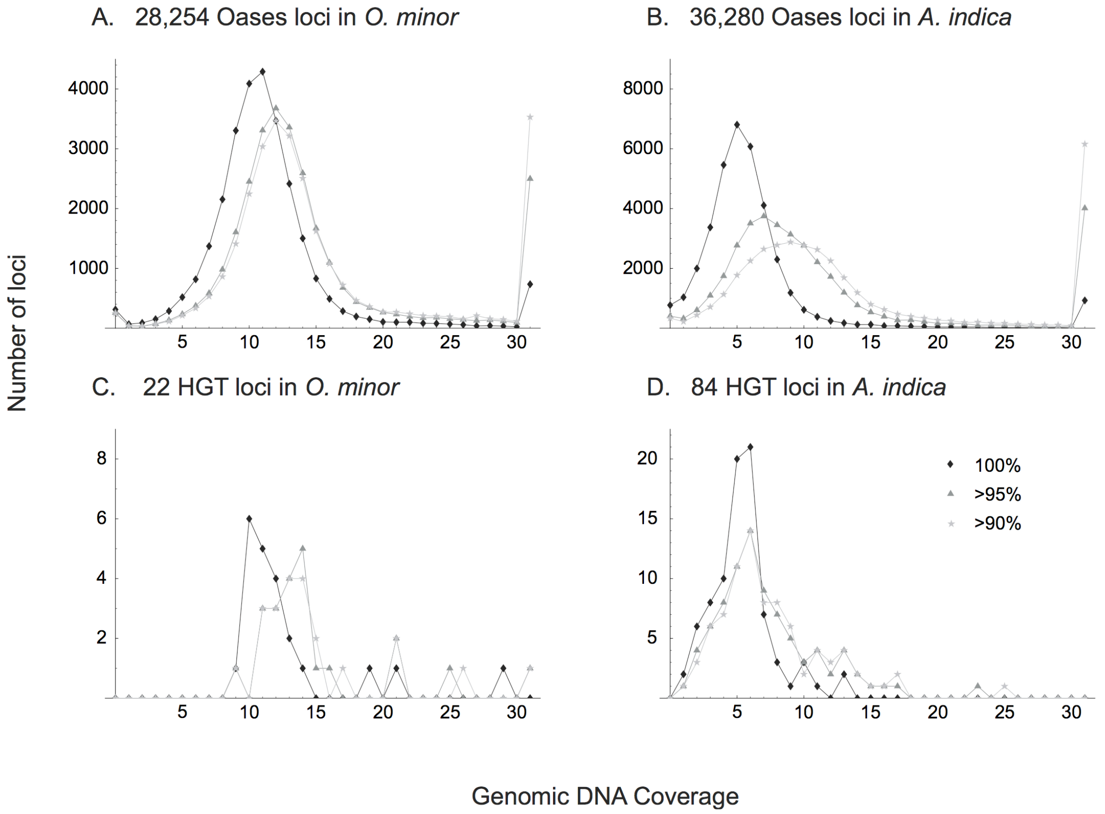

Figure S4 --- Genomic DNA coverage values compared between non-HGT genes and HGT genes (panels A vs. C for *O. minor* and B vs. D for *A. indica*). The Oases loci (see the main text) were used as representatives of non-HGT genes. Coverage values are based on BLASTN searches using transcript sequences (coding + UTR) as queries and DNA reads as databases. Median coverage value across sites was obtained for each Oases locus. The figure shows the distributions of coverage with three different criteria on identities, 100% (black diamonds), >95% (dark grey triangles), and >90% (light grey stars).

Figure S5

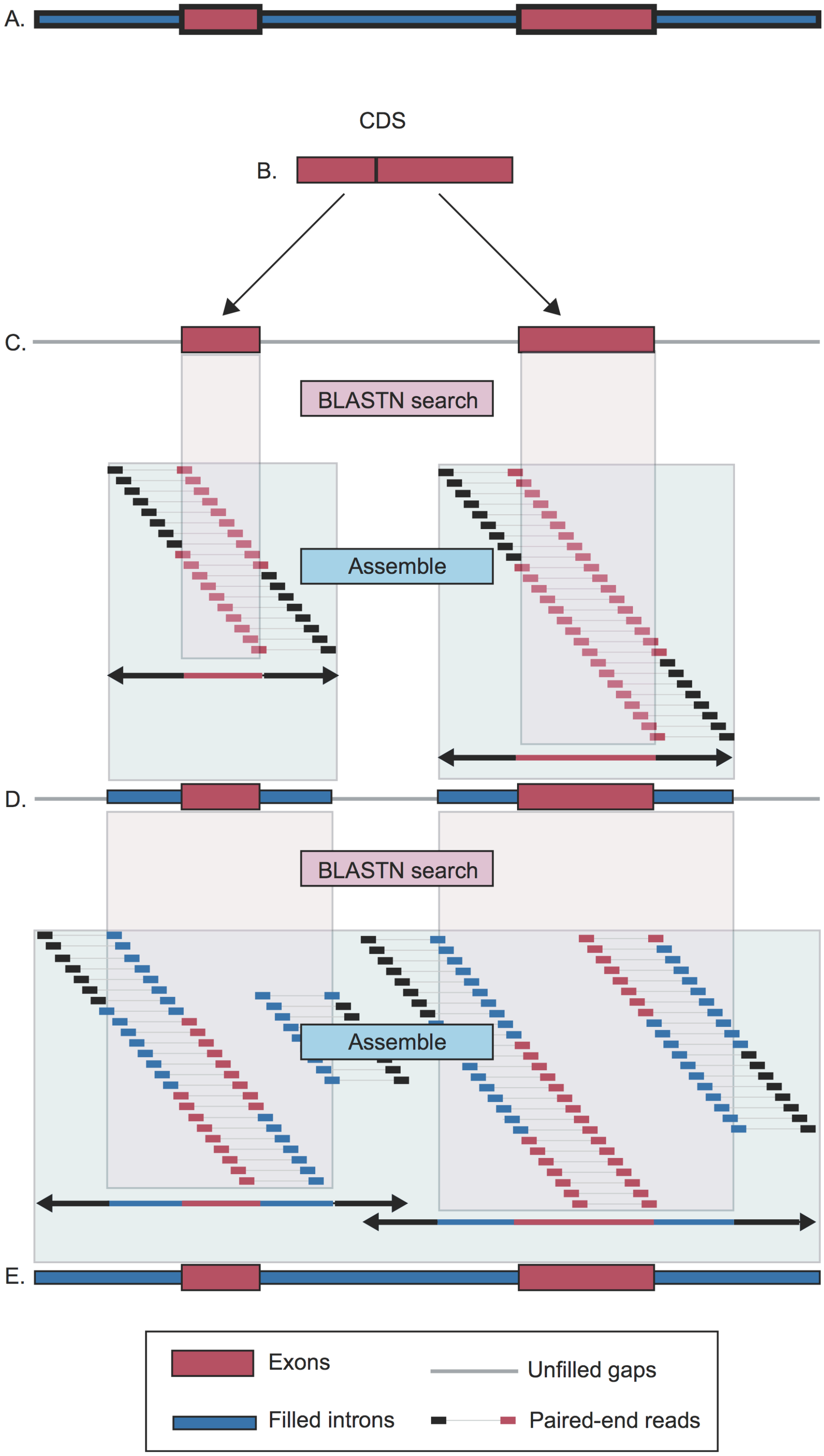
A.
CDS
B.
C.
BLASTN search
Assemble
D.
BLASTN search
Assemble
E.
Exons
Unfilled gaps
Filled introns
Paired-end reads

Figure S5 --- Reconstruction of genomic sequences based on the concatenated coding (i.e., exon) sequences of the 106 HGT genes. We used genomic sequences (paired-end short-read data) for assembling intron sequences with these coding sequences. The outline of our algorithm is illustrated, in which a hypothetical HGT gene with one intron is shown (A). (B) is the concatenated coding that consists of only exons to start with. On this sequence, we mapped genomic short-reads (C). The missing intron can be reconstructed mainly by using paired-end short-reads whose one read is well mapped within the exon region but the other is not (red-black and black-red reads in C). Short-reads were assembled by using phrap (http://www.phrap.org/ phredphrapconseed.html) and Se-Al (http://tree.bio.ed.ac.uk/software/seal/). After this process, the original assembled region was extended to some extent (boxed in blue). With this newly updated assembled sequence, we can then continue the same process, resulting in a further extended assembled sequence. This process was continued until the gap was completely filled (E). We have successfully filled the intron gaps with this procedure for more than 75% introns (see Table S6), and for the rest, unidentified gaps were remained.

Figure S6 (1/3)

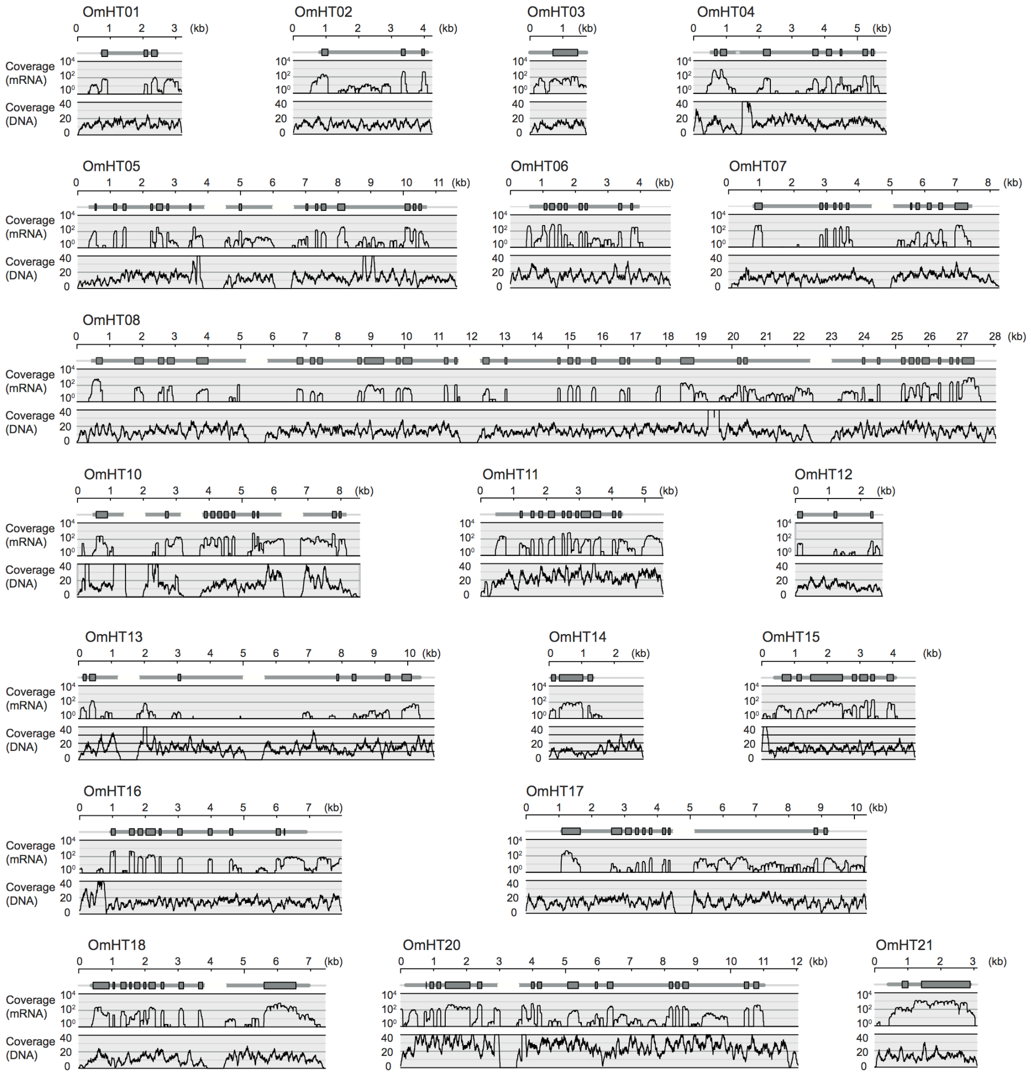

Figure S6 --- Exon-intron structure and coverage of mRNA and DNA reads.

Figure S6 (2/3)

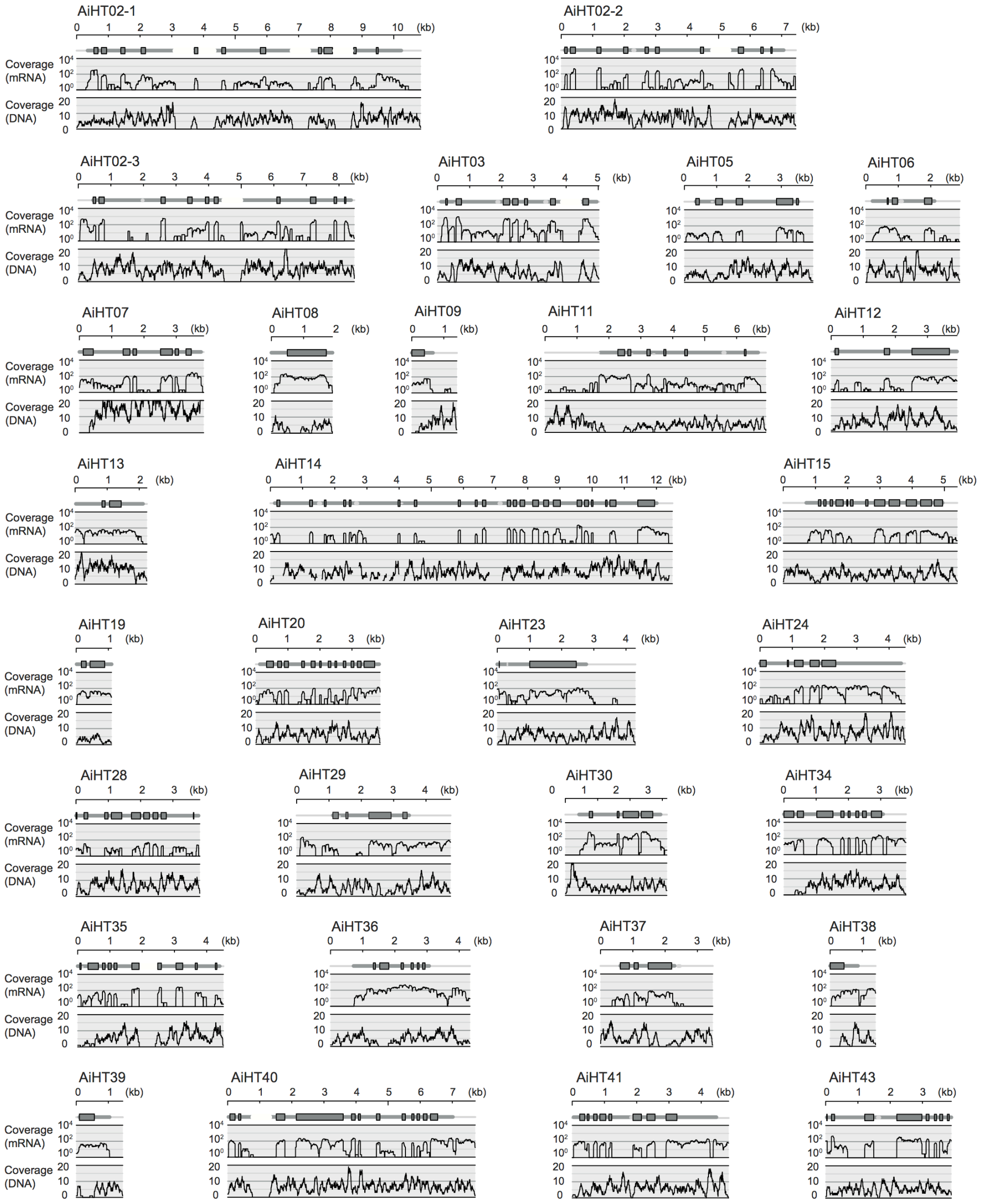
AiHT02-1
AiHT02-2
AiHT02-3
AiHT03
AiHT05
AiHT06
AiHT07
AiHT08
AiHT09
AiHT11
AiHT12
AiHT13
AiHT14
AiHT15
AiHT19
AiHT20
AiHT23
AiHT24
AiHT28
AiHT29
AiHT30
AiHT34
AiHT35
AiHT36
AiHT37
AiHT38
AiHT39
AiHT40
AiHT41
AiHT43
Coverage (mRNA)
Coverage (DNA)
(kb)

Figure S6 (3/3)

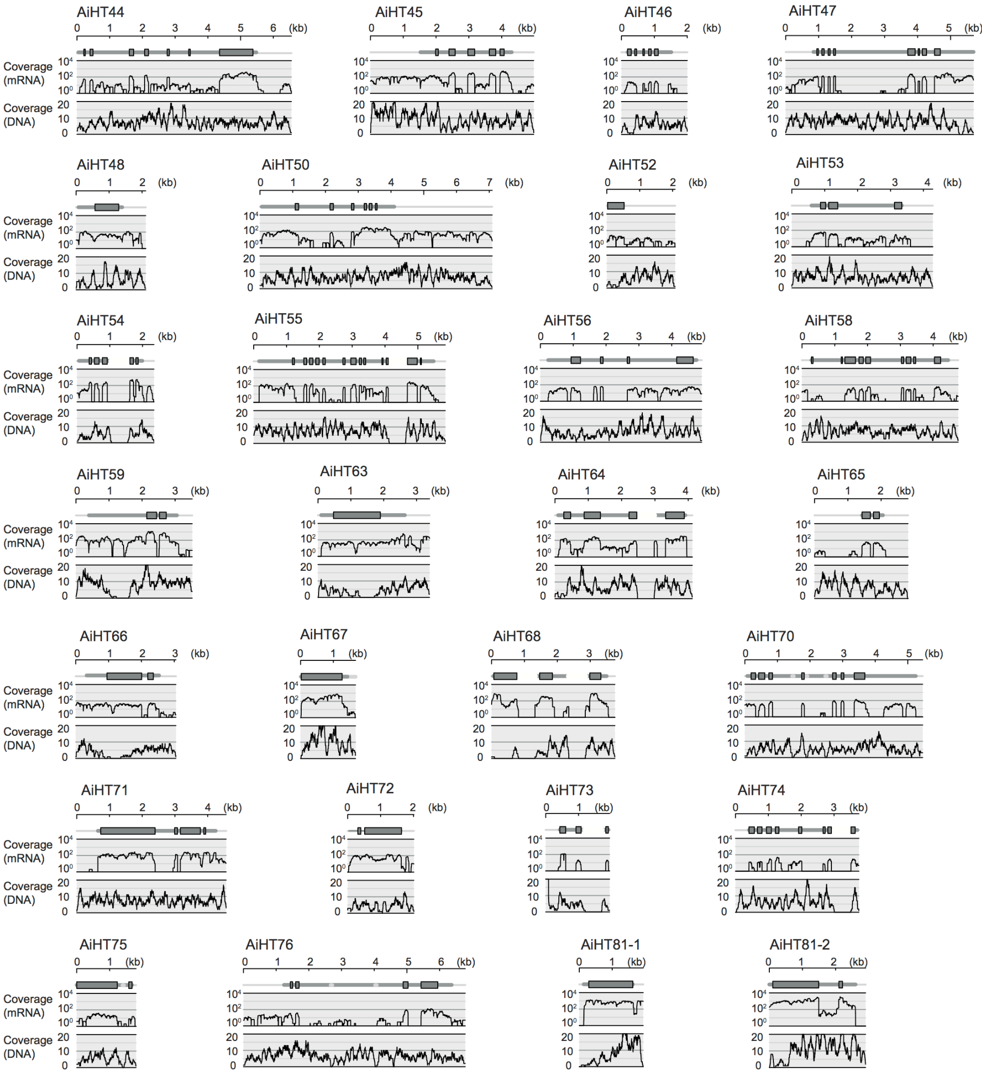
AiHT44
AiHT45
AiHT46
AiHT47
AiHT48
AiHT50
AiHT52
AiHT53
AiHT54
AiHT55
AiHT56
AiHT58
AiHT59
AiHT63
AiHT64
AiHT65
AiHT66
AiHT67
AiHT68
AiHT70
AiHT71
AiHT72
AiHT73
AiHT74
AiHT75
AiHT76
AiHT81-1
AiHT81-2
Coverage (mRNA)
Coverage (DNA)
(kb)
