# Supplementary material for: Horizontal Gene Transfer in Five Parasite Plant Species in Orobanchaceae
Source: Genome Biol Evol. 2018 Nov 8;10(12):3196–210. doi: 10.1093/gbe/evy219 (PMC6294234; doi:10.1093/gbe/evy219)
Supplement: Supplementary Data [file evy219_supp.zip › Suppl_Tables_H75_all.pdf]

Table S1 Summary of mRNA reads and the results of Oases

Species	Library	Tissues used for RNA extraction	Sampling locations	# of reads	length of reads	insert	total length	# of loci	# of transcripts
O. minor	Total	stem, flower bud, flower, root	Kanagawa, Japan	79,298,481	90bp	200bp	14.27Gb	28,254	162,409
	Library A	stem, flower bud	-	31,657,768	-	-	-	-	-
	Library B	stem, flower bud, flower	-	31,373,736	-	-	-	-	-
	Library C	stem, flower bud, root	-	16,266,977	-	-	-	-	-
A. indica	Total	flower, seed, stem	Cultivated	103,332,083	90bp	200bp	18.60Gb	36,280	434,426
	Library A	flower, seed, stem	-	103,332,083	-	-	-	-	-
P. keiskei	Total	leaf, winter bud, root, seed	Nagano, Japan	60,533,172	90bp	200bp	10.90Gb	20,584	131,636
	Library A	leaf, winter bud	-	19,350,793	-	-	-	-	-
	Library B	root	-	20,305,776	-	-	-	-	-
	Library C	seed	-	20,876,603	-	-	-	-	-
P. japonicum	Total	flower, seed, root, leaf	Shizuoka, Japan	33,760,268	90bp	200bp	6.08Gb	19,238	80,276
	Library A	flower, seed, root, leaf	-	33,760,268	-	-	-	-	-
M. roseum	Total	flower, seed, leaf, root	Shizuoka, Japan	52,998,402	90bp	200bp	9.54Gb	21,523	113,519
	Library A	flower, seed	-	19,571,921	-	-	-	-	-
	Library B	leaf, root	-	33,426,481	-	-	-	-	-

Table S2 Number of reads for genomic DNA

Species	Sampling locations	# of reads	length of reads	insert	total length
O. minor	Kanagawa, Japan	206,409,290	90bp	500bp	37.15Gb
A. indica	Cultivated	207,639,844	90bp	500bp	37.37Gb
P. keiskei	Nagano, Japan	76,957,394	90bp	500bp	13.85Gb
P. japonicum	Shizuoka, Japan	78,226,272	90bp	500bp	14.08Gb
M. roseum	Shizuoka, Japan	77,951,123	90bp	500bp	14.03Gb

Table S3 **Genome data used for screening and homology search of HGT genes**

Species	Abbreviation1*	Abbreviation2**	Family	Order	Higher Categories	FASTA files (CDS or TSA)	FASTA files (genome assembly)	Database	Data Type	Usage
Mimulus guttatus	Mgu	1_Mgu	Phrymaceae	Lamiales	Asterids	Mguttatus_256_v2.0.cds.fa	Mguttatus_256_v2.0.fasta	PhytozomeV10	CDS	Scereening / Homology search
Glycine max	Gma	10_Gma	Fabaceae	Fabales	Fabids / Rosids	Gmax_275_Wm82.a2.v1.cds.fa	Gmax_275_v2.0.fasta	PhytozomeV10	CDS	Scereening / Homology search
Phaseolus vulgaris	Pvu	11_Pvu	Fabaceae	Fabales	Fabids / Rosids	Pvulgaris_218_v1.0.cds.fa	Pvulgaris_218_v1.0.fasta	PhytozomeV10	CDS	Scereening / Homology search
Lotus japonicus	Lja	12_Lja	Fabaceae	Fabales	Fabids / Rosids	Lj2.5_cds.fa	Lj2.5_pseudomol.fasta	Kazusa	CDS	Scereening / Homology search
Medicago truncatula	Mtr	13_Mtr	Fabaceae	Fabales	Fabids / Rosids	Mtruncatula_285_Mt4.0v1.cds.fa	Mtruncatula_285_Mt4.0.fasta	PhytozomeV10	CDS	Scereening / Homology search
Trifolium pratense	Tpr	14_Tpr	Fabaceae	Fabales	Fabids / Rosids	GAOU01.1.fsa_nt	-	NCBI	TSA	Scereening / Homology search
Arachis hypogaea	Ahy	15_Ahy	Fabaceae	Fabales	Fabids / Rosids	GBIY01.1.fsa_nt	-	NCBI	TSA	Scereening / Homology search
Lupinus angustifolius	Lan	16_Lan	Fabaceae	Fabales	Fabids / Rosids	GBRP01.1.fsa_nt	-	NCBI	TSA	Scereening / Homology search
Cercis gigantea	Cgi	17_Cgi	Fabaceae	Fabales	Fabids / Rosids	GAOK01.1.fsa_nt	-	NCBI	TSA	Scereening / Homology search
Brachypodium distachyon	Bdi	42_Bdi	Poaceae	Poales	commelinids / Monocots	Bdistachyon_283_v2.1.cds.fa	Bdistachyon_283_assembly_v2.0.fasta	PhytozomeV10	CDS	Scereening / Homology search
Oryza sativa	Osa	43_Osa	Poaceae	Poales	commelinids / Monocots	Osativa_204_v7.0.cds.fa	Osativa_204_v7.0.fasta	PhytozomeV10	CDS	Scereening / Homology search
Panicum virgatum	Pvi	44_Pvi	Poaceae	Poales	commelinids / Monocots	Pvirgatum_273_v1.1.cds.fa	Pvirgatum_273_v1.0.fasta	PhytozomeV10	CDS	Scereening / Homology search
Setaria italica	Sit	45_Sit	Poaceae	Poales	commelinids / Monocots	Sitalica_164_v2.1.cds.fa	Sitalica_164_v2.fasta	PhytozomeV10	CDS	Scereening / Homology search
Sorghum bicolor	Sbi	46_Sbi	Poaceae	Poales	commelinids / Monocots	Sbicolor_255_v2.1.cds.fa	Sbicolor_255_v2.0.fasta	PhytozomeV10	CDS	Scereening / Homology search
Zea mays	Zma	47_Zma	Poaceae	Poales	commelinids / Monocots	Zmays_284_6a.cds.fa	Zmays_284_AGPv3.fasta	PhytozomeV10	CDS	Scereening / Homology search
Andrographis paniculata		2_Apa	Acanthaceae	Lamiales	Asterids	GBSY01.1.fa	-	NCBI	TSA	Homology search
Avicennia officinalis		3_Aof	Acanthaceae	Lamiales	Asterids	GFLY01.1.fsa_nt	-	NCBI	TSA	Homology search
Olea europaea		4_Oeu	Oleaceae	Lamiales	Asterids	GDUL01.1.fsa_nt	-	NCBI	TSA	Homology search
Paulownia tomentosa		5_Pto	Paulowniaceae	Lamiales	Asterids	GEFV01.1.fsa_nt	-	NCBI	TSA	Homology search
Salvia pomifera		6_Spo	Lamiaceae	Lamiales	Asterids	GDKL01.1.fsa_nt	-	NCBI	TSA	Homology search
Tectona grandis		7_Tgr	Lamiaceae	Lamiales	Asterids	GFGL01.1.fsa_nt	-	NCBI	TSA	Homology search
Solanum lycopersicum		8_Sly	Solanaceae	Solanales	Asterids	Slycopersicum_225_iTAGv2.3.cds.fa	-	PhytozomeV10	CDS	Homology search
Solanum tuberosum		9_Stu	Solanaceae	Solanales	Asterids	Stuberosum_206_v3.4.cds.fa	-	PhytozomeV10	CDS	Homology search
Fragaria vesca		18_Fve	Rosaceae	Rosales	Fabids / Rosids	Fvesca_226_v1.1.cds.fasta	-	PhytozomeV10	CDS	Homology search
Malus domestica		19_Mdo	Rosaceae	Rosales	Fabids / Rosids	Mdomestica_196_v1.0.cds.fasta	-	PhytozomeV10	CDS	Homology search
Prunus persica		20_Ppe	Rosaceae	Rosales	Fabids / Rosids	Ppersica_139_v1.0.cds.fasta	-	PhytozomeV10	CDS	Homology search
Cucumis sativus		21_Csa	Cucurbitaceae	Cucurbitales	Fabids / Rosids	Csativus_122_v1.0.cds.fasta	-	PhytozomeV10	CDS	Homology search
Arabidopsis lyrata		22_Aly	Brassicaceae	Brassicales	Malvids / Rosids	Alyrata_107_v1.0.cds.fasta	-	PhytozomeV10	CDS	Homology search
Arabidopsis thaliana		23_Ath	Brassicaceae	Brassicales	Malvids / Rosids	Athaliana_167_TAIR10.cds.fasta	-	PhytozomeV10	CDS	Homology search
Brassica rapa		24_Bra	Brassicaceae	Brassicales	Malvids / Rosids	BrapaFPsc_277_v1.3.cds.fasta	-	PhytozomeV10	CDS	Homology search
Boechera stricta		25_Bst	Brassicaceae	Brassicales	Malvids / Rosids	Bstricta_278_v1.2.cds.fasta	-	PhytozomeV10	CDS	Homology search
Capsella grandiflora		26_Cgr	Brassicaceae	Brassicales	Malvids / Rosids	Cgrandiflora_266_v1.1.cds.fasta	-	PhytozomeV10	CDS	Homology search
Capsella rubella		27_Cru	Brassicaceae	Brassicales	Malvids / Rosids	Crubella_183_v1.0.cds.fa	-	PhytozomeV10	CDS	Homology search
Eutrema salsugineum		28_Esa	Brassicaceae	Brassicales	Malvids / Rosids	Esalsugineum_173_v1.0.cds.fa	-	PhytozomeV10	CDS	Homology search
Carica papaya		29_Cpa	Caricaceae	Brassicales	Malvids / Rosids	Cpapaya_113_ASGPBv0.4.cds.fa	-	PhytozomeV10	CDS	Homology search
Gossypium raimondii		30_Gra	Malvaceae	Malvales	Malvids / Rosids	Graimondii_221_v2.1.cds.fa	-	PhytozomeV10	CDS	Homology search
Theobroma cacao		31_Tca	Malvaceae	Malvales	Malvids / Rosids	Tcacao_233_v1.1.cds.fa	-	PhytozomeV10	CDS	Homology search
Citrus clementina		32_Ccl	Rutaceae	Sapindales	Malvids / Rosids	Cclementina_182_v1.0.cds.fa	-	PhytozomeV10	CDS	Homology search
Citrus sinensis		33_Csi	Rutaceae	Sapindales	Malvids / Rosids	Csinensis_154_v1.1.cds.fa	-	PhytozomeV10	CDS	Homology search
Linum usitatissimum		34_Lus	Linaceae	Malpighiales	Malvids / Rosids	Lusitatissimum_200_v1.0.cds.fa	-	PhytozomeV10	CDS	Homology search
Manihot esculenta		35_Mes	Euphorbiaceae	Malpighiales	Malvids / Rosids	Mesculenta_147_v4.1.cds.fasta	-	PhytozomeV10	CDS	Homology search
Ricinus communis		36_Rco	Euphorbiaceae	Malpighiales	Malvids / Rosids	Rcommunis_119_v0.1.cds.fasta	-	PhytozomeV10	CDS	Homology search
Populus trichocarpa		37_Ptr	Salicaceae	Malpighiales	Malvids / Rosids	Ptrichocarpa_210_v3.0.cds.fa	-	PhytozomeV10	CDS	Homology search
Salix purpurea		38_Spu	Salicaceae	Malpighiales	Malvids / Rosids	Spurpurea_289_v1.0.cds.fa	-	PhytozomeV10	CDS	Homology search
Eucalyptus grandis		39_Egr	Myrtaceae	Myrtales	Malvids / Rosids	Egrandis_201_v1.1.cds.fa	-	PhytozomeV10	CDS	Homology search
Vitis vinifera		40_Vvi	Vitaceae	Vitales	Rosids incertae sedis	Vvinifera_145_Genoscope.12X.cds.fa	-	PhytozomeV10	CDS	Homology search
Aquilegia coerulea		41_Aco	Ranunculaceae	early-diverging	Basal Eudicots	Acoerulea_195_v1.1.cds.fa	-	PhytozomeV10	CDS	Homology search
Ananas comosus		48_Aco	Bromeliaceae	Poales	commelinids / Monocots	Acomosus_321_v3.cds.fa	-	PhytozomeV12	CDS	Homology search
Musa acuminata		49_Mac	Musaceae	Zingiberales	commelinids / Monocots	Macuminata_304_v1.cds.fa	-	PhytozomeV12	CDS	Homology search
Spirodela polyrhiza		50_Spo	Araceae	Alismatales	Monocots	Spolyrhiza_290_v2.cds.fa	-	PhytozomeV12	CDS	Homology search
Zostera marina		51_Zma	Zosteraceae	Alismatales	Monocots	Zmarina_324_v2.2.cds.fa	-	PhytozomeV12	CDS	Homology search
Amborella trichopoda		52_Atr	Amborellaceae	Amborellales	Basal Magnoliophyta	Atrichopoda_291_v1.0.cds.fa	-	PhytozomeV12	CDS	Homology search

*: Abbreviations used in Figure 2, S1 and S2.
**: Abbreviations used in Figure 3 and S3.

Table S4 Summary of the screening process

Parasites	Host groups	Number of genes			
		before screening	1st screening	2nd screening	Final screening
O. minor	Fabaceae	28,254	3,183	818	22
A. indica	Fabaceae	36,280	2,140	633	0
P. keiskei	Fabaceae	20,584	3,247	685	0
P. japonicum	Fabaceae	19,238	3,318	547	0
M. roseum	Fabaceae	21,523	3,147	890	0
O. minor	Poaceae	28,254	928	132	0
A. indica	Poaceae	36,280	1,330	614	84
P. keiskei	Poaceae	20,584	1,176	178	0
P. japonicum	Poaceae	19,238	1,186	142	0
M. roseum	Poaceae	21,523	1,362	292	0

Table S5 List of homologs used for the phylogenetic analyses

Loci	Species	ID of CDSs or TSAs	Abbreviations*1	Products*2
OmHT01	*T. pratense*	RC.42229	Tpr	NoData
OmHT01	*M. truncatula*	Medtr1g017270.1	Mtr-1	FAD/NAD(P)-binding oxidoreductase family protein
OmHT01	*M. truncatula*	Medtr3g103510.1	Mtr-2*	FAD/NAD(P)-binding oxidoreductase family protein
OmHT01	*P. vulgaris*	Phvul.009G074500.1	Pvu*	FAD/NAD(P)-binding oxidoreductase family protein
OmHT01	*G. max*	Glyma.04G048200.1	Gma-1*	squalene epoxidase 2
OmHT01	*G. max*	Glyma.06G049500.1	Gma-2*	FAD/NAD(P)-binding oxidoreductase family protein
OmHT01	*G. max*	Glyma.U038300.1	Gma-3*	FAD/NAD(P)-binding oxidoreductase family protein
OmHT01	*A. hypogaea*	Contig4512	Ahy	NoData
OmHT01	*C. gigantea*	Cgm37964_c1.1	Cgi*	NoData
OmHT02	*T. pratense*	RC.10825	Tpr	NoData
OmHT02	*M. truncatula*	Medtr1g060070.1	Mtr	Iron-sulphur cluster biosynthesis family protein
OmHT02	*L. japonicus*	chr1.CM0010.110.r2.d	Lja	
OmHT02	*P. vulgaris*	Phvul.001G189600.1	Pvu	Iron-sulphur cluster biosynthesis family protein
OmHT02	*G. max*	Glyma.19G194700.2	Gma-1	Iron-sulphur cluster biosynthesis family protein
OmHT02	*G. max*	Glyma.03G195000.1	Gma-2	Iron-sulphur cluster biosynthesis family protein
OmHT02	*L. angstifolius*	comp4284_c0_seq1	Lan	NoData
OmHT02	*C. gigantea*	Cgm31016_c0.1	Cgi	NoData
OmHT03	*T. pratense*	RC.29156	Tpr	NoData
OmHT03	*M. truncatula*	Medtr3g007750.1	Mtr	Ribosomal RNA adenine dimethylase family protein
OmHT03	*L. japonicus*	LjSGA_048522.1	Lja	
OmHT03	*P. vulgaris*	Phvul.008G161800.1	Pvu	Ribosomal RNA adenine dimethylase family protein
OmHT03	*G. max*	Glyma.06G316500.1	Gma-1	Ribosomal RNA adenine dimethylase family protein
OmHT03	*G. max*	Glyma.06G315500.2	Gma-2	Ribosomal RNA adenine dimethylase family protein
OmHT03	*A. hypogaea*	Contig7937	Ahy	NoData
OmHT03	*C. gigantea*	Cgm29652_c0.1	Cgi	NoData
OmHT04	*T. pratense*	RC.34162	Tpr	NoData
OmHT04	*M. truncatula*	Medtr4g080790.1	Mtr	electron transfer flavoprotein alpha
OmHT04	*P. vulgaris*	Phvul.011G139500.1+60	Pvu	electron transfer flavoprotein alpha
OmHT04	*G. max*	Glyma.06G270300.1	Gma-1	electron transfer flavoprotein alpha
OmHT04	*G. max*	Glyma.12G134400.1	Gma-2	electron transfer flavoprotein alpha
OmHT04	*A. hypogaea*	Contig8171	Ahy	NoData
OmHT04	*L. angstifolius*	comp5187_c0_seq1	Lan	NoData
OmHT04	*C. gigantea*	Cgm19859_c0.1	Cgi	NoData
OmHT05	*T. pratense*	RC.32426	Tpr	NoData
OmHT05	*M. truncatula*	Medtr4g080800.1	Mtr-1	PDI-like 5-4
OmHT05	*M. truncatula*	Medtr2g083260.1	Mtr-2*	PDI-like 5-4
OmHT05	*L. japonicus*	chr3.CM1543.10.r2.m	Lja	
OmHT05	*P. vulgaris*	Phvul.011G139400.1	Pvu-1	PDI-like 5-4
OmHT05	*P. vulgaris*	Phvul.005G092800.1	Pvu-2*	PDI-like 5-3
OmHT05	*G. max*	Glyma.06G270200.1	Gma-1	PDI-like 5-4
OmHT05	*G. max*	Glyma.12G134500.1	Gma-2	PDI-like 5-4
OmHT05	*G. max*	Glyma.13G288600.1	Gma-3*	PDI-like 5-4
OmHT05	*G. max*	Glyma.12G213100.1	Gma-4*	PDI-like 5-4
OmHT05	*A. hypogaea*	Contig19234	Ahy	NoData
OmHT05	*L. angstifolius*	comp6488_c0_seq1	Lan	NoData
OmHT05	*C. gigantea*	Cgm37444_c0.2	Cgi	NoData
OmHT06	*T. pratense*	RC.13251	Tpr	NoData
OmHT06	*M. truncatula*	Medtr4g119840.1	Mtr	alanine-tRNA ligases;nucleic acid binding;ligases, forming ami
OmHT06	*L. japonicus*	LjSGA_011895.2	Lja	
OmHT06	*P. vulgaris*	Phvul.003G105100.1	Pvu	alanine-tRNA ligases;nucleic acid binding;ligases, forming ami
OmHT06	*L. angstifolius*	comp8551_c0_seq1	Lan	NoData
OmHT07	*T. pratense*	RC.41913	Tpr	NoData
OmHT07	*M. truncatula*	Medtr5g093580.1	Mtr	co-factor for nitrate, reductase and xanthine dehydrogenase 5
OmHT07	*L. japonicus*	LjSGA_024515.1	Lja	
OmHT07	*P. vulgaris*	Phvul.008G270700.1	Pvu	co-factor for nitrate, reductase and xanthine dehydrogenase 5
OmHT07	*G. max*	Glyma.02G293600.1	Gma-1	co-factor for nitrate, reductase and xanthine dehydrogenase 5
OmHT07	*G. max*	Glyma.14G019100.1	Gma-2	co-factor for nitrate, reductase and xanthine dehydrogenase 5
OmHT07	*L. angstifolius*	comp4069_c0_seq1	Lan	NoData
OmHT08	*T. pratense*	RC.37121	Tpr	NoData
OmHT08	*M. truncatula*	Medtr6g004840.1	Mtr	embryo defective 3012
OmHT08	*L. japonicus*	chr2.CM0263	Lja	
OmHT08	*P. vulgaris*	Phvul.004G006800.1	Pvu	embryo defective 3012
OmHT08	*G. max*	Glyma.05G003500.1	Gma	embryo defective 3012
OmHT08	*A. hypogaea*	Contig5516	Ahy	NoData
OmHT08	*C. gigantea*	Cgm41382_c0.4	Cgi	NoData
OmHT09	*T. pratense*	RC.41584	Tpr-1	NoData
OmHT09	*T. pratense*	RC.9423	Tpr-2*	NoData
OmHT09	*M. truncatula*	Medtr8g007390.1	Mtr-1	RNA-binding (RRM/RBD/RNP motifs) family protein
OmHT09	*M. truncatula*	Medtr7g081210.1	Mtr-2*	RNA-binding (RRM/RBD/RNP motifs) family protein
OmHT09	*P. vulgaris*	Phvul.010G044700.1	Pvu	RNA-binding (RRM/RBD/RNP motifs) family protein
OmHT09	*G. max*	Glyma.03G107700.1	Gma-1	RNA-binding (RRM/RBD/RNP motifs) family protein
OmHT09	*G. max*	Glyma.07G119000.1	Gma-2	RNA-binding (RRM/RBD/RNP motifs) family protein
OmHT09	*G. max*	Glyma.18G248200.1	Gma-3*	RNA-binding (RRM/RBD/RNP motifs) family protein
OmHT09	*A. hypogaea*	Contig12066	Ahy-1	NoData
OmHT09	*A. hypogaea*	Contig9142	Ahy-2*	NoData
OmHT09	*C. gigantea*	Cgm39612_c0.1	Cgi*	NoData
OmHT10	*T. pratense*	RC.42261	Tpr-1	NoData
OmHT10	*T. pratense*	RC.15611	Tpr-2	NoData
OmHT10	*M. truncatula*	Medtr8g446850.1	Mtr-1	UDP-glcnac-adolichol phosphate glcnac-1-p-transferase
OmHT10	*M. truncatula*	Medtr3g045150.1	Mtr-2	UDP-glcnac-adolichol phosphate glcnac-1-p-transferase
OmHT10	*M. truncatula*	Medtr0012s0090.1	Mtr-3	UDP-glcnac-adolichol phosphate glcnac-1-p-transferase
OmHT10	*L. japonicus*	chr5.CM0278.110.r2.m	Lja	
OmHT10	*P. vulgaris*	Phvul.007G249500.1	Pvu	UDP-glcnac-adolichol phosphate glcnac-1-p-transferase
OmHT10	*G. max*	Glyma.02G190700.1	Gma-1	UDP-glcnac-adolichol phosphate glcnac-1-p-transferase
OmHT10	*G. max*	Glyma.10G105900.1	Gma-2	UDP-glcnac-adolichol phosphate glcnac-1-p-transferase
OmHT10	*A. hypogaea*	Contig910	Ahy	NoData
OmHT10	*C. gigantea*	Cgm35537_c0.1	Cgi	NoData
OmHT11	*T. pratense*	RC.6350	Tpr-1	NoData
OmHT11	*T. pratense*	RC.42854	Tpr-2*	NoData
OmHT11	*M. truncatula*	Medtr3g048190.1	Mtr-1	Class II aaRS and biotin synthetases superfamily protein
OmHT11	*M. truncatula*	Medtr3g114570.1	Mtr-2	Class II aaRS and biotin synthetases superfamily protein
OmHT11	*M. truncatula*	Medtr7g110660.1	Mtr-3*	Class II aaRS and biotin synthetases superfamily protein
OmHT11	*L. japonicus*	chr2.CM0177.390.r2.m	Lja-1	
OmHT11	*L. japonicus*	chr1.CM0122.1320.r2.m	Lja-2	

OmHT11	*P. vulgaris*	Phvul.001G218300.1	Pvi	Class II aaRS and biotin synthetases superfamily protein
OmHT11	*G. max*	Glyma.19G221400.1	Gma-1	Class II aaRS and biotin synthetases superfamily protein
OmHT11	*G. max*	Glyma.03G224500.1	Gma-2	Class II aaRS and biotin synthetases superfamily protein
OmHT12	*T. pratense*	RC.37978	Tpr	NoData
OmHT12	*L. japonicus*	chr3.CM0005.510.r2.m	Lja	
OmHT12	*G. max*	Glyma.13G189600.1	Gma	microtubule-associated protein 65-5
OmHT12	*L. angstifolius*	comp55292_c0_seq1	Lan	NoData
OmHT12	*C. gigantea*	Cgm38411_c0.3	Cgi	NoData
OmHT13	*M. truncatula*	Medtr8g075480.1	Mtr	S-adenosyl-L-methionine-dependent methyltransferases super
OmHT13	*P. vulgaris*	Phvul.003G263600.1	Pvu	S-adenosyl-L-methionine-dependent methyltransferases super
OmHT13	*G. max*	Glyma.02G047300.1	Gma	S-adenosyl-L-methionine-dependent methyltransferases super
OmHT13	*A. hypogaea*	Contig64749	Ahy	NoData
OmHT13	*C. gigantea*	Cgm34438_c0.3	Cgi	NoData
OmHT14	*M. truncatula*	Medtr7g074850.1	Mtr-1	NAD(P)-binding Rossmann-fold superfamily protein
OmHT14	*M. truncatula*	Medtr7g074730.1	Mtr-2	NAD(P)-binding Rossmann-fold superfamily protein
OmHT14	*M. truncatula*	Medtr7g074820.1	Mtr-3	NAD(P)-binding Rossmann-fold superfamily protein
OmHT14	*P. vulgaris*	Phvul.008G076500.1	Pvu-1	NAD(P)-binding Rossmann-fold superfamily protein
OmHT14	*P. vulgaris*	Phvul.011G212600.1	Pvu-2*	NAD(P)-binding Rossmann-fold superfamily protein
OmHT14	*G. max*	Glyma.12G238200.1	Gma-1*	NAD(P)-binding Rossmann-fold superfamily protein
OmHT14	*G. max*	Glyma.13G203900.1	Gma-2*	NAD(P)-binding Rossmann-fold superfamily protein
OmHT14	*C. gigantea*	Cgm38754_c0.1	Cgi*	NoData
OmHT15	*M. truncatula*	Medtr3g052610.1	Mtr-1	RNA helicase family protein
OmHT15	*M. truncatula*	Medtr4g485610.1	Mtr-2	RNA helicase family protein
OmHT15	*M. truncatula*	Medtr1g086640.1	Mtr-3	RNA helicase family protein
OmHT15	*L. japonicus*	LjSGA_019513.1	Lja-1	
OmHT15	*L. japonicus*	chr1.CM0122.1830.r2.d	Lja-2*	
OmHT15	*P. vulgaris*	Phvul.001G214700.1	Pvu*	RNA helicase family protein
OmHT15	*G. max*	Glyma.02G010500.1	Gma-1	RNA helicase family protein
OmHT15	*G. max*	Glyma.19G218200.1	Gma-2*	RNA helicase family protein
OmHT15	*G. max*	Glyma.03G221200.1	Gma-3*	RNA helicase family protein
OmHT15	*A. hypogaea*	Contig16092	Ahy*	NoData
OmHT15	*C. gigantea*	Cgm36035_c0.3	Cgi	NoData
OmHT16	*T. pratense*	RC.27005	Tpr-1	NoData
OmHT16	*T. pratense*	RC.31901	Tpr-2	NoData
OmHT16	*M. truncatula*	Medtr5g064160.1	Mtr-1	Trypsin family protein with PDZ domain
OmHT16	*M. truncatula*	Medtr5g464730.1	Mtr-2	Trypsin family protein with PDZ domain
OmHT17	*T. pratense*	RC.31431	Tpr	NoData
OmHT17	*M. truncatula*	Medtr3g048950.1	Mtr	Amidase family protein
OmHT17	*L. japonicus*	chr6.CM0836.110.r2.d	Lja	
OmHT17	*P. vulgaris*	Phvul.006G036200.1	Pvu	Amidase family protein
OmHT17	*G. max*	Glyma.08G311400.1	Gma-1	Amidase family protein
OmHT17	*G. max*	Glyma.18G104200.1	Gma-2	Amidase family protein
OmHT17	*A. hypogaea*	Contig12411	Ahy	NoData
OmHT17	*C. gigantea*	Cgm39087_c0.1	Cgi	NoData
OmHT18	*T. pratense*	RC.37779	Tpr	NoData
OmHT18	*M. truncatula*	Medtr4g078640.1	Mtr-1	FtsJ-like methyltransferase family protein
OmHT18	*M. truncatula*	Medtr4g070380.1	Mtr-2	FtsJ-like methyltransferase family protein
OmHT18	*L. japonicus*	chr3.CM0091.1190.r2.d	Lja	
OmHT18	*P. vulgaris*	Phvul.011G044300.1	Pvu	FtsJ-like methyltransferase family protein
OmHT18	*G. max*	Glyma.12G042600.1	Gma-1	FtsJ-like methyltransferase family protein
OmHT18	*G. max*	Glyma.11G116800.1	Gma-2	FtsJ-like methyltransferase family protein
OmHT18	*A. hypogaea*	Contig7150	Ahy	NoData
OmHT19	*T. pratense*	RC.32963	Tpr	NoData
OmHT19	*M. truncatula*	Medtr4g098640.1	Mtr	ubiquitin family protein
OmHT19	*L. japonicus*	chr4.LjT10K15.20.r2.m	Lja	
OmHT19	*P. vulgaris*	Phvul.003G229300.1	Pvu	ubiquitin family protein
OmHT19	*G. max*	Glyma.05G113600.1	Gma-1	ubiquitin family protein
OmHT19	*G. max*	Glyma.17G153700.1	Gma-2	ubiquitin family protein
OmHT19	*A. hypogaea*	Contig10042	Ahy	NoData
OmHT19	*L. angstifolius*	comp8172_c0_seq1	Lan	NoData
OmHT20	*T. pratense*	RC.30812	Tpr	NoData
OmHT20	*M. truncatula*	Medtr7g096260.2	Mtr-1	nucleotide binding;nucleic acid binding;RNA binding
OmHT20	*M. truncatula*	Medtr7g094660.1	Mtr-2	nucleotide binding;nucleic acid binding;RNA binding
OmHT20	*L. japonicus*	chr1.LjT23J20.40.r2.m	Lja-1	
OmHT20	*L. japonicus*	chr1.LjT23J20.20.r2.m	Lja-2	
OmHT20	*P. vulgaris*	Phvul.001G070900.1	Pvu	nucleotide binding;nucleic acid binding;RNA binding
OmHT20	*G. max*	Glyma.14G154500.1	Gma-1	nucleotide binding;nucleic acid binding;RNA binding
OmHT20	*G. max*	Glyma.13G032300.1	Gma-2	nucleotide binding;nucleic acid binding;RNA binding
OmHT20	*A. hypogaea*	Contig1189	Ahy	NoData
OmHT20	*L. angstifolius*	comp8325_c0_seq1	Lan	NoData
OmHT20	*C. gigantea*	Cgm39412_c0.2	Cgi	NoData
OmHT21	*T. pratense*	RC.20694	Tpr	NoData
OmHT21	*M. truncatula*	Medtr8g099865.1	Mtr	fucosyltransferase 1
OmHT21	*L. japonicus*	chr3.LjT45M09.140.r2.d	Lja	
OmHT21	*P. vulgaris*	Phvul.005G182600.1	Pvu	fucosyltransferase 1
OmHT21	*G. max*	Glyma.13G370500.1	Gma-1	fucosyltransferase 1
OmHT21	*G. max*	Glyma.13G370400.1	Gma-2	fucosyltransferase 2
OmHT21	*A. hypogaea*	Contig40086	Ahy	NoData
OmHT21	*C. gigantea*	Cgm38483_c0.1	Cgi	NoData
OmHT22	*T. pratense*	RC.30446	Tpr	NoData
OmHT22	*M. truncatula*	Medtr3g460700.1	Mtr	S-adenosyl-L-methionine-dependent methyltransferases super
OmHT22	*L. japonicus*	chr6.LjT111F18.110.r2.m	Lja	
OmHT22	*P. vulgaris*	Phvul.006G059200.1	Pvu	S-adenosyl-L-methionine-dependent methyltransferases super
OmHT22	*G. max*	Glyma.18G075000.1	Gma-1	S-adenosyl-L-methionine-dependent methyltransferases super
OmHT22	*G. max*	Glyma.08G332000.1	Gma-2	S-adenosyl-L-methionine-dependent methyltransferases super
OmHT22	*A. hypogaea*	Contig2643	Ahy	NoData
OmHT22	*L. angstifolius*	comp2631_c0_seq1	Lan	NoData
OmHT22	*C. gigantea*	Cgm35057_c0.1	Cgi	NoData
AiHT01	*P. virgatum*	Pavir.J11036.1	Pvi-1	
AiHT01	*P. virgatum*	Pavir.J01022.1	Pvi-2	
AiHT01	*S. italica*	Si036782m	Sit	
AiHT01	*S. bicolor*	Sobic.001G053000.1	Sbi	
AiHT02	*B. distachyon*	Bradi1g12540.1	Bdi-1	ankyrin repeat family protein
AiHT02	*B. distachyon*	Bradi1g68102.1	Bdi-2	ankyrin repeat family protein
AiHT02	*O. sativa*	LOC_Os03g47650.1	Osa	ankyrin repeat family protein
AiHT02	*P. virgatum*	Pavir.Ib04221.1	Pvi	ankyrin repeat family protein
AiHT02	*S. bicolor*	Sobic.001G137400.1	Sbi-1	ankyrin repeat family protein

AiHT02	*S. bicolor*	Sobic.001G137500.1	Sbi-2	ankyrin repeat family protein
AiHT02	*S. bicolor*	Sobic.001G137600.1	Sbi-3	ankyrin repeat family protein
AiHT03	*B. distachyon*	Bradi3g26900.1	Bdi	NAD(P)-linked oxidoreductase superfamily protein
AiHT03	*O. sativa*	LOC_Os10g28320.1	Osa	NAD(P)-linked oxidoreductase superfamily protein
AiHT03	*P. virgatum*	Pavir.Ib03389.3	Pvi	NAD(P)-linked oxidoreductase superfamily protein
AiHT03	*S. italica*	Si036472m	Sit	NAD(P)-linked oxidoreductase superfamily protein
AiHT03	*Z. mays*	GRMZM5G813143_T01	Zma	NAD(P)-linked oxidoreductase superfamily protein
AiHT03	*S. bicolor*	Sobic.001G242800.1	Sbi	NAD(P)-linked oxidoreductase superfamily protein
AiHT04	*P. virgatum*	Pavir.J11055.1	Pvi-1	
AiHT04	*P. virgatum*	Pavir.Ib01780.1	Pvi-2	
AiHT04	*S. italica*	Si035364m	Sit	
AiHT04	*Z. mays*	GRMZM2G063498_T01	Zma-1	
AiHT04	*Z. mays*	GRMZM2G159956_T01	Zma-2	
AiHT04	*S. bicolor*	Sobic.001G358600.1	Sbi	
AiHT05	*B. distachyon*	Bradi1g69670.1	Bdi	tubulin alpha-5
AiHT05	*P. virgatum*	Pavir.J19441.1	Pvi-1	tubulin alpha-5
AiHT05	*P. virgatum*	Pavir.J34802.1	Pvi-2	tubulin alpha-5
AiHT05	*S. italica*	Si035680m	Sit	tubulin alpha-5
AiHT05	*Z. mays*	GRMZM2G051782_T01	Zma	tubulin alpha-5
AiHT05	*S. bicolor*	Sobic.001G453700.1	Sbi	tubulin alpha-5
AiHT06	*B. distachyon*	Bradi1g74922.1	Bdi	ATPase, AAA-type, CDC48 protein
AiHT06	*Z. mays*	GRMZM2G063060_T01	Zma-1	ATPase, AAA-type, CDC48 protein
AiHT06	*Z. mays*	GRMZM2G703490_T01	Zma-2	ATPase, AAA-type, CDC48 protein
AiHT06	*S. bicolor*	Sobic.001G501300.1	Sbi	ATPase, AAA-type, CDC48 protein
AiHT07	*B. distachyon*	Bradi1g28530.3	Bdi	Regulator of chromosome condensation (RCC1) family protein
AiHT07	*O. sativa*	LOC_Os07g27490.1	Osa	Regulator of chromosome condensation (RCC1) family protein
AiHT07	*P. virgatum*	Pavir.J39002.1	Pvi	Regulator of chromosome condensation (RCC1) family protein
AiHT07	*S. italica*	Si029674m	Sit	Regulator of chromosome condensation (RCC1) family protein
AiHT07	*Z. mays*	GRMZM2G095562_T01	Zma-1	Regulator of chromosome condensation (RCC1) family protein
AiHT07	*Z. mays*	GRMZM2G064873_T01	Zma-2	Regulator of chromosome condensation (RCC1) family protein
AiHT07	*S. bicolor*	Sobic.002G128200.1	Sbi	Regulator of chromosome condensation (RCC1) family protein
AiHT08	*O. sativa*	LOC_Os07g36830.1	Osa	
AiHT08	*P. virgatum*	Pavir.Ba00786.1	Pvi-1	
AiHT08	*P. virgatum*	Pavir.J35132.1	Pvi-2	
AiHT08	*P. virgatum*	Pavir.Bb03036.1	Pvi-3	
AiHT08	*Z. mays*	GRMZM2G008528_T01	Zma	
AiHT08	*S. bicolor*	Sobic.002G335400.1	Sbi	
AiHT09	*B. distachyon*	Bradi1g24170.2	Bdi	Putative methyltransferase family protein
AiHT09	*P. virgatum*	Pavir.Bb03116.1	Pvi-1	Putative methyltransferase family protein
AiHT09	*P. virgatum*	Pavir.J07808.1	Pvi-2	Putative methyltransferase family protein
AiHT09	*S. italica*	Si030798m	Sit	Putative methyltransferase family protein
AiHT09	*Z. mays*	GRMZM2G081888_T01	Zma	Putative methyltransferase family protein
AiHT09	*S. bicolor*	Sobic.002G345000.1	Sbi	Putative methyltransferase family protein
AiHT10	*B. distachyon*	Bradi2g50850.1	Bdi	Acetamidase/Formamidase family protein
AiHT10	*O. sativa*	LOC_Os01g55950.1	Osa	Acetamidase/Formamidase family protein
AiHT10	*P. virgatum*	Pavir.Ea02809.1	Pvi-1	Acetamidase/Formamidase family protein
AiHT10	*P. virgatum*	Pavir.Eb03260.1	Pvi-2	Acetamidase/Formamidase family protein
AiHT10	*P. virgatum*	Pavir.J37309.1	Pvi-3	Acetamidase/Formamidase family protein
AiHT10	*S. italica*	Si001397m	Sit	Acetamidase/Formamidase family protein
AiHT10	*Z. mays*	GRMZM2G424857_T01	Zma-1	Acetamidase/Formamidase family protein
AiHT10	*Z. mays*	GRMZM2G089944_T01	Zma-2*	Acetamidase/Formamidase family protein
AiHT10	*S. bicolor*	Sobic.003G306700.1	Sbi-1	Acetamidase/Formamidase family protein
AiHT10	*S. bicolor*	Sobic.003G306600.1	Sbi-2*	Acetamidase/Formamidase family protein
AiHT11	*P. virgatum*	Pavir.Ea04104.1	Pvi-1	SNARE-like superfamily protein
AiHT11	*P. virgatum*	Pavir.Eb04007.1	Pvi-2	SNARE-like superfamily protein
AiHT11	*Z. mays*	GRMZM2G168519_T02	Zma	SNARE-like superfamily protein
AiHT11	*S. bicolor*	Sobic.003G438200.1	Sbi	SNARE-like superfamily protein
AiHT12	*B. distachyon*	Bradi3g47270.1	Bdi	phosphoinositide binding
AiHT12	*P. virgatum*	Pavir.Aa01524.1	Pvi-1	phosphoinositide binding
AiHT12	*P. virgatum*	Pavir.Ab01979.1	Pvi-2	phosphoinositide binding
AiHT12	*S. italica*	Si016118m	Sit	phosphoinositide binding
AiHT12	*Z. mays*	GRMZM2G101523_T02	Zma	phosphoinositide binding
AiHT12	*S. bicolor*	Sobic.003G438400.1	Sbi-1	phosphoinositide binding
AiHT12	*S. bicolor*	Sobic.004G196700.1	Sbi-2	phosphoinositide binding
AiHT13	*P. virgatum*	Pavir.Aa00431.1	Pvi-1	Rubisco methyltransferase family protein
AiHT13	*P. virgatum*	Pavir.Ab02897.1	Pvi-2	Rubisco methyltransferase family protein
AiHT13	*S. italica*	Si017054m	Sit	Rubisco methyltransferase family protein
AiHT13	*Z. mays*	GRMZM2G063316_T01	Zma	Rubisco methyltransferase family protein
AiHT13	*S. bicolor*	Sobic.004G247800.1	Sbi	Rubisco methyltransferase family protein
AiHT14	*B. distachyon*	Bradi3g57387.1	Bdi	P-loop containing nucleoside triphosphate hydrolases superfar
AiHT14	*O. sativa*	LOC_Os02g53520.1	Osa	P-loop containing nucleoside triphosphate hydrolases superfar
AiHT14	*P. virgatum*	Pavir.Aa00394.1	Pvi	P-loop containing nucleoside triphosphate hydrolases superfar
AiHT14	*S. italica*	Si016200m	Sit	P-loop containing nucleoside triphosphate hydrolases superfar
AiHT14	*Z. mays*	GRMZM2G385925_T01	Zma	P-loop containing nucleoside triphosphate hydrolases superfar
AiHT14	*S. bicolor*	Sobic.004G315700.1	Sbi	P-loop containing nucleoside triphosphate hydrolases superfar
AiHT15	*B. distachyon*	Bradi3g54847.1	Bdi	endoribonucleases
AiHT15	*O. sativa*	LOC_Os02g56565.1	Osa	endoribonucleases
AiHT15	*P. virgatum*	Pavir.Aa00141.1	Pvi-1	endoribonucleases
AiHT15	*P. virgatum*	Pavir.Ab03257.1	Pvi-2	endoribonucleases
AiHT15	*S. italica*	Si016449m	Sit	endoribonucleases
AiHT15	*Z. mays*	GRMZM2G150912_T01	Zma	endoribonucleases
AiHT15	*S. bicolor*	Sobic.004G339100.1	Sbi	endoribonucleases
AiHT16	*P. virgatum*	Pavir.Ha00529.1	Pvi-1	Major facilitator superfamily protein
AiHT16	*P. virgatum*	Pavir.Hb01136.1	Pvi-2	Major facilitator superfamily protein
AiHT16	*S. italica*	Si026160m	Sit	Major facilitator superfamily protein
AiHT16	*Z. mays*	GRMZM2G034389_T01	Zma	peptide transporter 2
AiHT16	*S. bicolor*	Sobic.005G107500.1	Sbi	nitrate transporter 1:2
AiHT17	*P. virgatum*	Pavir.Ha00750.1	Pvi-1	DNA binding;ATP binding
AiHT17	*P. virgatum*	Pavir.Hb00873.1	Pvi-2	DNA binding;ATP binding
AiHT17	*S. italica*	Si027633m	Sit-1	DNA binding;ATP binding
AiHT17	*S. italica*	Si027595m	Sit-2	DNA binding;ATP binding
AiHT17	*S. bicolor*	Sobic.005G123900.1	Sbi	DNA binding;ATP binding
AiHT18	*O. sativa*	LOC_Os04g08350.2	Osa	cysteine synthase C1
AiHT18	*P. virgatum*	Pavir.J33604.1	Pvi-1	cysteine synthase C1
AiHT18	*P. virgatum*	Pavir.J08717.1	Pvi-2	cysteine synthase C1
AiHT18	*S. italica*	Si010368m	Sit	cysteine synthase C1
AiHT18	*S. bicolor*	Sobic.006G016900.1	Sbi	cysteine synthase C1

AiHT19	*P. virgatum*	Pavir.Ga00840.1	Pvi-1	GRAM domain-containing protein / ABA-responsive protein-rela
AiHT19	*P. virgatum*	Pavir.Gb00697.1	Pvi-2	GRAM domain-containing protein / ABA-responsive protein-rela
AiHT19	*S. italica*	Si011043m	Sit	GRAM domain-containing protein / ABA-responsive protein-rela
AiHT19	*Z. mays*	GRMZM2G114153_T01	Zma	GRAM domain-containing protein / ABA-responsive protein-rela
AiHT19	*S. bicolor*	Sobic.006G154500.1	Sbi	GRAM domain-containing protein / ABA-responsive protein-rela
AiHT20	*B. distachyon*	Bradi5g23130.3	Bdi-1	diacylglycerol kinase 5
AiHT20	*B. distachyon*	Bradi3g15500.1	Bdi-2*	diacylglycerol kinase 5
AiHT20	*P. virgatum*	Pavir.J36841.1	Pvi-1	diacylglycerol kinase 5
AiHT20	*P. virgatum*	Pavir.Ga00370.1	Pvi-2	diacylglycerol kinase 5
AiHT20	*P. virgatum*	Pavir.J11311.1	Pvi-3*	diacylglycerol kinase 5
AiHT20	*S. italica*	Si009926m	Sit-1	diacylglycerol kinase 5
AiHT20	*S. italica*	Si013477m	Sit-2*	diacylglycerol kinase 5
AiHT20	*Z. mays*	GRMZM2G106578_T01	Zma-1	diacylglycerol kinase 5
AiHT20	*S. bicolor*	Sobic.006G230400.1	Sbi	diacylglycerol kinase 5
AiHT21	*O. sativa*	LOC_Os09g33450.1	Osa	DHHC-type zinc finger family protein
AiHT21	*P. virgatum*	Pavir.Fa02137.1	Pvi-1	DHHC-type zinc finger family protein
AiHT21	*P. virgatum*	Pavir.Fb00090.1	Pvi-2	DHHC-type zinc finger family protein
AiHT21	*S. italica*	Si013846m	Sit	DHHC-type zinc finger family protein
AiHT21	*Z. mays*	GRMZM2G163717_T03	Zma	DHHC-type zinc finger family protein
AiHT21	*S. bicolor*	Sobic.007G002600.1	Sbi	DHHC-type zinc finger family protein
AiHT22	*P. virgatum*	Pavir.Ia00629.1	Pvi-1	ribosomal protein S10
AiHT22	*P. virgatum*	Pavir.Ib03191.1	Pvi-2	ribosomal protein S10
AiHT22	*Z. mays*	GRMZM2G095511_T01	Zma-1	ribosomal protein S10
AiHT22	*Z. mays*	GRMZM6G870163_T01	Zma-2	ribosomal protein S10
AiHT22	*S. bicolor*	Sobic.007G019100.1	Sbi	ribosomal protein S10
AiHT23	*B. distachyon*	Bradi3g16160.1	Bdi	Amino acid permease family protein
AiHT23	*O. sativa*	LOC_Os03g37984.2	Osa	Amino acid permease family protein
AiHT23	*P. virgatum*	Pavir.Fa01869.1	Pvi	Amino acid permease family protein
AiHT23	*S. italica*	Si013633m	Sit	Amino acid permease family protein
AiHT23	*Z. mays*	GRMZM2G078292_T01	Zma	Amino acid permease family protein
AiHT23	*S. bicolor*	Sobic.007G053100.1	Sbi	Amino acid permease family protein
AiHT24	*B. distachyon*	Bradi3g34780.1	Bdi	
AiHT24	*P. virgatum*	Pavir.Fa01563.1	Pvi-1	
AiHT24	*P. virgatum*	Pavir.Fb01150.1	Pvi-2	
AiHT24	*S. italica*	Si013766m	Sit	
AiHT24	*Z. mays*	GRMZM2G167694_T01	Zma	
AiHT24	*S. bicolor*	Sobic.007G119400.1	Sbi	
AiHT25	*B. distachyon*	Bradi4g40670.1	Bdi	thiamin biosynthesis protein, putative
AiHT25	*O. sativa*	LOC_Os12g09000.1	Osa	thiamin biosynthesis protein, putative
AiHT25	*P. virgatum*	Pavir.Ca00302.1	Pvi-1	thiamin biosynthesis protein, putative
AiHT25	*P. virgatum*	Pavir.Cb01947.1	Pvi-2	thiamin biosynthesis protein, putative
AiHT25	*S. italica*	Si009759m	Sit	thiamin biosynthesis protein, putative
AiHT25	*Z. mays*	GRMZM2G401934_T03	Zma	thiamin biosynthesis protein, putative
AiHT25	*S. bicolor*	Sobic.008G067100.1	Sbi	thiamin biosynthesis protein, putative
AiHT26	*B. distachyon*	Bradi2g39840.1	Bdi	Vacuolar sorting protein 39
AiHT26	*O. sativa*	LOC_Os05g01360.1	Osa	Vacuolar sorting protein 39
AiHT26	*P. virgatum*	Pavir.J01171.2	Pvi-1	Vacuolar sorting protein 39
AiHT26	*P. virgatum*	Pavir.J40078.1	Pvi-2	Vacuolar sorting protein 39
AiHT26	*S. italica*	Si021122m	Sit	Vacuolar sorting protein 39
AiHT26	*S. bicolor*	Sobic.009G002300.1	Sbi	Vacuolar sorting protein 39
AiHT27	*P. virgatum*	Pavir.Ab01636.1	Pvi	RNI-like superfamily protein
AiHT27	*S. italica*	Si017739m	Sit	RNI-like superfamily protein
AiHT27	*Z. mays*	GRMZM2G002427_T01	Zma	RNI-like superfamily protein
AiHT27	*S. bicolor*	Sobic.009G034400.1	Sbi	RNI-like superfamily protein
AiHT28	*P. virgatum*	Pavir.Aa03584.1	Pvi-1	Alg9-like mannosyltransferase family
AiHT28	*P. virgatum*	Pavir.Ab00031.1	Pvi-2	Alg9-like mannosyltransferase family
AiHT28	*S. italica*	Si016829m	Sit	Alg9-like mannosyltransferase family
AiHT28	*Z. mays*	GRMZM2G164175_T02	Zma-1	Alg9-like mannosyltransferase family
AiHT28	*Z. mays*	GRMZM2G000937_T01	Zma-2	Alg9-like mannosyltransferase family
AiHT28	*S. bicolor*	Sobic.009G194000.1	Sbi	Alg9-like mannosyltransferase family
AiHT29	*Z. mays*	GRMZM2G135044_T01	Zma	Nodulin MtN21 /EamA-like transporter family protein
AiHT29	*S. bicolor*	Sobic.010G004200.1	Sbi	Nodulin MtN21 /EamA-like transporter family protein
AiHT30	*P. virgatum*	Pavir.Aa02139.1	Pvi-1	
AiHT30	*P. virgatum*	Pavir.J35971.1	Pvi-2*	
AiHT30	*P. virgatum*	Pavir.Ea01291.1	Pvi-3*	
AiHT30	*S. italica*	Si017662m	Sit	
AiHT30	*Z. mays*	GRMZM2G000713_T01	Zma	
AiHT30	*S. bicolor*	Sobic.010G062000.1	Sbi-1	
AiHT30	*S. bicolor*	Sobic.010G062100.1	Sbi-2	
AiHT30	*S. bicolor*	Sobic.004G323900.1	Sbi-3	stress-inducible protein, putative
AiHT31	*P. virgatum*	Pavir.Da01336.1	Pvi	kinesin like protein for actin based chloroplast movement 2
AiHT31	*S. italica*	Si005702m	Sit	kinesin like protein for actin based chloroplast movement 1
AiHT31	*Z. mays*	GRMZM2G017257_T01	Zma	kinesin like protein for actin based chloroplast movement 1
AiHT31	*S. bicolor*	Sobic.010G085100.1	Sbi	kinesin like protein for actin based chloroplast movement 2
AiHT32	*Z. mays*	GRMZM2G009724_T01	Zma	BTB-POZ and MATH domain 1
AiHT32	*S. bicolor*	Sobic.010G222100.1	Sbi	BTB-POZ and MATH domain 1
AiHT33	*P. virgatum*	Pavir.Ea02508.1	Pvi	BTB-POZ and MATH domain 1
AiHT33	*Z. mays*	GRMZM2G103251_T01	Zma-1	BTB-POZ and MATH domain 2
AiHT33	*Z. mays*	GRMZM2G143782_T01	Zma-2	BTB-POZ and MATH domain 1
AiHT33	*S. bicolor*	Sobic.010G222400.1	Sbi	BTB-POZ and MATH domain 1
AiHT34	*O. sativa*	LOC_Os06g49060.1	Osa	Protein of unknown function, DUF547
AiHT34	*P. virgatum*	Pavir.Db00226.1	Pvi-1	Protein of unknown function, DUF547
AiHT34	*P. virgatum*	Pavir.J04024.1	Pvi-2	Protein of unknown function, DUF547
AiHT34	*S. italica*	Si006225m	Sit	Protein of unknown function, DUF547
AiHT34	*Z. mays*	GRMZM2G089132_T01	Zma	Protein of unknown function, DUF547
AiHT34	*S. bicolor*	Sobic.010G254700.1	Sbi	Protein of unknown function, DUF547
AiHT35	*O. sativa*	LOC_Os06g49080.1	Osa	Zinc finger C-x8-C-x5-C-x3-H type family protein
AiHT35	*P. virgatum*	Pavir.Db00225.1	Pvi-1	Zinc finger C-x8-C-x5-C-x3-H type family protein
AiHT35	*P. virgatum*	Pavir.J04026.1	Pvi-2	Zinc finger C-x8-C-x5-C-x3-H type family protein
AiHT35	*S. italica*	Si006602m	Sit	Zinc finger C-x8-C-x5-C-x3-H type family protein
AiHT35	*Z. mays*	GRMZM2G089050_T01	Zma	Zinc finger C-x8-C-x5-C-x3-H type family protein
AiHT35	*S. bicolor*	Sobic.010G254900.1	Sbi	Zinc finger C-x8-C-x5-C-x3-H type family protein
AiHT36	*O. sativa*	LOC_Os06g50930.1	Osa	Rhodanese/Cell cycle control phosphatase superfamily protein
AiHT36	*P. virgatum*	Pavir.J05059.1	Pvi-2	Rhodanese/Cell cycle control phosphatase superfamily protein
AiHT36	*P. virgatum*	Pavir.J05061.1	Pvi-1	Rhodanese/Cell cycle control phosphatase superfamily protein
AiHT36	*S. italica*	Si008168m	Sit	Rhodanese/Cell cycle control phosphatase superfamily protein
AiHT36	*Z. mays*	GRMZM2G048085_T01	Zma	Rhodanese/Cell cycle control phosphatase superfamily protein

AiHT36	*S. bicolor*	Sobic.010G270300.1	Sbi	Rhodanese/Cell cycle control phosphatase superfamily protein
AiHT37	*O. sativa*	LOC_Os06g50940.1	Osa	GDSL-like Lipase/Acylhydrolase superfamily protein
AiHT37	*P. virgatum*	Pavir.Db00017.1	Pvi	GDSL-like Lipase/Acylhydrolase superfamily protein
AiHT37	*S. italica*	Si008290m	Sit*	GDSL-like Lipase/Acylhydrolase superfamily protein
AiHT37	*Z. mays*	GRMZM2G048458_T01	Zma-1	GDSL-like Lipase/Acylhydrolase superfamily protein
AiHT37	*Z. mays*	GRMZM2G048073_T01	Zma-2	GDSL-like Lipase/Acylhydrolase superfamily protein
AiHT37	*Z. mays*	GRMZM2G022402_T01	Zma-3*	GDSL-like Lipase/Acylhydrolase superfamily protein
AiHT37	*Z. mays*	GRMZM2G132331_T01	Zma-4*	GDSL-like Lipase/Acylhydrolase superfamily protein
AiHT37	*S. bicolor*	Sobic.010G270700.1	Sbi-1	GDSL-like Lipase/Acylhydrolase superfamily protein
AiHT37	*S. bicolor*	Sobic.003G205900.1	Sbi-2*	GDSL-like Lipase/Acylhydrolase superfamily protein
AiHT37	*S. bicolor*	Sobic.010G270900.1	Sbi-3*	GDSL-like Lipase/Acylhydrolase superfamily protein
AiHT38	*O. sativa*	LOC_Os06g51010.1	Osa	Arabidopsis protein of unknown function (DUF241)
AiHT38	*P. virgatum*	Pavir.Db00024.1	Pvi	Arabidopsis protein of unknown function (DUF241)
AiHT38	*S. italica*	Si007045m	Sit	Arabidopsis protein of unknown function (DUF241)
AiHT38	*S. bicolor*	Sobic.010G271400.1	Sbi	Arabidopsis protein of unknown function (DUF241)
AiHT39	*O. sativa*	LOC_Os06g51070.1	Osa	NAC domain containing protein 36
AiHT39	*P. virgatum*	Pavir.J01198.1	Pvi-1	NAC domain containing protein 36
AiHT39	*P. virgatum*	Pavir.Db00028.1	Pvi-2	NAC domain containing protein 36
AiHT39	*S. italica*	Si006994m	Sit	NAC domain containing protein 36
AiHT39	*Z. mays*	GRMZM2G074358_T01	Zma	NAC domain containing protein 36
AiHT39	*S. bicolor*	Sobic.010G273700.1	Sbi	NAC domain containing protein 36
AiHT40	*B. distachyon*	Bradi1g29850.1	Bdi	starch branching enzyme 2.2
AiHT40	*O. sativa*	LOC_Os06g51084.1	Osa	starch branching enzyme 2.2
AiHT40	*P. virgatum*	Pavir.J01199.1	Pvi-1	starch branching enzyme 2.2
AiHT40	*P. virgatum*	Pavir.Db00029.1	Pvi-2	starch branching enzyme 2.2
AiHT40	*S. italica*	Si005911m	Sit	starch branching enzyme 2.2
AiHT40	*Z. mays*	GRMZM2G088753_T01	Zma	starch branching enzyme 2.2
AiHT40	*S. bicolor*	Sobic.010G273800.1	Sbi	starch branching enzyme 2.2
AiHT41	*B. distachyon*	Bradi1g29840.2	Bdi	Protein of unknown function (DUF300)
AiHT41	*O. sativa*	LOC_Os06g51100.1	Osa	Protein of unknown function (DUF300)
AiHT41	*P. virgatum*	Pavir.J01200.1	Pvi-1	Protein of unknown function (DUF300)
AiHT41	*P. virgatum*	Pavir.Db00030.1	Pvi-2	Protein of unknown function (DUF300)
AiHT41	*S. italica*	Si006336m	Sit	Protein of unknown function (DUF300)
AiHT41	*Z. mays*	GRMZM2G088737_T01	Zma-1	Protein of unknown function (DUF300)
AiHT41	*Z. mays*	GRMZM2G373717_T01	Zma-2	Protein of unknown function (DUF300)
AiHT41	*S. bicolor*	Sobic.010G273900.1	Sbi	Protein of unknown function (DUF300)
AiHT42	*P. virgatum*	Pavir.Db00031.5	Pvi	CYCLIN B2;4
AiHT42	*S. italica*	Si006509m	Sit	Cyclin B2;3
AiHT42	*Z. mays*	GRMZM2G073671_T01	Zma	Cyclin B2;3
AiHT42	*S. bicolor*	Sobic.010G274000.1	Sbi	Cyclin B2;3
AiHT43	*B. distachyon*	Bradi1g29800.2	Bdi	catalase 2
AiHT43	*O. sativa*	LOC_Os06g51150.1	Osa	catalase 2
AiHT43	*P. virgatum*	Pavir.Db00032.1	Pvi-1	catalase 2
AiHT43	*P. virgatum*	Pavir.J03636.1	Pvi-2	catalase 2
AiHT43	*S. italica*	Si006189m	Sit	catalase 2
AiHT43	*Z. mays*	GRMZM2G088212_T01	Zma	catalase 2
AiHT43	*S. bicolor*	Sobic.010G274500.1	Sbi	catalase 2
AiHT44	*B. distachyon*	Bradi1g04226.2	Bdi-1	Transducin/WD40 repeat-like superfamily protein
AiHT44	*B. distachyon*	Bradi1g14400.1	Bdi-2	Transducin/WD40 repeat-like superfamily protein
AiHT44	*O. sativa*	LOC_Os01g08770.1	Osa	Transducin/WD40 repeat-like superfamily protein
AiHT44	*P. virgatum*	Pavir.Ba02231.1	Pvi-1	Transducin/WD40 repeat-like superfamily protein
AiHT44	*P. virgatum*	Pavir.Bb02004.1	Pvi-2	Transducin/WD40 repeat-like superfamily protein
AiHT44	*S. italica*	Si000998m	Sit-1	Transducin/WD40 repeat-like superfamily protein
AiHT44	*S. italica*	Si016892m	Sit-2	Transducin/WD40 repeat-like superfamily protein
AiHT44	*Z. mays*	GRMZM2G096051_T01	Zma	Transducin/WD40 repeat-like superfamily protein
AiHT44	*S. bicolor*	Sobic.010G274600.1	Sbi-1	Transducin/WD40 repeat-like superfamily protein
AiHT44	*S. bicolor*	Sobic.004G157400.1	Sbi-2	Transducin/WD40 repeat-like superfamily protein
AiHT45	*B. distachyon*	Bradi1g29750.1	Bdi	Protein of unknown function (DUF833)
AiHT45	*O. sativa*	LOC_Os09g15480.1	Osa	Protein of unknown function (DUF833)
AiHT45	*P. virgatum*	Pavir.J17263.1	Pvi-1	Protein of unknown function (DUF833)
AiHT45	*P. virgatum*	Pavir.Db00037.1	Pvi-2	Protein of unknown function (DUF833)
AiHT45	*S. italica*	Si007094m	Sit	Protein of unknown function (DUF833)
AiHT45	*Z. mays*	GRMZM2G037284_T01	Zma	Protein of unknown function (DUF833)
AiHT45	*S. bicolor*	Sobic.010G275100.1	Sbi	Protein of unknown function (DUF833)
AiHT46	*B. distachyon*	Bradi1g29730.1	Bdi	high mobility group B2
AiHT46	*P. virgatum*	Pavir.J04920.2	Pvi-1	high mobility group B3
AiHT46	*P. virgatum*	Pavir.Db00040.1	Pvi-2	high mobility group B3
AiHT46	*S. italica*	Si007288m	Sit	high mobility group B2
AiHT46	*Z. mays*	GRMZM5G834758_T02	Zma	high mobility group B2
AiHT46	*S. bicolor*	Sobic.010G275200.1	Sbi	high mobility group B3
AiHT47	*P. virgatum*	Pavir.Db00317.1	Pvi-1	titan9
AiHT47	*P. virgatum*	Pavir.Ea01114.1	Pvi-2	titan9
AiHT47	*S. italica*	Si002590m	Sit	titan9
AiHT47	*Z. mays*	GRMZM2G007258_T01	Zma-1	titan9
AiHT47	*Z. mays*	GRMZM2G019810_T03	Zma-2	titan9
AiHT47	*S. bicolor*	Sobic.003G127500.1	Sbi-1	titan9
AiHT47	*S. bicolor*	Sobic.007G184100.1	Sbi-2	titan9
AiHT48	*B. distachyon*	Bradi1g51940.2	Bdi	cell division cycle 48C
AiHT48	*O. sativa*	LOC_Os06g01980.1	Osa	cell division cycle 48C
AiHT48	*P. virgatum*	Pavir.Ca00597.1	Pvi-1	cell division cycle 48C
AiHT48	*P. virgatum*	Pavir.Da02408.1	Pvi-2*	cell division cycle 48C
AiHT48	*P. virgatum*	Pavir.J00276.1	Pvi-3*	cell division cycle 48C
AiHT48	*P. virgatum*	Pavir.Da02401.1	Pvi-4*	cell division cycle 48C
AiHT48	*S. italica*	Si040216m	Sit-1	cell division cycle 48C
AiHT48	*S. italica*	Si005901m	Sit-2	cell division cycle 48C
AiHT48	*Z. mays*	GRMZM2G082131_T01	Zma-1	cell division cycle 48C
AiHT48	*Z. mays*	GRMZM2G475360_T03	Zma-2*	cell division cycle 48C
AiHT48	*S. bicolor*	Sobic.010G007100.1	Sbi*	cell division cycle 48C
AiHT49	*B. distachyon*	Bradi4g07610.1	Bdi	
AiHT49	*O. sativa*	LOC_Os12g28270.1	Osa	
AiHT49	*P. virgatum*	Pavir.Cb00776.1	Pvi	
AiHT49	*S. italica*	Si021935m	Sit	
AiHT49	*Z. mays*	GRMZM2G319465_T01	Zma	
AiHT49	*S. bicolor*	Sobic.K002400.1	Sbi	
AiHT50	*B. distachyon*	Bradi3g08480.1	Bdi	Peroxisomal membrane 22 kDa (Mpv17/PMP22) family protein
AiHT50	*P. virgatum*	Pavir.J11740.1	Pvi-1	Peroxisomal membrane 22 kDa (Mpv17/PMP22) family protein
AiHT50	*P. virgatum*	Pavir.J39363.1	Pvi-2	Peroxisomal membrane 22 kDa (Mpv17/PMP22) family protein

AiHT50	*S. italica*	Si018070m	Sit	Peroxisomal membrane 22 kDa (Mpv17/PMP22) family protein
AiHT50	*Z. mays*	GRMZM2G155220_T01	Zma	Peroxisomal membrane 22 kDa (Mpv17/PMP22) family protein
AiHT50	*S. bicolor*	Sobic.004G097200.1	Sbi	Peroxisomal membrane 22 kDa (Mpv17/PMP22) family protein
AiHT51	*B. distachyon*	Bradi1g25860.1	Bdi	thiazole biosynthetic enzyme, chloroplast (ARA6) (THI1) (THI4
AiHT51	*P. virgatum*	Pavir.Db00653.1	Pvi	thiazole biosynthetic enzyme, chloroplast (ARA6) (THI1) (THI4
AiHT51	*S. italica*	Si030346m	Sit	thiazole biosynthetic enzyme, chloroplast (ARA6) (THI1) (THI4
AiHT51	*Z. mays*	GRMZM2G074097_T01	Zma-1	thiazole biosynthetic enzyme, chloroplast (ARA6) (THI1) (THI4
AiHT51	*Z. mays*	GRMZM2G018375_T01	Zma-2	thiazole biosynthetic enzyme, chloroplast (ARA6) (THI1) (THI4
AiHT51	*S. bicolor*	Sobic.003G191000.1	Sbi-1	thiazole biosynthetic enzyme, chloroplast (ARA6) (THI1) (THI4
AiHT51	*S. bicolor*	Sobic.002G384400.1	Sbi-2	thiazole biosynthetic enzyme, chloroplast (ARA6) (THI1) (THI4
AiHT52	*B. distachyon*	Bradi1g24040.2	Bdi	splicing factor Prp18 family protein
AiHT52	*P. virgatum*	Pavir.Bb03146.1	Pvi	splicing factor Prp18 family protein
AiHT52	*S. italica*	Si029857m	Sit	splicing factor Prp18 family protein
AiHT52	*Z. mays*	GRMZM2G046916_T01	Zma-1	splicing factor Prp18 family protein
AiHT52	*Z. mays*	GRMZM2G041724_T01	Zma-2	splicing factor Prp18 family protein
AiHT52	*Z. mays*	GRMZM2G053642_T01	Zma-3	splicing factor Prp18 family protein
AiHT52	*Z. mays*	GRMZM2G034709_T01	Zma-4	splicing factor Prp18 family protein
AiHT52	*Z. mays*	GRMZM2G034651_T01	Zma-5	
AiHT52	*Z. mays*	GRMZM2G073041_T01	Zma-6	
AiHT52	*Z. mays*	GRMZM2G053622_T01	Zma-7	
AiHT52	*S. bicolor*	Sobic.002G346900.1	Sbi	splicing factor Prp18 family protein
AiHT53	*B. distachyon*	Bradi4g37327.1	Bdi	NAC domain containing protein 57
AiHT53	*P. virgatum*	Pavir.Bb02626.1	Pvi-1	NAC domain containing protein 57
AiHT53	*P. virgatum*	Pavir.Ba01386.1	Pvi-2	NAC domain containing protein 57
AiHT53	*S. italica*	Si032702m	Sit	NAC domain containing protein 57
AiHT53	*Z. mays*	GRMZM5G885329_T01	Zma	NAC domain containing protein 57
AiHT53	*S. bicolor*	Sobic.002G290900.1	Sbi	NAC domain containing protein 57
AiHT54	*O. sativa*	LOC_Os04g58700.1	Osa	Leucine-rich repeat protein kinase family protein
AiHT54	*P. virgatum*	Pavir.Ga00525.1	Pvi-1	Leucine-rich repeat protein kinase family protein
AiHT54	*P. virgatum*	Pavir.Gb00222.1	Pvi-2	Leucine-rich repeat protein kinase family protein
AiHT54	*S. italica*	Si021400m	Sit	Leucine-rich repeat protein kinase family protein
AiHT54	*Z. mays*	GRMZM2G119759_T01	Zma	Leucine-rich repeat protein kinase family protein
AiHT54	*S. bicolor*	Sobic.006G272500.1	Sbi	Leucine-rich repeat protein kinase family protein
AiHT55	*B. distachyon*	Bradi2g20360.2	Bdi-1	Pseudouridine synthase family protein
AiHT55	*B. distachyon*	Bradi2g51417.1	Bdi-2	Pseudouridine synthase family protein
AiHT55	*P. virgatum*	Pavir.J05644.1	Pvi-1	Pseudouridine synthase family protein
AiHT55	*P. virgatum*	Pavir.Ca01394.1	Pvi-2	Pseudouridine synthase family protein
AiHT55	*S. italica*	Si021383m	Sit	Pseudouridine synthase family protein
AiHT55	*Z. mays*	GRMZM2G096553_T01	Zma-1	Pseudouridine synthase family protein
AiHT55	*Z. mays*	GRMZM2G173137_T02	Zma-2	Pseudouridine synthase family protein
AiHT55	*S. bicolor*	Sobic.009G191500.1	Sbi	Pseudouridine synthase family protein
AiHT56	*B. distachyon*	Bradi1g51187.1	Bdi	PRP38 family protein
AiHT56	*P. virgatum*	Pavir.Db02243.1	Pvi	PRP38 family protein
AiHT56	*S. italica*	Si006617m	Sit	PRP38 family protein
AiHT56	*Z. mays*	GRMZM2G173693_T01	Zma	PRP38 family protein
AiHT56	*S. bicolor*	Sobic.010G033400.1	Sbi	PRP38 family protein
AiHT57	*B. distachyon*	Bradi1g06630.1	Bdi	associated protein 19
AiHT57	*P. virgatum*	Pavir.J06884.1	Pvi	associated protein 19
AiHT57	*S. italica*	Si007399m	Sit	associated protein 19
AiHT57	*Z. mays*	GRMZM2G361220_T01	Zma	associated protein 19
AiHT57	*S. bicolor*	Sobic.001G067900.1	Sbi	associated protein 19
AiHT58	*B. distachyon*	Bradi3g07926.5	Bdi-1*	Coatomer, beta¥' subunit
AiHT58	*B. distachyon*	Bradi3g07918.1	Bdi-2*	Coatomer, beta¥' subunit
AiHT58	*P. virgatum*	Pavir.Da00644.1	Pvi-1	Coatomer, beta¥' subunit
AiHT58	*P. virgatum*	Pavir.Db01603.1	Pvi-2	Coatomer, beta¥' subunit
AiHT58	*P. virgatum*	Pavir.Aa02949.1	Pvi-3*	Coatomer, beta¥' subunit
AiHT58	*P. virgatum*	Pavir.Ab00758.1	Pvi-4*	Coatomer, beta¥' subunit
AiHT58	*S. italica*	Si005833m	Sit-1	Coatomer, beta¥' subunit
AiHT58	*S. italica*	Si016124m*	Sit-2*	Coatomer, beta¥' subunit
AiHT58	*Z. mays*	GRMZM2G443953_T01	Zma-1	Coatomer, beta¥' subunit
AiHT58	*Z. mays*	GRMZM2G141587_T01	Zma-2*	Coatomer, beta¥' subunit
AiHT58	*Z. mays*	GRMZM2G124886_T01	Zma-3*	Coatomer, beta¥' subunit
AiHT58	*S. bicolor*	Sobic.010G178600.1	Sbi	Coatomer, beta¥' subunit
AiHT59	*B. distachyon*	Bradi4g22180.2	Bdi	Protein kinase superfamily protein
AiHT59	*O. sativa*	LOC_Os11g11000.1	Osa	Protein kinase superfamily protein
AiHT59	*P. virgatum*	Pavir.J16568.1	Pvi-1	Protein kinase superfamily protein
AiHT59	*P. virgatum*	Pavir.Hb01186.1	Pvi-2	Protein kinase superfamily protein
AiHT59	*S. italica*	Si006000m	Sit-1	Protein kinase superfamily protein
AiHT59	*S. italica*	Si027842m	Sit-2	Protein kinase superfamily protein
AiHT59	*S. italica*	Si027711m	Sit-3	Protein kinase superfamily protein
AiHT59	*Z. mays*	GRMZM2G377115_T01	Zma	Protein kinase superfamily protein
AiHT59	*S. bicolor*	Sobic.005G101200.1	Sbi	Protein kinase superfamily protein
AiHT60	*P. virgatum*	Pavir.J11373.1	Pvi-1	F-box family protein with a domain of unknown function (DUF2
AiHT60	*P. virgatum*	Pavir.J10024.1	Pvi-2	
AiHT60	*P. virgatum*	Pavir.Da02389.1	Pvi-3	F-box family protein with a domain of unknown function (DUF2
AiHT60	*S. italica*	Si035951m	Sit	Protein of unknown function (DUF295)
AiHT61	*O. sativa*	LOC_Os03g05780.1	Osa	AMP-dependent synthetase and ligase family protein
AiHT61	*P. virgatum*	Pavir.J37841.1	Pvi-1	AMP-dependent synthetase and ligase family protein
AiHT61	*P. virgatum*	Pavir.Ia01374.1	Pvi-2	AMP-dependent synthetase and ligase family protein
AiHT61	*P. virgatum*	Pavir.Ib03807.1	Pvi-3	AMP-dependent synthetase and ligase family protein
AiHT61	*S. italica*	Si034944m	Sit-1	AMP-dependent synthetase and ligase family protein
AiHT61	*S. italica*	Si039879m	Sit-2	AMP-dependent synthetase and ligase family protein
AiHT61	*S. italica*	Si034892m	Sit-3*	AMP-dependent synthetase and ligase family protein
AiHT61	*Z. mays*	GRMZM2G174574_T02	Zma-1	AMP-dependent synthetase and ligase family protein
AiHT61	*Z. mays*	GRMZM2G122787_T02	Zma-2*	AMP-dependent synthetase and ligase family protein
AiHT61	*S. bicolor*	Sobic.001G187000.1	Sbi-1	AMP-dependent synthetase and ligase family protein
AiHT61	*S. bicolor*	Sobic.001G500800.1	Sbi-2*	AMP-dependent synthetase and ligase family protein
AiHT62	*B. distachyon*	Bradi1g15740.2	Bdi	DTW domain-containing protein
AiHT62	*O. sativa*	LOC_Os03g37100.1	Osa	DTW domain-containing protein
AiHT62	*P. virgatum*	Pavir.Ia01375.1	Pvi-1	DTW domain-containing protein
AiHT62	*P. virgatum*	Pavir.J37842.1	Pvi-2	DTW domain-containing protein
AiHT62	*S. italica*	Si035814m	Sit	DTW domain-containing protein
AiHT62	*Z. mays*	GRMZM2G090642_T01	Zma-1	DTW domain-containing protein
AiHT62	*Z. mays*	GRMZM2G352619_T01	Zma-2	DTW domain-containing protein
AiHT62	*Z. mays*	GRMZM2G380133_T01	Zma-3	DTW domain-containing protein
AiHT62	*S. bicolor*	Sobic.001G187100.1	Sbi	DTW domain-containing protein
AiHT63	*P. virgatum*	Pavir.Ib03805.1	Pvi	molybdate transporter 1

AiHT63	*S. italica*	Si035427m	Sit	molybdate transporter 1
AiHT63	*S. bicolor*	Sobic.001G187300.1	Sbi	molybdate transporter 1
AiHT64	*O. sativa*	LOC_Os07g45195.1	Osa-1	
AiHT64	*O. sativa*	LOC_Os07g45194.1	Osa-2	
AiHT64	*P. virgatum*	Pavir.J06640.1	Pvi-1	
AiHT64	*P. virgatum*	Pavir.J06144.10	Pvi-2	
AiHT64	*P. virgatum*	Pavir.Ab00102.1	Pvi-3	
AiHT64	*S. italica*	Si018136m	Sit	
AiHT64	*S. bicolor*	Sobic.001G415700.1	Sbi	
AiHT65	*P. virgatum*	Pavir.Ba02235.1	Pvi-1	O-fucosyltransferase family protein
AiHT65	*P. virgatum*	Pavir.Bb03156.1	Pvi-2	O-fucosyltransferase family protein
AiHT65	*Z. mays*	GRMZM2G030713_T01	Zma	O-fucosyltransferase family protein
AiHT65	*S. bicolor*	Sobic.002G347900.1	Sbi	O-fucosyltransferase family protein
AiHT66	*P. virgatum*	Pavir.Bb03186.1	Pvi-1	translocon at the outer envelope membrane of chloroplasts 75-
AiHT66	*P. virgatum*	Pavir.Ba00530.1	Pvi-2	translocon at the outer envelope membrane of chloroplasts 75-
AiHT66	*S. italica*	Si029048m	Sit	translocon at the outer envelope membrane of chloroplasts 75-
AiHT66	*S. bicolor*	Sobic.002G349900.1	Sbi	translocon at the outer envelope membrane of chloroplasts 75-
AiHT67	*P. virgatum*	Pavir.Bb01074.1	Pvi	wall associated kinase-like 6
AiHT67	*S. bicolor*	Sobic.002G166200.1	Sbi	wall associated kinase-like 1
AiHT68	*O. sativa*	LOC_Os04g12970.1	Osa-1	UDP-glucosyltransferase 74F2
AiHT68	*O. sativa*	LOC_Os04g12950.1	Osa-2	UDP-glucosyltransferase 74F2
AiHT68	*P. virgatum*	Pavir.Ga02622.1	Pvi-1	UDP-glucosyltransferase 74F2
AiHT68	*P. virgatum*	Pavir.J17513.1	Pvi-2	UDP-glucosyltransferase 74F2
AiHT68	*S. italica*	Si010049m	Sit	Uridine diphosphate glycosyltransferase 74E2
AiHT68	*Z. mays*	GRMZM2G063042_T01	Zma-1	UDP-glucosyltransferase 74F2
AiHT68	*Z. mays*	GRMZM2G035282_T01	Zma-2	Uridine diphosphate glycosyltransferase 74E2
AiHT68	*Z. mays*	GRMZM2G095280_T01	Zma-3	UDP-Glycosyltransferase superfamily protein
AiHT68	*Z. mays*	GRMZM2G334336_T01	Zma-4	UDP-Glycosyltransferase superfamily protein
AiHT68	*S. bicolor*	Sobic.006G024000.1	Sbi	UDP-glucosyltransferase 74F2
AiHT69	*P. virgatum*	Pavir.Hb01859.1	Pvi	ankyrin repeat family protein
AiHT69	*Z. mays*	GRMZM2G536120_T01	Zma	ankyrin repeat family protein
AiHT69	*S. bicolor*	Sobic.008G025200.1	Sbi-1	ankyrin repeat family protein
AiHT69	*S. bicolor*	Sobic.008G025400.1	Sbi-2	ankyrin repeat family protein
AiHT70	*P. virgatum*	Pavir.Da02235.1	Pvi-1	BRCT domain-containing DNA repair protein
AiHT70	*P. virgatum*	Pavir.Db02197.1	Pvi-2	BRCT domain-containing DNA repair protein
AiHT70	*S. italica*	Si006812m	Sit	BRCT domain-containing DNA repair protein
AiHT70	*S. bicolor*	Sobic.010G033600.1	Sbi	BRCT domain-containing DNA repair protein
AiHT71	*B. distachyon*	Bradi2g00417.1	Bdi	multidrug resistance-associated protein 3
AiHT71	*O. sativa*	LOC_Os02g18670.1	Osa	multidrug resistance-associated protein 3
AiHT71	*P. virgatum*	Pavir.Bb00325.1	Pvi	multidrug resistance-associated protein 3
AiHT71	*S. italica*	Si032963m	Sit-1	multidrug resistance-associated protein 3
AiHT71	*S. italica*	Si027817m	Sit-2	multidrug resistance-associated protein 3
AiHT71	*Z. mays*	GRMZM5G832772_T01	Zma	multidrug resistance-associated protein 3
AiHT72	*B. distachyon*	Bradi3g55500.1	Bdi	
AiHT72	*P. virgatum*	Pavir.Gb01821.1	Pvi	
AiHT73	*B. distachyon*	Bradi4g31220.2	Bdi	chaperonin 20
AiHT73	*P. virgatum*	Pavir.Ba01868.1	Pvi	chaperonin 20
AiHT73	*S. italica*	Si030910m	Sit	chaperonin 20
AiHT73	*S. bicolor*	Sobic.002G214600.1	Sbi	chaperonin 20
AiHT74	*B. distachyon*	Bradi2g47987.1	Bdi	Insulinase (Peptidase family M16) protein
AiHT74	*O. sativa*	LOC_Os01g51390.1	Osa	Insulinase (Peptidase family M16) protein
AiHT74	*P. virgatum*	Pavir.J40338.1	Pvi-1	Insulinase (Peptidase family M16) protein
AiHT74	*P. virgatum*	Pavir.Eb02626.1	Pvi-2	Insulinase (Peptidase family M16) protein
AiHT74	*S. italica*	Si001093m	Sit	Insulinase (Peptidase family M16) protein
AiHT74	*Z. mays*	GRMZM5G827505_T02	Zma	Insulinase (Peptidase family M16) protein
AiHT74	*S. bicolor*	Sobic.003G274800.1	Sbi	Insulinase (Peptidase family M16) protein
AiHT75	*B. distachyon*	Bradi5g08230.1	Bdi-1	2-oxoglutarate dehydrogenase, E1 component
AiHT75	*B. distachyon*	Bradi5g08235.1	Bdi-2	2-oxoglutarate dehydrogenase, E1 component
AiHT75	*P. virgatum*	Pavir.Ga01944.1	Pvi-1	2-oxoglutarate dehydrogenase, E1 component
AiHT75	*P. virgatum*	Pavir.Gb01953.1	Pvi-2	2-oxoglutarate dehydrogenase, E1 component
AiHT75	*S. italica*	Si009245m	Sit	2-oxoglutarate dehydrogenase, E1 component
AiHT75	*Z. mays*	GRMZM2G151041_T01	Zma-1	2-oxoglutarate dehydrogenase, E1 component
AiHT75	*Z. mays*	GRMZM2G142863_T01	Zma-2	2-oxoglutarate dehydrogenase, E1 component
AiHT75	*S. bicolor*	Sobic.006G052500.1	Sbi-1	2-oxoglutarate dehydrogenase, E1 component
AiHT75	*S. bicolor*	Sobic.006G052800.1	Sbi-2	2-oxoglutarate dehydrogenase, E1 component
AiHT76	*B. distachyon*	Bradi5g21658.2	Bdi	methyl-CPG-binding domain protein 13
AiHT76	*O. sativa*	LOC_Os04g52380.1	Osa	methyl-CPG-binding domain protein 13
AiHT76	*P. virgatum*	Pavir.J26297.1	Pvi	methyl-CPG-binding domain protein 13
AiHT76	*S. italica*	Si011769m	Sit	methyl-CPG-binding domain protein 13
AiHT76	*Z. mays*	GRMZM2G119802_T01	Zma	methyl-CPG-binding domain protein 13
AiHT77	*B. distachyon*	Bradi1g12550.2	Bdi	thiaminC
AiHT77	*O. sativa*	LOC_Os03g47610.1	Osa	thiaminC
AiHT77	*P. virgatum*	Pavir.J26403.1	Pvi-1	thiaminC
AiHT77	*P. virgatum*	Pavir.J27913.1	Pvi-2	thiaminC
AiHT77	*S. italica*	Si034631m	Sit	thiaminC
AiHT77	*Z. mays*	GRMZM2G027663_T01	Zma	thiaminC
AiHT77	*S. bicolor*	Sobic.001G138000.1	Sbi	thiaminC
AiHT78	*O. sativa*	LOC_Os08g40110.2	Osa	Peptidase M20/M25/M40 family protein
AiHT78	*P. virgatum*	Pavir.Fa00445.1	Pvi-1	Peptidase M20/M25/M40 family protein
AiHT78	*P. virgatum*	Pavir.Fb01931.1	Pvi-2	Peptidase M20/M25/M40 family protein
AiHT78	*S. italica*	Si013712m	Sit	Peptidase M20/M25/M40 family protein
AiHT78	*Z. mays*	GRMZM2G088627_T01	Zma	Peptidase M20/M25/M40 family protein
AiHT78	*S. bicolor*	Sobic.007G208300.1	Sbi	Peptidase M20/M25/M40 family protein
AiHT79	*B. distachyon*	Bradi3g21500.2	Bdi-1	Flavodoxin family protein
AiHT79	*B. distachyon*	Bradi2g49010.1	Bdi-2*	Flavodoxin family protein
AiHT79	*O. sativa*	LOC_Os01g53250.1	Osa*	Flavodoxin family protein
AiHT79	*P. virgatum*	Pavir.Ia03658.1	Pvi-1	Flavodoxin family protein
AiHT79	*P. virgatum*	Pavir.Ea02961.1	Pvi-2*	Flavodoxin family protein
AiHT79	*S. italica*	Si000652m	Sit*	Flavodoxin family protein
AiHT80	*B. distachyon*	Bradi5g25382.1	Bdi*	
AiHT80	*O. sativa*	LOC_Os04g40920.1	Osa*	F-box family protein with a domain of unknown function (DUF2
AiHT80	*P. virgatum*	Pavir.J01999.1	Pvi-1*	
AiHT80	*P. virgatum*	Pavir.Fb01975.1	Pvi-2*	F-box family protein with a domain of unknown function (DUF2
AiHT80	*S. italica*	Si035547m	Sit-1*	
AiHT80	*S. italica*	Si038919m	Sit-2*	F-box family protein with a domain of unknown function (DUF2
AiHT80	*S. bicolor*	Sobic.004G328500.1	Sbi-1	
AiHT80	*S. bicolor*	Sobic.007G197300.1	Sbi-2*	F-box family protein with a domain of unknown function (DUF2

AiHT81	*S. hermonthica*	ShContig9483	Striga
AiHT81	*B. distachyon*	Bradi2g36840.1	Bdi-1
AiHT81	*B. distachyon*	Bradi1g00410.1	Bdi-2
AiHT81	*O. sativa*	LOC_Os01g56900.1	Osa
AiHT81	*S. italica*	Si029853m	Sit-1
AiHT81	*S. italica*	Si029864m	Sit-2
AiHT81	*S. italica*	Si022060m	Sit-3
AiHT81	*S. bicolor*	Sobic.001G152700.1	Sbi-1
AiHT81	*S. bicolor*	Sobic.K014500.1	Sbi-2
AiHT81	*S. bicolor*	Sobic.003G368800.1	Sbi-3

*1: The abbreviations are used in Figure 2 and Figure S1.

*2: We refered to "best Athaliana TAIR10 hit defline" column of "annotaion info" file in Phytozome database.

*3: We refered to "Gene Ontology terms" column of "annotation_info" file in Phytozome database.

Table S6 Summary of HGT genes

Locus	Lineage Class	Assembly length (bp)	Transcript length (bp)	CDS length (bp)	# of Exons	# of Introns	# of correctly spliced introns *3	# of fully assembled introns *4	Preservation of CDS		CDSs (TSAs) used for Ka & Ks analysis	CDS length (bp) *5	Ka	Ks	Ka/Ks
OmHT01	*T. pratense*	3,185	465	465	3	2	2	2	Incomplete	Partial (30%, 5'+3'Lack); Nonsense;	RC.42229	336	0.004	0.065	0.061
OmHT02	*T. pratense*	4,237	538	390	3	2	2	2	Complete	-	RC.10825	327	0.049	0.198	0.248
OmHT03	*T. pratense*	1,717	1,179	757	1	0	-	-	Incomplete	Partial (70%, 5'Lack); Frameshift;	RC.29156	480	0.032	0.181	0.176
OmHT04	*T. pratense*	5,878	1,273	1,077	8	7	7	7	Complete	-	RC.34162	873	0.022	0.090	0.240
OmHT05	*T. pratense*	10,640	1,810	1,443	15	14	14	12	Complete	-	RC.32426	726	0.014	0.044	0.327
OmHT06	*T. pratense*	4,924	1,106	777	8	7	7	7	Complete	-	RC.13251	276	0.073	0.287	0.256
OmHT07	*T. pratense*	7,772	1,573	1,371	11	10	10	9	Complete	-	RC.41913	612	0.017	0.105	0.164
OmHT08	*T. pratense*	26,558	6,032	5,871	36	35	35	32	Complete	-	RC.37121	5688	0.033	0.103	0.318
OmHT09 *1	*T. pratense*	NA	1,523	1,216	2	1	NA	NA	Incomplete	Partial (15% or 50%, 5'+3'Lack); Frameshift; Indel	RC.41584	693	0.021	0.097	0.214
OmHT10	*T. pratense*	7,075	1,534	1,260	11	10	10	7	Complete	-	RC.42261	888	0.067	0.071	0.950
OmHT11	*T. pratense*	5,553	1,804	1,397	11	10	10	10	Incomplete	Frameshift; Nonsense; Indel(120bp)	RC.6350	1386	0.071	0.315	0.227
OmHT12	*T. pratense and/or M. truncatula*	2,671	314	314	3	2	2	2	Incomplete	Partial (20%, 5'+3'Lack)	RC.37978	273	0.038	0.037	1.005
OmHT13	*T. pratense and/or M. truncatula*	9,803	1,313	951	7	6	6	4	Complete	-	Medtr8g075480.1	474	0.036	0.140	0.259
OmHT14	*T. pratense and/or M. truncatula*	2,892	990	990	3	2	2	2	Complete	-	Medtr7g074730.1	648	0.097	0.332	0.293
OmHT15	*T. pratense and/or M. truncatula*	4,691	2,346	2,063	7	6	5*	6	Incomplete	Frameshift; Indel(120bp)	Medtr1g086640.1	1110	0.044	0.320	0.139
OmHT16	*T. pratense and/or M. truncatula*	7,973	1,908	1,257	10	9	9	9	Complete	-	Medtr5g064160.1	783	0.121	0.181	0.665
OmHT17	*T. pratense and M. truncatula*	9,866	1,659	1,659	10	9	9	8	Complete	-	RC.31431	726	0.024	0.281	0.084
OmHT18	*T. pratense and M. truncatula*	6,946	2,897	2,463	11	10	10	9	Complete	-	RC.37779	1806	0.099	0.572	0.172
OmHT19 *1	*L. japonicus*	NA	1,082	672	7	6	NA	NA	Complete	-	chr4.LjT10K15.20.r2.m	357	0.019	0.212	0.088
OmHT20	*L. japonicus*	11,545	3,119	2,550	15	14	14	13	Complete	-	chr1.LjT23J20.40.r2.m	1167	0.084	0.130	0.646
OmHT21	*P. vulgaris* and/or *G. max*	3,098	2,066	1,652	2	1	1	1	Incomplete	Partial (90%, 5'Lack)	Glyma.13G370400.1	1149	0.112	0.307	0.364
OmHT22 *1	*L. angstifolius*	NA	2,473	1,965	17	16	NA	NA	Complete	-	comp2631_c0_seq1	822	0.016	0.172	0.094
AiHT01 *1	*S. bicolor* and/ or *Z. mays*	NA	621	621	1	0	NA	NA	Incomplete	Partial (35%, 5'Lack); Nonsense;	Sobic.001G053000.1	138	0.068	0.188	0.362
AiHT02-1	*S. bicolor* and/ or *Z. mays*	8,851	2,414	1,491	11	10	10	6	Incomplete	Frameshift;	Sobic.001G137400.1	389	0.054	0.108	0.496
AiHT02-2	*S. bicolor* and/ or *Z. mays*	6,953	1,514	1,149	10	9	9	8	Complete	-	Sobic.001G137500.1	389	0.096	0.128	0.748
AiHT02-3	*S. bicolor* and/ or *Z. mays*	7,971	1,308	1,149	10	9	9	8	Complete	-	Sobic.001G137500.1	389	0.078	0.129	0.604
AiHT03	*S. bicolor* and/ or *Z. mays*	3,955	1,426	1,026	7	6	6	5	Complete	-	Sobic.001G242800.1	1,023	0.022	0.089	0.252
AiHT04 *1	*S. bicolor* and/ or *Z. mays*	NA	1,467	1,425	4	3	NA	NA	Nearly complete	Partial (90%, 5'Lack);	GRMZM2G063498_T01	990	0.062	0.130	0.476
AiHT05	*S. bicolor* and/ or *Z. mays*	3,979	1,100	1,100	5	4	4	4	Incomplete	Partial (80%, 3'Lack);	Sobic.001G453700.1	750	0.004	0.141	0.025
AiHT06	*S. bicolor* and/ or *Z. mays*	2,892	961	427	3	2	2	2	Incomplete	Partial (15%, 3'Lack);	GRMZM2G703490_T01	330	0.051	0.249	0.206
AiHT07	*S. bicolor* and/ or *Z. mays*	3,849	1,696	1,275	6	5	5	5	Nearly complete	Partial (90%, 3'Lack);	Sobic.002G128200.1	795	0.007	0.066	0.103
AiHT08	*S. bicolor* and/ or *Z. mays*	1,899	1,593	1,215	1	0	-	-	Complete	-	Sobic.002G335400.1	495	0.082	0.200	0.408
AiHT09	*S. bicolor* and/ or *Z. mays*	1,411	662	397	1	0	-	-	Incomplete	Partial (50%, 5'Lack);	Sobic.002G345000.1	396	0.053	0.164	0.324
AiHT10 *1	*S. bicolor* and/ or *Z. mays*	NA	1,837	1,368	7	6	NA	NA	Complete	-	Sobic.003G306700.1	1,236	0.014	0.111	0.130
AiHT11	*S. bicolor* and/ or *Z. mays*	6,917	1,544	621	6	5	4*	5	Complete	-	Sobic.003G438200.1	399	0.020	0.085	0.231
AiHT12	*S. bicolor* and/ or *Z. mays*	3,921	1,686	1,450	3	2	2	2	Incomplete	Partial (45%, 3'Lack); Frameshift; Indel(100bp);	Sobic.003G438400.1	909	0.021	0.092	0.231
AiHT13	*S. bicolor* and/ or *Z. mays*	2,238	1,955	461	2	1	1	1	Incomplete	Partial (30%, 5'Lack); Frameshift;	Sobic.004G247800.1	453	0.029	0.022	1.343
AiHT14	*S. bicolor* and/ or *Z. mays*	12,497	2,868	2,736	22	21	21	21	Incomplete	Partial (90%, 5'Lack); Frameshift; Nonsense;	Sobic.004G315700.1	1,680	0.010	0.067	0.143
AiHT15	*S. bicolor* and/ or *Z. mays*	5,398	2,609	2,233	12	11	7*	11	Incomplete	Frameshift; Nonsense;	Sobic.004G339100.1	1,791	0.036	0.150	0.240
AiHT16 *1	*S. bicolor* and/ or *Z. mays*	NA	1,923	1680	5	4	NA	NA	Incomplete	Partial (95%, 3'Lack); Frameshift;	Sobic.005G107500.1	855	0.050	0.282	0.176
AiHT17 *1	*S. bicolor* and/ or *Z. mays*	NA	1,932	1932	1	0	NA	NA	Incomplete	Partial (60%, 5'+3'Lack);	Sobic.005G123900.1	1,710	0.034	0.125	0.268
AiHT18 *1	*S. bicolor* and/ or *Z. mays*	NA	1,247	1056	10	9	NA	NA	Complete	-	Sobic.006G016900.1	735	0.028	0.567	0.049
AiHT19	*S. bicolor* and/ or *Z. mays*	1,101	967	605	2	1	1	1	Incomplete	Frameshift;	Sobic.006G154500.1	459	0.048	0.203	0.239
AiHT20	*S. bicolor* and/ or *Z. mays*	3,881	1,824	1,479	12	11	11	11	Incomplete	Partial (85%, 5'Lack); Nonsense;	Sobic.006G230400.1	1,215	0.016	0.128	0.128
AiHT21 *1	*S. bicolor* and/ or *Z. mays*	NA	561	561	4	3	NA	NA	Incomplete	Partial (45%, 3'Lack)	Sobic.007G002600.1	183	0.007	0.236	0.030
AiHT22 *1	*S. bicolor* and/ or *Z. mays*	NA	524	330	1	0	-	-	Complete	-	Sobic.007G019100.1	318	0.042	0.229	0.181
AiHT23	*S. bicolor* and/ or *Z. mays*	4,292	1,822	1,451	2	1	1	1	Incomplete	Frameshift;	Sobic.007G053100.1	1,403	0.010	0.161	0.060
AiHT24	*S. bicolor* and/ or *Z. mays*	4,498	2,555	1,215	5	4	3*	4	Nearly complete	Partial (90%, 5'Lack)	Sobic.007G119400.1	1,077	0.050	0.162	0.310
AiHT25 *1	*S. bicolor* and/ or *Z. mays*	NA	2,479	1644	9	8	NA	NA	Complete	-	Sobic.008G067100.1	1,467	0.039	0.180	0.215
AiHT26 *1	*S. bicolor* and/ or *Z. mays*	NA	805	805	5	4	NA	NA	Incomplete	Partial (35%, 5'+3'Lack); Frameshift;	Sobic.009G002300.1	681	0.029	0.081	0.351
AiHT27 *1	*S. bicolor* and/ or *Z. mays*	NA	442	345	1	0	NA	NA	Incomplete	Partial (35%, 5'Lack)	Sobic.009G034400.1	339	0.055	0.128	0.429
AiHT28	*S. bicolor* and/ or *Z. mays*	3,834	1,442	1,367	9	8	7*	8	Incomplete	Partial (75%, 5'+3'Lack); Frameshift;	Sobic.009G194000.1	447	0.033	0.093	0.350
AiHT29	*S. bicolor* and/ or *Z. mays*	4,760	1,120	1,058	4	3	0*	3	Incomplete	Frameshift;	Sobic.010G004200.1	390	0.134	0.257	0.519
AiHT30	*S. bicolor* and/ or *Z. mays*	3,140	1,557	1,002	4	3	3	3	Complete	-	Sobic.010G062000.1	525	0.065	0.214	0.301
AiHT31 *1	*S. bicolor* and/ or *Z. mays*	NA	731	731	4	3	NA	NA	Incomplete	Partial (20%, 5'Lack); Frameshift; Nonsense;	Sobic.010G085100.1	726	0.044	0.013	3.293
AiHT32 *1	*S. bicolor* and/ or *Z. mays*	NA	1,403	1092	1	0	NA	NA	Complete	-	Sobic.010G222100.1	1,092	0.085	0.340	0.251
AiHT33 *1	*S. bicolor* and/ or *Z. mays*	NA	1,249	1098	2	1	NA	NA	Complete	-	Pavir.Ea02508.1	240	0.040	0.122	0.330
AiHT34	*S. bicolor* and/ or *Z. mays*	3,782	2,343	1,698	8	7	7	7	Complete	-	Sobic.010G254700.1	1,149	0.018	0.181	0.099
AiHT35	*S. bicolor* and/ or *Z. mays*	4,019	1,450	1,296	10	9	9	8	Complete	-	Sobic.010G254900.1	543	0.054	0.199	0.271
AiHT36	*S. bicolor* and/ or *Z. mays*	4,335	1,365	624	6	5	0*	5	Incomplete	Splicing_error;	Sobic.010G270300.1	333	0.008	0.106	0.076
AiHT37	*S. bicolor* and/ or *Z. mays*	3,475	1,235	1,143	3	2	2	2	Complete	-	Sobic.010G270700.1	678	0.037	0.179	0.207
AiHT38	*S. bicolor* and/ or *Z. mays*	1,414	865	432	1	0	-	-	Incomplete	Partial (50%, 5'Lack)	Sobic.010G271400.1	237	0.023	0.328	0.070
AiHT39	*S. bicolor* and/ or *Z. mays*	1,450	942	468	1	0	-	-	Incomplete	Partial (40%, 5'Lack)	GRMZM2G074358_T01	219	0.025	0.176	0.140
AiHT40	*S. bicolor* and/ or *Z. mays*	7,216	3,958	2,840	12	11	11	10	Nearly complete	Partial (90%, 5'Lack)	Sobic.010G273800.1	2,031	0.015	0.096	0.156
AiHT41	*S. bicolor* and/ or *Z. mays*	4,851	2,761	1,446	8	7	7	7	Complete	-	Sobic.010G273900.1	1,296	0.024	0.092	0.261
AiHT42 *1	*S. bicolor* and/ or *Z. mays*	NA	1,402	1272	10	9	NA	NA	Complete	-	Sobic.010G274000.1	1,137	0.032	0.118	0.270
AiHT43	*S. bicolor* and/ or *Z. mays*	3,896	1,545	1,479	8	7	6*	7	Incomplete	Indel(300bp);	Sobic.010G274500.1	1,056	0.005	0.162	0.030
AiHT44	*S. bicolor* and/ or *Z. mays*	6,553	1,796	1,527	7	6	6	6	Complete	-	Sobic.010G274600.1	1,293	0.020	0.110	0.178
AiHT45	*S. bicolor* and/ or *Z. mays*	4,976	1,493	801	5	4	4	4	Complete	-	Sobic.010G275100.1	798	0.012	0.091	0.127
AiHT46	*S. bicolor* and/ or *Z. mays*	2,002	500	433	6	5	5	5	Incomplete	Partial (60%, 5'Lack): Indel(60bp);	Sobic.010G275200.1	399	0.013	0.189	0.068
AiHT47	*S. bicolor* and/ or *Z. mays*	5,695	1,913	804	8	7	7	7	Complete	-	Sobic.007G184100.1	555	0.087	0.132	0.661
AiHT48	*S. bicolor* and/ or *Z. mays*	2,111	1,326	720	1	0	-	-	Incomplete	Partial (45%, 5'+3'Lack); Frameshift;	GRMZM2G082131_T01	660	0.061	0.115	0.527
AiHT49 *1	*S. bicolor* and/ or *Z. mays*	NA	1,514	1407	5	4	NA	NA	Complete	-	Sobic.K002400.1	303	0.004	0.061	0.072
AiHT50	*S. italica* and/or *P. virgatum*	6,589	2,037	492	6	5	5	2	Incomplete	Partial (60%, 5'Lack)	Si018070m	399	0.000	0.011	0.000
AiHT51 *1	*S. italica* and/or *P. virgatum*	NA	1,047	1047	2	1	NA	NA	Complete	-	Si030346m	717	0.040	0.376	0.106
AiHT52	*S. italica* and/or *P. virgatum*	2,077	504	504	1	0	-	-	Incomplete	Partial (35%, 5'+3'Lack)	Si029857m	432	0.016	0.274	0.059
AiHT53	*S. italica* and/or *P. virgatum*	4,284	930	657	3	2	2	2	Incomplete	Nonsense;	Pavir.Bb02626.1	330	0.035	0.108	0.321
AiHT54	*S. italica* and/or *P. virgatum*	1,849	980	545	5	4	4	3	Incomplete	Partial (25%, 5'+3'Lack); Nonsense	Si021400m	540	0.020	0.153	0.130
AiHT55	*S. italica* and/or *P. virgatum*	5,336	2,602	1,218	13	12	12	11	Incomplete	Partial (60%, 5'+3'Lack); Frameshift; Nonsense;	Si021383m	789	0.040	0.162	0.244
AiHT56	*S. italica* and/or *P. virgatum*	4,913	1,782	946	4	3	0*	3	Incomplete	Partial (80%, 3'Lack); Frameshift; Nonsense;	Si006617m	690	0.051	0.228	0.224
AiHT57 *1	*S. italica* and/or *P. virgatum*	NA	780	633	7	6	NA	NA	Complete	-	Pavir.J06884.1	327	0.000	0.220	0.000
AiHT58	*S. italica* and/or *P. virgatum*	4,263	1,393	1,163	9	8	8	8	Incomplete	Partial (45%, 3'Lack)	Pavir.Da00644.1	825	0.008	0.108	0.078
AiHT59	*S. italica* and/or *P. virgatum*	3,017	2,579	507	2	1	1	1	Incomplete	Partial (25%, 5'Lack)	Si006000m	267	0.000	0.031	0.000
AiHT60 *1	*S. italica* and/or *P. virgatum*	NA	1,233	1167	1	0	NA	NA	Complete	-	Pavir.J11373.1	1,083	0.111	0.400	0.278
AiHT61 *1	PACCMAD *2	NA	2,693	2259	6	5	NA	NA	Incomplete	Frameshift; Splicing_error;	Pavir.Ib03807.1	603	0.104	0.239	0.433
AiHT62 *1	PACCMAD *2	NA	1,899	1304	2	1	NA	NA	Incomplete	Frameshift; Splicing_error;	Pavir.Ia01375.1	564	0.026	0.221	0.115
AiHT63	PACCMAD *2	3,395	2,546	1,428	1	0	-	-	Incomplete	Frameshift;	Si035427m	303	0.075	0.411	0.183
AiHT64	PACCMAD *2	3,621	1,695	1,493	4	3	3	2	Incomplete	Frameshift;	LOC_Os07g45195.1	561	0.119	0.302	0.393
AiHT65	PACCMAD *2	2,310	534	427	2	1	1	1	Incomplete	Partial (25%, 5'Lack); Nonsense	Sobic.002G347900.1	426	0.042	0.513	0.081
AiHT66	PACCMAD *2	2,550	2,020	1,228	2	1	1	1	Incomplete	Partial (50%, 3'Lack); Frameshift;	Pavir.Ba00530.1	792	0.082	0.312	0.262
AiHT67	PACCMAD *2	1,679	1,410	1,250	1	0	-	-	Incomplete	Partial (85%, 5'Lack)	Sobic.002G166200.1	699	0.025	0.210	0.118
AiHT68	PACCMAD *2	3,251	1,625	1,431	3	2	2	0	Incomplete	Nonsense;	Sobic.006G024000.1	1,023	0.071	0.404	0.175
AiHT69 *1	PACCMAD *2	NA	1,239	1095	8	7	NA	NA	Nearly complete	Partial (95%, 5'Lack)	Sobic.008G025400.1	456	0.123	0.367	0.336
AiHT70	PACCMAD *2	4,957	2,059	1,040	7	6	6	6	Incomplete	Frameshift;	Pavir.Da02235.1	1,011	0.121	0.575	0.210
AiHT71	*B. distachyon*	4,065	2,811	2,420	4	3	3	3	Incomplete	Partial (60%, 5'+3'Lack); Frameshift;	Bradi2g00417.1	1,704	0.071	0.434	0.163
AiHT72	*B. distachyon*	1,511	1,209	1,209	2	1	0	1	Incomplete	Partial (95%, 3'Lack);	Bradi3g55500.1	684	0.089	0.085	1.049
AiHT73	*B. distachyon*	1,447	414	414	3	2	2	1	Incomplete	Partial (55%, 5'+3'Lack)	Bradi4g31220.2	381	0.106	0.276	0.385
AiHT74	Poaceae	3,257	1,030	963	8	7	7	6	Incomplete	Partial (60%, 3'Lack)	Pavir.Eb02626.1	498	0.094	0.322	0.291
AiHT75	Poaceae	1,302	1,335	1,335	2	1	1	1	Incomplete	Partial (45%, 5'+3'Lack)	GRMZM2G151041_T01	702	0.017	0.400	0.043
AiHT76	Poaceae	6,285	1,454	837	4	3	3	3	Incomplete	Partial (65%, 3'Lack)	Pavir.J26297.1	351	0.100	0.273	0.365
AiHT77 *1	Poaceae	NA	1,977	1917	4	3	NA	NA	Complete	-	Si034631m	1,782	0.031	0.405	0.076
AiHT78 *1	Poaceae	NA	1,818	1380	5	4	NA	NA	Complete	-	Pavir.Fb01931.1	1,200	0.073	0.481	0.152
AiHT79 *1	Poaceae	NA	2,211	1905	11	10	NA	NA	Complete	-	Pavir.Ia03658.1	1,023	0.104	0.265	0.390
AiHT80 *1	Poaceae	NA	1,909	1374	2	1	NA	NA	Complete	-	Sobic.004G328500.1	264	0.036	0.485	0.074
AiHT81-1	*S. italica* and/or *P. virgatum*	1,943	1,494	1,341	1	0	-	-	Complete	-	Si022060m	690	0.075	0.178	0.422
AiHT81-2	*B. distachyon*	2,405	1,993	1,497	2	1	1	1	Complete	-	Bradi1g00410.1	690	0.088	0.154	0.574

*1 : Genes are duplidcated in parasite species.

*2: PACCMAD include S. italica, P. virgatum, S. bicolor and Z. mays.

*3 : At least one RNA read for each Exon-Intron junction are incorrectly spliced.

*4 : Number of introns without unassembled gap.

*5: CDS length used for phyogenetic and Ka & Ks analyses

Table S7 Summary of the results of BLASTN & TBLASTX searches for CDSs of HGT genes against 52 angiosperm species

52 Angiosperm species	Species	*Mimulus guttatus*		*Andrographis paniculata*		*Avicennia officinalis*		*Olea europaea*		*Paulownia tomentosa*		*Salvia pomifera*		*Tectona grandis*		*Solanum lycopersicum*		*Solanum tuberosum*		*Glycine max*		*Phaseolus vulgaris*		*Lotus japonicus*		[illegible]
	Family	Phrymaceae		Acanthaceae		Acanthaceae		Oleaceae		Paulowniaceae		Lamiaceae		Lamiaceae		Solanaceae		Solanaceae		Fabaceae		Fabaceae		Fabaceae		[illegible]
	Order	Lamiales		Lamiales		Lamiales		Lamiales		Lamiales		Lamiales		Lamiales		Solanales		Solanales		Fabales		Fabales		Fabales		[illegible]
	Higher Categories	Asterids		Asterids		Asterids		Asterids		Asterids		Asterids		Asterids		Asterids		Asterids		Fabids / Rosids		Fabids / Rosids		Fabids / Rosids		[illegible]
	Sequence Files	Mguttatus_256_v2.0.cds		GBSY01.1.fa		GFLY01.1.fsa_nt		GDUL01.1.fsa_nt		GEFV01.1.fsa_nt		GDKL01.1.fsa_nt		GFGL01.1.fsa_nt		Slycopersicum_225_iTAGv2		Stuberosum_206_v3.4.cds.fa		Gmax_275_Wm82.a2.v1.cds		Pvulgaris_218_v1.0.cds.fa		Lj2.5_cds.fa		[illegible]
	Databases	PhytozomeV10		NCBI		NCBI		NCBI		NCBI		NCBI		NCBI		PhytozomeV10		PhytozomeV10		PhytozomeV10		PhytozomeV10		Kazusa		[illegible]
	CDS/TSA	CDS		TSA		TSA		TSA		TSA		TSA		TSA		CDS		CDS		CDS		CDS		CDS		[illegible]
	HGT Loci	Subject Sequence ID	Bit Sc	Subject Sequence ID	Bit Sc	Subject Sequence ID	Bit Sc	Subject Sequence ID	Bit Sc	Subject Sequence ID	Bit Sc	Subject Sequence ID	Bit Sc	Subject Sequence ID	Bit Sc	Subject Sequence ID	Bit Sc	Subject Sequence ID	Bit Sc	Subject Sequence ID	Bit Sc	Subject Sequence ID	Bit Sc	Subject Sequence ID	[illegible]	[illegible]
BLASTN search	OmHT01	NoHit	-	NoHit	-	NoHit	-	NoHit	-	NoHit	-	NoHit	-	NoHit	-	NoHit	-	NoHit	-	Glyma.U038300.2	388	Phvul.009G074500.1	357	LjSGA_051584.1	[illegible]	[illegible]
	OmHT02	NoHit	-	NoHit	-	NoHit	-	NoHit	-	NoHit	-	NoHit	-	NoHit	-	NoHit	-	PGSC0003DMT400006222	[illegible]	Glyma.19G194700.3	344	Phvul.001G189600.2	294	chr1.CM0010.110.r2.[illegible]	[illegible]	[illegible]
	OmHT03	NoHit	-	NoHit	-	NoHit	-	NoHit	-	NoHit	-	NoHit	-	NoHit	-	NoHit	-	NoHit	-	Glyma.06G316500.1	675	Phvul.008G161800.1	619	LjSGA_048522.1	[illegible]	[illegible]
	OmHT04	NoHit	-	NoHit	-	NoHit	-	NoHit	-	NoHit	-	NoHit	-	NoHit	-	NoHit	-	NoHit	-	Glyma.06G270300.1	###	Phvul.011G139500.1	551	LjSGA_055600.1	[illegible]	[illegible]
	OmHT05	NoHit	-	NoHit	-	GFLY01005902.1	616	NoHit	-	NoHit	-	NoHit	-	NoHit	-	NoHit	-	NoHit	-	Glyma.12G134500.1	###	Phvul.011G139400.1	###	chr3.CM1543.10.r2.[illegible]	[illegible]	[illegible]
	OmHT06	NoHit	-	NoHit	-	NoHit	-	NoHit	-	NoHit	-	NoHit	-	NoHit	-	NoHit	-	NoHit	-	NoHit	-	Phvul.003G105100.1	520	LjSGA_011895.2	[illegible]	[illegible]
	OmHT07	NoHit	-	NoHit	-	NoHit	-	NoHit	-	NoHit	-	NoHit	-	NoHit	-	NoHit	-	NoHit	-	Glyma.14G019100.1	###	Phvul.008G270700.1	###	LjSGA_024515.1	[illegible]	[illegible]
	OmHT08	NoHit	-	NoHit	-	NoHit	-	NoHit	-	NoHit	-	NoHit	-	GFGL01153285.1	473	NoHit	-	PGSC0003DMT400002496	[illegible]	Glyma.05G003500.1	###	Phvul.004G006800.1	###	chr2.CM0263.90.r2.[illegible]	[illegible]	[illegible]
	OmHT09	NoHit	-	NoHit	-	NoHit	-	NoHit	-	NoHit	-	NoHit	-	NoHit	-	NoHit	-	NoHit	-	Glyma.03G107700.1	900	Phvul.010G044700.1	839	NoHit	[illegible]	[illegible]
	OmHT10	NoHit	-	NoHit	-	NoHit	-	NoHit	-	NoHit	-	NoHit	-	NoHit	-	NoHit	-	NoHit	-	Glyma.02G190700.1	###	Phvul.007G249500.1	###	chr5.CM0278.110.r2.[illegible]	[illegible]	[illegible]
	OmHT11	NoHit	-	NoHit	-	NoHit	-	NoHit	-	NoHit	-	NoHit	-	NoHit	-	Solyc03g117330.2.1	195	PGSC0003DMT400003207	[illegible]	Glyma.03G224500.1	603	Phvul.001G218300.1	586	chr2.CM0177.390.r2.[illegible]	[illegible]	[illegible]
	OmHT12	NoHit	-	NoHit	-	NoHit	-	NoHit	-	NoHit	-	NoHit	-	NoHit	-	NoHit	-	NoHit	-	Glyma.13G189600.1	387	NoHit	-	chr3.CM0005.510.r2.[illegible]	[illegible]	[illegible]
	OmHT13	NoHit	-	NoHit	-	NoHit	-	NoHit	-	NoHit	-	NoHit	-	NoHit	-	NoHit	-	NoHit	-	Glyma.02G047300.1	898	Phvul.003G263600.1	870	NoHit	[illegible]	[illegible]
	OmHT14	NoHit	-	NoHit	-	NoHit	-	NoHit	-	NoHit	-	NoHit	-	NoHit	-	NoHit	-	NoHit	-	Glyma.13G203900.1	279	Phvul.008G076500.1	538	NoHit	[illegible]	[illegible]
	OmHT15	NoHit	-	gb|GBSY01014937.1|	399	NoHit	-	NoHit	-	NoHit	-	NoHit	-	NoHit	-	NoHit	-	NoHit	-	Glyma.02G010500.1	###	Phvul.001G214700.1	###	chr1.CM0122.1830.r2.[illegible]	[illegible]	[illegible]
	OmHT16	NoHit	-	NoHit	-	NoHit	-	NoHit	-	NoHit	-	NoHit	-	NoHit	-	NoHit	-	NoHit	-	NoHit	-	NoHit	-	NoHit	[illegible]	[illegible]
	OmHT17	NoHit	-	NoHit	-	NoHit	-	NoHit	-	NoHit	-	gb|GDKL01017865.1|	760	NoHit	-	NoHit	-	NoHit	-	Glyma.08G311400.1	###	Phvul.006G036200.1	###	chr6.CM0836.110.r2.[illegible]	[illegible]	[illegible]
	OmHT18	Migut.N01247.1	372	NoHit	-	NoHit	-	NoHit	-	NoHit	-	gb|GDKL01028498.1|	274	NoHit	-	NoHit	-	NoHit	-	Glyma.11G116800.1	###	Phvul.011G044300.1	###	chr3.CM0091.1190.r2.[illegible]	[illegible]	[illegible]
[illegible]	[illegible]	[illegible]	[illegible]	[illegible]	[illegible]	[illegible]	[illegible]	[illegible]	[illegible]	[illegible]	[illegible]	[illegible]	[illegible]	[illegible]	[illegible]	[illegible]	[illegible]	[illegible]	[illegible]	[illegible]	[illegible]	[illegible]	[illegible]	[illegible]	[illegible]	[illegible]
	AiHT68	Migut.N03050.1	231	gb|GBSY01019740.1|	226	GFLY01009276.1	264	GDUL01001074.1	255	GEFV01017335.1	260	gb|GDKL01016456.1|	279	GFGL01120452.1	270	Solyc12g096820.1.1	242	PGSC0003DMT40007[illegible]	249	[illegible]	[illegible]	[illegible]	[illegible]	[illegible]	[illegible]	[illegible]
	AiHT69	Migut.B01098.1	181	gb|GBSY01018260.1|	166	GFLY01024407.1	178	GDUL01003641.1	77	GEFV01158348.1	174	gb|GDKL01024557.1|	183	GFGL01125179.1	186	Solyc01g097190.2.1	119	PGSC0003DMT40009[illegible]	100	[illegible]	[illegible]	[illegible]	[illegible]	[illegible]	[illegible]	[illegible]
	AiHT70	Migut.I00845.1	183	gb|GBSY01016602.1|	194	GFLY01037578.1	184	GDUL01045142.1	55	GEFV01114068.1	193	gb|GDKL01043006.1|	183	GFGL01121887.1	190	Solyc06g066780.2.1	191	PGSC0003DMT40004[illegible]	190	[illegible]	[illegible]	[illegible]	[illegible]	[illegible]	[illegible]	[illegible]
	AiHT71	Migut.M01669.1	502	gb|GBSY01022377.1|	361	GFLY01010272.1	443	GDUL01026477.1	79	GEFV01151871.1	507	gb|GDKL01047081.1|	359	GFGL01157948.1	511	Solyc07g065320.2.1	504	PGSC0003DMT40005[illegible]	504	[illegible]	[illegible]	[illegible]	[illegible]	[illegible]	[illegible]	[illegible]
	AiHT72	Migut.L01485.1	37	gb|GBSY01019911.1|	35	GFLY01025419.1	37	GDUL01005907.1	35	GEFV01024456.1	35	gb|GDKL01010299.1|	38	GFGL01185406.1	40	Solyc08g074370.2.1	35	PGSC0003DMT40000[illegible]	33	[illegible]	[illegible]	[illegible]	[illegible]	[illegible]	[illegible]	[illegible]
	AiHT73	Migut.L01586.1	139	gb|GBSY01017591.1|	153	GFLY01036746.1	153	GDUL01001508.1	156	GEFV01115367.1	150	gb|GDKL01028372.1|	147	GFGL01105769.1	147	Solyc07g042250.2.1	146	PGSC0003DMT40000[illegible]	144	[illegible]	[illegible]	[illegible]	[illegible]	[illegible]	[illegible]	[illegible]
	AiHT74	Migut.H00639.1	434	gb|GBSY01021188.1|	425	GFLY01009559.1	415	GDUL01001936.1	177	GEFV01121857.1	431	gb|GDKL01017293.1|	440	GFGL01151667.1	436	Solyc05g012480.2.1	395	PGSC0003DMT40000[illegible]	423	[illegible]	[illegible]	[illegible]	[illegible]	[illegible]	[illegible]	[illegible]
	AiHT75	Migut.B01245.1	453	gb|GBSY01022334.1|	462	GFLY01027320.1	456	GDUL01010241.1	194	GEFV01144670.1	455	gb|GDKL01003453.1|	462	GFGL01155097.1	464	Solyc04g011350.2.1	453	PGSC0003DMT40006[illegible]	455	[illegible]	[illegible]	[illegible]	[illegible]	[illegible]	[illegible]	[illegible]
	AiHT76	Migut.F00353.1	56	gb|GBSY01021885.1|	63	GFLY01018416.1	64	GDUL01070113.1	31	GEFV01060874.1	66	gb|GDKL01051269.1|	63	GFGL01137706.1	56	Solyc08g080690.2.1	65	PGSC0003DMT40000[illegible]	59	[illegible]	[illegible]	[illegible]	[illegible]	[illegible]	[illegible]	[illegible]
	AiHT77	Migut.G00281.3	###	gb|GBSY01021600.1|	###	GFLY01029740.1	###	GDUL01019608.1	213	GEFV01089123.1	###	gb|GDKL01055486.1|	###	GFGL01088616.1	###	Solyc06g006080.2.1	###	PGSC0003DMT40001[illegible]	###	[illegible]	[illegible]	[illegible]	[illegible]	[illegible]	[illegible]	[illegible]
	AiHT78	Migut.N01367.1	648	gb|GBSY01019772.1|	638	GFLY01013606.1	631	GDUL01001309.1	363	GEFV01087649.1	630	gb|GDKL01022451.1|	462	GFGL01140422.1	468	Solyc01g112280.2.1	630	PGSC0003DMT40000[illegible]	417	[illegible]	[illegible]	[illegible]	[illegible]	[illegible]	[illegible]	[illegible]
	AiHT79	Migut.L00308.1	312	gb|GBSY01013518.1|	263	GFLY01030127.1	430	GDUL01059451.1	65	GEFV01116666.1	452	gb|GDKL01024529.1|	447	GFGL01156650.1	452	Solyc02g085820.2.1	452	PGSC0003DMT40003[illegible]	459	[illegible]	[illegible]	[illegible]	[illegible]	[illegible]	[illegible]	[illegible]
	AiHT80	Migut.M01867.1	35	gb|GBSY01021313.1|	35	GFLY01037051.1	34	GDUL01065936.1	34	GEFV01071428.1	33	gb|GDKL01004557.1|	32	GFGL01051457.1	38	Solyc04g082060.2.1	39	PGSC0003DMT40002[illegible]	39	[illegible]	[illegible]	[illegible]	[illegible]	[illegible]	[illegible]	[illegible]
	AiHT81-1	Migut.D02473.2	34	gb|GBSY01022015.1|	33	GFLY01009946.1	32	GDUL01028787.1	35	GEFV01140453.1	35	gb|GDKL01047142.1|	33	GFGL01170525.1	39	Solyc03g120130.2.1	42	PGSC0003DMT40001[illegible]	42	[illegible]	[illegible]	[illegible]	[illegible]	[illegible]	[illegible]	[illegible]
	AiHT81-2	Migut.N01727.1	36	gb|GBSY01005881.1|	35	GFLY01021567.1	36	GDUL01029040.1	31	GEFV01131779.1	39	gb|GDKL01005880.1|	37	GFGL01208987.1	42	Solyc08g061610.2.1	33	PGSC0003DMT40009[illegible]	35	[illegible]	[illegible]	[illegible]	[illegible]	[illegible]	[illegible]	[illegible]

Colors indicate that

Lamiales (excluding Orobanchaceae) is the closest relatives to the parasite family Orobanchaceae.

Solanales is the closest relative to the order Lamiales.

Fabaceae is the major host family of *O. minor*.

Fabids (excluding Fabaceae) is the closest relatives to the family Fabaceae.

Poaceae is the major host family of *A. indica*.

Commelinids (excluding Poaceae) is the closest relatives to the family Poaceae.

Table S8 **Summary of the results of the PAML analyses**

Parasite genes	Parasite species	Host1 genes	Host1 species	Host2 genes	Host2 species	Seq length	Model 0(Null)	Model 2(Alternative)					
							ω(0)	ω(Parasite)	ω(Hosts)	High/Low	-2lnL[M2/M0], df=1	P-value	FDR(Benjamini-Hochberg)
OmHT01	*O. minor*	RC.42229	*T. pratense*	Medtr1g017270.1	*M. truncatula*	435	0.22	1.08	0.12	H	8.293	4.0E-03	2.9E−02
OmHT02	*O. minor*	RC.10825	*T. pratense*	Medtr1g060070.1	*M. truncatula*	327	0.18	0.55	0.09	H	7.721	5.5E-03	3.0E−02
OmHT03	*O. minor*	RC.29156	*T. pratense*	Medtr3g007750.1	*M. truncatula*	591	0.19	0.45	0.12	H	5.170	2.3E-02	7.2E−02
OmHT04	*O. minor*	RC.34162	*T. pratense*	Medtr4g080790.1	*M. truncatula*	672	0.17	0.15	0.18	L	0.078	7.8E-01	9.0E−01
OmHT05	*O. minor*	RC.32426	*T. pratense*	Medtr4g080800.1	*M. truncatula*	1,431	0.19	0.24	0.18	H	0.349	5.5E-01	7.2E−01
OmHT06	*O. minor*	RC.13251	*T. pratense*	Medtr4g119840.1	*M. truncatula*	330	0.24	0.23	0.25	L	0.007	9.3E-01	9.3E−01
OmHT07	*O. minor*	RC.41913	*T. pratense*	Medtr5g093580.1	*M. truncatula*	1,221	0.17	0.28	0.15	H	1.061	3.0E-01	5.1E−01
OmHT08	*O. minor*	RC.37121	*T. pratense*	Medtr6g004840.1	*M. truncatula*	5,706	0.40	0.43	0.39	H	0.295	5.9E-01	7.2E−01
OmHT09	*O. minor*	RC.41584	*T. pratense*	Medtr8g007390.1	*M. truncatula*	1,104	0.16	0.37	0.12	H	5.288	2.1E-02	7.9E−02
OmHT10	*O. minor*	RC.42261	*T. pratense*	Medtr3g045150.1	*M. truncatula*	1,170	0.30	0.61	0.25	H	4.300	3.8E-02	9.3E−02
OmHT11	*O. minor*	RC.6350	*T. pratense*	Medtr3g048190.1	*M. truncatula*	1,329	0.31	0.24	0.35	L	1.444	2.3E-01	4.6E−01
OmHT12	*O. minor*	RC.37978	*T. pratense*	chr3.CM0005.510.r2.m	*L. japnicus*	309	0.37	999.00	0.32	H	4.424	3.5E-02	9.7E−02
OmHT13	*O. minor*	Medtr8g075480.1	*M. truncatula*	Phvul.003G263600.1	*P. vulgaris*	726	0.22	0.25	0.22	H	0.068	7.9E-01	8.7E−01
OmHT14	*O. minor*	Medtr7g074730.1	*M. truncatula*	Phvul.008G076500.1	*P. vulgaris*	795	0.29	0.42	0.24	H	2.235	1.3E-01	3.0E−01
OmHT15	*O. minor*	Medtr1g086640.1	*M. truncatula*	Glyma.02G010500.1	*G. max*	1,974	0.09	0.20	0.05	H	23.183	1.5E-06	3.2E−05
OmHT16	*O. minor*	RC.31901	*T. pratense*	Medtr5g464730.1	*M. truncatula*	1,047	0.46	0.36	0.50	L	0.612	4.3E-01	6.8E−01
OmHT17	*O. minor*	Medtr3g048950.1	*M. truncatula*	Phvul.006G036200.1	*P. vulgaris*	1,389	0.13	0.10	0.15	L	1.165	2.8E-01	5.1E−01
OmHT18	*O. minor*	Medtr4g078640.1	*M. truncatula*	Phvul.011G044300.1	*P. vulgaris*	2,103	0.19	0.33	0.15	H	10.646	1.1E-03	1.2E−02
OmHT19	*O. minor*	chr4.LjT10K15.20.r2.m	*L. japnicus*	Medtr4g098640.1	*M. truncatula*	660	0.19	0.18	0.19	L	0.013	9.1E-01	9.5E−01
OmHT20	*O. minor*	chr1.LjT23J20.40.r2.m	*L. japnicus*	Medtr7g094660.1	*M. truncatula*	2,358	0.50	0.83	0.43	H	6.797	9.1E-03	4.0E−02
OmHT21	*O. minor*	Glyma.13G370500.1	*G. max*	Medtr8g099865.1	*M. truncatula*	1,401	0.37	0.42	0.35	H	0.501	4.8E-01	6.6E−01
OmHT22	*O. minor*	comp2631_c0_seq1	*L. angstifolius*	Medtr3g460700.1	*M. truncatula*	984	0.13	0.07	0.13	L	0.605	4.4E-01	6.4E−01
AiHT01	*A. indica*	Sobic.001G053000.1	*S. bicolor*	Si036782m	*S. italica*	606	0.167	0.454	0.095	H	10.940	9.4E-04	1.3E−02
AiHT02-1	*A. indica*	Sobic.001G137400.1	*S. bicolor*	Pavir.Ib04221.1	*P. virgatum*	1,413	0.425	0.444	0.417	H	0.042	8.4E-01	9.4E−01
AiHT02-2	*A. indica*	Sobic.001G137500.1	*S. bicolor*	Bradi1g68102.1	*B. distachyon*	930	0.371	0.466	0.352	H	0.417	5.2E-01	7.4E−01
AiHT02-3	*A. indica*	Sobic.001G137500.1	*S. bicolor*	Bradi1g68102.1	*B. distachyon*	930	0.363	0.402	0.354	H	0.092	7.6E-01	8.8E−01
AiHT03	*A. indica*	Sobic.001G242800.1	*S. bicolor*	Si036472m	*S. italica*	1,023	0.171	0.628	0.115	H	10.780	1.0E-03	1.2E−02
AiHT04	*A. indica*	Sobic.001G358600.1	*S. bicolor*	Si035364m	*S. italica*	1,314	0.210	0.504	0.143	H	12.243	4.7E-04	7.8E−03
AiHT05	*A. indica*	Sobic.001G453700.1	*S. bicolor*	Si035680m	*S. italica*	1,083	0.022	0.064	0.010	H	4.608	3.2E-02	1.7E−01
AiHT06	*A. indica*	Sobic.001G501300.1	*S. bicolor*	Bradi1g74922.1	*B. distachyon*	417	0.140	0.406	0.052	H	12.687	3.7E-04	7.6E−03
AiHT07	*A. indica*	Sobic.002G128200.1	*S. bicolor*	Si029674m	*S. italica*	1,230	0.116	0.121	0.115	H	0.007	9.4E-01	9.7E−01
AiHT08	*A. indica*	Sobic.002G335400.1	*S. bicolor*	Pavir.Ba00786.1	*P. virgatum*	1,134	0.312	0.441	0.234	H	4.715	3.0E-02	1.7E−01
AiHT09	*A. indica*	Sobic.002G345000.1	*S. bicolor*	Si030798m	*S. italica*	387	0.222	0.158	0.256	L	0.427	5.1E-01	7.6E−01
AiHT10	*A. indica*	Sobic.003G306700.1	*S. bicolor*	Si001397m	*S. italica*	1,347	0.103	0.177	0.084	H	2.223	1.4E-01	3.3E−01
AiHT11	*A. indica*	Sobic.003G438200.1	*S. bicolor*	Pavir.Ea04104.1	*P. virgatum*	615	0.210	0.068	0.238	L	1.332	2.5E-01	4.9E−01
AiHT12	*A. indica*	Sobic.004G196700.1	*S. bicolor*	Si016118m	*S. italica*	1,326	0.254	0.368	0.195	H	4.050	4.4E-02	1.7E−01
AiHT13	*A. indica*	Sobic.004G247800.1	*S. bicolor*	Si017054m	*S. italica*	456	0.145	2.712	0.106	H	7.236	7.1E-03	6.6E−02
AiHT14	*A. indica*	Sobic.004G315700.1	*S. bicolor*	Si016200m	*S. italica*	2,718	0.156	0.196	0.148	H	0.478	4.9E-01	7.8E−01
AiHT15	*A. indica*	Sobic.004G339100.1	*S. bicolor*	Si016449m	*S. italica*	2,130	0.265	0.384	0.227	H	3.571	5.9E-02	2.1E−01
AiHT16	*A. indica*	Sobic.005G107500.1	*S. bicolor*	Si026160m	*S. italica*	1,656	0.148	0.222	0.121	H	4.889	2.7E-02	1.6E−01
AiHT17	*A. indica*	Sobic.005G123900.1	*S. bicolor*	Si027595m	*S. italica*	1,806	0.284	0.472	0.256	H	2.893	8.9E-02	2.5E−01
AiHT18	*A. indica*	Sobic.006G016900.1	*S. bicolor*	Si010368m	*S. italica*	963	0.046	0.048	0.044	H	0.027	8.7E-01	9.5E−01
AiHT19	*A. indica*	Sobic.006G154500.1	*S. bicolor*	Si011043m	*S. italica*	507	0.234	0.460	0.139	H	5.431	2.0E-02	1.4E−01
AiHT20	*A. indica*	Sobic.006G230400.1	*S. bicolor*	Si009926m	*S. italica*	1,464	0.168	0.199	0.160	H	0.248	6.2E-01	7.9E−01
AiHT21	*A. indica*	Sobic.007G002600.1	*S. bicolor*	Si013846m	*S. italica*	555	0.214	0.296	0.155	H	1.768	1.8E-01	3.9E−01
AiHT22	*A. indica*	Sobic.007G019100.1	*S. bicolor*	Pavir.Ia00629.1	*P. virgatum*	327	0.232	0.253	0.223	H	0.041	8.4E-01	9.3E−01
AiHT23	*A. indica*	Sobic.007G053100.1	*S. bicolor*	Si013633m	*S. italica*	1,443	0.067	0.033	0.076	L	1.778	1.8E-01	4.0E−01
AiHT24	*A. indica*	Sobic.007G119400.1	*S. bicolor*	Si013766m	*S. italica*	1,095	0.247	0.689	0.131	H	18.326	1.9E-05	1.5E−03
AiHT25	*A. indica*	Sobic.008G067100.1	*S. bicolor*	Si009759m	*S. italica*	1,488	0.180	0.271	0.140	H	4.081	4.3E-02	1.8E−01
AiHT26	*A. indica*	Sobic.009G002300.1	*S. bicolor*	Si021122m	*S. italica*	789	0.273	0.577	0.199	H	4.057	4.4E-02	1.7E−01
AiHT27	*A. indica*	Sobic.009G034400.1	*S. bicolor*	Si017739m	*S. italica*	336	0.278	0.215	0.307	L	0.191	6.6E-01	8.1E−01
AiHT28	*A. indica*	Sobic.009G194000.1	*S. bicolor*	Si016829m	*S. italica*	1,257	0.214	0.244	0.205	H	0.172	6.8E-01	8.0E−01
AiHT29	*A. indica*	Sobic.010G004200.1	*S. bicolor*	GRMZM2G135044_T01	*Z. mays*	390	0.397	0.463	0.358	H	0.260	6.1E-01	8.0E−01
AiHT30	*A. indica*	Sobic.010G062000.1	*S. bicolor*	GRMZM2G000713_T01	*Z. mays*	855	0.428	0.363	0.467	L	0.359	5.5E-01	7.5E−01
AiHT31	*A. indica*	Sobic.010G085100.1	*S. bicolor*	Si005702m	*S. italica*	687	0.104	0.263	0.061	H	4.606	3.2E-02	1.6E−01
AiHT32	*A. indica*	Sobic.010G222100.1	*S. bicolor*	GRMZM2G009724_T01	*Z. mays*	1,083	0.299	0.353	0.255	H	1.234	2.7E-01	5.1E−01
AiHT33	*A. indica*	Sobic.010G222400.1	*S. bicolor*	Pavir.Ea02508.1	*P. virgatum*	966	0.580	0.529	0.625	L	0.250	6.2E-01	8.0E−01
AiHT34	*A. indica*	Sobic.010G254700.1	*S. bicolor*	Si006225m	*S. italica*	1,407	0.155	0.078	0.195	L	4.288	3.8E-02	1.8E−01
AiHT35	*A. indica*	Sobic.010G254900.1	*S. bicolor*	Si006602m	*S. italica*	1,020	0.291	0.291	0.291	H	0.000	1.0E+00	1.0E+00
AiHT36	*A. indica*	Sobic.010G270300.1	*S. bicolor*	Si008168m	*S. italica*	336	0.132	0.121	0.133	L	0.006	9.4E-01	9.6E−01
AiHT37	*A. indica*	Sobic.010G270700.1	*S. bicolor*	Pavir.Db00017.1	*P. virgatum*	996	0.297	0.259	0.318	L	0.392	5.3E-01	7.5E−01
AiHT38	*A. indica*	Sobic.010G271400.1	*S. bicolor*	Si007045m	*S. italica*	273	0.175	0.069	0.253	L	3.181	7.5E-02	2.5E−01
AiHT39	*A. indica*	Sobic.010G273700.1	*S. bicolor*	GRMZM2G074358_T01	*Z. mays*	294	0.137	0.039	0.200	L	2.426	1.2E-01	3.0E−01
AiHT40	*A. indica*	Sobic.010G273800.1	*S. bicolor*	Si005911m	*S. italica*	2,115	0.184	0.225	0.175	H	0.381	5.4E-01	7.4E−01
AiHT41	*A. indica*	Sobic.010G273900.1	*S. bicolor*	Si006336m	*S. italica*	1,404	0.191	0.336	0.159	H	3.025	8.2E-02	2.5E−01
AiHT42	*A. indica*	Sobic.010G274000.1	*S. bicolor*	Si006509m	*S. italica*	1,269	0.179	0.623	0.104	H	18.249	1.9E-05	8.0E−04
AiHT43	*A. indica*	Sobic.010G274500.1	*S. bicolor*	Si006189m	*S. italica*	1,476	0.063	0.066	0.062	H	0.014	9.1E-01	9.7E−01
AiHT44	*A. indica*	Sobic.010G274600.1	*S. bicolor*	Si000998m	*S. italica*	1,512	0.188	0.236	0.176	H	0.553	4.6E-01	8.1E−01
AiHT45	*A. indica*	Sobic.010G275100.1	*S. bicolor*	Si007094m	*S. italica*	798	0.104	0.102	0.105	L	0.001	9.7E-01	9.8E−01
AiHT46	*A. indica*	Sobic.010G275200.1	*S. bicolor*	Si007288m	*S. italica*	402	0.072	0.060	0.077	L	0.073	7.9E-01	9.0E−01
AiHT47	*A. indica*	Sobic.007G184100.1	*S. bicolor*	Si002590m	*S. italica*	771	0.485	0.544	0.468	H	0.115	7.3E-01	8.6E−01
AiHT48	*A. indica*	GRMZM2G082131_T01	*Z. mays*	Si040216m	*S. italica*	699	0.378	1.127	0.271	H	8.184	4.2E-03	4.4E−02
AiHT49	*A. indica*	Sobic.K002400.1	*S. bicolor*	Si021935m	*S. italica*	828	0.145	0.206	0.132	H	0.461	5.0E-01	7.6E−01
AiHT50	*A. indica*	Si018070m	*S. italica*	Sobic.004G097200.1	*S. bicolor*	483	0.090	0.000	0.093	L	0.508	4.8E-01	7.9E−01
AiHT51	*A. indica*	Si030346m	*S. italica*	Sobic.002G384400.1	*S. bicolor*	1,038	0.188	0.128	0.273	L	6.261	1.2E-02	1.0E−01
AiHT52	*A. indica*	Si029857m	*S. italica*	Sobic.002G346900.1	*S. bicolor*	495	0.047	0.105	0.025	H	3.184	7.4E-02	2.6E−01
AiHT53	*A. indica*	Si032702m	*S. italica*	Sobic.002G290900.1	*S. bicolor*	648	0.226	0.403	0.176	H	2.063	1.5E-01	3.6E−01
AiHT54	*A. indica*	Si021400m	*S. italica*	Sobic.006G272500.1	*S. bicolor*	540	0.201	0.193	0.204	L	0.009	9.2E-01	9.7E−01
AiHT55	*A. indica*	Si021383m	*S. italica*	Sobic.009G191500.1	*S. bicolor*	1,185	0.311	0.471	0.257	H	2.683	1.0E-01	2.8E−01
AiHT56	*A. indica*	Si006617m	*S. italica*	Sobic.010G033400.1	*S. bicolor*	933	0.330	0.621	0.164	H	14.447	1.4E-04	4.0E−03
AiHT57	*A. indica*	Si007399m	*S. italica*	Sobic.001G067900.1	*S. bicolor*	468	0.009	0.000	0.012	L	0.654	4.2E-01	7.6E−01
AiHT58	*A. indica*	Si005833m	*S. italica*	Sobic.010G178600.1	*S. bicolor*	1,161	0.067	0.082	0.061	H	0.182	6.7E-01	8.1E−01
AiHT59	*A. indica*	Si006000m	*S. italica*	Sobic.005G101200.1	*S. bicolor*	507	0.198	0.000	0.210	L	1.997	1.6E-01	3.5E−01
AiHT60	*A. indica*	Si035951m	*S. italica*	Pavir.Da02389.1	*P. virgatum*	1,119	0.308	0.361	0.288	H	0.507	4.8E-01	7.8E−01
AiHT61	*A. indica*	Sobic.001G187000.1	*S. bicolor*	Si034944m	*S. italica*	1,365	0.294	0.267	0.313	L	0.331	5.6E-01	7.6E−01
AiHT62	*A. indica*	Sobic.001G187100.1	*S. bicolor*	Si035814m	*S. italica*	1,053	0.293	0.221	0.326	L	1.002	3.2E-01	6.0E−01
AiHT63	*A. indica*	Sobic.001G187300.1	*S. bicolor*	Si035427m	*S. italica*	1,224	0.226	0.289	0.154	H	5.178	2.3E-02	1.5E−01
AiHT64	*A. indica*	Sobic.001G415700.1	*S. bicolor*	LOC_Os07g45194.1	*O. sativa*	807	0.675	0.816	0.627	H	0.419	5.2E-01	7.5E−01
AiHT65	*A. indica*	Sobic.002G347900.1	*S. bicolor*	Pavir.Ba02235.1	*P. virgatum*	417	0.084	0.115	0.065	H	0.884	3.5E-01	6.4E−01
AiHT66	*A. indica*	Si029048m	*S. italica*	Sobic.002G349900.1	*S. bicolor*	897	0.257	0.176	0.308	L	2.434	1.2E-01	3.1E−01
AiHT67	*A. indica*	Sobic.002G166200.1	*S. bicolor*	Pavir.Bb01074.1	*P. virgatum*	708	0.148	0.106	0.163	L	0.536	4.6E-01	8.0E−01
AiHT68	*A. indica*	Sobic.006G024000.1	*S. bicolor*	LOC_Os04g12970.1	*O. sativa*	1,341	0.199	0.223	0.193	H	0.201	6.5E-01	8.1E−01
AiHT69	*A. indica*	Sobic.008G025200.1	*S. bicolor*	GRMZM2G536120_T01	*Z. mays*	975	0.433	0.442	0.425	H	0.016	9.0E-01	9.7E−01
AiHT70	*A. indica*	Sobic.010G033600.1	*S. bicolor*	Si006812m	*S. italica*	948	0.217	0.241	0.197	H	0.462	5.0E-01	7.8E−01
AiHT71	*A. indica*	Bradi2g00417.1	*B. distachyon*	LOC_Os02g18670.1	*O. sativa*	1,986	0.248	0.320	0.224	H	2.037	1.5E-01	3.5E−01
AiHT72	*A. indica*	Bradi3g55500.1	*B. distachyon*	Pavir.Gb01821.1	*P. virgatum*	714	0.694	1.172	0.532	H	2.951	8.6E-02	2.5E−01
AiHT73	*A. indica*	Bradi4g31220.1	*B. distachyon*	Sobic.002G214600.1	*S. bicolor*	414	0.263	0.659	0.193	H	4.133	4.2E-02	1.8E−01
AiHT74	*A. indica*	Sobic.003G274800.1	*S. bicolor*	Bradi2g47987.1	*B. distachyon*	939	0.153	0.173	0.145	H	0.234	6.3E-01	7.9E−01
AiHT75	*A. indica*	Sobic.006G052500.1	*S. bicolor*	Bradi5g08230.1	*B. distachyon*	1,332	0.047	0.073	0.038	H	2.589	1.1E-01	2.9E−01
AiHT76	*A. indica*	Bradi5g21658.1	*B. distachyon*	LOC_Os04g52380.1	*O. sativa*	705	0.619	1.548	0.532	H	3.098	7.8E-02	2.5E−01
AiHT77	*A. indica*	Sobic.001G138000.1	*S. bicolor*	Bradi1g12550.2	*B. distachyon*	1,728	0.079	0.102	0.068	H	1.482	2.2E-01	4.5E−01
AiHT78	*A. indica*	Sobic.007G208300.1	*S. bicolor*	LOC_Os08g40110.2	*O. sativa*	1,242	0.143	0.124	0.152	L	0.433	5.1E-01	7.7E−01
AiHT79	*A. indica*	Bradi3g21500.2	*B. distachyon*	Pavir.Ia03658.1	*P. virgatum*	1,290	0.455	0.542	0.428	H	0.524	4.7E-01	7.9E−01
AiHT80	NA	NA	NA	NA	NA	NA	NA	NA	NA	NA	NA	NA	NA
AiHT81-1	*A. indica*	Si022060m	*S. italica*	Sobic.001G152700.1	*S. bicolor*	1,323	0.829	0.572	1.192	L	5.536	1.9E-02	1.4E−01
AiHT81-2	*A. indica*	Bradi1g00410.1	*B. distachyon*	LOC_Os01g56900.1	*O. sativa*	876	0.629	0.437	0.707	L	1.620	2.0E-01	4.2E−01

Table S9 Query gene lists used for SEA analysis by agriGO v1.2

Species	HGT loci	Gene ID	Products	GO ID
M. truncatula	OmHT01	Medtr1g017270	FAD/NAD(P)-binding oxidoreductase family protein	GO:0004506,GO:0050660,GO:0055114,GO:0016021
	OmHT02	Medtr1g060070	Iron-sulphur cluster biosynthesis family protein	
	OmHT03	Medtr3g007750	Ribosomal RNA adenine dimethylase family protein	GO:0000179,GO:0008649,GO:0000154
	OmHT04	Medtr4g080790	electron transfer flavoprotein alpha	
	OmHT05	Medtr4g080800	PDI-like 5-4	GO:0045454
	OmHT06	Medtr4g119840	alanine-tRNA ligases;nucleic acid binding;ligases, forming aminoacyl-tRNA	GO:0000166,GO:0004813,GO:0005524,GO:0006419,GO:0016876,GO:0043039
	OmHT07	Medtr5g093580	co-factor for nitrate, reductase and xanthine dehydrogenase 5	GO:0003824
	OmHT08	Medtr6g004840	embryo defective 3012	
	OmHT09	Medtr8g007390	RNA-binding (RRM/RBD/RNP motifs) family protein	GO:0003676
	OmHT10	Medtr8g446850	UDP-glcnac-adolichol phosphate glcnac-1-p-transferase	GO:0008963,GO:0016021
	OmHT11	Medtr3g048190	Class II aaRS and biotin synthetases superfamily protein	GO:0000166,GO:0004812,GO:0005524,GO:0006418,GO:0004827,GO:0006433,GO:0005737
	OmHT13	Medtr8g075480	S-adenosyl-L-methionine-dependent methyltransferases superfamily protein	
	OmHT14	Medtr7g074850	NAD(P)-binding Rossmann-fold superfamily protein	GO:0003824,GO:0050662,GO:0044237
	OmHT15	Medtr3g052610	RNA helicase family protein	GO:0003676,GO:0005524,GO:0008026,GO:0004386
	OmHT16	Medtr5g064160	Trypsin family protein with PDZ domain	
	OmHT17	Medtr3g048950	Amidase family protein	GO:0016884
	OmHT18	Medtr4g078640	FtsJ-like methyltransferase family protein	GO:0008168,GO:0032259,GO:0006364,GO:0005634
	OmHT19	Medtr4g098640	ubiquitin family protein	
	OmHT20	Medtr7g096260	nucleotide binding;nucleic acid binding;RNA binding	GO:0003676
	OmHT21	Medtr8g099865	fucosyltransferase 1	GO:0008107,GO:0042546,GO:0016020
	OmHT22	Medtr3g460700	S-adenosyl-L-methionine-dependent methyltransferases superfamily protein	
L. japonicus	OmHT02	chr1.CM0010.110.r2.d	NoData	GO:0005198,GO:0016226,GO:0051536
	OmHT03	LjSGA_048522.1	NoData	GO:0000154,GO:0000179,GO:0003723,GO:0005819,GO:0030686
	OmHT05	chr3.CM1543.10.r2.m	NoData	
	OmHT06	LjSGA_011895.2	NoData	GO:0004813,GO:0005524,GO:0005737,GO:0006419
	OmHT07	LjSGA_024515.1	NoData	GO:0001403,GO:0005515,GO:0005737,GO:0006464,GO:0006979,GO:0007114,GO:0007124,GO:0008265,GO:0040035,GO:0042292
	OmHT08	chr2.CM0263	NoData	GO:0005783,GO:0005829,GO:0005886,GO:0005986,GO:0006012,GO:0006464,GO:0006468,GO:0006723,GO:0006979,GO:0008375,GO:0009409,GO:0009414,GO:0009506,
	OmHT10	chr5.CM0278.110.r2.m	NoData	GO:0000271,GO:0003975,GO:0005783,GO:0005886,GO:0008963,GO:0016021
	OmHT11	chr2.CM0177.390.r2.m	NoData	GO:0000166,GO:0004827,GO:0005524,GO:0005737,GO:0005829,GO:0006433,GO:0009506,GO:0016020
	OmHT12	chr3.CM0005.510.r2.m	NoData	GO:0000226,GO:0000910,GO:0005819,GO:0005874,GO:0008017,GO:0009506,GO:0009524,GO:0009574,GO:0022402,GO:0032403,GO:0046785,GO:0055028,GO:0071840
	OmHT15	LjSGA_019513.1	NoData	GO:0006412,GO:0009792,GO:0030686,GO:0031202,GO:0040010,GO:0040011,GO:0042273
	OmHT17	chr6.CM0836.110.r2.d	NoData	GO:0006412,GO:0009507,GO:0009570,GO:0016884
	OmHT18	chr3.CM0091.1190.r2.d	NoData	GO:0001510,GO:0005634,GO:0006364,GO:0008168,GO:0032259
	OmHT19	chr4.LjT10K15.20.r2.m	NoData	GO:0005515
	OmHT20	chr1.LjT23J20.40.r2.m	NoData	GO:0000166,GO:0003676,GO:0005730,GO:1901265
	OmHT21	chr3.LjT45M09.140.r2.d	NoData	
	OmHT22	chr6.LjT111F18.110.r2.m	NoData	GO:0003723,GO:0005730,GO:0005829,GO:0006364,GO:0008283,GO:0008757,GO:0009965,GO:0010015
P. vulgaris	OmHT02	Phvul.001G189600	Iron-sulphur cluster biosynthesis family protein	
	OmHT03	Phvul.008G161800	Ribosomal RNA adenine dimethylase family protein	GO:0000179,GO:0008649,GO:0000154,GO:0008168,GO:0008152
	OmHT04	Phvul.011G139500	electron transfer flavoprotein alpha	
	OmHT04	Phvul.011G139600	electron transfer flavoprotein alpha	
	OmHT05	Phvul.011G139400	PDI-like 5-4	GO:0045454
	OmHT06	Phvul.003G105100	alanine-tRNA ligases;nucleic acid binding;ligases, forming aminoacyl-tRNA	GO:0000166,GO:0004813,GO:0005524,GO:0006419,GO:0005737,GO:0016876,GO:0043039,GO:0006412
	OmHT07	Phvul.008G270700	co-factor for nitrate, reductase and xanthine dehydrogenase 5	GO:0003824
	OmHT08	Phvul.004G006800	embryo defective 3012	
	OmHT09	Phvul.010G044700	RNA-binding (RRM/RBD/RNP motifs) family protein	GO:0003676
	OmHT10	Phvul.007G249500	UDP-glcnac-adolichol phosphate glcnac-1-p-transferase	GO:0008963,GO:0016021
	OmHT11	Phvul.001G218300	Class II aaRS and biotin synthetases superfamily protein	GO:0000166,GO:0004812,GO:0005524,GO:0006418,GO:0005737,GO:0004827,GO:0006433,GO:0006412
	OmHT13	Phvul.003G263600	S-adenosyl-L-methionine-dependent methyltransferases superfamily protei	GO:0008168,GO:0008276,GO:0006479,GO:0005737
	OmHT14	Phvul.008G076500	NAD(P)-binding Rossmann-fold superfamily protein	GO:0016491,GO:0008152,GO:0003854,GO:0016616,GO:0006694,GO:0055114,GO:0003824,GO:0050662,GO:0044237
	OmHT17	Phvul.006G036200	Amidase family protein	GO:0016884
	OmHT18	Phvul.011G044300	FtsJ-like methyltransferase family protein	GO:0003676,GO:0008168,GO:0032259,GO:0006364,GO:0005634,GO:0001510
	OmHT19	Phvul.003G229300	ubiquitin family protein	
	OmHT20	Phvul.001G070900	nucleotide binding;nucleic acid binding;RNA binding	GO:0003676
	OmHT21	Phvul.005G182600	fucosyltransferase 1	GO:0008107,GO:0042546,GO:0016020
	OmHT22	Phvul.006G059200	S-adenosyl-L-methionine-dependent methyltransferases superfamily protein	
G. max	OmHT02	Glyma.19G194700	Iron-sulphur cluster biosynthesis family protein	
	OmHT03	Glyma.06G316500	Ribosomal RNA adenine dimethylase family protein	GO:0000179,GO:0008649,GO:0000154,GO:0008168,GO:0008152
	OmHT04	Glyma.06G270300	electron transfer flavoprotein alpha	
	OmHT05	Glyma.06G270200	PDI-like 5-4	GO:0045454
	OmHT07	Glyma.02G293600	co-factor for nitrate, reductase and xanthine dehydrogenase 5	GO:0003824
	OmHT08	Glyma.05G003500	embryo defective 3012	
	OmHT09	Glyma.03G107700	RNA-binding (RRM/RBD/RNP motifs) family protein	GO:0003676
	OmHT10	Glyma.02G190700	UDP-glcnac-adolichol phosphate glcnac-1-p-transferase	GO:0008963,GO:0016021
	OmHT11	Glyma.19G221400	Class II aaRS and biotin synthetases superfamily protein	GO:0000166,GO:0004812,GO:0005524,GO:0006418,GO:0004827,GO:0006433,GO:0005737
	OmHT12	Glyma.13G189600	microtubule-associated protein 65-5	GO:0008017,GO:0000226,GO:0000910
	OmHT13	Glyma.02G047300	S-adenosyl-L-methionine-dependent methyltransferases superfamily protei	GO:0008168,GO:0008276,GO:0006479,GO:0005737
	OmHT15	Glyma.02G010500	RNA helicase family protein	GO:0003676,GO:0005524,GO:0008026,GO:0004386
	OmHT17	Glyma.08G311400	Amidase family protein	GO:0016884
	OmHT18	Glyma.12G042600	FtsJ-like methyltransferase family protein	GO:0008168,GO:0032259,GO:0006364,GO:0005634
	OmHT19	Glyma.05G113600	ubiquitin family protein	
	OmHT20	Glyma.14G154500	nucleotide binding;nucleic acid binding;RNA binding	GO:0003676
	OmHT21	Glyma.13G370500	fucosyltransferase 1	GO:0008107,GO:0042546,GO:0016020
	OmHT22	Glyma.18G075000	S-adenosyl-L-methionine-dependent methyltransferases superfamily protein	
O. sativa	AiHT02	LOC_Os03g47650	ankyrin repeat family protein	GO:0008150,GO:0005575,GO:0005488,GO:0005515
	AiHT03	LOC_Os10g28320	NAD(P)-linked oxidoreductase superfamily protein	GO:0005886,GO:0003824,GO:0008152,GO:0009536,GO:0055114,GO:0016491
	AiHT07	LOC_Os07g27490	Regulator of chromosome condensation (RCC1) family protein	GO:0005515,GO:0008150,GO:0005575
	AiHT08	LOC_Os07g36830		
	AiHT10	LOC_Os01g55950	Acetamidase/Formamidase family protein	GO:0016787,GO:0005575,GO:0008152,GO:0016811
	AiHT14	LOC_Os02g53520	P-loop containing nucleoside triphosphate hydrolases superfamily protein	GO:0000166,GO:0009987,GO:0008152,GO:0003774,GO:0003777,GO:0005524,GO:0007018
	AiHT15	LOC_Os02g56565	endoribonucleases	GO:0008152,GO:0005623,GO:0008150,GO:0004518
	AiHT18	LOC_Os04g08350	cysteine synthase C1	GO:0003824,GO:0009653,GO:0030154,GO:0007275,GO:0008152,GO:0006950,GO:0009056,GO:0009987,GO:0005488,GO:0009058,GO:0005739,GO:0009536,GO:0030170
	AiHT21	LOC_Os09g33450	DHHC-type zinc finger family protein	GO:0008150,GO:0005488,GO:0005886,GO:0008270
	AiHT23	LOC_Os03g37984	Amino acid permease family protein	GO:0009987,GO:0006810,GO:0005215,GO:0055085,GO:0016020,GO:0015171,GO:0003333
	AiHT25	LOC_Os12g09000	thiamin biosynthesis protein, putative	GO:0016301,GO:0009058,GO:0009987,GO:0008152,GO:0016740,GO:0009536,GO:0004789,GO:0009228
	AiHT26	LOC_Os05g01360	Vacuolar sorting protein 39	GO:0008150,GO:0008152,GO:0005829,GO:0030234,GO:0006886,GO:0016192,GO:0005083
	AiHT34	LOC_Os06g49060	Protein of unknown function, DUF547	GO:0006139,GO:0005773,GO:0003700,GO:0009058
	AiHT35	LOC_Os06g49080	Zinc finger C-x8-C-x5-C-x3-H type family protein	GO:0005635,GO:0005515,GO:0008150,GO:0003676,GO:0008270
	AiHT36	LOC_Os06g50930	Rhodanese/Cell cycle control phosphatase superfamily protein	GO:0008150,GO:0006950,GO:0003674,GO:0009628,GO:0009719,GO:0009536
	AiHT37	LOC_Os06g50940	GDSL-like Lipase/Acylhydrolase superfamily protein	GO:0016787,GO:0006629,GO:0005623,GO:0008152,GO:0016788
	AiHT38	LOC_Os06g51010	Arabidopsis protein of unknown function (DUF241)	GO:0003674,GO:0009536
	AiHT39	LOC_Os06g51070	NAC domain containing protein 36	GO:0007275,GO:0009653,GO:0016043,GO:0000003,GO:0005575,GO:0008150,GO:0009791,GO:0003700,GO:0009058,GO:0006139,GO:0003677,GO:0006355
	AiHT40	LOC_Os06g51084	starch branching enzyme 2.2	GO:0009058,GO:0009987,GO:0005975,GO:0008152,GO:0016740,GO:0009536,GO:0003824,GO:0043169,GO:0004553
	AiHT41	LOC_Os06g51100	Protein of unknown function (DUF300)	GO:0008150
	AiHT43	LOC_Os06g51150	catalase 2	GO:0003824,GO:0019725,GO:0008152,GO:0005777,GO:0004096,GO:0020037,GO:0055114,GO:0006979,GO:0005506
	AiHT44	LOC_Os01g08770	Transducin/WD40 repeat-like superfamily protein	GO:0005515,GO:0008150,GO:0005622,GO:0000166
	AiHT45	LOC_Os09g15480	Protein of unknown function (DUF833)	GO:0003674,GO:0008150
	AiHT48	LOC_Os06g01980	cell division cycle 48C	GO:0016787,GO:0000166,GO:0008152,GO:0009056,GO:0006139,GO:0000003,GO:0009791,GO:0009790,GO:0005524,GO:0009378,GO:0006281,GO:0006310,GO:0016887
	AiHT49	LOC_Os12g28270		GO:0016787
	AiHT54	LOC_Os04g58700	Leucine-rich repeat protein kinase family protein	GO:0005886,GO:0006464,GO:0016301,GO:0000166,GO:0007165,GO:0004672,GO:0005524,GO:0006468,GO:0005515
	AiHT59	LOC_Os11g11000	Protein kinase superfamily protein	GO:0009536,GO:0000166,GO:0016301,GO:0006464
	AiHT61	LOC_Os03g05780	AMP-dependent synthetase and ligase family protein	GO:0006629,GO:0005777,GO:0008152,GO:0003824,GO:0009058,GO:0009987,GO:0016740
	AiHT62	LOC_Os03g37100	DTW domain-containing protein	GO:0008150,GO:0003674
	AiHT64	LOC_Os07g45195		
	AiHT68	LOC_Os04g12970	UDP-glucosyltransferase 74F2	GO:0016740,GO:0008152,GO:0016758
	AiHT71	LOC_Os02g18670	multidrug resistance-associated protein 3	GO:0055085,GO:0016021
	AiHT74	LOC_Os01g51390	Insulinase (Peptidase family M16) protein	GO:0016020,GO:0009536,GO:0005739,GO:0005488,GO:0005618,GO:0005730,GO:0019538,GO:0005773,GO:0016787,GO:0004222,GO:0006508,GO:0008270
	AiHT76	LOC_Os04g52380	methyl-CPG-binding domain protein 13	GO:0008150,GO:0000166,GO:0003677,GO:0005634
	AiHT77	LOC_Os03g47610	thiaminC	GO:0009536,GO:0009991,GO:0009987,GO:0003824,GO:0008152,GO:0005488,GO:0009607,GO:0009058,GO:0016787,GO:0009605,GO:0009228
	AiHT78	LOC_Os08g40110	Peptidase M20/M25/M40 family protein	GO:0008152,GO:0016787,GO:0008150,GO:0005773
	AiHT81	LOC_Os01g56900		
P. virgatum	AiHT01	Pavir.J11036		
	AiHT02	Pavir.Ib04221	ankyrin repeat family protein	GO:0005515
	AiHT03	Pavir.Ib03389	NAD(P)-linked oxidoreductase superfamily protein	
	AiHT04	Pavir.J11055		
	AiHT05	Pavir.J19441	tubulin alpha-5	GO:0051258,GO:0043234,GO:0003924,GO:0005525,GO:0006184
	AiHT07	Pavir.J39002	Regulator of chromosome condensation (RCC1) family protein	
	AiHT08	Pavir.Ba00786		
	AiHT09	Pavir.Bb03116	Putative methyltransferase family protein	
	AiHT10	Pavir.Ea02809	Acetamidase/Formamidase family protein	GO:0016811,GO:0008152
	AiHT11	Pavir.Ea04104	SNARE-like superfamily protein	GO:0016192,GO:0016021
	AiHT12	Pavir.Aa01524	phosphoinositide binding	GO:0046872
	AiHT13	Pavir.Aa00431	Rubisco methyltransferase family protein	GO:0005515
	AiHT14	Pavir.Aa00394	P-loop containing nucleoside triphosphate hydrolases superfamily protein	GO:0003777,GO:0005524,GO:0007018
	AiHT15	Pavir.Aa00141	endoribonucleases	
	AiHT16	Pavir.Ha00529	Major facilitator superfamily protein	GO:0005215,GO:0006857,GO:0016020
	AiHT17	Pavir.Ha00750	DNA binding;ATP binding	
	AiHT18	Pavir.J33604	cysteine synthase C1	GO:0003824,GO:0030170,GO:0008152
	AiHT19	Pavir.Ga00840	GRAM domain-containing protein / ABA-responsive protein-related	
	AiHT20	Pavir.J36841	diacylglycerol kinase 5	GO:0004143,GO:0007205
	AiHT21	Pavir.Fa02137	DHHC-type zinc finger family protein	GO:0008270
	AiHT22	Pavir.Ia00629	ribosomal protein S10	GO:0003735,GO:0006412,GO:0005622,GO:0005840
	AiHT23	Pavir.Fa01869	Amino acid permease family protein	GO:0006810,GO:0055085,GO:0016020
	AiHT24	Pavir.Fa01563		
	AiHT25	Pavir.Ca00302	thiamin biosynthesis protein, putative	GO:0004789,GO:0009228
	AiHT26	Pavir.J01171	Vacuolar sorting protein 39	GO:0006886,GO:0016192,GO:0005083
	AiHT27	Pavir.Ab01636	RNI-like superfamily protein	
	AiHT28	Pavir.Aa03584	Alg9-like mannosyltransferase family	GO:0016757,GO:0006506,GO:0031227
	AiHT30	Pavir.Aa02139		
	AiHT31	Pavir.Da01336	kinesin like protein for actin based chloroplast movement 2	
	AiHT33	Pavir.Ea02508	BTB-POZ and MATH domain 1	GO:0005515
	AiHT34	Pavir.Db00226	Protein of unknown function, DUF547	
	AiHT35	Pavir.Db00225	Zinc finger C-x8-C-x5-C-x3-H type family protein	GO:0003676,GO:0008270
	AiHT36	Pavir.J05061	Rhodanese/Cell cycle control phosphatase superfamily protein	
	AiHT37	Pavir.Db00017	GDSL-like Lipase/Acylhydrolase superfamily protein	GO:0016788,GO:0006629
	AiHT38	Pavir.Db00024	Arabidopsis protein of unknown function (DUF241)	
	AiHT39	Pavir.J01198	NAC domain containing protein 36	GO:0003677,GO:0006355
	AiHT40	Pavir.J01199	starch branching enzyme 2.2	GO:0003824,GO:0043169,GO:0005975,GO:0004553

	AiHT41	Pavir.J01200	Protein of unknown function (DUF300)	
	AiHT42	Pavir.Db00031	Cyclin B2;3	GO:0005634
	AiHT43	Pavir.Db00032	catalase 2	GO:0004096,GO:0020037,GO:0055114
	AiHT44	Pavir.Ba02231	Transducin/WD40 repeat-like superfamily protein	GO:0005515
	AiHT45	Pavir.J17263	Protein of unknown function (DUF833)	
	AiHT46	Pavir.J04920	high mobility group B3	GO:0005515
	AiHT47	Pavir.Db00317	titan9	
	AiHT48	Pavir.Ca00597	cell division cycle 48C	GO:0005524,GO:0009378,GO:0006281,GO:0006310
	AiHT49	Pavir.Cb00776		GO:0016787
	AiHT50	Pavir.J11740	Peroxisomal membrane 22 kDa (Mpv17/PMP22) family protein	GO:0016021
	AiHT51	Pavir.Db00653	thiazole biosynthetic enzyme, chloroplast (ARA6) (THI1) (THI4)	GO:0009055,GO:0016491,GO:0055114
	AiHT52	Pavir.Bb03146	splicing factor Prp18 family protein	GO:0008380,GO:0005681
	AiHT53	Pavir.Bb02626	NAC domain containing protein 57	GO:0003677,GO:0006355
	AiHT54	Pavir.Ga00525	Leucine-rich repeat protein kinase family protein	GO:0004672,GO:0005524,GO:0006468,GO:0005515
	AiHT55	Pavir.J05644	Pseudouridine synthase family protein	GO:0003723,GO:0009982,GO:0001522,GO:0009451
	AiHT56	Pavir.Db02243	PRP38 family protein	
	AiHT57	Pavir.J06884	associated protein 19	
	AiHT58	Pavir.Da00644	Coatomer, beta¥' subunit	GO:0005515,GO:0005198,GO:0006886,GO:0016192,GO:0030117
	AiHT59	Pavir.J16568	Protein kinase superfamily protein	
	AiHT60	Pavir.J11373	F-box family protein with a domain of unknown function (DUF295)	
	AiHT61	Pavir.J37841	AMP-dependent synthetase and ligase family protein	GO:0003824,GO:0008152
	AiHT62	Pavir.Ia01375	DTW domain-containing protein	
	AiHT63	Pavir.Ib03805	molybdate transporter 1	
	AiHT64	Pavir.J06640		
	AiHT65	Pavir.Ba02235	O-fucosyltransferase family protein	
	AiHT66	Pavir.Bb03186	translocon at the outer envelope membrane of chloroplasts 75-III	GO:0019867
	AiHT67	Pavir.Bb01074	wall associated kinase-like 6	GO:0004672,GO:0005524,GO:0006468,GO:0005509
	AiHT68	Pavir.Ga02622	UDP-glucosyltransferase 74F2	GO:0016758,GO:0008152
	AiHT69	Pavir.Hb01859	ankyrin repeat family protein	GO:0005515
	AiHT70	Pavir.Da02235	BRCT domain-containing DNA repair protein	GO:0005622
	AiHT71	Pavir.Bb00325	multidrug resistance-associated protein 3	GO:0005524,GO:0016887,GO:0042626,GO:0006810,GO:0055085,GO:0016021
	AiHT72	Pavir.Gb01821		
	AiHT73	Pavir.Ba01868	chaperonin 20	GO:0006457,GO:0005737
	AiHT74	Pavir.J40338	Insulinase (Peptidase family M16) protein	GO:0004222,GO:0006508,GO:0008270
	AiHT75	Pavir.Ga01944	2-oxoglutarate dehydrogenase, E1 component	GO:0016624,GO:0008152
	AiHT76	Pavir.J26297	methyl-CPG-binding domain protein 13	GO:0003677,GO:0005634
	AiHT77	Pavir.J26403	thiaminC	GO:0009228
	AiHT78	Pavir.Fa00445	Peptidase M20/M25/M40 family protein	GO:0016787,GO:0008152,GO:0008233,GO:0006508
	AiHT79	Pavir.Ia03658	Flavodoxin family protein	GO:0010181,GO:0016491,GO:0055114
S. italica	AiHT01	Si036782m.g		
	AiHT03	Si036472m.g	NAD(P)-linked oxidoreductase superfamily protein	GO:0055114,GO:0016491
	AiHT04	Si035364m.g		
	AiHT05	Si035680m.g	tubulin alpha-5	GO:0051258,GO:0043234,GO:0003924,GO:0005525,GO:0006184,GO:0007017,GO:0005874
	AiHT07	Si029674m.g	Regulator of chromosome condensation (RCC1) family protein	
	AiHT09	Si030798m.g	Putative methyltransferase family protein	
	AiHT10	Si001397m.g	Acetamidase/Formamidase family protein	GO:0016811,GO:0008152
	AiHT12	Si016118m.g	phosphoinositide binding	GO:0005515,GO:0046872
	AiHT13	Si017054m.g	Rubisco methyltransferase family protein	GO:0005515
	AiHT14	Si016200m.g	P-loop containing nucleoside triphosphate hydrolases superfamily protein	GO:0003777,GO:0005524,GO:0007018
	AiHT15	Si016449m.g	endoribonucleases	
	AiHT16	Si026160m.g	Major facilitator superfamily protein	GO:0005215,GO:0006857,GO:0016020
	AiHT17	Si027633m.g	DNA binding;ATP binding	
	AiHT18	Si010368m.g	cysteine synthase C1	GO:0003824,GO:0030170,GO:0008152
	AiHT19	Si011043m.g	GRAM domain-containing protein / ABA-responsive protein-related	
	AiHT20	Si009926m.g	diacylglycerol kinase 5	GO:0004143,GO:0007205
	AiHT21	Si013846m.g	DHHC-type zinc finger family protein	GO:0008270
	AiHT23	Si013633m.g	Amino acid permease family protein	GO:0055085,GO:0016020,GO:0006810,GO:0015171,GO:0003333
	AiHT24	Si013766m.g		
	AiHT25	Si009759m.g	thiamin biosynthesis protein, putative	GO:0004789,GO:0009228
	AiHT26	Si021122m.g	Vacuolar sorting protein 39	GO:0006886,GO:0016192,GO:0005083
	AiHT27	Si017739m.g	RNI-like superfamily protein	GO:0005515
	AiHT28	Si016829m.g	Alg9-like mannosyltransferase family	GO:0016757,GO:0006506,GO:0031227
	AiHT30	Si017662m.g		
	AiHT31	Si005702m.g	kinesin like protein for actin based chloroplast movement 1	GO:0003777,GO:0005524,GO:0007018
	AiHT34	Si006225m.g	Protein of unknown function, DUF547	
	AiHT35	Si006602m.g	Zinc finger C-x8-C-x5-C-x3-H type family protein	GO:0003676,GO:0008270
	AiHT36	Si008168m.g	Rhodanese/Cell cycle control phosphatase superfamily protein	
	AiHT38	Si007045m.g	Arabidopsis protein of unknown function (DUF241)	
	AiHT39	Si006994m.g	NAC domain containing protein 36	GO:0003677,GO:0006355
	AiHT40	Si005911m.g	starch branching enzyme 2.2	GO:0003824,GO:0043169,GO:0005975,GO:0004553
	AiHT41	Si006336m.g	Protein of unknown function (DUF300)	
	AiHT42	Si006509m.g	Cyclin B2;3	GO:0005634
	AiHT43	Si006189m.g	catalase 2	GO:0004096,GO:0020037,GO:0055114,GO:0006979,GO:0005506
	AiHT44	Si000998m.g	Transducin/WD40 repeat-like superfamily protein	GO:0005515
	AiHT45	Si007094m.g	Protein of unknown function (DUF833)	
	AiHT46	Si007288m.g	high mobility group B2	GO:0005515,GO:0003677
	AiHT47	Si002590m.g	titan9	
	AiHT48	Si040216m.g	cell division cycle 48C	GO:0005524,GO:0016887
	AiHT49	Si021935m.g		GO:0016787
	AiHT50	Si018070m.g	Peroxisomal membrane 22 kDa (Mpv17/PMP22) family protein	GO:0016021
	AiHT51	Si030346m.g	thiazole biosynthetic enzyme, chloroplast (ARA6) (THI1) (THI4)	
	AiHT52	Si029857m.g	splicing factor Prp18 family protein	GO:0008380,GO:0005681
	AiHT53	Si032702m.g	NAC domain containing protein 57	GO:0003677,GO:0006355
	AiHT54	Si021400m.g	Leucine-rich repeat protein kinase family protein	GO:0005515,GO:0006468,GO:0004672
	AiHT55	Si021383m.g	Pseudouridine synthase family protein	GO:0003723,GO:0009982,GO:0001522,GO:0009451
	AiHT56	Si006617m.g	PRP38 family protein	
	AiHT57	Si007399m.g	associated protein 19	GO:0015031,GO:0008565
	AiHT58	Si005833m.g	Coatomer, beta¥' subunit	GO:0005198,GO:0006886,GO:0016192,GO:0030117,GO:0005515
	AiHT59	Si006000m.g	Protein kinase superfamily protein	
	AiHT60	Si035951m.g	Protein of unknown function (DUF295)	GO:0005515
	AiHT61	Si034944m.g	AMP-dependent synthetase and ligase family protein	GO:0003824,GO:0008152
	AiHT62	Si035814m.g	DTW domain-containing protein	
	AiHT63	Si035427m.g	molybdate transporter 1	
	AiHT64	Si018136m.g		
	AiHT66	Si029048m.g	translocon at the outer envelope membrane of chloroplasts 75-III	GO:0019867
	AiHT68	Si010049m.g	Uridine diphosphate glycosyltransferase 74E2	GO:0016758,GO:0008152
	AiHT70	Si006812m.g	BRCT domain-containing DNA repair protein	GO:0005622
	AiHT71	Si032963m.g	multidrug resistance-associated protein 3	GO:0005524,GO:0016887,GO:0042626,GO:0006810,GO:0055085,GO:0016021
	AiHT73	Si030910m.g	chaperonin 20	GO:0006457,GO:0005737
	AiHT74	Si001093m.g	Insulinase (Peptidase family M16) protein	GO:0004222,GO:0006508,GO:0008270
	AiHT75	Si009245m.g	2-oxoglutarate dehydrogenase, E1 component	GO:0016624,GO:0008152,GO:0030976,GO:0006096,GO:0004591
	AiHT76	Si011769m.g	methyl-CPG-binding domain protein 13	GO:0003677,GO:0005634
	AiHT77	Si034631m.g	thiaminC	GO:0009228
	AiHT78	Si013712m.g	Peptidase M20/M25/M40 family protein	GO:0016787,GO:0008152
	AiHT81	Si029853m.g		
S. bicolor	AiHT01	Sobic.001G053000		
	AiHT02	Sobic.001G137400	ankyrin repeat family protein	GO:0005515
	AiHT02	Sobic.001G137500	ankyrin repeat family protein	GO:0005515
	AiHT03	Sobic.001G242800	NAD(P)-linked oxidoreductase superfamily protein	
	AiHT04	Sobic.001G358600		
	AiHT05	Sobic.001G453700	tubulin alpha-5	GO:0051258,GO:0043234,GO:0003924,GO:0005525,GO:0006184
	AiHT06	Sobic.001G501300	ATPase, AAA-type, CDC48 protein	GO:0005524,GO:0009378,GO:0006281,GO:0006310,GO:0016887
	AiHT07	Sobic.002G128200	Regulator of chromosome condensation (RCC1) family protein	
	AiHT08	Sobic.002G335400		
	AiHT09	Sobic.002G345000	Putative methyltransferase family protein	
	AiHT10	Sobic.003G306700	Acetamidase/Formamidase family protein	GO:0016811,GO:0008152
	AiHT11	Sobic.003G438200	SNARE-like superfamily protein	GO:0016192,GO:0016021
	AiHT12	Sobic.003G438400	phosphoinositide binding	GO:0046872
	AiHT13	Sobic.004G247800	Rubisco methyltransferase family protein	GO:0005515
	AiHT14	Sobic.004G315700	P-loop containing nucleoside triphosphate hydrolases superfamily protein	GO:0003777,GO:0005524,GO:0007018
	AiHT15	Sobic.004G339100	endoribonucleases	
	AiHT16	Sobic.005G107500	nitrate transporter 1:2	GO:0005215,GO:0006857,GO:0016020
	AiHT17	Sobic.005G123900	DNA binding;ATP binding	
	AiHT18	Sobic.006G016900	cysteine synthase C1	GO:0003824,GO:0030170,GO:0008152
	AiHT19	Sobic.006G154500	GRAM domain-containing protein / ABA-responsive protein-related	
	AiHT20	Sobic.006G230400	diacylglycerol kinase 5	GO:0004143,GO:0007205
	AiHT21	Sobic.007G002600	DHHC-type zinc finger family protein	GO:0008270
	AiHT22	Sobic.007G019100	ribosomal protein S10	GO:0003735,GO:0006412,GO:0005622,GO:0005840
	AiHT23	Sobic.007G053100	Amino acid permease family protein	GO:0006810,GO:0055085,GO:0016020
	AiHT24	Sobic.007G119400		
	AiHT25	Sobic.008G067100	thiamin biosynthesis protein, putative	GO:0004789,GO:0009228
	AiHT26	Sobic.009G002300	Vacuolar sorting protein 39	GO:0006886,GO:0016192,GO:0005083
	AiHT27	Sobic.009G034400	RNI-like superfamily protein	
	AiHT28	Sobic.009G194000	Alg9-like mannosyltransferase family	GO:0016757,GO:0006506,GO:0031227
	AiHT29	Sobic.010G004200	Nodulin MtN21 /EamA-like transporter family protein	GO:0016020
	AiHT30	Sobic.010G062000		
	AiHT31	Sobic.010G085100	kinesin like protein for actin based chloroplast movement 2	GO:0003777,GO:0005524,GO:0007018
	AiHT32	Sobic.010G222100	BTB-POZ and MATH domain 1	GO:0005515
	AiHT33	Sobic.010G222400	BTB-POZ and MATH domain 1	GO:0005515
	AiHT34	Sobic.010G254700	Protein of unknown function, DUF547	
	AiHT35	Sobic.010G254900	Zinc finger C-x8-C-x5-C-x3-H type family protein	GO:0003676,GO:0008270
	AiHT36	Sobic.010G270300	Rhodanese/Cell cycle control phosphatase superfamily protein	
	AiHT37	Sobic.010G270700	GDSL-like Lipase/Acylhydrolase superfamily protein	GO:0016788,GO:0006629
	AiHT38	Sobic.010G271400	Arabidopsis protein of unknown function (DUF241)	
	AiHT39	Sobic.010G273700	NAC domain containing protein 36	GO:0003677,GO:0006355
	AiHT40	Sobic.010G273800	starch branching enzyme 2.2	GO:0003824,GO:0043169,GO:0005975,GO:0004553
	AiHT41	Sobic.010G273900	Protein of unknown function (DUF300)	
	AiHT42	Sobic.010G274000	Cyclin B2;3	GO:0005634
	AiHT43	Sobic.010G274500	catalase 2	GO:0004096,GO:0020037,GO:0055114
	AiHT44	Sobic.010G274600	Transducin/WD40 repeat-like superfamily protein	GO:0005515

	AiHT45	Sobic.010G275100	Protein of unknown function (DUF833)	
	AiHT46	Sobic.010G275200	high mobility group B3	GO:0005515
	AiHT47	Sobic.003G127500	titan9	
	AiHT49	Sobic.K002400		GO:0016787
	AiHT50	Sobic.004G097200	Peroxisomal membrane 22 kDa (Mpv17/PMP22) family protein	GO:0016021
	AiHT51	Sobic.003G191000	thiazole biosynthetic enzyme, chloroplast (ARA6) (THI1) (THI4)	GO:0009055,GO:0016491,GO:0055114
	AiHT52	Sobic.002G346900	splicing factor Prp18 family protein	GO:0008380,GO:0005681
	AiHT53	Sobic.002G290900	NAC domain containing protein 57	GO:0003677,GO:0006355
	AiHT54	Sobic.006G272500	Leucine-rich repeat protein kinase family protein	GO:0004672,GO:0005524,GO:0006468,GO:0005515
	AiHT55	Sobic.009G191500	Pseudouridine synthase family protein	GO:0003723,GO:0009982,GO:0001522,GO:0009451
	AiHT56	Sobic.010G033400	PRP38 family protein	
	AiHT57	Sobic.001G067900	associated protein 19	
	AiHT58	Sobic.010G178600	Coatomer, beta¥' subunit	GO:0005515,GO:0005198,GO:0006886,GO:0016192,GO:0030117
	AiHT59	Sobic.005G101200	Protein kinase superfamily protein	
	AiHT61	Sobic.001G187000	AMP-dependent synthetase and ligase family protein	GO:0003824,GO:0008152
	AiHT62	Sobic.001G187100	DTW domain-containing protein	
	AiHT63	Sobic.001G187300	molybdate transporter 1	
	AiHT64	Sobic.001G415700		
	AiHT65	Sobic.002G347900	O-fucosyltransferase family protein	
	AiHT66	Sobic.002G349900	translocon at the outer envelope membrane of chloroplasts 75-III	GO:0019867
	AiHT67	Sobic.002G166200	wall associated kinase-like 1	GO:0004672,GO:0005524,GO:0006468,GO:0005509
	AiHT68	Sobic.006G024000	UDP-glucosyltransferase 74F2	GO:0016758,GO:0008152
	AiHT69	Sobic.008G025200	ankyrin repeat family protein	GO:0005515
	AiHT70	Sobic.010G033600	BRCT domain-containing DNA repair protein	GO:0005622
	AiHT73	Sobic.002G214600	chaperonin 20	GO:0006457,GO:0005737
	AiHT74	Sobic.003G274800	Insulinase (Peptidase family M16) protein	GO:0004222,GO:0006508,GO:0008270
	AiHT75	Sobic.006G052500	2-oxoglutarate dehydrogenase, E1 component	GO:0016624,GO:0008152
	AiHT77	Sobic.001G138000	thiaminC	GO:0009228
	AiHT78	Sobic.007G208300	Peptidase M20/M25/M40 family protein	GO:0016787,GO:0008152,GO:0008233,GO:0006508
	AiHT80	Sobic.004G328500		
	AiHT81	Sobic.001G152700		
	AiHT81	Sobic.003G368800		
Z. mays	AiHT03	GRMZM5G813143	NAD(P)-linked oxidoreductase superfamily protein	
	AiHT04	GRMZM2G063498		
	AiHT05	GRMZM2G051782	tubulin alpha-5	GO:0003924,GO:0005525,GO:0006184,GO:0051258,GO:0043234
	AiHT06	GRMZM2G063060	ATPase, AAA-type, CDC48 protein	GO:0005524
	AiHT07	GRMZM2G095562	Regulator of chromosome condensation (RCC1) family protein	
	AiHT08	GRMZM2G008528		
	AiHT09	GRMZM2G081888	Putative methyltransferase family protein	
	AiHT10	GRMZM2G424857	Acetamidase/Formamidase family protein	GO:0016811,GO:0008152
	AiHT11	GRMZM2G168519	SNARE-like superfamily protein	
	AiHT12	GRMZM2G101523	phosphoinositide binding	GO:0046872
	AiHT13	GRMZM2G063316	Rubisco methyltransferase family protein	GO:0005515
	AiHT14	GRMZM2G385925	P-loop containing nucleoside triphosphate hydrolases superfamily protein	GO:0003777,GO:0005524,GO:0008017,GO:0007018
	AiHT15	GRMZM2G150912	endoribonucleases	
	AiHT16	GRMZM2G034389	peptide transporter 2	GO:0005215,GO:0006810,GO:0016020
	AiHT19	GRMZM2G114153	GRAM domain-containing protein / ABA-responsive protein-related	
	AiHT20	GRMZM2G106578	diacylglycerol kinase 5	GO:0004143,GO:0007205
	AiHT21	GRMZM2G163717	DHHC-type zinc finger family protein	GO:0008270
	AiHT22	GRMZM2G095511	ribosomal protein S10	
	AiHT23	GRMZM2G078292	Amino acid permease family protein	GO:0006810,GO:0055085,GO:0016020
	AiHT24	GRMZM2G167694		
	AiHT25	GRMZM2G401934	thiamin biosynthesis protein, putative	GO:0004789,GO:0009228
	AiHT27	GRMZM2G002427	RNI-like superfamily protein	
	AiHT28	GRMZM2G164175	Alg9-like mannosyltransferase family	GO:0016757
	AiHT29	GRMZM2G135044	Nodulin MtN21 /EamA-like transporter family protein	GO:0016020
	AiHT30	GRMZM2G000713		
	AiHT31	GRMZM2G017257	kinesin like protein for actin based chloroplast movement 1	GO:0003777,GO:0005524,GO:0008017,GO:0007018
	AiHT32	GRMZM2G009724	BTB-POZ and MATH domain 1	GO:0005515
	AiHT33	GRMZM2G103251	BTB-POZ and MATH domain 2	GO:0005515
	AiHT34	GRMZM2G089132	Protein of unknown function, DUF547	
	AiHT35	GRMZM2G089050	Zinc finger C-x8-C-x5-C-x3-H type family protein	GO:0046872
	AiHT36	GRMZM2G048085	Rhodanese/Cell cycle control phosphatase superfamily protein	
	AiHT37	GRMZM2G048458	GDSL-like Lipase/Acylhydrolase superfamily protein	GO:0016788,GO:0006629
	AiHT39	GRMZM2G074358	NAC domain containing protein 36	GO:0003677,GO:0006355
	AiHT40	GRMZM2G088753	starch branching enzyme 2.2	GO:0003824,GO:0043169,GO:0005975,GO:0004553
	AiHT41	GRMZM2G088737	Protein of unknown function (DUF300)	
	AiHT42	GRMZM2G073671	Cyclin B2;3	GO:0005634
	AiHT43	GRMZM2G088212	catalase 2	GO:0004096,GO:0020037,GO:0055114
	AiHT44	GRMZM2G096051	Transducin/WD40 repeat-like superfamily protein	GO:0005515
	AiHT45	GRMZM2G037284	Protein of unknown function (DUF833)	
	AiHT46	GRMZM5G834758	high mobility group B3	
	AiHT47	GRMZM2G007258	titan9	
	AiHT48	GRMZM2G082131	cell division cycle 48C	GO:0005524
	AiHT49	GRMZM2G319465		GO:0016787
	AiHT50	GRMZM2G155220	Peroxisomal membrane 22 kDa (Mpv17/PMP22) family protein	GO:0016021
	AiHT51	GRMZM2G074097	thiazole biosynthetic enzyme, chloroplast (ARA6) (THI1) (THI4)	
	AiHT52	GRMZM2G046916	splicing factor Prp18 family protein	GO:0008380,GO:0005681
	AiHT53	GRMZM5G885329	NAC domain containing protein 57	GO:0003677,GO:0006355
	AiHT54	GRMZM2G119759	Leucine-rich repeat protein kinase family protein	GO:0005515,GO:0004672,GO:0006468
	AiHT55	GRMZM2G096553	Pseudouridine synthase family protein	GO:0003723,GO:0009982,GO:0001522,GO:0009451
	AiHT56	GRMZM2G173693	PRP38 family protein	
	AiHT57	GRMZM2G361220	associated protein 19	
	AiHT58	GRMZM2G443953	Coatomer, beta¥' subunit	GO:0005515,GO:0005198,GO:0006886,GO:0016192,GO:0030117
	AiHT59	GRMZM2G377115	Protein kinase superfamily protein	
	AiHT61	GRMZM2G174574		
	AiHT62	GRMZM2G090642	DTW domain-containing protein	
	AiHT65	GRMZM2G030713	O-fucosyltransferase family protein	
	AiHT68	GRMZM2G063042	UDP-glucosyltransferase 74F2	GO:0016758,GO:0008152
	AiHT69	GRMZM2G536120	ankyrin repeat family protein	GO:0005515
	AiHT71	GRMZM5G832772	multidrug resistance-associated protein 3	GO:0005524,GO:0016887,GO:0042626,GO:0006810,GO:0055085,GO:0016021
	AiHT74	GRMZM5G827505	Insulinase (Peptidase family M16) protein	
	AiHT75	GRMZM2G151041	2-oxoglutarate dehydrogenase, E1 component	GO:0016624,GO:0008152
	AiHT76	GRMZM2G119802	methyl-CPG-binding domain protein 13	GO:0003677,GO:0005634
	AiHT77	GRMZM2G027663	thiaminC	GO:0051536,GO:0009228
	AiHT78	GRMZM2G088627	Peptidase M20/M25/M40 family protein	GO:0016787,GO:0008152

We used "Supported species" as query list types and "Suggested background" as bg/ref types for SEA analyses.
We selected
"Medicago truncatula V4.0" and "Medicago genome locus(V4.0)" for *M. truncatula*
"Lotus japonicus" and "Lotus japonicus (Kazusa)" for *L. japonicus*
"Phaseolus vulgaris V1.0" and "Soybean genome locus (phytozome)" for *P. vulgaris*
"Glycine max Wm82.a2.v1" and "Glycine max Wm82.a2.v1" for *G. max*
"Rice MSU7.0 nonTE" and "Rice MSU7.0 nonTE transcript ID" for *O. sativa*
"Panicum virgatum v1.1" and "Panicum virgatum gene ID" for *P. virgatum*
"Setaria italica v2.1" and "Setaria italica v2.1" for *S. italica*
"Sorghum bicolorV2.1" and "Sorghum bicolorV2.1" for *S. bicolor*
"Zea mays AGPv3.30" and "Zea mays AGPv3.30" for *Z. mays*
as query list types and bg/ref types, respectively.

Table S10 **Summary of results of the SEA (Singular Enrichment Analysis) analysis using agriGO v1.2**

Species	GO_acc (GO ID)	term_type*1	Term	queryitem *2	querytotal	bgitem	bgtotal	pvalue *3	FDR *4	entries	HGT loci correspomding to entries
M. truncatula	GO:0034660	P	ncRNA metabolic process	4	14	107	18883	1.10E-06	0.00023	// Medtr3g048190 // Medtr3g460700 // Medtr4g119840 // Medtr3g007750	// OmHT11 // OmHT22 // OmHT06 // OmHT03
	GO:0006364	P	rRNA processing	2	14	18	18883	9.60E-05	0.0069	// Medtr3g460700 // Medtr3g007750	// OmHT22 // OmHT03
	GO:0016072	P	rRNA metabolic process	2	14	18	18883	9.60E-05	0.0069	// Medtr3g460700 // Medtr3g007750	// OmHT22 // OmHT03
	GO:0042254	P	ribosome biogenesis	2	14	31	18883	0.00027	0.011	// Medtr3g460700 // Medtr3g007750	// OmHT22 // OmHT03
	GO:0022613	P	ribonucleoprotein complex biogenesis	2	14	31	18883	0.00027	0.011	// Medtr3g460700 // Medtr3g007750	// OmHT22 // OmHT03
	GO:0034470	P	ncRNA processing	2	14	42	18883	0.00047	0.017	// Medtr3g460700 // Medtr3g007750	// OmHT22 // OmHT03
	GO:0043038	P	amino acid activation	2	14	66	18883	0.0011	0.027	// Medtr3g048190 // Medtr4g119840	// OmHT11 // OmHT06
	GO:0043039	P	tRNA aminoacylation	2	14	66	18883	0.0011	0.027	// Medtr3g048190 // Medtr4g119840	// OmHT11 // OmHT06
	GO:0006418	P	tRNA aminoacylation for protein translation	2	14	66	18883	0.0011	0.027	// Medtr3g048190 // Medtr4g119840	// OmHT11 // OmHT06
	GO:0004812	F	aminoacyl-tRNA ligase activity	2	14	66	18883	0.0011	0.056	// Medtr3g048190 // Medtr4g119840	// OmHT11 // OmHT06
	GO:0008757	F	S-adenosylmethionine-dependent methyltransferase activity	2	14	64	18883	0.0011	0.056	// Medtr3g460700 // Medtr3g007750	// OmHT22 // OmHT03
	GO:0016875	F	ligase activity, forming carbon-oxygen bonds	2	14	66	18883	0.0011	0.056	// Medtr3g048190 // Medtr4g119840	// OmHT11 // OmHT06
	GO:0016876	F	ligase activity, forming aminoacyl-tRNA and related compound	2	14	66	18883	0.0011	0.056	// Medtr3g048190 // Medtr4g119840	// OmHT11 // OmHT06
	GO:0016874	F	ligase activity	3	14	328	18883	0.0017	0.066	// Medtr3g048190 // Medtr4g119840 // Medtr3g048950	// OmHT11 // OmHT06 // OmHT17
	GO:0006399	P	tRNA metabolic process	2	14	92	18883	0.0021	0.046	// Medtr3g048190 // Medtr4g119840	// OmHT11 // OmHT06
	GO:0000154	P	rRNA modification	1	14	4	18883	0.0037	0.066	// Medtr3g007750	// OmHT03
	GO:0006419	P	alanyl-tRNA aminoacylation	1	14	4	18883	0.0037	0.066	// Medtr4g119840	// OmHT06
	GO:0004813	F	alanine-tRNA ligase activity	1	14	4	18883	0.0037	0.084	// Medtr4g119840	// OmHT06
	GO:0016433	F	rRNA (adenine) methyltransferase activity	1	14	4	18883	0.0037	0.084	// Medtr3g007750	// OmHT03
	GO:0000179	F	rRNA (adenine-N6,N6-)-dimethyltransferase activity	1	14	4	18883	0.0037	0.084	// Medtr3g007750	// OmHT03
	GO:0000166	F	nucleotide binding	8	14	4045	18883	0.0038	0.084	// Medtr7g096260 // Medtr8g007390 // Medtr5g093580 // Medtr3g052610 //	// OmHT20 // OmHT09 // OmHT07 // OmHT15 // OmHT06 // OmHT04 // OmHT11 // OmHT14
	GO:0006433	P	prolyl-tRNA aminoacylation	1	14	5	18883	0.0044	0.073	// Medtr3g048190	// OmHT11
	GO:0004827	F	proline-tRNA ligase activity	1	14	5	18883	0.0044	0.088	// Medtr3g048190	// OmHT11
	GO:0006396	P	RNA processing	2	14	143	18883	0.005	0.077	// Medtr3g460700 // Medtr3g007750	// OmHT22 // OmHT03
	GO:0008649	F	rRNA methyltransferase activity	1	14	6	18883	0.0052	0.093	// Medtr3g007750	// OmHT03
	GO:0006412	P	translation	3	14	500	18883	0.0055	0.078	// Medtr3g048190 // Medtr4g119840 // Medtr3g048950	// OmHT11 // OmHT06 // OmHT17
	GO:0008168	F	methyltransferase activity	2	14	189	18883	0.0085	0.14	// Medtr3g460700 // Medtr3g007750	// OmHT22 // OmHT03
	GO:0016741	F	transferase activity, transferring one-carbon groups	2	14	193	18883	0.0089	0.14	// Medtr3g460700 // Medtr3g007750	// OmHT22 // OmHT03
L. japonicus	GO:0034660	P	ncRNA metabolic process	6	13	488	30415	2.80E-08	2.30E-05	// chr2.CM0177.390.r2.m // LjSGA_019513.1 // chr6.LjT111F18.110.r2.m //	// OmHT11 // OmHT15 // OmHT22 // OmHT03 // OmHT06 // OmHT18
	GO:0006364	P	rRNA processing	4	13	172	30415	7.40E-07	0.00022	// LjSGA_048522.1 // chr3.CM0091.1190.r2.d // LjSGA_019513.1 // chr6.Lj	// OmHT03 // OmHT18 // OmHT15 // OmHT22
	GO:0016072	P	rRNA metabolic process	4	13	174	30415	7.80E-07	0.00022	// LjSGA_048522.1 // chr3.CM0091.1190.r2.d // LjSGA_019513.1 // chr6.Lj	// OmHT03 // OmHT18 // OmHT15 // OmHT22
	GO:0034470	P	ncRNA processing	4	13	343	30415	1.10E-05	0.0023	// LjSGA_048522.1 // chr3.CM0091.1190.r2.d // LjSGA_019513.1 // chr6.Lj	// OmHT03 // OmHT18 // OmHT15 // OmHT22
	GO:0042254	P	ribosome biogenesis	4	13	392	30415	1.80E-05	0.0031	// LjSGA_048522.1 // chr3.CM0091.1190.r2.d // LjSGA_019513.1 // chr6.Lj	// OmHT03 // OmHT18 // OmHT15 // OmHT22
	GO:0055044	C	symplast	2	13	16	30415	2.60E-05	0.0037	// chr3.CM0005.510.r2.m // chr2.CM0177.390.r2.m	// OmHT12 // OmHT11
	GO:0009506	C	plasmodesma	2	13	16	30415	2.60E-05	0.0037	// chr3.CM0005.510.r2.m // chr2.CM0177.390.r2.m	// OmHT12 // OmHT11
	GO:0022613	P	ribonucleoprotein complex biogenesis	4	13	475	30415	3.90E-05	0.0054	// LjSGA_048522.1 // chr3.CM0091.1190.r2.d // LjSGA_019513.1 // chr6.Lj	// OmHT03 // OmHT18 // OmHT15 // OmHT22
	GO:0030686	C	90S preribosome	2	13	40	30415	0.00014	0.014	// LjSGA_048522.1 // LjSGA_019513.1	// OmHT03 // OmHT15
	GO:0030684	C	preribosome	2	13	56	30415	0.00027	0.016	// LjSGA_048522.1 // LjSGA_019513.1	// OmHT03 // OmHT15
	GO:0005911	C	cell-cell junction	2	13	55	30415	0.00027	0.016	// chr3.CM0005.510.r2.m // chr2.CM0177.390.r2.m	// OmHT12 // OmHT11
	GO:0030054	C	cell junction	2	13	62	30415	0.00033	0.016	// chr3.CM0005.510.r2.m // chr2.CM0177.390.r2.m	// OmHT12 // OmHT11
	GO:0044085	P	cellular component biogenesis	5	13	1714	30415	0.0005	0.06	// LjSGA_048522.1 // chr3.CM0005.510.r2.m // LjSGA_019513.1 // chr6.LjT	// OmHT03 // OmHT12 // OmHT15 // OmHT22 // OmHT18
	GO:0006396	P	RNA processing	4	13	1040	30415	0.00077	0.08	// LjSGA_048522.1 // chr3.CM0091.1190.r2.d // LjSGA_019513.1 // chr6.Lj	// OmHT03 // OmHT18 // OmHT15 // OmHT22
	GO:0008168	F	methyltransferase activity	3	13	450	30415	0.00084	0.06	// LjSGA_048522.1 // chr3.CM0091.1190.r2.d // chr6.LjT111F18.110.r2.m	// OmHT03 // OmHT18 // OmHT22
	GO:0003975	F	UDP-N-acetylglucosamine-dolichyl-phosphate N-acetylglucosa	1	13	1	30415	0.00085	0.06	// chr5.CM0278.110.r2.m	// OmHT10
	GO:0042292	F	URM1 activating enzyme activity	1	13	1	30415	0.00085	0.06	// LjSGA_024515.1	// OmHT07
	GO:0016741	F	transferase activity, transferring one-carbon groups	3	13	455	30415	0.00087	0.06	// LjSGA_048522.1 // chr3.CM0091.1190.r2.d // chr6.LjT111F18.110.r2.m	// OmHT03 // OmHT18 // OmHT22
	GO:0000390	P	spliceosome disassembly	1	13	2	30415	0.0013	0.098	// LjSGA_019513.1	// OmHT15
	GO:0032988	P	ribonucleoprotein complex disassembly	1	13	2	30415	0.0013	0.098	// LjSGA_019513.1	// OmHT15
	GO:0006412	P	translation	4	13	1195	30415	0.0013	0.098	// chr2.CM0177.390.r2.m // LjSGA_019513.1 // chr6.CM0836.110.r2.d // Lj	// OmHT11 // OmHT15 // OmHT17 // OmHT06
	GO:0008963	F	phospho-N-acetylmuramoyl-pentapeptide-transferase activity	1	13	2	30415	0.0013	0.064	// chr5.CM0278.110.r2.m	// OmHT10
	GO:0009451	P	RNA modification	2	13	135	30415	0.0015	0.11	// LjSGA_048522.1 // chr3.CM0091.1190.r2.d	// OmHT03 // OmHT18
	GO:0016070	P	RNA metabolic process	6	13	3499	30415	0.0019	0.12	// chr2.CM0177.390.r2.m // LjSGA_019513.1 // chr6.LjT111F18.110.r2.m //	// OmHT11 // OmHT15 // OmHT22 // OmHT03 // OmHT06 // OmHT18
	GO:0006418	P	tRNA aminoacylation for protein translation	2	13	156	30415	0.002	0.12	// chr2.CM0177.390.r2.m // LjSGA_011895.2	// OmHT11 // OmHT06
	GO:0004812	F	aminoacyl-tRNA ligase activity	2	13	161	30415	0.0021	0.064	// chr2.CM0177.390.r2.m // LjSGA_011895.2	// OmHT11 // OmHT06
	GO:0043038	P	amino acid activation	2	13	164	30415	0.0022	0.12	// chr2.CM0177.390.r2.m // LjSGA_011895.2	// OmHT11 // OmHT06
	GO:0043039	P	tRNA aminoacylation	2	13	164	30415	0.0022	0.12	// chr2.CM0177.390.r2.m // LjSGA_011895.2	// OmHT11 // OmHT06
	GO:0016875	F	ligase activity, forming carbon-oxygen bonds	2	13	169	30415	0.0024	0.064	// chr2.CM0177.390.r2.m // LjSGA_011895.2	// OmHT11 // OmHT06
	GO:0016876	F	ligase activity, forming aminoacyl-tRNA and related compound	2	13	169	30415	0.0024	0.064	// chr2.CM0177.390.r2.m // LjSGA_011895.2	// OmHT11 // OmHT06
	GO:0016433	F	rRNA (adenine) methyltransferase activity	1	13	5	30415	0.0026	0.064	// LjSGA_048522.1	// OmHT03
	GO:0000179	F	rRNA (adenine-N6,N6-)-dimethyltransferase activity	1	13	5	30415	0.0026	0.064	// LjSGA_048522.1	// OmHT03
	GO:0008265	F	Mo-molybdopterin cofactor sulfurase activity	1	13	5	30415	0.0026	0.064	// LjSGA_024515.1	// OmHT07
	GO:0005689	C	U12-type spliceosomal complex	1	13	5	30415	0.0026	0.1	// LjSGA_019513.1	// OmHT15
	GO:0006433	P	prolyl-tRNA aminoacylation	1	13	6	30415	0.003	0.15	// chr2.CM0177.390.r2.m	// OmHT11
	GO:0004827	F	proline-tRNA ligase activity	1	13	6	30415	0.003	0.068	// chr2.CM0177.390.r2.m	// OmHT11
	GO:0007124	P	pseudohyphal growth	1	13	7	30415	0.0034	0.15	// LjSGA_024515.1	// OmHT07
	GO:0001403	P	invasive growth in response to glucose limitation	1	13	7	30415	0.0034	0.15	// LjSGA_024515.1	// OmHT07
	GO:0043228	C	non-membrane-bounded organelle	5	13	2632	30415	0.0035	0.11	// LjSGA_048522.1 // chr3.CM0005.510.r2.m // LjSGA_019513.1 // chr1.LjT	// OmHT03 // OmHT12 // OmHT15 // OmHT20 // OmHT22
	GO:0043232	C	intracellular non-membrane-bounded organelle	5	13	2632	30415	0.0035	0.11	// LjSGA_048522.1 // chr3.CM0005.510.r2.m // LjSGA_019513.1 // chr1.LjT	// OmHT03 // OmHT12 // OmHT15 // OmHT20 // OmHT22
	GO:0008757	F	S-adenosylmethionine-dependent methyltransferase activity	2	13	214	30415	0.0037	0.079	// LjSGA_048522.1 // chr6.LjT111F18.110.r2.m	// OmHT03 // OmHT22
	GO:0007114	P	cell budding	1	13	8	30415	0.0038	0.16	// LjSGA_024515.1	// OmHT07
	GO:0006419	P	alanyl-tRNA aminoacylation	1	13	9	30415	0.0043	0.16	// LjSGA_011895.2	// OmHT06
	GO:0004813	F	alanine-tRNA ligase activity	1	13	9	30415	0.0043	0.084	// LjSGA_011895.2	// OmHT06
	GO:0010467	P	gene expression	7	13	5659	30415	0.0046	0.16	// chr2.CM0177.390.r2.m // LjSGA_019513.1 // chr6.LjT111F18.110.r2.m //	// OmHT11 // OmHT15 // OmHT22 // OmHT03 // OmHT06 // OmHT18 // OmHT17
	GO:0070783	P	growth of unicellular organism as a thread of attached cells	1	13	10	30415	0.0047	0.16	// LjSGA_024515.1	// OmHT07
	GO:0044182	P	filamentous growth of a population of unicellular organisms	1	13	10	30415	0.0047	0.16	// LjSGA_024515.1	// OmHT07
	GO:0009574	C	preprophase band	1	13	10	30415	0.0047	0.13	// chr3.CM0005.510.r2.m	// OmHT12
	GO:0005819	C	spindle	2	13	246	30415	0.0049	0.13	// LjSGA_048522.1 // chr3.CM0005.510.r2.m	// OmHT03 // OmHT12
	GO:0000463	P	maturation of LSU-rRNA from tricistronic rRNA transcript (SSU	1	13	11	30415	0.0051	0.16	// LjSGA_019513.1	// OmHT15
	GO:0000470	P	maturation of LSU-rRNA	1	13	11	30415	0.0051	0.16	// LjSGA_019513.1	// OmHT15
	GO:0008649	F	rRNA methyltransferase activity	1	13	11	30415	0.0051	0.094	// LjSGA_048522.1	// OmHT03
	GO:0046785	P	microtubule polymerization	1	13	16	30415	0.0072	0.22	// chr3.CM0005.510.r2.m	// OmHT12
	GO:0006399	P	tRNA metabolic process	2	13	317	30415	0.0079	0.24	// chr2.CM0177.390.r2.m // LjSGA_011895.2	// OmHT11 // OmHT06
	GO:0000154	P	rRNA modification	1	13	19	30415	0.0085	0.24	// LjSGA_048522.1	// OmHT03
	GO:0008641	F	small protein activating enzyme activity	1	13	20	30415	0.0089	0.15	// LjSGA_024515.1	// OmHT07
	GO:0016874	F	ligase activity	3	13	1055	30415	0.0092	0.15	// chr2.CM0177.390.r2.m // LjSGA_011895.2 // chr6.CM0836.110.r2.d	// OmHT11 // OmHT06 // OmHT17
	GO:0031163	P	metallo-sulfur cluster assembly	1	13	22	30415	0.0098	0.26	// chr1.CM0010.110.r2.d	// OmHT02
	GO:0016226	P	iron-sulfur cluster assembly	1	13	22	30415	0.0098	0.26	// chr1.CM0010.110.r2.d	// OmHT02
P. vulgaris	GO:0034660	P	ncRNA metabolic process	4	13	124	15454	3.00E-06	0.00097	// Phvul.008G161800 // Phvul.011G044300 // Phvul.001G218300 // Phvul.(	// OmHT03 // OmHT18 // OmHT11 // OmHT06
	GO:0006364	P	rRNA processing	2	13	27	15454	0.00026	0.028	// Phvul.008G161800 // Phvul.011G044300	// OmHT03 // OmHT18
	GO:0016072	P	rRNA metabolic process	2	13	27	15454	0.00026	0.028	// Phvul.008G161800 // Phvul.011G044300	// OmHT03 // OmHT18
	GO:0044085	P	cellular component biogenesis	3	13	192	15454	0.00051	0.029	// Phvul.008G161800 // Phvul.011G044300 // Phvul.005G182600	// OmHT03 // OmHT18 // OmHT21
	GO:0042254	P	ribosome biogenesis	2	13	43	15454	0.00063	0.029	// Phvul.008G161800 // Phvul.011G044300	// OmHT03 // OmHT18
	GO:0022613	P	ribonucleoprotein complex biogenesis	2	13	46	15454	0.00072	0.029	// Phvul.008G161800 // Phvul.011G044300	// OmHT03 // OmHT18
	GO:0006418	P	tRNA aminoacylation for protein translation	2	13	48	15454	0.00078	0.029	// Phvul.001G218300 // Phvul.003G105100	// OmHT11 // OmHT06
	GO:0004812	F	aminoacyl-tRNA ligase activity	2	13	48	15454	0.00078	0.05	// Phvul.001G218300 // Phvul.003G105100	// OmHT11 // OmHT06
	GO:0043038	P	amino acid activation	2	13	49	15454	0.00081	0.029	// Phvul.001G218300 // Phvul.003G105100	// OmHT11 // OmHT06
	GO:0043039	P	tRNA aminoacylation	2	13	49	15454	0.00081	0.029	// Phvul.001G218300 // Phvul.003G105100	// OmHT11 // OmHT06
	GO:0016875	F	ligase activity, forming carbon-oxygen bonds	2	13	49	15454	0.00081	0.05	// Phvul.001G218300 // Phvul.003G105100	// OmHT11 // OmHT06
	GO:0016876	F	ligase activity, forming aminoacyl-tRNA and related compound	2	13	49	15454	0.00081	0.05	// Phvul.001G218300 // Phvul.003G105100	// OmHT11 // OmHT06
	GO:0009451	P	RNA modification	2	13	53	15454	0.00094	0.029	// Phvul.008G161800 // Phvul.011G044300	// OmHT03 // OmHT18
	GO:0043414	P	macromolecule methylation	2	13	56	15454	0.001	0.029	// Phvul.011G044300 // Phvul.003G263600	// OmHT18 // OmHT13
	GO:0032259	P	methylation	2	13	57	15454	0.0011	0.029	// Phvul.011G044300 // Phvul.003G263600	// OmHT18 // OmHT13
	GO:0006730	P	one-carbon metabolic process	2	13	59	15454	0.0012	0.029	// Phvul.011G044300 // Phvul.003G263600	// OmHT18 // OmHT13
	GO:0016874	F	ligase activity	3	13	259	15454	0.0012	0.05	// Phvul.006G036200 // Phvul.001G218300 // Phvul.003G105100	// OmHT17 // OmHT11 // OmHT06
	GO:0008168	F	methyltransferase activity	3	13	275	15454	0.0014	0.05	// Phvul.008G161800 // Phvul.011G044300 // Phvul.003G263600	// OmHT03 // OmHT18 // OmHT13
	GO:0016741	F	transferase activity, transferring one-carbon groups	3	13	277	15454	0.0015	0.05	// Phvul.008G161800 // Phvul.011G044300 // Phvul.003G263600	// OmHT03 // OmHT18 // OmHT13
	GO:0034470	P	ncRNA processing	2	13	74	15454	0.0018	0.041	// Phvul.008G161800 // Phvul.011G044300	// OmHT03 // OmHT18
	GO:0006433	P	prolyl-tRNA aminoacylation	1	13	2	15454	0.0025	0.054	// Phvul.001G218300	// OmHT11
	GO:0008963	F	phospho-N-acetylmuramoyl-pentapeptide-transferase activity	1	13	2	15454	0.0025	0.064	// Phvul.007G249500	// OmHT10
	GO:0004827	F	proline-tRNA ligase activity	1	13	2	15454	0.0025	0.064	// Phvul.001G218300	// OmHT11
	GO:0044281	P	small molecule metabolic process	4	13	769	15454	0.0031	0.059	// Phvul.011G044300 // Phvul.003G263600 // Phvul.001G218300 // Phvul.(	// OmHT18 // OmHT13 // OmHT11 // OmHT06
	GO:0006399	P	tRNA metabolic process	2	13	99	15454	0.0031	0.059	// Phvul.001G218300 // Phvul.003G105100	// OmHT11 // OmHT06
	GO:0006419	P	alanyl-tRNA aminoacylation	1	13	4	15454	0.0042	0.075	// Phvul.003G105100	// OmHT06
	GO:0016433	F	rRNA (adenine) methyltransferase activity	1	13	4	15454	0.0042	0.077	// Phvul.008G161800	// OmHT03
	GO:0004813	F	alanine-tRNA ligase activity	1	13	4	15454	0.0042	0.077	// Phvul.003G105100	// OmHT06
	GO:0000179	F	rRNA (adenine-N6,N6-)-dimethyltransferase activity	1	13	4	15454	0.0042	0.077	// Phvul.008G161800	// OmHT03
	GO:0008107	F	galactoside 2-alpha-L-fucosyltransferase activity	1	13	6	15454	0.0059	0.085	// Phvul.005G182600	// OmHT21
	GO:0008649	F	rRNA methyltransferase activity	1	13	6	15454	0.0059	0.085	// Phvul.008G161800	// OmHT03
	GO:0031127	F	alpha(1,2)-fucosyltransferase activity	1	13	6	15454	0.0059	0.085	// Phvul.005G182600	// OmHT21
	GO:0042546	P	cell wall biogenesis	1	13	7	15454	0.0067	0.11	// Phvul.005G182600	// OmHT21
	GO:0006396	P	RNA processing	2	13	155	15454	0.0074	0.12	// Phvul.008G161800 // Phvul.011G044300	// OmHT03 // OmHT18
	GO:0008417	F	fucosyltransferase activity	1	13	8	15454	0.0075	0.1	// Phvul.005G182600	// OmHT21
	GO:0000154	P	rRNA modification	1	13	10	15454	0.0092	0.14	// Phvul.008G161800	// OmHT03
	GO:0016780	F	phosphotransferase activity, for other substituted phosphate gr	1	13	10	15454	0.0092	0.12	// Phvul.007G249500	// OmHT10
G. max	GO:0034660	P	ncRNA metabolic process	3	13	246	29501	0.00016	0.025	// Glyma.19G221400 // Glyma.12G042600 // Glyma.06G316500	// OmHT11 // OmHT18 // OmHT03
	GO:0006364	P	rRNA processing	2	13	50	29501	0.00023	0.025	// Glyma.12G042600 // Glyma.06G316500	// OmHT18 // OmHT03
	GO:0016072	P	rRNA metabolic process	2	13	50	29501	0.00023	0.025	// Glyma.12G042600 // Glyma.06G316500	// OmHT18 // OmHT03
	GO:0044085	P	cellular component biogenesis	3	13	352	29501	0.00045	0.036	// Glyma.12G042600 // Glyma.13G370500 // Glyma.06G316500	// OmHT18 // OmHT21 // OmHT03
	GO:0042254	P	ribosome biogenesis	2	13	83	29501	0.00063	0.037	// Glyma.12G042600 // Glyma.06G316500	// OmHT18 // OmHT03
	GO:0022613	P	ribonucleoprotein complex biogenesis	2	13	87	29501	0.00069	0.037	// Glyma.12G042600 // Glyma.06G316500	// OmHT18 // OmHT03
	GO:0032259	P	methylation	2	13	113	29501	0.0011	0.049	// Glyma.02G047300 // Glyma.12G042600	// OmHT13 // OmHT18
	GO:0006730	P	one-carbon metabolic process	2	13	117	29501	0.0012	0.049	// Glyma.02G047300 // Glyma.12G042600	// OmHT13 // OmHT18
	GO:0008168	F	methyltransferase activity	3	13	499	29501	0.0012	0.12	// Glyma.02G047300 // Glyma.12G042600 // Glyma.06G316500	// OmHT13 // OmHT18 // OmHT03
	GO:0016741	F	transferase activity, transferring one-carbon groups	3	13	506	29501	0.0013	0.12	// Glyma.02G047300 // Glyma.12G042600 // Glyma.06G316500	// OmHT13 // OmHT18 // OmHT03
	GO:0034470	P	ncRNA processing	2	13	150	29501	0.002	0.071	// Glyma.12G042600 // Glyma.06G316500	// OmHT18 // OmHT03
	GO:0006433	P	prolyl-tRNA aminoacylation	1	13	4	29501	0.0022	0.071	// Glyma.19G221400	// OmHT11
	GO:0008963	F	phospho-N-acetylmuramoyl-pentapeptide-transferase activity	1	13	4	29501	0.0022	0.12	// Glyma.02G190700	// OmHT10
	GO:0004827	F	proline-tRNA ligase activity	1	13	4	29501	0.0022	0.12	// Glyma.19G221400	// OmHT11
	GO:0016433	F	rRNA (adenine) methyltransferase activity	1	13	6	29501	0.0031	0.12	// Glyma.06G316500	// OmHT03
	GO:0000179	F	rRNA (adenine-N6,N6-)-dimethyltransferase activity	1	13	6	29501	0.0031	0.12	// Glyma.06G316500	// OmHT03
	GO:0008107	F	galactoside 2-alpha-L-fucosyltransferase activity	1	13	10	29501	0.0048	0.14	// Glyma.13G370500	// OmHT21
	GO:0031127	F	alpha(1,2)-fucosyltransferase activity	1	13	10	29501	0.0048	0.14	// Glyma.13G370500	// OmHT21
	GO:0008649	F	rRNA methyltransferase activity	1	13	11	29501	0.0053	0.14	// Glyma.06G316500	// OmHT03
	GO:0000154	P	rRNA modification	1	13	12	29501	0.0057	0.17	// Glyma.06G316500	// OmHT03
	GO:0006396	P	RNA processing	2	13	273	29501	0.0063	0.17	// Glyma.12G042600 // Glyma.06G316500	// OmHT18 // OmHT03
	GO:0016780	F	phosphotransferase activity, for other substituted phosphate gr	1	13	15	29501	0.007	0.16	// Glyma.02G190700	// OmHT10
	GO:0008417	F	fucosyltransferase activity	1	13	16	29501	0.0075	0.16	// Glyma.13G370500	// OmHT21
	GO:0051301	P	cell division	1	13	21	29501	0.0096	0.22	// Glyma.13G189600	// OmHT12
	GO:0000910	P	cytokinesis	1	13	21	29501	0.0096	0.22	// Glyma.13G189600	// OmHT12
O. sativa	No significant GO term found										
P. virgatum	GO:0006772	P	thiamin metabolic process	2	48	15	36473	0.00023	0.11	// Pavir.Ca00302 // Pavir.J26403	// AiHT25 // AiHT77
	GO:0009228	P	thiamin biosynthetic process	2	48	15	36473	0.00023	0.11	// Pavir.Ca00302 // Pavir.J26403	// AiHT25 // AiHT77
	GO:0042723	P	thiamin and derivative metabolic process	2	48	25	36473	0.00058	0.14	// Pavir.Ca00302 // Pavir.J26403	// AiHT25 // AiHT77
	GO:0042724	P	thiamin and derivative biosynthetic process	2	48	25	36473	0.00058	0.14	// Pavir.Ca00302 // Pavir.J26403	// AiHT25 // AiHT77
	GO:0006766	P	vitamin metabolic process	2	48	57	36473	0.0028	0.29	// Pavir.Ca00302 // Pavir.J26403	// AiHT25 // AiHT77
	GO:0044272	P	sulfur compound biosynthetic process	2	48	57	36473	0.0028	0.29	// Pavir.Ca00302 // Pavir.J26403	// AiHT25 // AiHT77
	GO:0042364	P	water-soluble vitamin biosynthetic process	2	48	57	36473	0.0028	0.29	// Pavir.Ca00302 // Pavir.J26403	// AiHT25 // AiHT77
	GO:0006767	P	water-soluble vitamin metabolic process	2	48	57	36473	0.0028	0.29	// Pavir.Ca00302 // Pavir.J26403	// AiHT25 // AiHT77
	GO:0009110	P	vitamin biosynthetic process	2	48	57	36473	0.0028	0.29	// Pavir.Ca00302 // Pavir.J26403	// AiHT25 // AiHT77
	GO:0006790	P	sulfur metabolic process	2	48	67	36473	0.0038	0.35	// Pavir.Ca00302 // Pavir.J26403	// AiHT25 // AiHT77
	GO:0004789	F	thiamin-phosphate diphosphorylase activity	1	48	2	36473	0.0039	1	// Pavir.Ca00302	// AiHT25
	GO:0019438	P	aromatic compound biosynthetic process	2	48	74	36473	0.0045	0.39	// Pavir.Ca00302 // Pavir.J26403	// AiHT25 // AiHT77
S. italica	GO:0009228	P	thiamin biosynthetic process	3	54	7	21853	1.70E-06	0.0045	// Si034631m.g // Si009759m.g // Si030346m.g	// AiHT77 // AiHT25 // AiHT51
	GO:0044272	P	sulfur compound biosynthetic process	5	54	221	21853	0.00023	0.29	// Si034631m.g // Si009759m.g // Si030346m.g // Si013712m.g // Si010368	// AiHT77 // AiHT25 // AiHT51 // AiHT78 // AiHT18
	GO:0042364	P	water-soluble vitamin biosynthetic process	3	54	55	21853	0.0004	0.29	// Si034631m.g // Si009759m.g // Si030346m.g	// AiHT77 // AiHT25 // AiHT51
	GO:0007018	P	microtubule-based movement	3	54	57	21853	0.00044	0.29	// Si005702m.g // Si016200m.g // Si035680m.g	// AiHT31 // AiHT14 // AiHT05
	GO:0044444	C	cytoplasmic part	21	54	4132	21853	0.00051	0.083	// Si029048m.g // Si035427m.g // Si018070m.g // Si030910m.g // Si010368	// AiHT66 // AiHT63 // AiHT50 // AiHT73 // AiHT18 // AiHT71 // AiHT25 // AiHT34 // AiHT01 // AiHT13 // AiHT57 // AiHT77 // AiHT51 // AiHT78 // AiHT10 // AiHT28 // AiHT43 // AiHT05 // AiHT20 // AiHT58 // AiHT26
	GO:0005622	C	intracellular	28	54	6477	21853	0.00053	0.083	// Si010368m.g // Si009759m.g // Si006602m.g // Si034631m.g // Si018070	// AiHT18 // AiHT25 // AiHT35 // AiHT77 // AiHT50 // AiHT43 // AiHT13 // AiHT14 // AiHT31 // AiHT58 // AiHT42 // AiHT52 // AiHT34 // AiHT57 // AiHT66 // AiHT10 // AiHT05 // AiHT73 // AiHT76 // AiHT01 // AiHT71 // AiHT20 // AiHT63 // AiHT51 // AiHT28 // AiHT70 // AiHT78 // AiHT26
	GO:0006767	P	water-soluble vitamin metabolic process	3	54	65	21853	0.00063	0.29	// Si034631m.g // Si009759m.g // Si030346m.g	// AiHT77 // AiHT25 // AiHT51
	GO:0009110	P	vitamin biosynthetic process	3	54	67	21853	0.00069	0.29	// Si034631m.g // Si009759m.g // Si030346m.g	// AiHT77 // AiHT25 // AiHT51
	GO:0006790	P	sulfur metabolic process	5	54	287	21853	0.00075	0.29	// Si034631m.g // Si009759m.g // Si030346m.g // Si013712m.g // Si010368	// AiHT77 // AiHT25 // AiHT51 // AiHT78 // AiHT18
	GO:0043229	C	intracellular organelle	24	54	5256	21853	0.00082	0.083	// Si010368m.g // Si009759m.g // Si006602m.g // Si034631m.g // Si018070	// AiHT18 // AiHT25 // AiHT35 // AiHT77 // AiHT50 // AiHT43 // AiHT13 // AiHT14 // AiHT31 // AiHT42 // AiHT52 // AiHT34 // AiHT66 // AiHT10 // AiHT05 // AiHT73 // AiHT76 // AiHT01 // AiHT71 // AiHT20 // AiHT63 // AiHT51 // AiHT28 // AiHT78
	GO:0043226	C	organelle	24	54	5259	21853	0.00083	0.083	// Si010368m.g // Si009759m.g // Si006602m.g // Si034631m.g // Si018070	// AiHT18 // AiHT25 // AiHT35 // AiHT77 // AiHT50 // AiHT43 // AiHT13 // AiHT14 // AiHT31 // AiHT42 // AiHT52 // AiHT34 // AiHT66 // AiHT10 // AiHT05 // AiHT73 // AiHT76 // AiHT01 // AiHT71 // AiHT20 // AiHT63 // AiHT51 // AiHT28 // AiHT78
	GO:0044424	C	intracellular part	27	54	6303	21853	0.00085	0.083	// Si010368m.g // Si009759m.g // Si006602m.g // Si034631m.g // Si018070	// AiHT18 // AiHT25 // AiHT35 // AiHT77 // AiHT50 // AiHT43 // AiHT13 // AiHT14 // AiHT31 // AiHT58 // AiHT42 // AiHT52 // AiHT34 // AiHT57 // AiHT66 // AiHT10 // AiHT05 // AiHT73 // AiHT76 // AiHT01 // AiHT71 // AiHT20 // AiHT63 // AiHT51 // AiHT28 // AiHT78 // AiHT26
	GO:0016810	F	hydrolase activity, acting on carbon-nitrogen (but not peptide) b	3	54	86	21853	0.0014	0.71	// Si001397m.g // Si013712m.g // Si021935m.g	// AiHT10 // AiHT78 // AiHT49
	GO:0005737	C	cytoplasm	21	54	4449	21853	0.0014	0.11	// Si029048m.g // Si035427m.g // Si018070m.g // Si030910m.g // Si010368	// AiHT66 // AiHT63 // AiHT50 // AiHT73 // AiHT18 // AiHT71 // AiHT25 // AiHT34 // AiHT01 // AiHT13 // AiHT57 // AiHT77 // AiHT51 // AiHT78 // AiHT10 // AiHT28 // AiHT43 // AiHT05 // AiHT20 // AiHT58 // AiHT26
	GO:0006766	P	vitamin metabolic process	3	54	91	21853	0.0016	0.55	// Si034631m.g // Si009759m.g // Si030346m.g	// AiHT77 // AiHT25 // AiHT51
	GO:0005874	C	microtubule	3	54	95	21853	0.0018	0.12	// Si005702m.g // Si016200m.g // Si035680m.g	// AiHT31 // AiHT14 // AiHT05
	GO:0042744	P	hydrogen peroxide catabolic process	2	54	26	21853	0.0022	0.65	// Si006189m.g // Si010368m.g	// AiHT43 // AiHT18
	GO:0009507	C	chloroplast	10	54	1424	21853	0.0024	0.12	// Si029048m.g // Si030910m.g // Si010368m.g // Si009759m.g // Si036782	// AiHT66 // AiHT73 // AiHT18 // AiHT25 // AiHT01 // AiHT13 // AiHT77 // AiHT51 // AiHT43 // AiHT20
	GO:0070301	P	cellular response to hydrogen peroxide	2	54	28	21853	0.0025	0.67	// Si006189m.g // Si010368m.g	// AiHT43 // AiHT18
	GO:0010319	C	stromule	2	54	30	21853	0.0028	0.12	// Si006189m.g // Si030346m.g	// AiHT43 // AiHT51
	GO:0034614	P	cellular response to reactive oxygen species	2	54	31	21853	0.003	0.74	// Si006189m.g // Si010368m.g	// AiHT43 // AiHT18
	GO:0044422	C	organelle part	14	54	2547	21853	0.003	0.12	// Si029048m.g // Si018070m.g // Si030910m.g // Si035680m.g // Si030346	// AiHT66 // AiHT50 // AiHT73 // AiHT05 // AiHT51 // AiHT52 // AiHT25 // AiHT77 // AiHT71 // AiHT28 // AiHT14 // AiHT35 // AiHT43 // AiHT31
	GO:0044446	C	intracellular organelle part	14	54	2546	21853	0.003	0.12	// Si029048m.g // Si018070m.g // Si030910m.g // Si035680m.g // Si030346	// AiHT66 // AiHT50 // AiHT73 // AiHT05 // AiHT51 // AiHT52 // AiHT25 // AiHT77 // AiHT71 // AiHT28 // AiHT14 // AiHT35 // AiHT43 // AiHT31
	GO:0005773	C	vacuole	6	54	585	21853	0.0032	0.12	// Si029048m.g // Si032963m.g // Si006225m.g // Si035427m.g // Si001397	// AiHT66 // AiHT71 // AiHT34 // AiHT63 // AiHT10 // AiHT78
	GO:0009536	C	plastid	10	54	1496	21853	0.0034	0.12	// Si029048m.g // Si030910m.g // Si010368m.g // Si009759m.g // Si036782	// AiHT66 // AiHT73 // AiHT18 // AiHT25 // AiHT01 // AiHT13 // AiHT77 // AiHT51 // AiHT43 // AiHT20
	GO:0043231	C	intracellular membrane-bounded organelle	21	54	4799	21853	0.0037	0.12	// Si029048m.g // Si035427m.g // Si018070m.g // Si030910m.g // Si006509	// AiHT66 // AiHT63 // AiHT50 // AiHT73 // AiHT42 // AiHT18 // AiHT52 // AiHT25 // AiHT34 // AiHT01 // AiHT35 // AiHT13 // AiHT77 // AiHT71 // AiHT10 // AiHT28 // AiHT43 // AiHT20 // AiHT51 // AiHT78 // AiHT76
	GO:0043227	C	membrane-bounded organelle	21	54	4812	21853	0.0038	0.12	// Si029048m.g // Si035427m.g // Si018070m.g // Si030910m.g // Si006509	// AiHT66 // AiHT63 // AiHT50 // AiHT73 // AiHT42 // AiHT18 // AiHT52 // AiHT25 // AiHT34 // AiHT01 // AiHT35 // AiHT13 // AiHT77 // AiHT71 // AiHT10 // AiHT28 // AiHT43 // AiHT20 // AiHT51 // AiHT78 // AiHT76
	GO:0044464	C	cell part	32	54	8912	21853	0.0047	0.14	// Si010368m.g // Si009759m.g // Si006602m.g // Si034631m.g // Si018070	// AiHT18 // AiHT25 // AiHT35 // AiHT77 // AiHT50 // AiHT54 // AiHT43 // AiHT13 // AiHT14 // AiHT16 // AiHT23 // AiHT31 // AiHT58 // AiHT42 // AiHT52 // AiHT34 // AiHT57 // AiHT66 // AiHT10 // AiHT12 // AiHT05 // AiHT73 // AiHT76 // AiHT01 // AiHT71 // AiHT20 // AiHT63 // AiHT51 // AiHT28 // AiHT70 // AiHT78 // AiHT26
	GO:0005623	C	cell	32	54	8913	21853	0.0047	0.14	// Si010368m.g // Si009759m.g // Si006602m.g // Si034631m.g // Si018070	// AiHT18 // AiHT25 // AiHT35 // AiHT77 // AiHT50 // AiHT54 // AiHT43 // AiHT13 // AiHT14 // AiHT16 // AiHT23 // AiHT31 // AiHT58 // AiHT42 // AiHT52 // AiHT34 // AiHT57 // AiHT66 // AiHT10 // AiHT12 // AiHT05 // AiHT73 // AiHT76 // AiHT01 // AiHT71 // AiHT20 // AiHT63 // AiHT51 // AiHT28 // AiHT70 // AiHT78 // AiHT26
	GO:0034599	P	cellular response to oxidative stress	2	54	40	21853	0.0048	0.78	// Si006189m.g // Si010368m.g	// AiHT43 // AiHT18
	GO:0046484	P	oxazole or thiazole metabolic process	1	54	1	21853	0.0049	0.78	// Si030346m.g	// AiHT51
	GO:0018131	P	oxazole or thiazole biosynthetic process	1	54	1	21853	0.0049	0.78	// Si030346m.g	// AiHT51
	GO:0010266	P	response to vitamin B1	1	54	1	21853	0.0049	0.78	// Si034631m.g	// AiHT77
	GO:0006006	P	glucose metabolic process	4	54	274	21853	0.0049	0.78	// Si034631m.g // Si035680m.g // Si005911m.g // Si010368m.g	// AiHT77 // AiHT05 // AiHT40 // AiHT18
	GO:0019500	P	cyanide catabolic process	1	54	1	21853	0.0049	0.78	// Si010368m.g	// AiHT18
	GO:0015431	F	glutathione S-conjugate-exporting ATPase activity	1	54	1	21853	0.0049	0.71	// Si032963m.g	// AiHT71
	GO:0010290	F	chlorophyll catabolite transmembrane transporter activity	1	54	1	21853	0.0049	0.71	// Si032963m.g	// AiHT71
	GO:0050017	F	L-3-cyanoalanine synthase activity	1	54	1	21853	0.0049	0.71	// Si010368m.g	// AiHT18
	GO:0015630	C	microtubule cytoskeleton	3	54	137	21853	0.005	0.14	// Si005702m.g // Si016200m.g // Si035680m.g	// AiHT31 // AiHT14 // AiHT05
	GO:0016811	F	hydrolase activity, acting on carbon-nitrogen (but not peptide) b	2	54	42	21853	0.0053	0.71	// Si001397m.g // Si013712m.g	// AiHT10 // AiHT78
	GO:0044271	P	cellular nitrogen compound biosynthetic process	5	54	466	21853	0.006	0.9	// Si034631m.g // Si009759m.g // Si030346m.g // Si013712m.g // Si010368	// AiHT77 // AiHT25 // AiHT51 // AiHT78 // AiHT18
	GO:0033273	P	response to vitamin	1	54	2	21853	0.0074	0.9	// Si034631m.g	// AiHT77
	GO:0051410	P	detoxification of nitrogen compound	1	54	2	21853	0.0074	0.9	// Si010368m.g	// AiHT18
	GO:0015689	P	molybdate ion transport	1	54	2	21853	0.0074	0.9	// Si035427m.g	// AiHT63
	GO:0051409	P	response to nitrosative stress	1	54	2	21853	0.0074	0.9	// Si010368m.g	// AiHT18
	GO:0004046	F	aminoacylase activity	1	54	2	21853	0.0074	0.71	// Si013712m.g	// AiHT78
	GO:0004591	F	oxoglutarate dehydrogenase (succinyl-transferring) activity	1	54	2	21853	0.0074	0.71	// Si009245m.g	// AiHT75
	GO:0015098	F	molybdate ion transmembrane transporter activity	1	54	2	21853	0.0074	0.71	// Si035427m.g	// AiHT63
	GO:0019318	P	hexose metabolic process	4	54	312	21853	0.0076	0.9	// Si034631m.g // Si035680m.g // Si005911m.g // Si010368m.g	// AiHT77 // AiHT05 // AiHT40 // AiHT18
	GO:0005871	C	kinesin complex	2	54	57	21853	0.0093	0.24	// Si005702m.g // Si016200m.g	// AiHT31 // AiHT14
	GO:0003777	F	microtubule motor activity	2	54	58	21853	0.0096	0.77	// Si005702m.g // Si016200m.g	// AiHT31 // AiHT14
	GO:0019499	P	cyanide metabolic process	1	54	3	21853	0.0098	0.98	// Si010368m.g	// AiHT18
	GO:0080111	P	DNA demethylation	1	54	3	21853	0.0098	0.98	// Si006812m.g	// AiHT70
	GO:0016045	P	detection of bacterium	1	54	3	21853	0.0098	0.98	// Si034631m.g	// AiHT77
	GO:0048281	P	inflorescence morphogenesis	1	54	3	21853	0.0098	0.98	// Si006994m.g	// AiHT39
S. bicolor	GO:0006772	P	thiamin metabolic process	2	49	7	15594	0.00034	0.15	// Sobic.001G138000 // Sobic.008G067100	// AiHT77 // AiHT25
	GO:0009228	P	thiamin biosynthetic process	2	49	7	15594	0.00034	0.15	// Sobic.001G138000 // Sobic.008G067100	// AiHT77 // AiHT25
	GO:0042723	P	thiamin and derivative metabolic process	2	49	10	15594	0.00062	0.15	// Sobic.001G138000 // Sobic.008G067100	// AiHT77 // AiHT25
	GO:0042724	P	thiamin and derivative biosynthetic process	2	49	10	15594	0.00062	0.15	// Sobic.001G138000 // Sobic.008G067100	// AiHT77 // AiHT25
	GO:0044272	P	sulfur compound biosynthetic process	2	49	23	15594	0.0028	0.35	// Sobic.001G138000 // Sobic.008G067100	// AiHT77 // AiHT25
	GO:0006766	P	vitamin metabolic process	2	49	27	15594	0.0037	0.35	// Sobic.001G138000 // Sobic.008G067100	// AiHT77 // AiHT25
	GO:0042364	P	water-soluble vitamin biosynthetic process	2	49	27	15594	0.0037	0.35	// Sobic.001G138000 // Sobic.008G067100	// AiHT77 // AiHT25
	GO:0006767	P	water-soluble vitamin metabolic process	2	49	27	15594	0.0037	0.35	// Sobic.001G138000 // Sobic.008G067100	// AiHT77 // AiHT25
	GO:0009110	P	vitamin biosynthetic process	2	49	27	15594	0.0037	0.35	// Sobic.001G138000 // Sobic.008G067100	// AiHT77 // AiHT25
	GO:0006790	P	sulfur metabolic process	2	49	27	15594	0.0037	0.35	// Sobic.001G138000 // Sobic.008G067100	// AiHT77 // AiHT25
	GO:0019438	P	aromatic compound biosynthetic process	2	49	30	15594	0.0045	0.39	// Sobic.001G138000 // Sobic.008G067100	// AiHT77 // AiHT25
	GO:0004789	F	thiamin-phosphate diphosphorylase activity	1	49	1	15594	0.0063	1	// Sobic.008G067100	// AiHT25
Z. mays	GO:0009228	P	thiamin biosynthetic process	3	52	5	25864	4.20E-07	0.00042	// GRMZM2G074097 // GRMZM2G401934 // GRMZM2G027663	// AiHT51 // AiHT25 // AiHT77
	GO:0006772	P	thiamin metabolic process	3	52	7	25864	9.10E-07	0.00042	// GRMZM2G074097 // GRMZM2G401934 // GRMZM2G027663	// AiHT51 // AiHT25 // AiHT77
	GO:0042723	P	thiamin and derivative metabolic process	3	52	7	25864	9.10E-07	0.00042	// GRMZM2G074097 // GRMZM2G401934 // GRMZM2G027663	// AiHT51 // AiHT25 // AiHT77
	GO:0042724	P	thiamin and derivative biosynthetic process	3	52	7	25864	9.10E-07	0.00042	// GRMZM2G074097 // GRMZM2G401934 // GRMZM2G027663	// AiHT51 // AiHT25 // AiHT77
	GO:0042364	P	water-soluble vitamin biosynthetic process	3	52	68	25864	0.0004	0.14	// GRMZM2G074097 // GRMZM2G401934 // GRMZM2G027663	// AiHT51 // AiHT25 // AiHT77
	GO:0006767	P	water-soluble vitamin metabolic process	3	52	71	25864	0.00045	0.14	// GRMZM2G074097 // GRMZM2G401934 // GRMZM2G027663	// AiHT51 // AiHT25 // AiHT77
	GO:0009110	P	vitamin biosynthetic process	3	52	84	25864	0.00072	0.18	// GRMZM2G074097 // GRMZM2G401934 // GRMZM2G027663	// AiHT51 // AiHT25 // AiHT77
	GO:0006766	P	vitamin metabolic process	3	52	87	25864	0.00079	0.18	// GRMZM2G074097 // GRMZM2G401934 // GRMZM2G027663	// AiHT51 // AiHT25 // AiHT77
	GO:0044272	P	sulfur compound biosynthetic process	3	52	111	25864	0.0016	0.32	// GRMZM2G074097 // GRMZM2G401934 // GRMZM2G027663	// AiHT51 // AiHT25 // AiHT77
	GO:0046483	P	heterocycle metabolic process	6	52	714	25864	0.0031	0.57	// GRMZM2G074097 // GRMZM2G051782 // GRMZM5G832772 // GRMZ	// AiHT51 // AiHT05 // AiHT71 // AiHT59 // AiHT25 // AiHT77
	GO:0016811	F	hydrolase activity, acting on carbon-nitrogen (but not peptide) b	2	52	41	25864	0.0034	0.37	// GRMZM2G424857 // GRMZM2G088627	// AiHT10 // AiHT78
	GO:0010266	P	response to vitamin B1	1	52	1	25864	0.004	0.67	// GRMZM2G027663	// AiHT77
	GO:0015431	F	glutathione S-conjugate-exporting ATPase activity	1	52	1	25864	0.004	0.37	// GRMZM5G832772	// AiHT71
	GO:0004789	F	thiamin-phosphate diphosphorylase activity	1	52	1	25864	0.004	0.37	// GRMZM2G401934	// AiHT25
	GO:0010290	F	chlorophyll catabolite transmembrane transporter activity	1	52	1	25864	0.004	0.37	// GRMZM5G832772	// AiHT71
	GO:0008972	F	phosphomethylpyrimidine kinase activity	1	52	1	25864	0.004	0.37	// GRMZM2G401934	// AiHT25
	GO:0008902	F	hydroxymethylpyrimidine kinase activity	1	52	1	25864	0.004	0.37	// GRMZM2G401934	// AiHT25
	GO:0006790	P	sulfur metabolic process	3	52	164	25864	0.0046	0.69	// GRMZM2G074097 // GRMZM2G401934 // GRMZM2G027663	// AiHT51 // AiHT25 // AiHT77
	GO:0018130	P	heterocycle biosynthetic process	3	52	167	25864	0.0048	0.69	// GRMZM2G074097 // GRMZM2G401934 // GRMZM2G027663	// AiHT51 // AiHT25 // AiHT77
	GO:0005777	C	peroxisome	3	52	172	25864	0.0052	0.62	// GRMZM2G088212 // GRMZM2G155220 // GRMZM2G174574	// AiHT43 // AiHT50 // AiHT61
	GO:0042579	C	microbody	3	52	172	25864	0.0052	0.62	// GRMZM2G088212 // GRMZM2G155220 // GRMZM2G174574	// AiHT43 // AiHT50 // AiHT61
	GO:0016462	F	pyrophosphatase activity	7	52	1068	25864	0.0054	0.37	// GRMZM2G063060 // GRMZM2G051782 // GRMZM2G017257 // GRMZ	// AiHT06 // AiHT05 // AiHT31 // AiHT71 // AiHT48 // AiHT14 // AiHT77
	GO:0033273	P	response to vitamin	1	52	2	25864	0.006	0.79	// GRMZM2G027663	// AiHT77
	GO:0004046	F	aminoacylase activity	1	52	2	25864	0.006	0.37	// GRMZM2G088627	// AiHT78
	GO:0080041	F	ADP-ribose pyrophosphohydrolase activity	1	52	2	25864	0.006	0.37	// GRMZM2G027663	// AiHT77
	GO:0016818	F	hydrolase activity, acting on acid anhydrides, in phosphorus-cc	7	52	1092	25864	0.0061	0.37	// GRMZM2G063060 // GRMZM2G051782 // GRMZM2G017257 // GRMZ	// AiHT06 // AiHT05 // AiHT31 // AiHT71 // AiHT48 // AiHT14 // AiHT77
	GO:0016817	F	hydrolase activity, acting on acid anhydrides	7	52	1115	25864	0.0068	0.37	// GRMZM2G063060 // GRMZM2G051782 // GRMZM2G017257 // GRMZ	// AiHT06 // AiHT05 // AiHT31 // AiHT71 // AiHT48 // AiHT14 // AiHT77
	GO:0019438	P	aromatic compound biosynthetic process	3	52	201	25864	0.008	0.92	// GRMZM2G074097 // GRMZM2G401934 // GRMZM2G027663	// AiHT51 // AiHT25 // AiHT77
	GO:0016045	P	detection of bacterium	1	52	3	25864	0.008	0.92	// GRMZM2G027663	// AiHT77
	GO:0003844	F	1,4-alpha-glucan branching enzyme activity	1	52	3	25864	0.008	0.37	// GRMZM2G088753	// AiHT40
	GO:0004591	F	oxoglutarate dehydrogenase (succinyl-transferring) activity	1	52	3	25864	0.008	0.37	// GRMZM2G151041	// AiHT75
	GO:0050897	F	cobalt ion binding	2	52	67	25864	0.0085	0.37	// GRMZM2G088212 // GRMZM2G151041	// AiHT43 // AiHT75
	GO:0007017	P	microtubule-based process	3	52	210	25864	0.009	0.95	// GRMZM2G051782 // GRMZM2G017257 // GRMZM2G385925	// AiHT05 // AiHT31 // AiHT14
	GO:0043229	C	intracellular organelle	24	52	7690	25864	0.0091	0.62	// GRMZM2G074097 // GRMZM2G095511 // GRMZM2G106578 // GRMZ	// AiHT51 // AiHT22 // AiHT20 // AiHT10 // AiHT28 // AiHT34 // AiHT05 // AiHT31 // AiHT71 // AiHT43 // AiHT78 // AiHT14 // AiHT61 // AiHT06 // AiHT76 // AiHT25 // AiHT42 // AiHT52 // AiHT77 // AiHT46 // AiHT50 // AiHT75 // AiHT13 // AiHT35
	GO:0043226	C	organelle	24	52	7692	25864	0.0091	0.62	// GRMZM2G074097 // GRMZM2G095511 // GRMZM2G106578 // GRMZ	// AiHT51 // AiHT22 // AiHT20 // AiHT10 // AiHT28 // AiHT34 // AiHT05 // AiHT31 // AiHT71 // AiHT43 // AiHT78 // AiHT14 // AiHT61 // AiHT06 // AiHT76 // AiHT25 // AiHT42 // AiHT52 // AiHT77 // AiHT46 // AiHT50 // AiHT75 // AiHT13 // AiHT35
	GO:0051187	P	cofactor catabolic process	2	52	70	25864	0.0092	0.95	// GRMZM2G377115 // GRMZM2G151041	// AiHT59 // AiHT75
	GO:0015630	C	microtubule cytoskeleton	3	52	215	25864	0.0096	0.62	// GRMZM2G051782 // GRMZM2G017257 // GRMZM2G385925	// AiHT05 // AiHT31 // AiHT14

*1: The term_types F, P, and C indicate molecular function, biological preocess, and celluer component, respectively.

*2: We set "1" as minimum number of mapping entries, instead of the default value, "5".

*3: We selected "Fisher" test as a statistical method and listed up GO terms showing p-values < 0.01.

*4: We selected "Yekutielli (FDR under dependency)" as multi-test adjustment method.
